# Tumor-promoting function and prognostic significance of the RNA-binding protein T-cell intracellular antigen-1 in esophageal squamous cell carcinoma

**DOI:** 10.18632/oncotarget.7937

**Published:** 2016-03-06

**Authors:** Junichi Hamada, Katsutoshi Shoda, Kiyoshi Masuda, Yuji Fujita, Takuya Naruto, Tomohiro Kohmoto, Yuko Miyakami, Miki Watanabe, Yasusei Kudo, Hitoshi Fujiwara, Daisuke Ichikawa, Eigo Otsuji, Issei Imoto

**Affiliations:** ^1^ Department of Human Genetics, Institute of Biomedical Sciences, Tokushima University Graduate School, Tokushima, 770-8503, Japan; ^2^ Division of Digestive Surgery, Department of Surgery, Kyoto Prefectural University of Medicine, Kyoto, 602-8566, Japan; ^3^ Student Lab, Tokushima University Faculty of Medicine, Tokushima, 770-8503, Japan; ^4^ Department of Oral Molecular Pathology, Institute of Biomedical Sciences, Tokushima University Graduate School, Tokushima, 770-8503, Japan

**Keywords:** T-cell intracellular antigen-1, isoform, oncogene, RNA-binding protein, esophageal squamous cell carcinoma

## Abstract

T-cell intracellular antigen-1 (TIA1) is an RNA-binding protein involved in many regulatory aspects of mRNA metabolism. Here, we report previously unknown tumor-promoting activity of TIA1, which seems to be associated with its isoform-specific molecular distribution and regulation of a set of cancer-related transcripts, in esophageal squamous cell carcinoma (ESCC). Immunohistochemical overexpression of TIA1 ectopically localized in the cytoplasm of tumor cells was an independent prognosticator for worse overall survival in a cohort of 143 ESCC patients. Knockdown of TIA1 inhibited proliferation of ESCC cells. By exogenously introducing each of two major isoforms, TIA1a and TIA1b, only TIA1a, which was localized to both the nucleus and cytoplasm, promoted anchorage-dependent and anchorage-independent ESCC cell proliferation. Ribonucleoprotein immunoprecipitation, followed by microarray analysis or massive-parallel sequencing, identified a set of TIA1-binding mRNAs, including *SKP2* and *CCNA2*. TIA1 increased SKP2 and CCNA2 protein levels through the suppression of mRNA decay and translational induction, respectively. Our findings uncover a novel oncogenic function of TIA1 in esophageal tumorigenesis, and implicate its use as a marker for prognostic evaluation and as a therapeutic target in ESCC.

## INTRODUCTION

Esophageal cancer is the eighth most common cancer worldwide, and the sixth most common cause of death from cancer [1]. Despite the advances in multimodal therapies, the outcome of this disease remains poor [2]. In Asian countries, including Japan, esophageal squamous cell carcinoma (ESCC) lacking accessible genomic characteristics accounts for > 90% of esophageal cancers [3]. The identification of deregulated molecules that lead to unrestrained cell growth and/or cell death evasion during carcinogenesis is needed for the establishment of novel prognostic markers and therapeutic targets to guide individualized treatment of ESCC.

RNA-binding proteins (RBPs) coordinate the lives of mRNAs by contributing to various post-transcriptional modification processes under both physiological and pathological conditions [4, 5]. Given the central role of RBPs in the regulation of gene expression, malfunction of RBP or mutations in the RNA elements they recognize can lead to cancer development and progression. Indeed, several RBPs have been reported to be involved in carcinogenesis by increasing or decreasing expression of various genes [6–8]. In addition, a global comparison of the transcriptional profiles in the Cancer Genome Atlas Pan-Cancer Dataset revealed that a significant change in RBP expression occurs and contributes to the dysregulated transcription of a set of oncogenes and tumor-suppressor genes across most cancer types [9]. However, the significance of RBPs in carcinogenesis remains primarily unknown.

The T-cell intracellular antigen-1 gene (*TIA1*) encodes TIA1 protein, a cytotoxic granule-associated RBP that possesses nucleolytic activity against cytotoxic lymphocyte (CTL) target cells [10]. TIA1 also acts as a stress-induced inhibitor of translation by localizing to stress granules (SGs) with polyA RNA and contributes to the induction of apoptosis in CTL targets [11–13]. The two alternative splice transcripts, *TIA1 variant 1* (*TIA1-v1*, NM_022173.2) and *variant 2* (*TIA1-v2*, NM_022037.2), which lack and include exon 5, respectively, encode two different isoforms, TIA1b (or TIA1 isoform 2, NP_071505.2) and TIA1a (or TIA1 isoform 1, NP_071320.2), respectively [14]. Although several reports refer to the relationship between TIA1 and epithelial carcinogenesis [14], the significance of TIA1 and/or its isoforms in ESCC remains unclear.

In this study, we first report the importance of TIA1, particularly the TIA1a isoform, as a molecule exerting oncogenic activity by ectopically localizing to the cytoplasm and increasing/decreasing the expression of a set of cancer-related genes, including SKP2 and CCNA2, in the malignant progression of ESCC.

## RESULTS

### TIA1 protein expression and its association with the clinicopathological characteristics in ESCC

**Figure 1:**
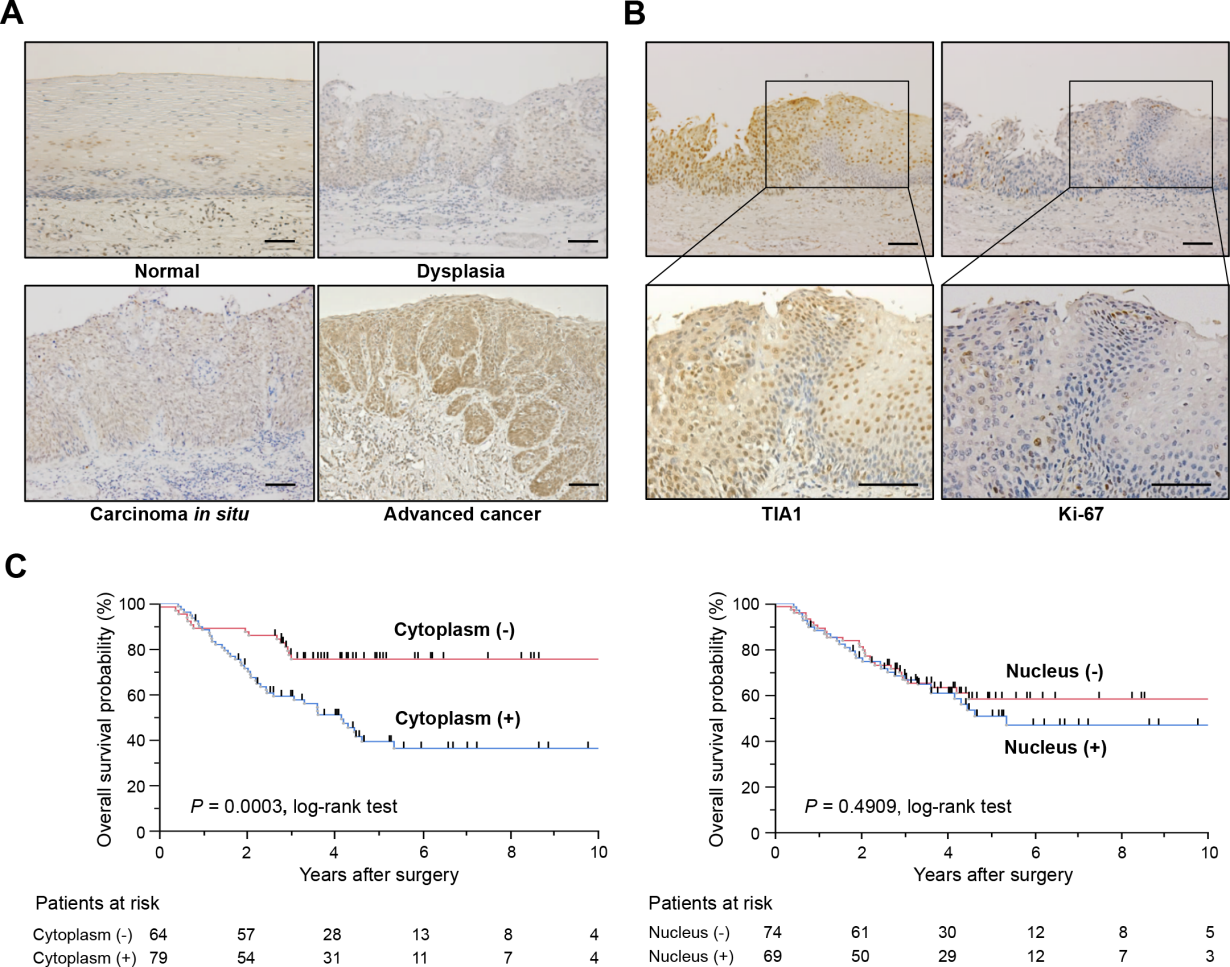
TIA1 protein overexpression and localization and its association with overall survival in primary ESCC tumors (**A**) Representative results of the immunohistochemical detection of TIA1 protein in normal mucosa, dysplasia, carcinoma *in situ* and advanced squamous cell carcinoma of the esophagus. Scale bars: 40 μm. (**B**) Representative results of the immunohistochemical detection of TIA1 and Ki-67 in serial sections of primary ESCC tissues. Scale bars: 40 μm. (**C**) Kaplan–Meier curves for overall survival rates of 143 ESCC patients according to the cytoplasmic (left) and nuclear (right) expression levels of TIA1 protein. The log-rank test was used for statistical analysis. Differences resulting in values of *P* < 0.05 are considered statistically significant.

To determine the clinical significance of TIA1 in esophageal carcinogenesis, immunohistochemical staining (IHC) was performed in surgically resected tissues (Figure 1A). TIA1 immunoreactivity was observed primarily in the nucleus of normal esophageal epithelia and in dysplasic lesions, which displayed higher TIA1 immunoreactivity than the normal mucosa. In carcinoma *in situ* and advanced cancers, TIA1 staining was observed in both the cytoplasm and the nucleus, and cytoplasmic TIA1 immunoreactivity was higher in advanced cancers than in carcinoma *in situ*. Notably, higher immunoreactivity toward Ki-67, a cell proliferation marker, was observed preferentially in tumor cells with higher cytoplasmic TIA1 immunoreactivity (Figure 1B), suggesting that cytoplasmic TIA1 promotes the proliferation of ESCC cells [15, 16]. A similar pattern of TIA1 immunoreactivity was observed in squamous cell carcinomas of other tissues (Supplementary Figure S1A).

We then examined the clinicopathological significance of TIA1 expression in primary ESCC tumors based on the IHC staining pattern (Supplementary Figure S1B). Among 143 cases, positive cytoplasmic and nuclear TIA1 immunoreactivities were observed in 79 (55.2%) and 69 (48.3%) patients, respectively, based on their intensity scores (Table 1). Because positive cytoplasmic and nuclear TIA1 immunoreactivities were detected at the same levels between patients with and without neoadjuvant chemotherapy with 5-fluorouracil plus cisplatin (FP, Supplementary Tables S1 and S2), we combined all patients for further analyses. No significant association was observed between any clinicopathological factors and nuclear or cytoplasmic TIA1 immunoreactivity (Table 1). Kaplan–Meier survival estimates showed that positive cytoplasmic TIA1 immunoreactivity was significantly associated with worse overall survival in all 143 cases (*P* = 0.0003), but nuclear TIA1 immunoreactivity was not (Figure 1C). No synergistic effect between positive cytoplasmic and nuclear TIA1 immunoreactivities on overall survival was observed even after dividing ESCC cases into four groups according to both cytoplasmic and nuclear TIA1 staining patterns (Supplementary Figure S1C). In the Cox proportional hazards regression model, cytoplasmic TIA1 immunoreactivity, lymphatic invasion, venous invasion, pT and pN categories, and preoperative therapy procedures were statistically significant prognosticators for overall survival by univariate analyses (Table 2). Multivariate analyses showed that cytoplasmic TIA1 immunoreactivity and pT and pN categories were independent predictive factors regardless of the models used (Table 2), suggesting that overexpressed TIA1 is involved in the development and progression of ESCC through cytoplasmic localization.

**Table 1:** Association between clinicopathological characteristics and TIA1 expression

Clinicopatho logical factors	*n*	TIA1 immunoreactivity (Cytoplasm)		*P* value^a^	TIA1 immunoreactivity (Nucleus)		*P* value^a^	TIA1 immunoreactivity (Whole)		*P* value^a^
		Positive (%)	Negative (%)		Positive (%)	Negative (%)		Positive (%)	Negative (%)	
Total	143	79 (55.2)	64 (44.8)		69 (48.3)	74 (51.7)		67 (46.9)	76 (53.1)	
Gender										
Male	117	69 (59.0)	48 (41.0)	0.0574	53 (45.3)	64 (54.7)	0.1329	57 (48.7)	60 (51.3)	0.7523
Female	26	10 (38.5)	16 (61.5)		16 (61.5)	10 (38.5)		10 (38.5)	16 (61.5)	
Age										
Mean ± SD (yr)	63.2 ± 7.3	62.8 ± 7.5	63.7 ± 6.9	0.4047	62.7 ± 6.5	63.7 ± 7.9	0.5228	62.7 ± 6.59	63.6 ± 7.84	0.4924
Location^b^										
Upper	27	12 (44.4)	15 (55.6)	0.1526	14 (51.9)	13 (48.1)	0.5404	12 (44.4)	15 (55.6)	0.9272
Middle	69	36 (52.2)	33 (47.8)		30 (43.5)	39 (56.5)		32 (46.4)	37 (53.6)	
Lower	47	31 (66.0)	16 (34.0)		25 (53.2)	22 (46.8)		23 (48.9)	24 (51.1)	
Histopathological grading^c^										
Well and moderately differentiated	96	55 (57.3)	41 (42.7)	0.4822	46 (47.9)	51 (53.1)	0.6378	44 (45.8)	52 (54.2)	0.7270
Poorly	47	24 (51.1)	23 (48.9)		24 (51.1)	23 (48.9)		23 (48.9)	24 (51.1)	
Size										
Mean ± SD (mm)	45.2 ± 26.9	46.5 ± 26.2	43.7 ± 27.7	0.4189	46.2 ± 27.2	44.4 ± 26.7	0.6248	47.2 ± 27.7	43.5 ± 26.1	0.4675
Lymphatic invasion (ly)										
Negative	68	44 (64.7)	31 (45.6)	0.3873	33 (48.5)	35 (51.5)	0.9496	32 (47.1)	36 (52.9)	0.9626
Positive	75	35 (51.5)	33 (48.5)		36 (52.9)	39 (57.4)		35 (46.7)	40 (53.3)	
Venous invasion (v)										
Negative	88	32 (36.4)	23 (26.1)		43 (48.9)	45 (51.1)	0.8530	42 (47.7)	46 (52.3)	0.7910
Positive	55	47 (53.4)	41 (46.6)	0.5762	26 (29.5)	29 (33.0)		25 (45.5)	30 (54.5)	
Depth of tumor invasion (pT)										
pT1	55	26 (47.3)	29 (52.7)	0.1298	25 (45.5)	30 (54.5)	0.5965	25 (45.5)	30 (54.5)	0.7910
pT2-4	88	53 (60.2)	35 (39.8)		44 (50.0)	44 (50.0)		42 (47.7)	46 (52.3)	
N stage (pN)										
pN0	51	26 (51.0)	25 (49.0)	0.4455	23 (45.1)	28 (54.9)	0.5739	22 (43.1)	29 (56.9)	0.5069
pN1-3	92	53 (57.6)	39 (42.4)		46 (50.0)	46 (50.0)		45 (48.9)	47 (51.1)	
pStage										
pI	36	21 (58.3)	15 (41.7)	0.6660	15 (41.7)	21 (58.3)	0.3596	17 (47.2)	19 (52.8)	0.9591
pII-VI	107	58 (54.2)	49 (45.8)		54 (50.5)	53 (49.5)		50 (46.7)	57 (53.3)	
Neoadjuvant therapy^d^										
Absent	78	47 (60.3)	31 (39.7)	0.1866	37 (47.4)	41 (52.6)	0.8306	40 (51.3)	38 (48.7)	0.2444
Present	65	32 (49.2)	33 (50.8)		32 (49.2)	33 (50.8)		27 (41.5)	38 (58.5)	

Statistically significant value are in boldface type.

a *P* value are from *χ*^2^ or Fisher's exact test and were statistically significant at < 0.05.

b Upper, cervical + upper thoracic esophagus; Middle, mid-thoracic esophagus; Lower, lower thoracic + abdominal esophagus.

c Well, well differentiated SCC; Moderate, moderate differentiated SCC; Poorly, poorly differentiated SCC.

d Neoadjuvant therapy, preoperative treatment with FP [5-fluorouracil (800 mg/body/day) plus cisplatin (80 mg/body/day)].

**Table 2:** Cox proportional hazard regression analysis for overall survival

	Univariate			Multivariate^a^		
				Model 1	Model 2	Model 3
	Hazard ratio	95% confidence interval	*P* value	*P* value	*P* value	*P* value
Gender						
Male versus Female	1.04	0.5502−2.1854	0.9058	0.7848	–	–
Age						
≥ 65 yr versus < 65 yr	1.07	0.6335−1.7892	0.8029	0.6317	–	–
Histopathological grading						
Poorly versus Well-moderately differentiated	1.16	0.6758−1.9660	0.5733	0.6501	–	–
Size						
≥ 40 versus < 40	1.64	0.9798−2.7823	0.0597	0.4626	–	–
Lymphatic invasion						
Positive versus Negative	2.06	1.2200−3.5765	**0.0066**	0.1544	0.1912	–
Venous invasion						
Positive versus Negative	1.69	1.0044−2.8334	**0.0481**	0.8674	0.9900	–
Depth of tumor invasion (pT)						
pT2-3 versus pT1	3.91	2.0659−8.2066	**< 0.0001**	**0.0192**	**0.0195**	**0.0044**
N stage (pN)						
pN1-3 versus pN0	4.70	2.3546−10.7289	**< 0.0001**	**0.0013**	**0.0020**	**0.0007**
Neoadjuvant chemotherapy						
Positive versus Negative	1.84	1.0893−3.1287	**0.0226**	0.4305	0.2573	–
TIA1 immunoreactivity in the cytoplasm						
Positive versus Negative	2.84	1.1619−5.2878	**0.0002**	**0.0059**	**0.0033**	**0.0024**
TIA1 immunoreactivity in the nucleus						
Positive versus Negative	1.20	0.7149−2.0146	0.4913	**-**	**–**	**–**

Note: Statistically significant value are in boldface type.

a Model 1, all factors excluding TIA1 immunoreactivity in the nucleus model were included; Model 2, factors whose *p*-value < 0.05 in the univariate analysis were included; Model 3, a step-wise procedure was used.

### Expression of TIA1 in ESCC cell lines

**Figure 2:**
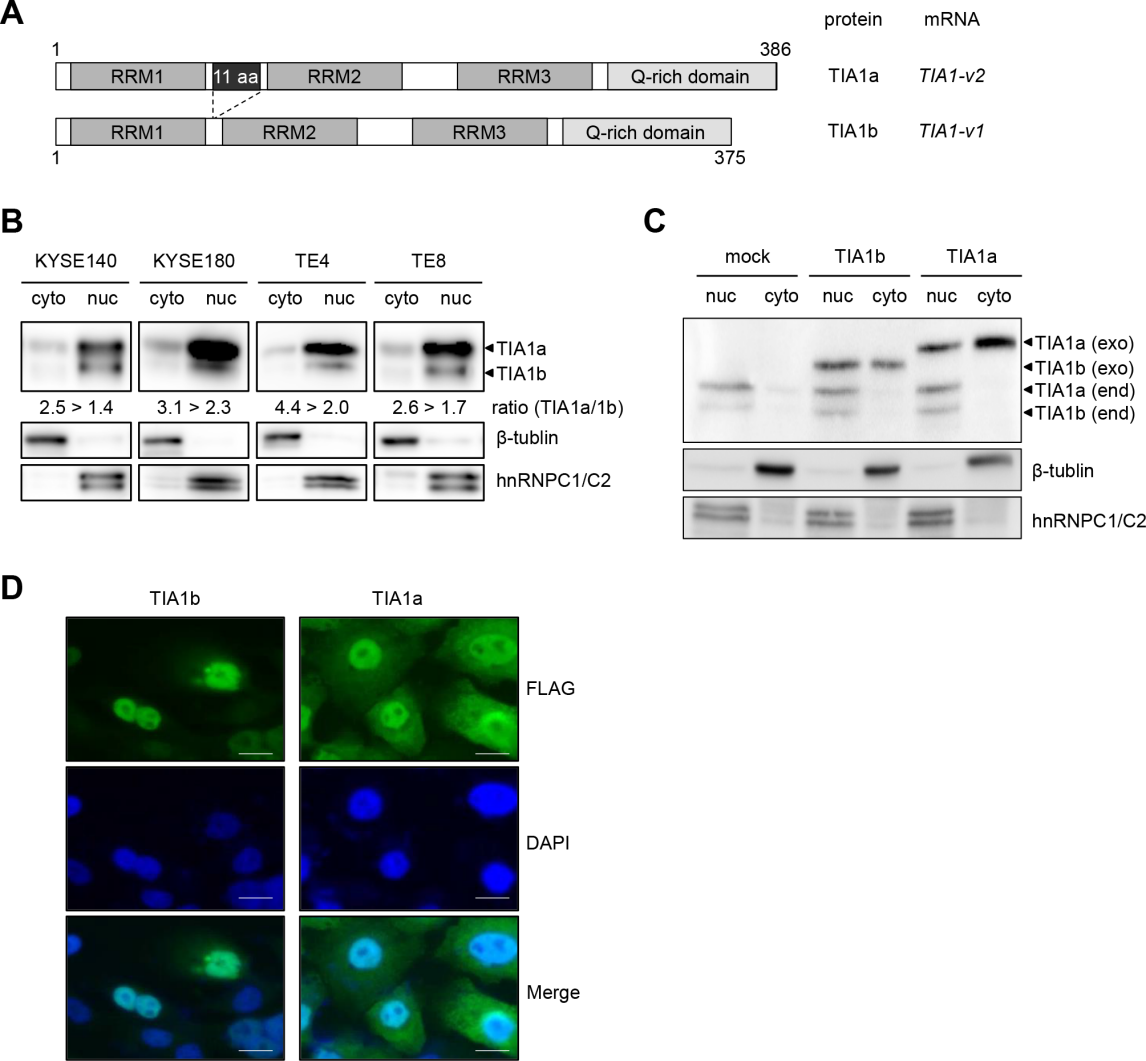
Subcellular distribution of the TIA1 isoforms (**A**) Schematic structures of the TIA1a and TIA1b protein isoforms with and without 11 amino acids, translated from the *TIA1-v2* and *TIA1-v1* transcripts, respectively. Both isoforms include three RNA recognition motifs (RRM) and a carboxyl-terminal glutamine-rich domain (Q-rich domain). Numbers indicate amino acid residues corresponding to each TIA1 isoform. (**B**) Subcellular distribution of endogenous TIA1 in ESCC cells. Cytoplasmic and nuclear fractions were prepared from KYSE140, KYSE180, TE4 and TE8 cells. Amounts of TIA1, β-tubulin (cytoplasmic marker) and hnRNPC1/C2 (nuclear marker) were measured by western blot. The intensities of specific bands corresponding to the TIA1 isoforms were measured with a densitometer and are presented as ratios in the inset. (**C**) The subcellular distribution of exogenously expressed TIA1 isoforms. Cytoplasmic and nuclear fractions were prepared from KYSE2270 cells stably transfected with mock-, TIA1a- or TIA1b-expressing constructs. Amounts of exogenous (exo) and endogenous (end) TIA1 isoforms (arrowheads), β-tubulin (cytoplasmic marker) and hnRNPC1/C2 (nuclear marker) were measured by western blot analysis. (**D**) Representative images of exogenously expressed FLAG-tagged TIA1 isoforms in KYSE2270 cells detected by FIC using anti-FLAG antibody (green). Nuclei were counterstained with DAPI (blue). Scale bars: 20 μm.

*TIA1* mRNA overexpression, compared with the esophagus, was also detected in 30 of 45 ESCC cell lines by quantitative real-time PCR (qPCR, Supplementary Figure S2A). Similarly, TIA1 protein overexpression was observed in most of cancer cells compared with normal mucosa (Supplementary Figure S2B). The human *TIA1* gene generates two major variants (*v1* and *v2*) through alternative splicing of exon 5, encoding the shorter TIA1b and longer TIA1a isoforms, respectively (Figure 2A). TIA1 contains three domains with RNA recognition motifs (RRMs), and the 11 amino acids encoded by exon 5 are located between RRM1 and RRM2. Most ESCC cells constitutively express *TIA1-v2* mRNA and a small amount of *TIA1-v1* mRNA (Supplementary Figure S3A), resulting in the predominant expression of TIA1a protein compared with TIA1b protein (Supplementary Figure S2B). Similarly, both non-tumor and tumor tissues of primary ESCC predominantly expressed *TIA1-v2* mRNA, and the *TIA1* mRNA expression levels in tumors were higher than in those in paired non-tumor tissues in 3/6 (50%) of ESCC cases whose RNA was available (Supplementary Figure S3B). Western blot analysis using subcellular components obtained by cell fractionation showed that endogenous TIA1b was detected primarily in the nuclear lysate, whereas endogenous TIA1a was detected in both nuclear and cytoplasmic lysates, although most TIA1a was located in the nucleus (Figure 2B). Exogenously expressed TIA1b protein in KYSE2270 cells with lower endogenous TIA1 expression localized predominantly to the nucleus, while a larger fraction of the exogenously expressed TIA1a protein localized to the cytoplasm compared with TIA1b protein, as demonstrated by western blot analysis (Figure 2C) and by fluorescent immunocytochemical staining (FIC, Figure 2D).

### Involvement of TIA1 in ESCC cell proliferation

**Figure 3:**
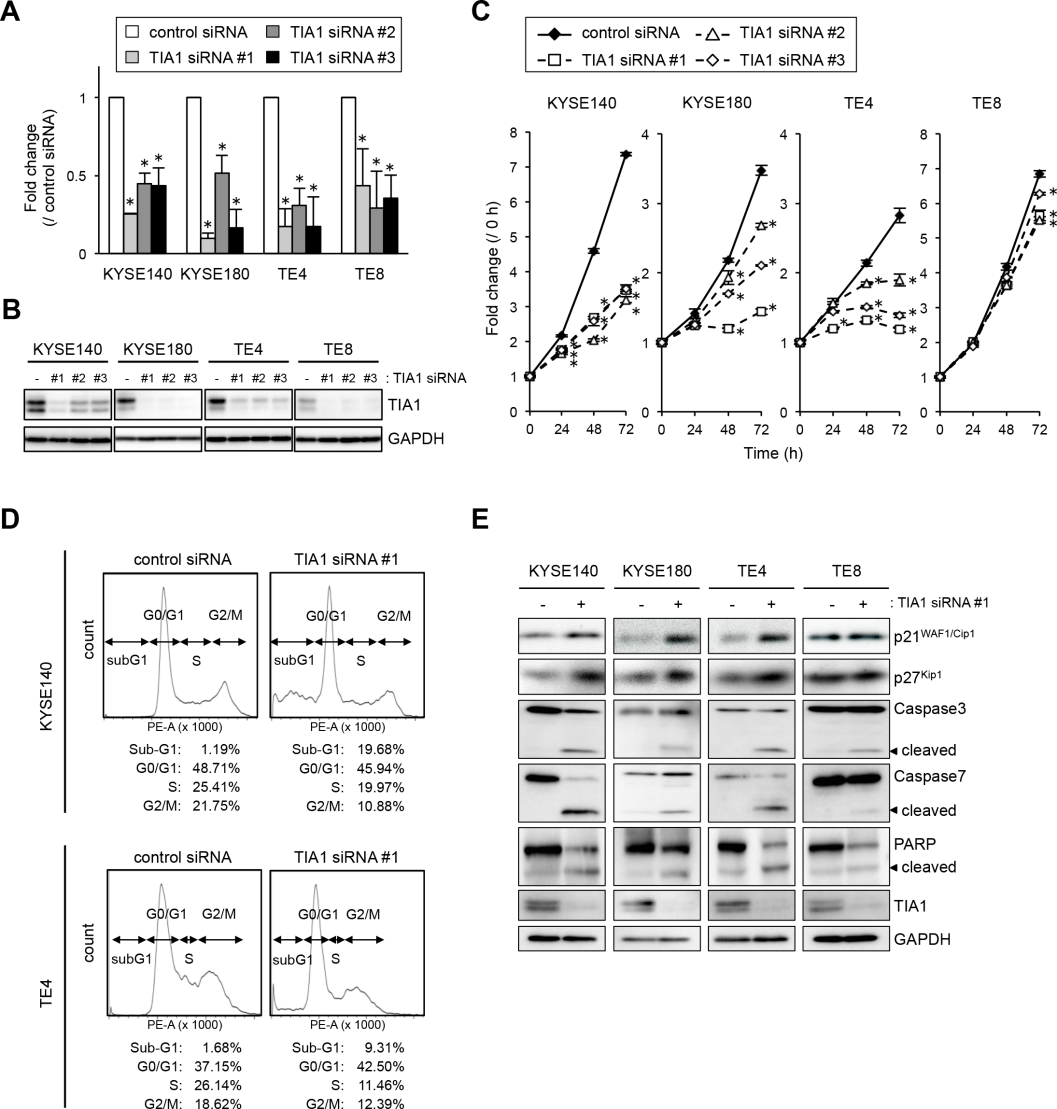
Effects of TIA1 knockdown on cell proliferation (**A**) KYSE140, KYSE180, TE4 or TE8 cells were transfected with each 10 nM TIA1-specific or control siRNA for 48 h, and the *TIA1* mRNA expression levels were evaluated by qPCR using *GAPDH* mRNA as an endogenous control. The values are expressed as fold changes (mean ± SD, *n* = 3) compared with the respective values in control siRNA-transfected cells. *significantly different from the control value by Student's *t* test (*P* < 0.05). (**B**) Effectiveness of siRNA transfection. KYSE140, KYSE180, TE4 or TE8 cells were treated as described in (A), and the expression levels of TIA1 protein were evaluated by western blot analysis using GAPDH as a loading control. (**C**) KYSE140, KYSE180, TE4 or TE8 cells were transfected with each 10 nM TIA1-specific or control siRNA for 24 h, and cellular proliferation was measured using a WST assay at the indicated times. The values are expressed as fold changes (mean ± SD, *n* = 4) compared with the respective values in control cells (0 h). *significantly different from the control value by Student's *t* test (*P* < 0.05). (**D**) Representative results of cell cycle analysis. After KYSE140 or TE4 cells were treated with 10 nM TIA1-specific or control siRNA for 48 h, then the cells were stained with PI and subjected to FACS analysis. Raw data were quantified for cell cycle analysis by FACSVerse software. (**E**) KYSE140, KYSE180, TE4 and TE8 cells were transfected with 10 nM TIA1-specific or control (-) siRNA for 48 h. Levels of caspase-3, caspase-7, PARP, p21^WAF1/Cip1^, p27^Kip1^ and TIA1 proteins were measured by western blot analysis using GAPDH as a loading control.

To gain insight into the potential function of TIA1 whose overexpression could be associated with esophageal carcinogenesis, we first tested the effects of small interfering RNA (siRNA) targeting TIA1 on cell proliferation. By silencing endogenous TIA1 using three different siRNAs, cell proliferation was significantly suppressed in KYSE140, KYSE180 and TE4 cells. In TE8 cells, which express lower levels of TIA1, treatment with *TIA1*-specific siRNAs led to minimal suppression (Figure 3A–3C). FIC demonstrated that Ki-67 expression correlated with TIA1 expression; cells that retained TIA1 expression tested positive for Ki-67 expression, whereas cells in which TIA1 was effectively knocked down tested negative for Ki-67 expression (Supplementary Figure S4A). Using fluorescence-activated cell sorting (FACS) analysis to examine the mode of action of TIA1 in the cell cycle, an accumulation of cells in G_0_–G_1_ phase, with an accompanying increase in sub-G_1_ phase cells and a decrease in S and G_2_–M phase cells, was observed in *TIA1* siRNA-treated cells compared with control siRNA-treated cells (Figure 3D). Knockdown of endogenous TIA1 significantly increased p21^WAF1/Cip1^ and p27^Kip1^ protein levels, with extensive cleavage of caspase-3, caspase-7 and poly [ADP ribose] polymerase (PARP), which are markers of apoptosis (Figure 3E). These results suggest that TIA1 silencing in ESCC cells contributes to cell cycle arrest at the G_1_-S checkpoint and the induction of apoptosis.

**Figure 4:**
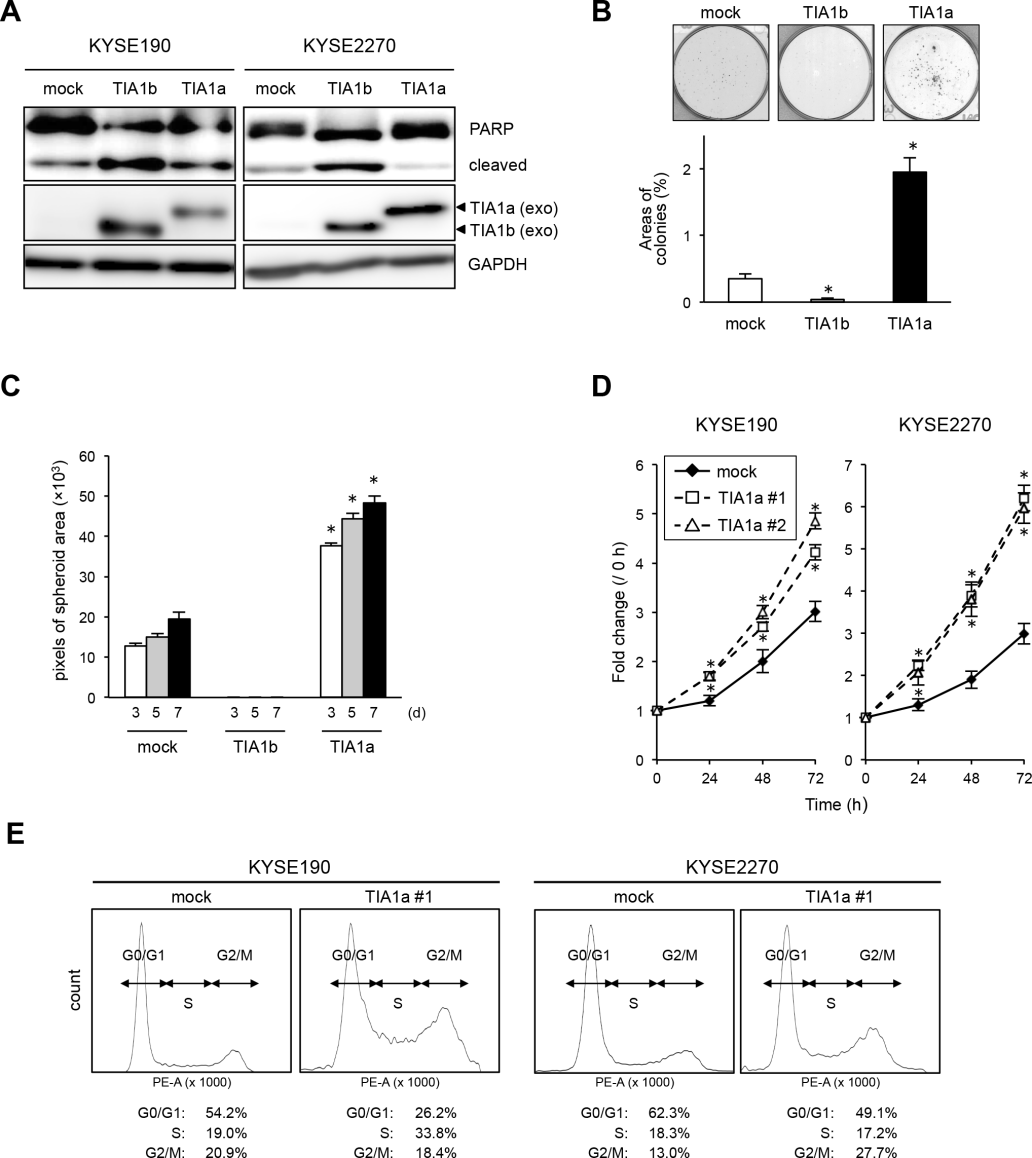
TIA1a promotes cell proliferation in ESCC cells (**A**) KYSE190 and KYSE2270 cells were transiently infected with a mock, TIA1a-, or 1b-expressing retroviruses, and the levels of PARP and exogenous TIA1 (exo) isoforms were measured by western blot analysis using GAPDH as a loading control. (**B**) KYSE190 cells transiently infected with either a mock-, pTIA1[v1]-FLAG- or pTIA1[v2]-FLAG-expressing retrovirus (1,000 cells/well) were plated in six-well plates and treated with 0.5 mg/mL G418 for two weeks. The colonies in each well were stained with crystal violet, and the colony areas were calculated using ImageJ software. *significantly different from the control value by Student's *t* test (*P* < 0.05). (**C**) For spheroid formation assay, KYSE190 cells transiently transfected with mock or each TIA1 isoform were seeded in ultra-low attachment 96-well round bottom plates and incubated at 37°C for the indicated times (d, days). The areas of spheroids were determined as described in the Materials and Methods section (mean ± SD, *n* = 8). *significantly different from the control value by Student's *t* test (*P* < 0.05). (**D**) The number of viable cells of each stable transfectant was assessed using a WST assay for the indicated times. The values are expressed as fold changes (mean ± SD, *n* = 4) compared with the respective control values (0 h). *, significantly different from the control value by Student's *t* test (*P* < 0.05). (**E**) Representative results of FACS analysis using stable transfectants. The raw data were quantified for cell cycle analysis using FACSVerse software.

We next introduced a TIA1[v1]- or TIA1[v2]-expressing retrovirus into KYSE190 and KYSE2270 cells expressing relatively lower levels of endogenous TIA1 to determine the effects of exogenously expressed TIA1 isoforms. Notably, PARP cleavage was pronounced only in TIA1b-overexpressing cells (Figure 4A). Colony formation assays revealed that TIA1a-expressing cells formed more colonies than control and TIA1b-expressing cells (Figure 4B). Anchorage-independent *in vitro* 3D cell culture showed that exogenously expressed TIA1a accelerated spheroid formation, while exogenously expressed TIA1b inhibited spheroid formation (Figure 4C). Furthermore, higher Ki-67 expression was observed in cells exogenously expressing TIA1a, compared with cells lacking exogenous TIA1a expression (Supplementary Figure S4B). These results indicate the distinct functions of TIA1a and TIA1b in ESCC cells: TIA1a promotes anchorage-dependent and anchorage-independent cell proliferation, while TIA1b does not. These distinct functions of two isoforms were partially supported by knockdown experiments using isoform-specific siRNAs even though these siRNAs showed limited specificity and efficiency (Supplementary Figure S5A). In comparison with depletion of TIA1b, anchorage-dependent cell proliferation was more effectively suppressed by depletion of TIA1a with an increased p21^WAF1/Cip1^ protein level and extensive cleavage of PARP (Supplementary Figure S5A, S5B).

To determine the chronic effects of each TIA1 isoform on ESCC cell proliferation *in vitro*, we established stable transfectants expressing FLAG-tagged TIA1a or TIA1b protein using KYSE190 and KYSE2270 cells. Stable expression of TIA1a increased cell proliferation compared with control cells (Figure 4D). Consistent with the observations in the transient transfection experiments, stable expression of TIA1b facilitated cell death in most cell lines during isolation and subculture after transfection of the construct. FACS analysis indicated an accumulation of cells in S and G_2_–M phase and a decrease in G_0_–G_1_ phase cells in TIA1a-expressing cells compared with control cells (Figure 4E). Taken together, these results suggest that TIA1a contributes to the anchorage-dependent and anchorage-independent growth of ESCC cells *in vitro*.

### Identification of putative target mRNAs for cytoplasmic TIA1

**Figure 5:**
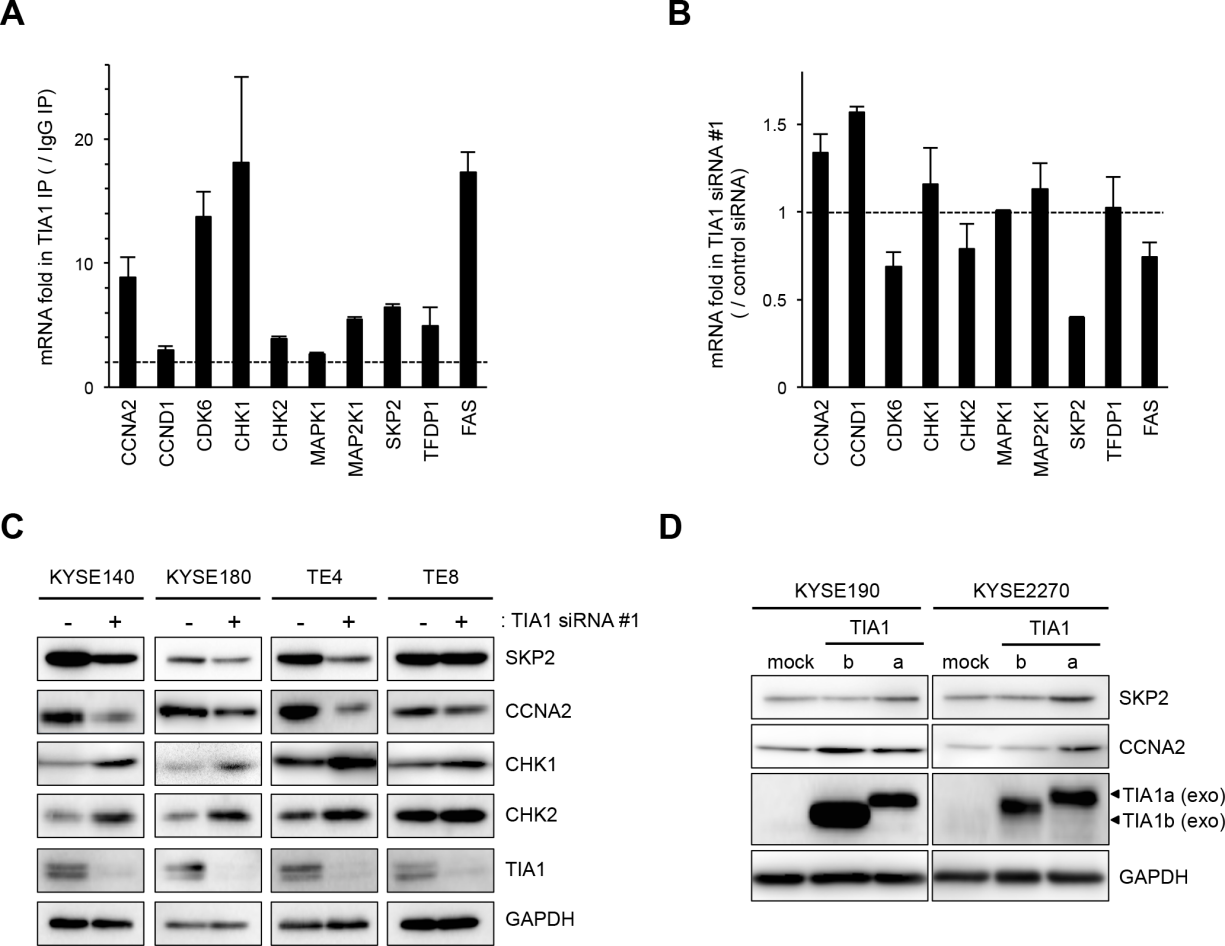
Binding of TIA1 with mRNAs of putative target mRNAs and its effect on protein levels in ESCC cells (**A**) Bindings between TIA1 and the nine putative target transcripts encoding cell cycle regulatory proteins identified through RIP-chip and RIP-seq (Supplementary Table S5) using anti-TIA1 antibody, as well as the known target, *FAS* mRNA, were validated by qPCR after RIP using KYSE180 cells. TIA1-mRNA bindings were measured by RIP followed by qPCR amplification and expressed as the enrichment of individual mRNAs in the TIA1 IP relative to an IgG IP. The data were normalized to the levels of *GAPDH* mRNA, an abundant mRNA that is not a target of TIA1 and that is present as a low-level co-precipitated contaminant in all IP samples. Representative results (mean ± SD, *n* = 3) of four independent experiments are shown. (**B**) Effects of TIA1 silencing on the expression of putative target mRNAs in ESCC cells. KYSE180 cells were transfected with 10 nM TIA1-specific or control siRNA for 48 h. The amounts of the 10 target mRNAs were measured by qPCR using *GAPDH* mRNA as an endogenous control. The values are expressed as fold changes (mean ± SD, *n* = 3) compared with the respective values in control siRNA-transfected cells. (**C**) Effects of TIA1 silencing on the expression of proteins encoded by the putative target genes in ESCC cells. KYSE140, KYSE180, TE4, or TE8 cells were transfected with 10 nM TIA1-specific or control (−) siRNA for 48 h. The levels of SKP2, CCNA2, CHK1, CHK2 and TIA1 proteins were measured by western blot analysis using GAPDH as a loading control. (**D**) Effects of exogenous overexpression of each TIA1 isoform on the expression of proteins encoded by putative target genes in ESCC cells. Lysates were prepared from KYSE190 or KYSE2270 cells overexpressing TIA1. The levels of SKP2, CCNA2 and exogenous TIA1 proteins were measured by western blot analysis using GAPDH as a loading control.

We further investigated the molecular mechanisms of cytoplasmic TIA1-promoted ESCC cell growth. Cytoplasmic TIA1 was shown to bind to transcripts through their 5′ and/or 3′ untranslated regions (UTRs) and to alter their localization, stability and/or translation [11, 16–21]. Therefore, we screened putative TIA1-binding mRNAs using ribonucleoprotein immunoprecipitation (RIP), which was conducted using lysates prepared from the cytoplasm and anti-TIA1 antibody, followed by microarray analysis (RIP-chip) or massive-parallel sequencing (RIP-seq) in KYSE180 cells. We identified 2,679 mRNAs whose enrichment in TIA1 immunoprecipitates differed by > 2.5-fold compared with that of *GAPDH* mRNA in both RIP-chip and RIP-seq (Supplementary Table S3). Gene Ontology (GO) analysis using these 2,679 mRNAs ranked ‘mitotic cell cycle’ (*P* = 6.04E–14) and ‘cell cycle’ (*P* = 4.23E–11) as the two top biological processes (Supplementary Table S4). Pathway analysis using the Kyoto Encyclopedia of Genes and Genomes (KEGG) database with 2,679 mRNAs also ranked ‘cell cycle’ (*P* = 1.14E–14) as the most significant functional pathway (Table 3). Of the top 207 transcripts annotated as ‘cell cycle’ by GO analysis (Supplementary Table S5), we focused on nine well-known cell cycle regulators (*CCNA2*, *CCND1*, *CDK6*, *CHK1*, *CHK2*, *MAPK1*, *MAP2K1*,*SKP2*, and *TFDP1*) for further analyses, due to their known functions in cancers. The binding of these genes with TIA1 protein was successfully validated by RIP followed by qPCR; their affinities were similar to the known TIA1-binding transcript *FAS* mRNA (Figure 5A and Supplementary Figure S6A) [22]. The binding of each TIA1 isoform to these candidate targets was assessed using exogenously expressed each FLAG-tagged TIA1 isoform in HEK293 cells (Supplementary Figure S7A). TIA1a showed more potent binding to each mRNA than TIA1b, although both isoforms bound to all of the mRNAs we analyzed (Supplementary Figure S7B).

**Table 3:** Functional pathways of TIA1 target genes

KEGG Pathway Term	*P* value (FDR)
hsa04110 Cell cycle	1.14E–04
hsa03040 Spliceosome	4.37E–04
hsa04722 Neurotrophin signaling pathway	0.006
hsa04114 Oocyte meiosis	0.031

To investigate the consequences of the binding between TIA1 and these nine genes in ESCC cells, the effects of TIA1 knockdown on the levels of these mRNAs and *FAS* mRNA were examined. TIA1 knockdown did not significantly influence the levels of most of these mRNAs, although *SKP2* mRNA levels decreased moderately (Figure 5B and Supplementary Figure S6B). In those nine genes, the effects of TIA1 knockdown on the levels of the proteins encoded by the *SKP2*, *CCNA2*, *CHK1* and *CHK2* mRNAs were assessed. Compared with control siRNA-treated cells, *TIA1* siRNA-treated KYSE140, KYSE180 and TE4 cells showed lower levels of SKP2 and CCNA2 proteins and higher levels of CHK1 and CHK2 proteins (Figure 5C). Since SKP2 and CCNA2 are known to be implicated in the pathogenesis of various cancers as oncogenes (23–27), the effects of each TIA1 protein isoform on the SKP2 and CCNA2 protein levels were investigated using cells transiently expressing each isoform *via* retroviral infection. Cells exogenously expressing TIA1a showed higher levels of SKP2 and CCNA2 protein compared with control cells. In contrast, cells exogenously expressing TIA1b did not show any increase in SKP2 protein, although KYSE190 cells expressing TIA1b showed higher levels of CCNA2 protein (Figure 5D). The increased *SKP2* mRNA level was observed in cells exogenously expressing TIA1a but not in cells exogenously expressing TIA1b, whereas the *CCNA2* mRNA level was not changed in either cells exogenously expressing TIA1a or TIA1b (Supplementary Figure S6C).

### Binding and functional association between TIA1 and cancer-associated mRNAs

**Figure 6:**
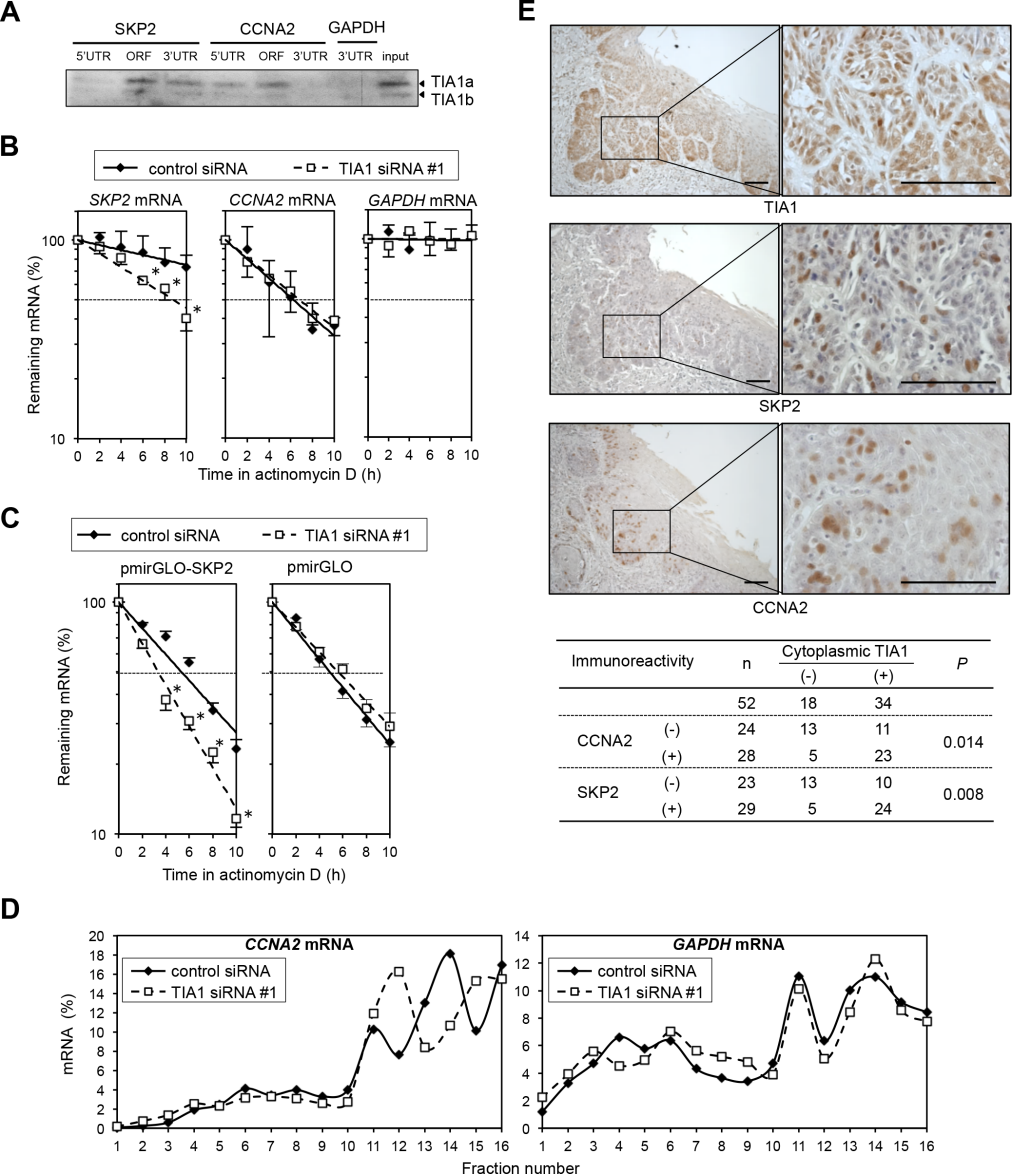
Differing effects of TIA1 on the *SKP2* and *CCNA2* genes in ESCC cells (**A**) Biotinylated transcripts from the *SKP2* 5′ UTR, the *SKP2* ORF, the *SKP2* 3′ UTR, the *CCNA2* 5′ UTR, the *CCNA2* ORF and the *CCNA2* 3′ UTR, as well as a biotinylated fragment of the *GAPDH* 3′ UTR (negative control), were prepared. Bindings between TIA1 and each fragment were tested by biotin pulldown assays using cell lysates extracted from KYSE180 cells. The amounts of TIA1 in pulldown samples were determined by western blot analysis using an anti-TIA1 antibody. Similar results were obtained in three independent experiments. (**B**) Effects of TIA1silencing on the stability of *SKP2* and *CCNA2* mRNA were determined in KYSE180 cells transfected with control siRNA or TIA1 siRNA. After treatment with 2 μg/mL actinomycin D, the amounts of *SKP2* (left), *CCNA2* (middle) and *GAPDH* (right) mRNAs in cells were measured by qPCR and normalized to *18S* rRNA levels. The data (mean ± SD, *n* = 3) are expressed as percentages of *SKP2*, *CCNA2* or *GAPDH* mRNA levels before exposure to actinomycin D (time 0). *significantly different from the control value by Student's *t* test (*P* < 0.05). (**C**) TIA1 increases the stability of *SKP2* mRNA through its 3′ UTR. The pmirGLO-SKP2 plasmid expressing the chimeric reporter transcript bearing the *Luc2* CDS linked to the *SKP2* 3′ UTR or control plasmid (pmirGLO) was co-transfected with control siRNA or TIA1 siRNA into KYSE180 cells for 48 h. After treatment with 2 μg/mL actinomycin D, the amounts of *Luc2* mRNA was measured by qPCR and normalized to *18S* rRNA levels. Data (mean ± SD, *n* = 3) are expressed as percentages of *luciferase* mRNA level before exposure to actinomycin D (time 0). *significantly different from the control value by Student's *t* test (*P* < 0.05). (**D**) Relative polyribosome distribution of the *CCNA2* mRNA (left) and the housekeeping *GAPDH* mRNA (right) in TIA1 knockdown cells and control KYSE180 cells was analyzed by sucrose gradient fractionation. From left to right, percentages of the total mRNA in fractions lacking ribosomes or ribosome subunits (fractions 1 and 2), fractions containing ribosome subunits or single ribosomes (fractions 3 to 7), and fractions spanning polysomes of increasing molecular weights (fractions 8 to 16) were shown (see Supplementary Figure S9C). Representative data are from three independent experiments. (**E**) IHC detection of TIA1, SKP2 and CCNA2 in primary ESCC tissues. Serial sections of ESCC were subjected to immunohistochemistry with goat anti-TIA1 (top), rabbit anti-SKP2 (middle) or mouse monoclonal anti-CCNA2 (lower) antibodies. Scale bars: 40 μm.

The binding between TIA1 protein and *SKP2* and *CCNA2* mRNAs was further analyzed by pulldown assays using biotinylated partial *SKP2* and *CCNA2* mRNA fragments spanning the 5′ UTR, open reading frame (ORF), or 3′ UTR in cytoplasmic lysates prepared from KYSE180 cells (Figure 6A). *SKP2* mRNA bound to TIA1 through its ORF and 3′ UTR, while *CCNA2* mRNA bound to TIA1 through its 5′ UTR and ORF. Previous studies showed that the binding of RBPs with the 5′ UTRs of mRNAs alters their translation, while their binding with the 3′ UTRs of mRNAs changes the stability of these transcripts [16, 17]. In an mRNA decay assay using KYSE180 cells, TIA1 knockdown decreased the stability of *SKP2* mRNA but did not alter that of *CCNA2* mRNA (Figure 6B). Stable overexpression of TIA1a increased the stability of *SKP2* mRNA in KYSE190 and KYSE2270 cells (Supplementary Figure S8A). In an mRNA decay assay using KYSE140 and KYSE180 cells transfected with reporter constructs containing the coding sequence (CDS) of the *firefly luciferase* gene (*Luc2*) with the 3′ UTR of *SKP2* or *Luc2* alone, TIA1 knockdown decreased the *Luc2* mRNA expression level (Figure 6C and Supplementary Figure S8B). In an mRNA decay assay using cells transfected with the expression construct containing FLAG-tagged SKP2 CDS, on the other hand, TIA1 knockdown did not change the FLAG-tagged *SKP2* mRNA expression level (Supplementary Figure S8C). Those results suggest that TIA1 increases the *SKP2* mRNA stability through binding to its 3′ UTR. In ESCC cells, TIA1 depletion did not affect the change of exogenously expressed FLAG-tagged CCNA2 protein level after addition of cycloheximide (Supplementary Figure S9A). In the polysome profiling assay using KYSE180 cells, TIA1 depletion caused a shift in *CCNA2* mRNA from heavy to light polysomal fractions (Figure 6D and Supplementary Figure S9B). Those results suggest that TIA1 reduction induced decreased translation initiation of *CCNA2* mRNA without the stability of CCNA2 protein, although binding site of TIA1 to *CCNA2* mRNA necessary for this translation promoting effect remains undetermined.

The correlation between the TIA1 and SKP2/CCNA2 protein levels was tested in ESCC tumors. In 52 tumors for which serial sections were available, IHC revealed that the expression levels of SKP2 and CCNA2 proteins in tumor cells correlated positively with the cytoplasmic TIA1 expression level (Figure 6E) and tended to be associated with worse overall survival (Supplementary Figure S10A and S10B), although CCNA2 immunoreactivity did not reach a statistically significant level. In addition silencing of either SKP2 or CCNA2 in stable TIA1a-transfectants inhibited anchorage-dependent and anchorage-independent cell proliferations (Supplementary Figure S11). These results suggest that overexpressed cytoplasmic TIA1 promotes ESCC tumorigenesis at least partly through its binding with *SKP2* and *CCNA2* mRNAs and induction of SKP2 and CCNA2 protein overexpression.

## DISCUSSION

To our knowledge, this study is the first to demonstrate the clinical and functional significance of the TIA1 protein in ESCC tumorigenesis. Previously, a few reports described the expression of TIA1 in human carcinomas. The overexpression of TIA1 transcript has been reported in hepatocellular carcinoma tissues compared with paired adjacent non-tumor tissues using multiple samples [13]. In contrast, Izquierdo and his colleague showed the downregulation of the TIA1 protein in a subset of epithelial tumors, relative to normal tissues [14, 28]. Using a larger set of human ESCC cases, we demonstrated that TIA1 is overexpressed and ectopically localized to the cytoplasm, and that cytoplasmic TIA1 immunoreactivity is an independent prognosticator of overall survival. In addition, we demonstrated the oncogenic activity of TIA1, particularly TIA1a, and its molecular mechanisms, at least in part, using a series of experiments in ESCC cells. Our results suggest that TIA1 works as an ESCC oncogene and is likely to be a marker of malignant potential as well as a possible therapeutic target for this tumor type.

Most RBPs, including TIA1, Hu-antigen R (HuR) and AUF1, are expressed predominantly in the nucleus and shuttle between the nucleus and cytoplasm. Their cytoplasmic abundance is substantively linked to their effects on target mRNAs, and dysfunction in their subcellular distribution may have pathological consequences [29]. Indeed, cytoplasmic HuR expression has been shown to be associated with malignant clinicopathological features and poor prognosis in ESCC, indicating that ectopically localized HuR is involved in cancer-promoting RNA metabolism and contributes to the progression of ESCC [30]. In contrast, the cytoplasmic localization of TIA1 protein in epithelial tumor cells has not been well analyzed, although the nucleo-cytoplasmic shuttling of TIA1 under stress conditions has been described in some cells [31]. Because TIA1 overexpression and cytoplasmic localization are commonly observed in various squamous cell carcinomas, cancer-promoting effects of TIA1 through cytoplasmic localization might be cell lineage-specific.

Until now, each TIA1 isoform was considered to have equivalent RNA binding properties and subcellular distribution because these isoforms share most known motifs. Thus, most previous studies used the shorter TIA1b isoform to analyze TIA1 function by exogenously introducing recombinant constructs [11, 28]. In this study, we found that TIA1a and TIA1b exert distinct effects on ESCC cell proliferation: TIA1a promotes anchorage-dependent and anchorage-independent cell proliferation, whereas TIA1b tends to inhibit cell proliferation and/or induces cell death. Therefore, the oncogenic activities of TIA1 we found are not inconsistent with previous reports demonstrating that TIA1 works as a tumor suppressor [12, 14, 28]. Because a larger fraction of TIA1a localizes to the cytoplasm, compared with TIA1b, and because exogenously introduced each isoform shows different mRNA binding affinities, TIA1a may function as an oncogene in ESCC through localizing to the cytoplasm and contributing to specific aspects of RNA metabolism. Although how transcription of those isoforms were regulated differentially in normal and tumor cells and how the 11 amino acid residues contribute to them remains unclear, the functional modification of the TIA1a isoform by focusing on the 11 amino acid residues is a potential therapeutic target for ESCC. This hypothesis is supported, in part, by recent reports showing that the expression pattern of each TIA1 isoform is defined by tissue type and that TIA1b has greater splicing stimulatory activity than TIA1a [11]. Isoform-specific subcellular localization and influences on the mRNA binding affinities of AUF1, which has four isoforms, have also been reported [32–35].

RBPs coordinate a set of multiple functionally related genes by orchestrating their splicing, export, stability, localization and translation in cellular processes [36]. In ESCC cells, over-representation of specific pathways in the TIA1-associated gene set revealed that TIA1 coordinates the expression of a gene cluster encoding cell cycle regulators. This *in silico* prediction corresponded with the results of our *in vitro* functional experiments and with the clinicopathological significance of TIA1 immunoreactivity in ESCC cases, supporting our hypothesis that TIA1 is a possible therapeutic target for simultaneously and efficiently manipulating various target genes/pathways contributing to esophageal carcinogenesis.

Most of the specific *cis* elements that affect mRNA decay and/or translation of a subset of transcripts are found in the 5′ and 3′ UTRs, and the most well-known of these motifs are the adenine-uridine-rich element sequences (AREs) [37–39]. TIA1 was reported to be a negative posttranscriptional modulator through its bindings with AREs by stimulating assembly of the translational silencer complex that was routed to discrete cytoplasmic SGs [40]. Because some RBPs can act as both positive and negative posttranscriptional regulators [41–44], the finding that the expression levels of SKP2 and CCNA2 proteins increased through bindings between TIA1 protein and their transcripts was not surprising. Our results suggested that TIA1 binds to the 3′ UTR of *SKP2* mRNA, resulting in the attenuation of mRNA decay, while TIA1 binds to the 5′ UTR and/or ORF of *CCNA2* mRNA, resulting in the increased translation initiation without acquisition of mRNA. Because previous studies predicted the presence of TIA1 binding motifs in the 3′ UTR of *SKP2* and 5′ UTR of *CCNA2* mRNAs [18], TIA1 might be a suppressor of mRNA decay and translational inducer of *SKP2* and *CCNA2*, respectively. Further elucidation of the precise mechanisms by which TIA1 increases/decreases the expression of a set of genes will reveal novel physiological and pathophysiological functions of TIA1.

## MATERIALS AND METHODS

### Cell lines and primary tissue samples

In total, 45 ESCC cell lines were used, 34 of which belonged to the KYSE series established from surgically resected tumors [45] obtained from Dr. Yutaka Shimada or provided by the Japanese Collection of Research Bioresources (JCRB, Ibaraki, Japan). Ten were from the TE series provided by the Cell Bank, RIKEN BioResource Center (Tsukuba, Japan), and the T. T cell line was provided by JCRB.

ESCC tumor samples were obtained from 143 patients with histologically proven primary ESCC who underwent esophagectomy (potentially curative R0 resection) at the Kyoto Prefectural University of Medicine Hospital (Kyoto, Japan) between 1998 and 2011. The samples were fixed with formalin for 24 h and then embedded in paraffin. Among the patients, 65 received preoperative neoadjuvant therapy with FP (Table 1, Supplementary Table S1), and 39 received postoperative adjuvant chemotherapy with low-dose FP [5-fluorouracil (250–500 mg/body/day) plus cisplatin (10 mg/body/day)] and oral fluoropyrimidine [5-fluorouracil (150–200 mg/body/day) or UFT (300–400 mg/body/day)] for three years [46]. None had synchronous or metachronous multiple cancers in other organs. Relevant clinical and survival data were available for all patients. In this series, all M_1_ tumors had distant lymph node metastases but no organ metastasis. Disease stage was defined in accordance with the International Union Against Cancer tumor-lymph node-metastases (TNM) classification [47]. The median follow-up period for the surviving patients was 40.5 months (range, 0.16 to 156.9 months). Additional tissue samples, including normal and precancerous regions of the esophagus and non-esophageal SCC, were also obtained from patients treated at Tokushima University. Formal written consent was obtained from all patients after approval of all aspects of these studies by the local ethics committee (Kyoto Prefectural University of Medicine and Tokushima University).

### Antibodies

The antibodies used in this study are listed in Supplementary Table S6.

### Immunohistochemical staining and scoring

Paraffin-embedded sections (4-μm thick) were subjected to IHC staining for each protein using the avidin–biotin-peroxidase method. Briefly, antigen retrieval was performed by heating dewaxed and dehydrated sections in Dako Real Target Retrieval Solution (DAKO, Glostrup, Denmark) at 98°C for 30 min. Then, the sections were treated with 0.3% H_2_O_2_ for 30 min to quench the endogenous peroxidase and treated with protein blocker. Next, the sections were incubated with primary antibodies at 4°C overnight. After incubation with secondary antibodies, an R.T.U. VECTASTAIN Universal Quick Kit (Vector Laboratories; Burlingame, CA, USA) and a Universal LSAB™+ Kit/HRP (DAKO) were used for colour development with diaminobenzidine tetrahydrochloride. The sections were counterstained with Mayer's hematoxylin, dehydrated in ascending grades of ethanol and mounted.

Tumor tissues were compared with paired non-tumor tissues. The percentage of the total cell population expressing the target protein and the overall staining intensity in tumor cells were evaluated for each case at 200× magnification. Expression of a target protein was considered positive when over 10% of the tumor cells showed strong or diffuse staining. The nuclear TIA1 staining intensity was considered positive (+) when the cell showed staining that was stronger than that seen in non-tumor esophageal epithelial cells in the parabasal layer, whereas the intensity of cytoplasmic TIA1 staining was considered positive (+) when the cells more or less showed staining. All stained slides were blindly and independently evaluated by two investigators without knowledge of the clinicopathological data, and any discordant result was settled by using a conference microscope.

### Quantitative real-time PCR

One microgram of total RNA was reverse-transcribed using the PrimeScript RT reagent kit (TaKaRa, Otsu, Japan). Transcript levels were quantified using the specific primer sets (Supplementary Table S7) and SYBR Green Master Mix (Applied Biosystems, Waltham, MA, USA), as described elsewhere [48]. *GAPDH* mRNA and *18S* rRNA levels were also measured and used as internal controls for normalization.

### Western blot analysis

Whole-cell lysate preparation and western blot analysis were performed as previously described [49]. Subcellular components were isolated using a NE-PER Nuclear Protein Extraction Kit (Thermo Fisher Scientific, Waltham, MA, USA) according to the manufacturer's instructions.

### Fluorescent immunocytochemical staining

Cells were cultured on chamber slides, fixed in 4% paraformaldehyde for 30 min at room temperature, permeabilised with 0.1% Triton X-100 in phosphate-buffered saline (PBS) for 1 min and treated with blocking reagent (1% BSA) for 30 min. After the cells were incubated with primary antibodies for 1 h at room temperature, the bound antibody was visualized using fluorescence-labelled secondary antibodies. After mounting using ProLong Gold Antifade Reagent with 4′, 6-diamidino-2-phenylindole (DAPI), the cells were observed under a fluorescence microscope (LSM510; Carl Zeiss, Oberkochen, Germany).

### Plasmid construction

The full coding sequences of human *TIA1 variant 1* (NM_022037), *TIA1 variant 2* (NM_022173), *SKP2* (NM_005983), and *CCNA2* (NM_001237) were amplified by PCR (Supplementary Table S7) using cDNA prepared from KYSE140 cells. These coding sequences were cloned into the mammalian expression vector pCMV-3Tag1A (Stratagene, La Jolla, CA, USA) to append the FLAG epitope to their NH_2_ termini. FLAG-tagged TIA1 variants were amplified by PCR (Supplementary Table S7) and cloned into the retroviral vector pMXs-Neo (Cell Biolabs, San Diego, CA, USA). The 3′ UTR of *SKP2* were amplified by PCR (Supplementary Table S7) using cDNA prepared from KYSE140 cells, and ligated into the pmirGLO vector (Promega, Madison, WI, USA) to generate the plasmid expressing a chimeric RNA that contains the *Luc2* CDS linked to the *SKP2* 3′ UTR (pmirGLO-SKP2).

### Stable transfection experiments

To establish ESCC cell lines stably overexpressing each TIA1 isoform, cells were infected with each TIA1 variant-expressing retroviruses and selected by treatment with 0.5 mg/mL G418 for four weeks. Control cells were obtained using retroviruses obtained from the empty pMXs-Neo vector that were packaged in PLAT-A cells. Clones were subsequently isolated, subcultured and tested for TIA1 overexpression by western blot and FIC.

### Transient transfection experiments

Various siRNAs targeting mRNAs of TIA1 (#1-3), each TIA1 isoform, SKP2, and CCNA2, as well as control siRNAs (Supplementary Table S8), were transfected separately into ESCC cells at a final concentration of 10 nM using Lipofectamine RNAiMax reagent (Invitrogen, Carlsbad, CA, USA). A control plasmid (pCMV-3Tag1A), a pCMV-3Tag1A-containing FLAG-tagged TIA1 variant 1 (pTIA1[v1]-FLAG), a pCMV-3Tag1A-containing FLAG-tagged TIA1 variant 2 (pTIA1[v2]-FLAG), and pmirGLO-containing *SKP2* 3′ UTR (pmirGLO-SKP2) were transfected separately into ESCC cell lines or HEK293 cells using Lipofectamine-2000 reagent (Invitrogen).

### Cell proliferation and cell cycle analyses

Cell growth was assessed at the indicated time after cell seeding (5 × 10^3^ cells/96-well plate) using a water-soluble tetrazolium (WST) salt assay (Cell Counting Kit-8; Dojindo, Mashikimachi, Japan) according to the manufacturer's instructions. The results are expressed as the mean absolute absorbance at the indicated time divided by the mean absolute absorbance of each sample cultured for 24 h after seeding.

For colony formation assays, cells infected with either mock-, pTIA1[v1]-FLAG- or pTIA1[v2]-FLAG-expressing retroviruses (1,000 cells/well) were plated in six-well plates and treated with 0.5 mg/mL G418 for two weeks. The colonies in each well were stained with crystal violet, and the areas of colonies formed were determined using ImageJ software (http://imagej.nih.gov/ij/).

For spheroid formation assays, 100 μL/well of cell suspensions at optimized densities (10,000 cells/mL) were dispensed into PrimeSurface 96-well round-bottomed plates (Sumitomo Bakelite, Tokyo, Japan). Plates were centrifuged for 5 min at 1,000 rpm and incubated at 37°C in an atmosphere of 5% CO_2_. The areas of the spheroids formed were determined using ImageJ software.

Cell cycles were evaluated by FACS as described elsewhere [50]. Cell counting was performed using a FACSVerse flow cytometer (Becton Dickinson, Mountain View, CA, USA).

### Ribonucleoprotein immunoprecipitation assay

Immunoprecipitation of ribonucleoprotein complexes was performed using the RiboCluster Profiler RIP-Assay kit (Medical & Biological Laboratories, Nagoya, Japan) according to the manufacturer's protocol. Briefly, cytoplasmic lysates (500 μg protein) prepared from ESCC cells were incubated for 1 h at 4°C with 80 μL of a 50% (v/v) suspension of protein A agarose beads precoated with 15 μg of rabbit IgG_1_ (Medical & Biological Laboratories) or rabbit anti-TIA1 antibody (Supplementary Table S6). After the quality and quantity of RNAs in the input lysates and the IP materials were assessed using an Agilent 2100 Bioanalyzer (Agilent Technologies, Santa Clara, CA, USA), total transcripts and immunoprecipitated transcripts were analyzed using microarray and/or RNA sequencing (RNA-seq).

Microarray data were obtained using a whole human genome microarray (8 × 60 k, Agilent Technologies), as described previously [50], and analyzed using GeneSpring 13.0 software (Agilent Technologies). All microarray data are available at the Gene Expression Omnibus (GSE71342).

To prepare the template for RNA-Seq, first- and second-strand cDNA synthesis was performed using 100 ng of RNA. This was followed by single-primer isothermal amplification using an NEBNext Ultra RNA Library Prep Kit for Illumina (New England Biolabs, Ipswich, MA, USA) according to the manufacturer's protocol. These steps amplified polyA- and non-polyA-tailed RNAs and removed ribosomal RNA. The cDNA library was sequenced using an Illumina MiSeq instrument (Illumina). The quality of the bases was checked using the FASTQC program. Called bases were aligned to the human hg19 genome using the Tophat program, the Bowtie algorithm and Ensembl hg19 (v62) as gene model annotations, followed by genomic mapping. The aligned reads were assembled into transcripts (both known and novel) using the Cufflinks program with Ensembl hg19 (v62) transcripts as a guide. FPKM (fragments per kilobase of exon model per million mapped reads) values were calculated after fragment bias correction and normalization to total hits. The data were visualized in the UCSC genome browser. All RNA-seq data are available at the DNA Data Bank of Japan (PRJDB4086).

After mRNA expression was normalized to constitutive mRNA expression levels determined by microarray, the association of mRNAs with TIA1 was assessed by estimating the enrichment of mRNAs in TIA1 IP samples, compared with those of *GAPDH* mRNA, an abundant mRNA that is not a target of TIA1.

The enrichment analyses of the set of TIA1 target genes and functional pathways related to this gene set were performed using GO enrichment analysis and KEGG pathway analysis, respectively. Over-representation of specific GO and KEGG pathways in a gene set was statistically analyzed by DAVID Bioinformatics Resources 6.7 software (http://david.abcc.ncifcrf.gov/home.jsp) [51, 52]. The enrichment *P*-value calculation, i.e. number of genes in the list that hit a given biology class as compared to pure random chance, was performed with Benjamini and Hochberg multiple testing correction.

### Biotin pulldown analysis

cDNA corresponding to each gene fragment was used as a template for the *in vitro* synthesis of biotinylated transcripts. The T7 RNA polymerase promoter sequence was added to the 5′ ends of all of the fragments using PCR (Supplementary Table S7). The biotinylated *GAPDH* 3′ UTR was prepared as previously described [49]. Biotinylated RNAs were synthesized using the MaxiScript T7 kit (Ambion, Austin, TX, USA). Whole-cell lysates (40 μg for each sample) were incubated with one of the purified biotinylated fragments (4 μg) for 1 h at room temperature. Complexes were isolated with paramagnetic streptavidin-conjugated beads (Dynabeads M280 Streptavidin; Invitrogen), and bound proteins in the pulldown materials were assayed by western blot analysis using anti-TIA1 antibody as described above.

### mRNA decay assay

To measure relative mRNA stabilities in cells with normal TIA1 or reduced TIA1 levels, cultures were treated with 2 μg/mL transcriptional inhibitor, actinomycin D (Merck Millipore, Darmstadt, Germany), for the indicated times. At subsequent times, mRNA levels were measured by qPCR and normalized to *18S* rRNA levels. The data are expressed as percentages of each mRNA level before exposure to actinomycin D (time zero).

### Protein decay assay

To measure relative protein stabilities in cells with normal TIA1 or reduced TIA1 levels, cultures were treated with 0.1 mg/mL translational inhibitor, cycloheximide (Sigma-Aldrich, St Louis, MO, USA), for the indicated times. At subsequent times, protein levels were assayed by western blot analysis.

### Sucrose gradient polyribosome fractionation

KYSE180 cells at < 80% confluence were incubated for 5 min in 0.1 mg/mL cycloheximide and then lifted in 1mL PEB lysis buffer (0.3 M NaCl, 15 mM MgCl_2_, 15 mM Tris-HCl, pH 7.6, 1% Triton X-100, and 0.1 mg/mL cycloheximide) by scraping and lysed on ice for 10 min. The lysates were centrifuged at 14,000 rpm at 4°C for 10 min. The supernatant (1 mg protein) was layered onto liner 10–50% sucrose gradient. After centrifuging at 4°C for 90 min at 39,000 rpm, 500 μL fractions were collected. RNA for qPCR in each fraction was extracted with TRIzol-LS Reagent (Thermo Fisher Scientific) according to the manufacturer's instructions.

### Luciferase reporter assay

PmirGLO-SKP2 or control plasmid (pmirGLO) was transfected into ESCC cells in which endogenous TIA1 was silenced by siRNA. After 48 h, cultures were treated with 2 μg/mL actinomycin D (Merck Millipore) for the indicated times. At subsequent times, *Luc2* mRNA levels were measured by qPCR and normalized to *18S* rRNA levels. The data are expressed as percentages of each mRNA level before exposure to actinomycin D (time zero).

### Statistical analysis

The clinicopathological variables pertaining to the corresponding patients were analyzed by using the *χ* ^2^ test or Fisher's exact test. For survival analysis, Kaplan–Meier survival curves were constructed for groups based on univariate predictors, and differences between the groups were tested using the log-rank test. Univariate and multivariate survival analyses were performed using the likelihood ratio test of the stratified Cox proportional hazards model. Differences between subgroups were evaluated using Student's *t*-test. Differences were assessed with a two-sided test and considered significant at the *P* < 0.05 level.

## SUPPLEMENTARY MATERIALS FIGURES AND TABLES


