# Supplementary material for: Tumor-promoting function and prognostic significance of the RNA-binding protein T-cell intracellular antigen-1 in esophageal squamous cell carcinoma
Source: Oncotarget. 2016 Mar 6;7(13):17111–28. doi: 10.18632/oncotarget.7937 (PMC4941375; doi:10.18632/oncotarget.7937)
Supplement: Supplementary file 1 [file oncotarget-07-17111-s001.pdf]

Tumor-promoting function and prognostic significance of the RNA-binding protein T-cell intracellular antigen-1 in esophageal squamous cell carcinoma

Supplementary Materials

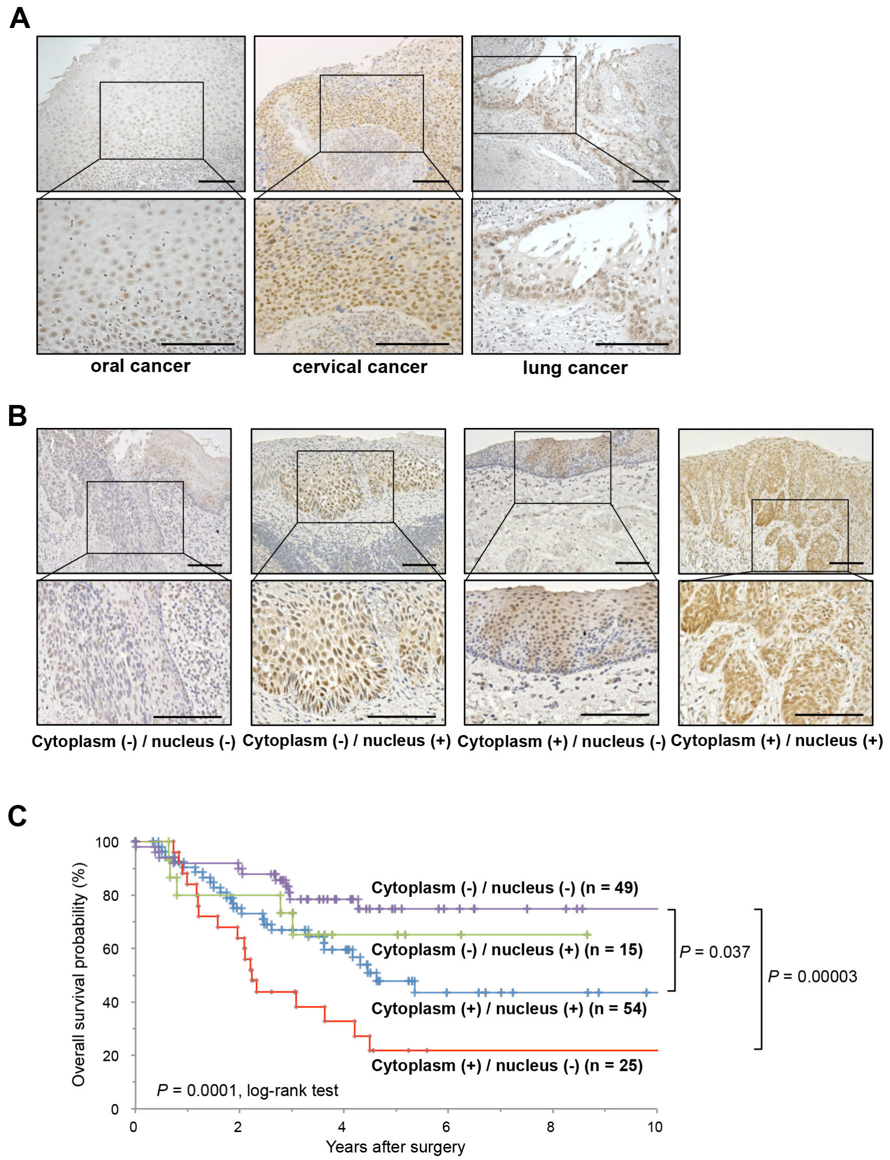

Supplementary Figure S1: Subcellular distribution of TIA1 in cancer tissues and its clinicopathological significance in ESCC cases. (**A**) IHC detection of TIA1 in primary squamous cell carcinoma. Sections of oral, cervical, and lung squamous cell carcinomas were subjected to IHC analysis with goat anti-TIA1 antibody. As observed in ESCC, TIA1 immunoreactivity was observed in both the cytoplasm and the nuclei of tumor cells. Scale bars: 40 µm. (**B**) Representative IHC staining patterns of TIA1 in primary ESCC tissues. Patterns were classified into four groups using the scoring system described in the Materials and Methods section: negative in both the cytoplasm and the nucleus, positive primarily in the nucleus, positive primarily in the cytoplasm, and positive in both the cytoplasm and the nucleus. Scale bars: 40 µm. (**C**) Kaplan–Meier curves for overall survival rates of 143 ESCC patients according to both the cytoplasmic and nuclear expression levels of TIA1 protein. The log-rank test was used for statistical analysis. Differences resulting in values of $P < 0.05$ are considered statistically significant. Post-hoc test using Holm's method demonstrated statistically significant difference was observed between the cytoplasm (−)/nucleus (−) group and the cytoplasm (+)/nucleus (+) group or the cytoplasm (+)/nucleus (−) group.

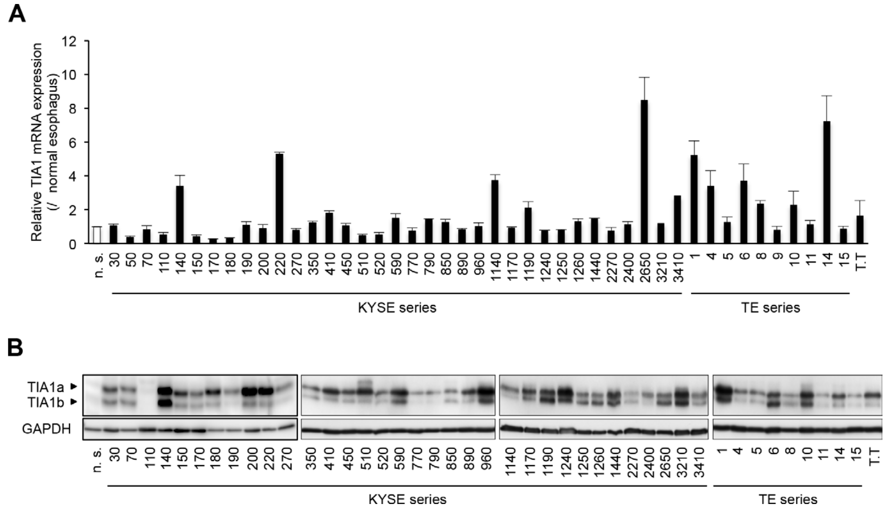

Supplementary Figure S2: TIA1 mRNA and protein expressions in ESCC cell lines. (**A**) Amounts of total *TIA1* mRNA in a set of ESCC cell lines and the normal esophageal tissue were measured by qPCR using *GAPDH* mRNA as an endogenous control. The values are expressed as fold changes (mean ± SD, *n* = 3) compared with the control value from the normal esophageal tissue (n.s.). (**B**) TIA1 protein levels in a set of ESCC cell lines and the normal esophageal tissue were measured by western blot analysis using GAPDH as a loading control. ESCC cells constitutively express the TIA1a isoform (upper band) and an equal or smaller amount of the TIA1b isoform (lower band).

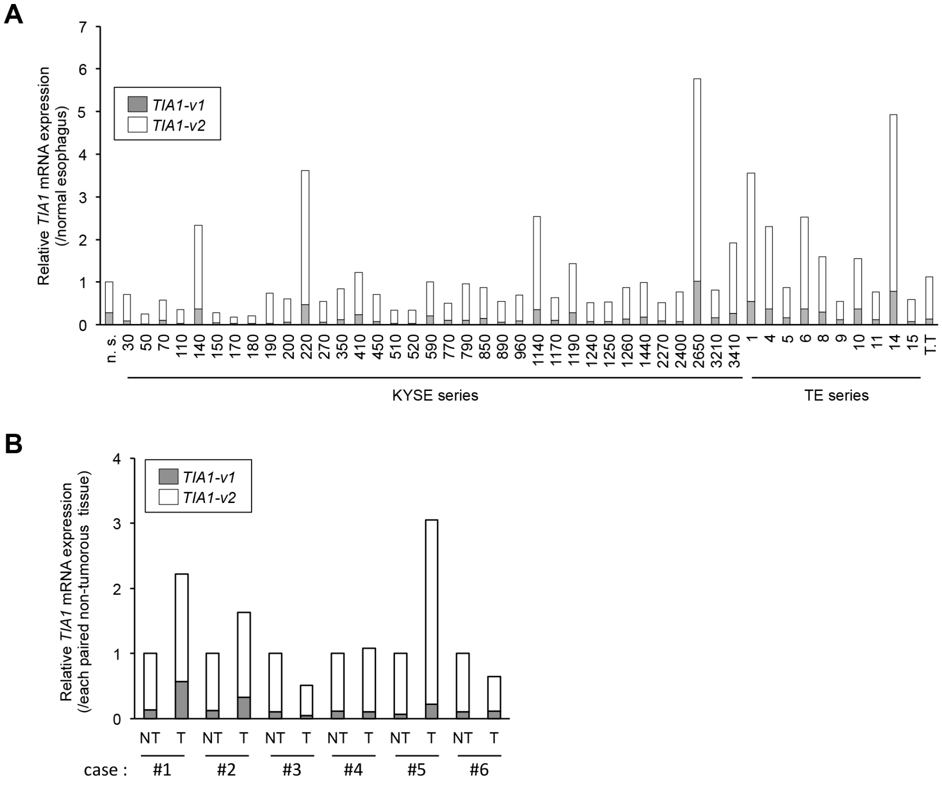

Supplementary Figure S3: Expression patterns of TIA1 transcript variants encoding different isoforms in ESCC. (**A**) Amounts of *TIA1-v1* and *-v2* mRNAs were separately measured by qPCR using *GAPDH* mRNA as an endogenous control. The values are expressed as fold changes (mean ± SD, *n* = 3) compared with the respective control values in normal esophagus (n.s.). (**B**) Total RNA was prepared from six frozen ESCC tumor tissues and paired non-tumor tissues, and the amounts of *TIA1-v1* and *-v2* mRNAs were separately measured by qPCR using *GAPDH* mRNA as an endogenous control. The values are expressed as fold changes compared with the respective controls in corresponding non-tumor esophageal tissues.

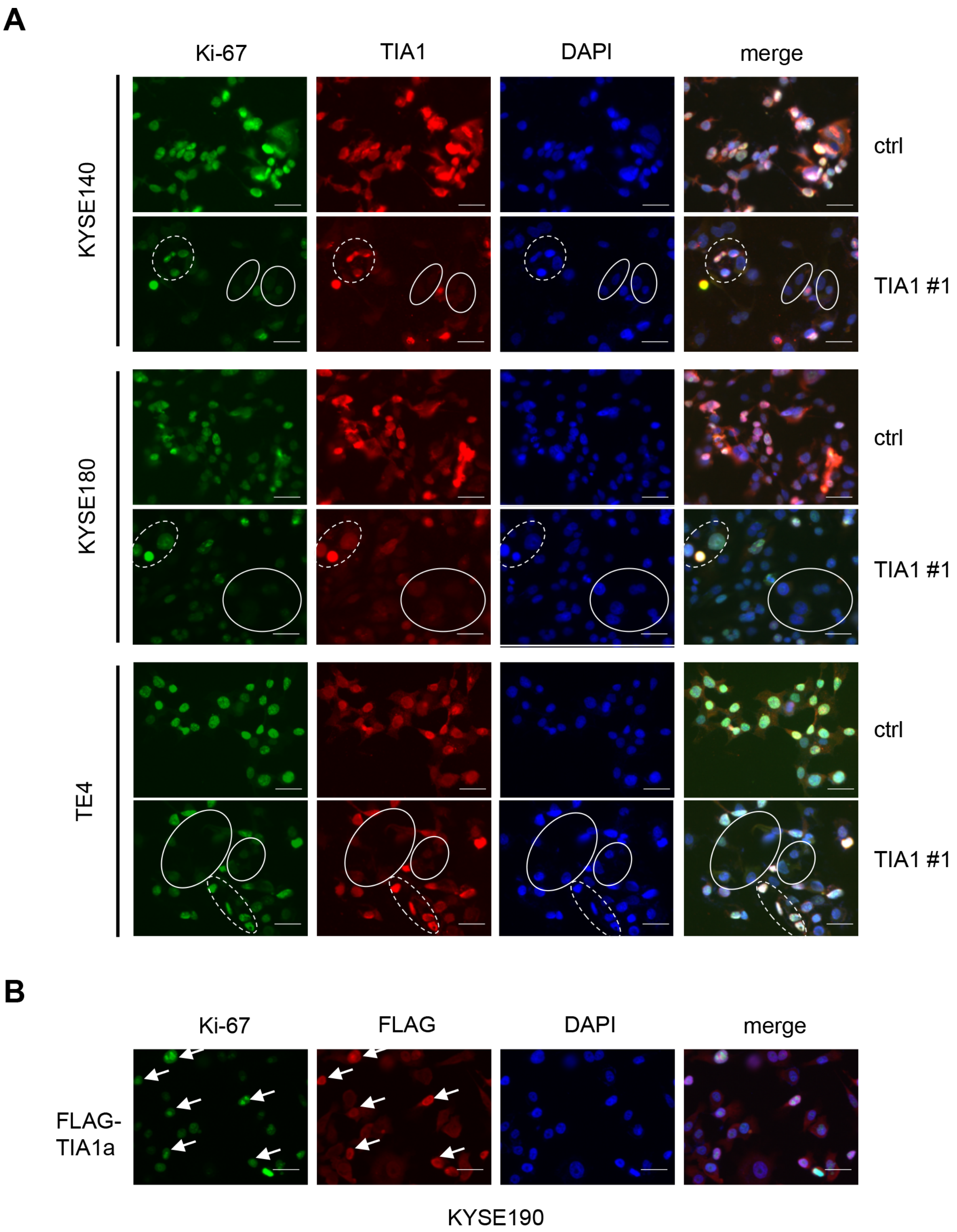

Supplementary Figure S4: Expression status of TIA1 and Ki-67 assessed using FIC in ESCC cells. (**A**) KYSE140, KYSE180 and TE4 cells were transfected with 10 nM TIA1-specific (TIA1 #1) or control (ctrl) siRNA for 48 h and subjected to FIC with anti-Ki-67 (green) and anti-TIA1 (red) antibodies. Nuclei were counterstained with DAPI (blue). Ki-67 immunoreactivity correlated with TIA1 expression. Cells that retained the expression of endogenous TIA1 even after TIA1-specific siRNA treatment showed higher Ki-67 immunoreactivity (dashed-line circle) than cells that effectively lost TIA1 protein expression (solid-line circle). Scale bars: 80 µm. (**B**) KYSE190 cells infected with the FLAG-tagged TIA1a-expressing retrovirus for 48 h were examined by FIC with anti-Ki-67 (green) and anti-FLAG (red) antibodies. Nuclei were counterstained with DAPI (blue). Scale bars: 80 µm. TIA1-expressing cells (arrows) showed higher Ki-67 immunoreactivity than did non-expressing cells.

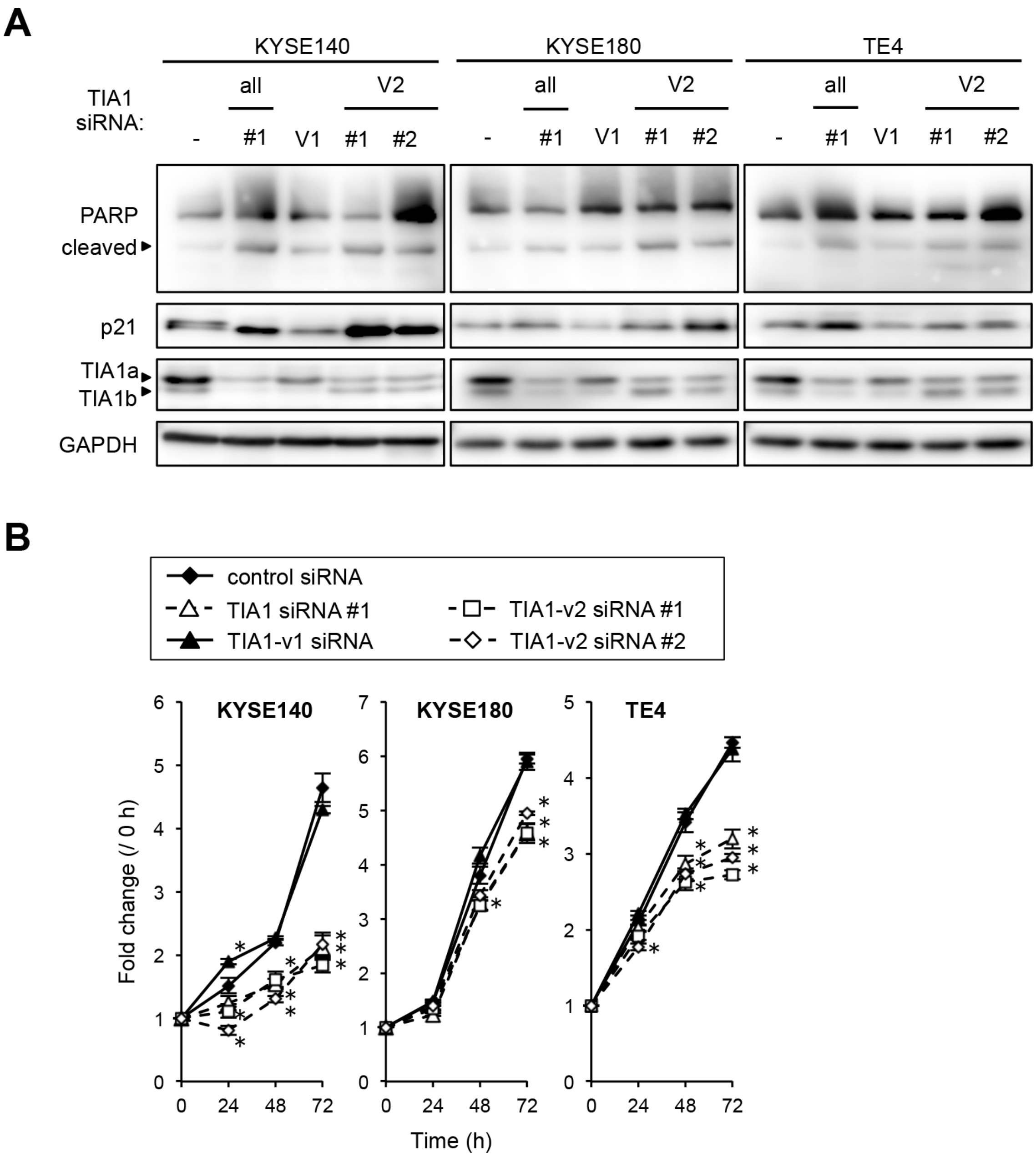

Supplementary Figure S5: Effects of TIA1 isoform-specific siRNA treatment on proliferation of ESCC cells. (**A**) KYSE140, KYSE180, or TE4 were transfected with 10 nM TIA1-specific (TIA1 siRNA #1), each TIA1 isoform-specific or control (−) siRNA, and levels of PARP, $p21^{WAF1/Cip1}$ and TIA1 proteins were measured 48 h after transfection by western blot analysis using GAPDH as a loading control. (**B**) KYSE140, KYSE180, or TE4 cells were transfected with 10 nM TIA1-specific (TIA1 siRNA #1), each TIA1 isoform-specific or control siRNA for 24 h, and cellular proliferation was measured using a WST assay at the indicated times. The values are expressed as fold changes (mean ± SD, $n = 4$) compared with the respective values in control cells (0 h). *significantly different from the control value by Student's t test ($P < 0.05$).

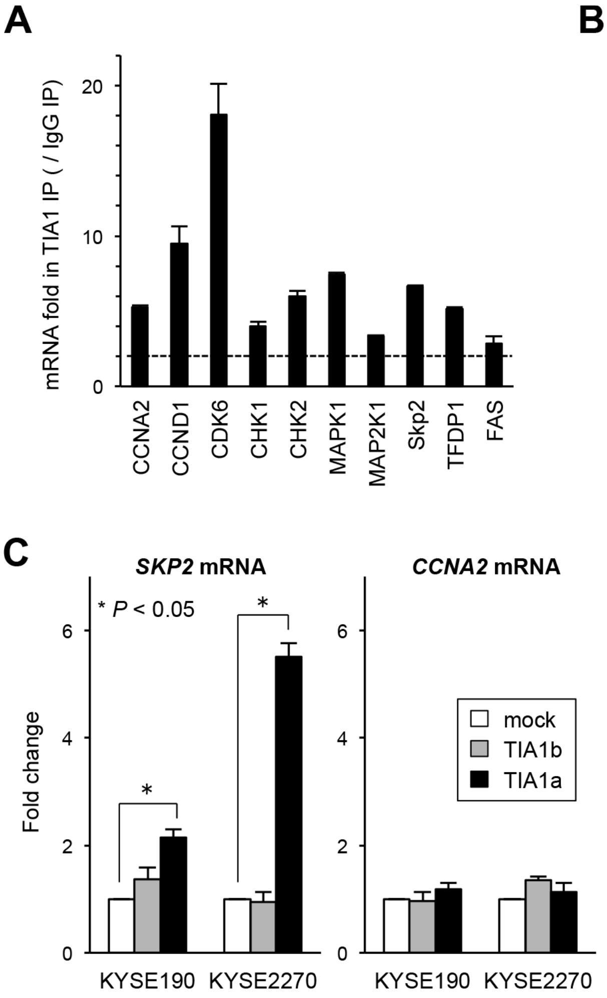

Supplementary Figure S6: Binding of TIA1 with the mRNAs of putative target genes and its effect on protein levels in KYSE140 cells. (**A**) Bindings between TIA1 and its putative target mRNAs, examined in KYSE180 cells and shown in Figure 5A, were also validated in KYSE140 cells. See the figure legend for Figure 5A for details. Representative results from four independent experiments are shown. (**B**) Effects of TIA1 silencing on the levels of putative target mRNAs expression, examined in KYSE180 cells and shown in Figure 5B, were also validated in KYSE140 cells. See the figure legend for Figure 5B for details. The values are expressed as fold changes (mean ± SD, $n = 3$) compared with the respective values in control siRNA-transfected cells. (**C**) Effects of exogenous overexpression of each TIA1 isoform on the expression of putative TIA1-binding mRNAs in ESCC cells. Total RNAs were prepared from KYSE190 or KYSE2270 cells transiently infected with either a mock-, pTIA1[v1]-FLAG- or pTIA1[v2]-FLAG-expressing retrovirus. The levels of *SKP2* and *CCNA2* mRNAs were measured by qPCR using *GAPDH* mRNA as an endogenous control. The values are expressed as fold changes (mean ± SD, $n = 3$) compared with the respective values in control cells (mock). *significantly different from the control value by Student's t test ($P < 0.05$).

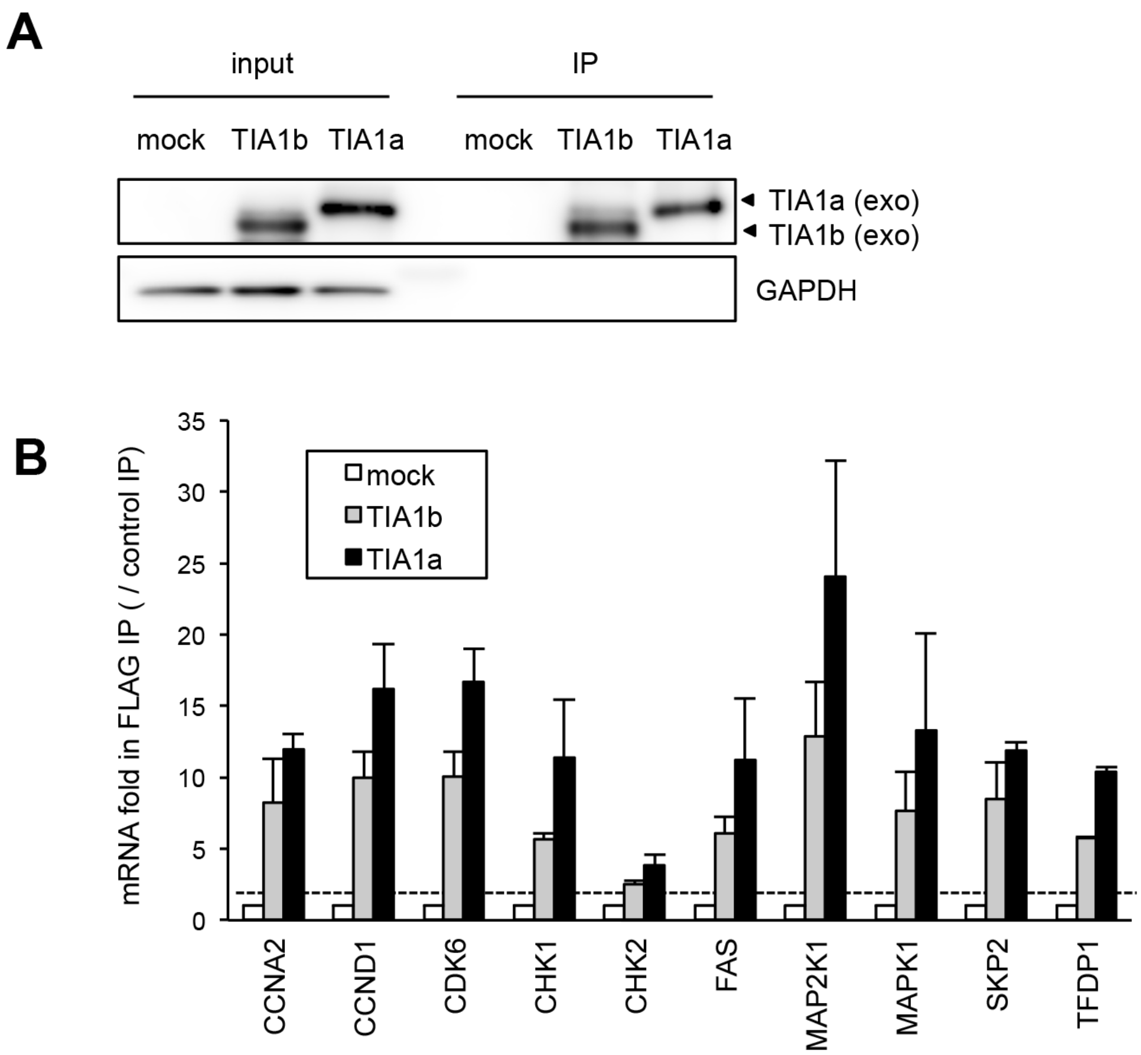

Supplementary Figure S7: Binding of exogenously expressed TIA1 isoforms with their putative target mRNAs in HEK293 cells. (**A**) HEK293 cells were transiently transfected with FLAG-TIA1[v1], FLAG-TIA1[v2] or empty vector for 48 h. Amounts of FLAG-tagged TIA1b and TIA1a proteins in the cytoplasmic lysates (input) and immunoprecipitates using anti-FLAG antibody (IP) were measured by western blot analysis using anti-TIA1 antibody. (**B**) Bindings between TIA1 isoforms and target mRNAs were measured by RIP using anti-FLAG antibody and lysates prepared from HEK293 cells expressing each FLAG-tagged isoform, followed by qPCR amplification. Bindings are represented as relative enrichment with respect to background binding (mock). The data were normalized to the levels of *GAPDH* mRNA, an abundant mRNA that is not a target of TIA1 and that is present as a low-level co-precipitated contaminant in all IP samples. Representative results of four independent experiments are shown.

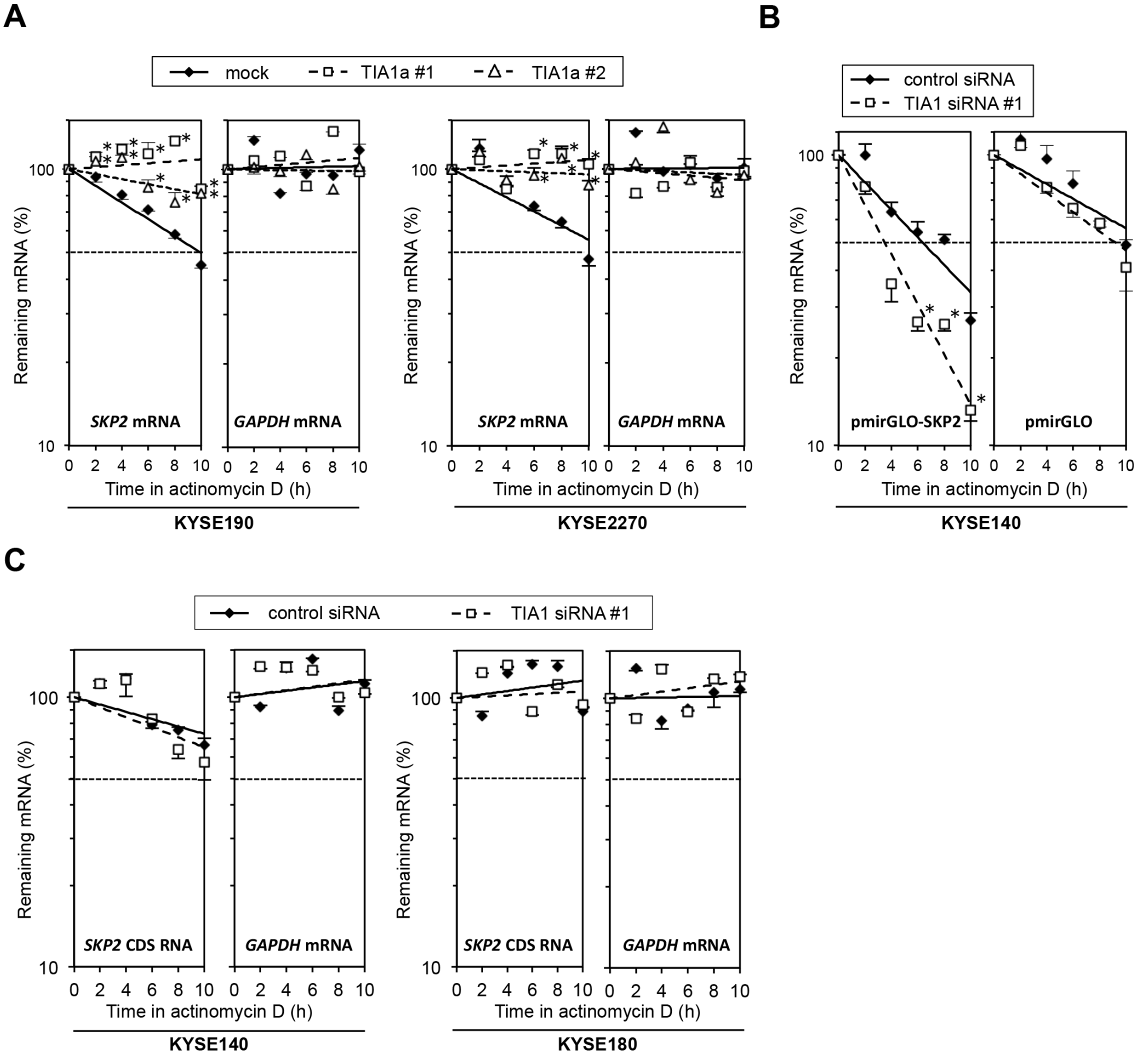

Supplementary Figure S8: Effects of TIA1 on the stability of *SKP2* mRNA in ESCC cells. (**A**) Amounts of *SKP2* and *GAPDH* mRNAs in KYSE190 (left) or KYSE2270 (right) cells stably overexpressing TIA1a or their control mock transfectants treated with actinomycin D (2 µg/mL) were measured by qPCR and normalized to *18S* rRNA levels. Data (mean ± SD, $n = 3$) are expressed as percentages of each mRNA level before exposure to actinomycin D (time 0). *significantly different from the control value by Student's *t* test ($P < 0.05$). (**B**) Effects of TIA1 silencing on stability of the chimeric reporter transcript bearing the *Luc2* CDS and the *SKP2* 3′ UTR, which were examined in KYSE180 cells in Figure 6C, were validated in KYSE140 cells. See Figure 6C for details. Representative results from four independent experiments are shown. (**C**) Effects of TIA1 on the stability of *SKP2* CDS RNA. KYSE140 or KYSE180 cells transiently infected with FLAG-SKP2-expressing retrovirus were treated with 10 nM TIA1 (TIA1 siRNA #1) or control siRNA at the presence of 2 µg/mL actinomycin D, the amounts of Flag-*SKP2* CDS RNA (left) and *GAPDH* (right) mRNAs were measured by qPCR and normalized to *18S* rRNA levels. The data (mean ± SD, $n = 3$) are expressed as percentages of each RNA level before exposure to actinomycin D (time 0).

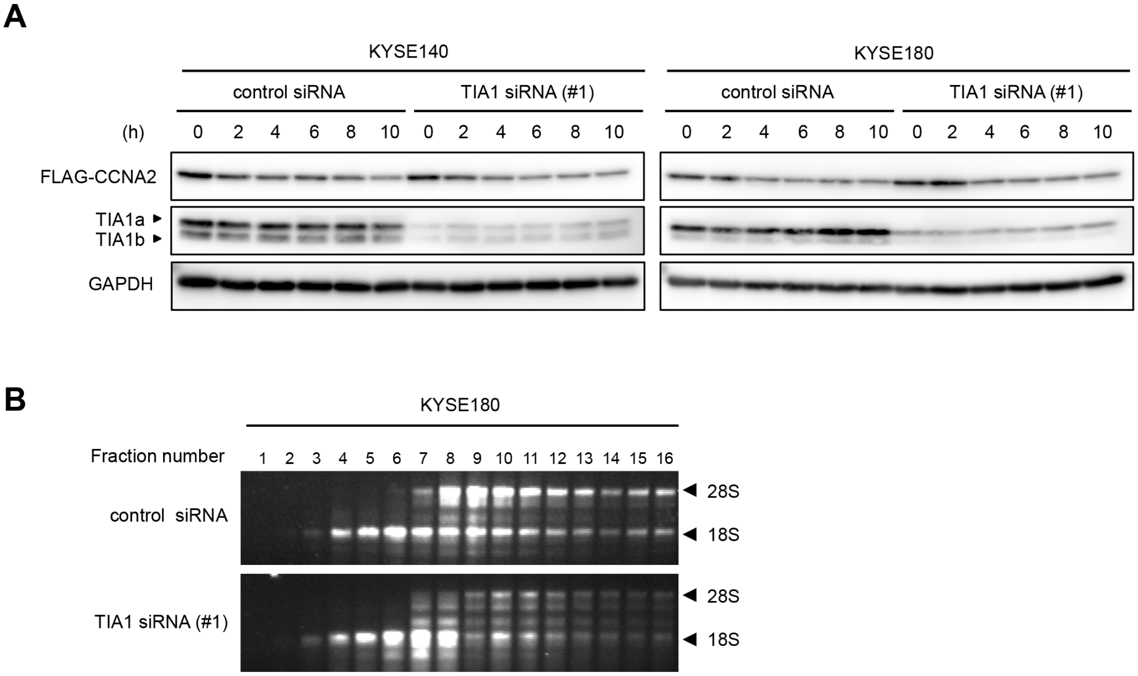

Supplementary Figure S9: Effects of TIA1 on the translational efficiency of CCNA2 mRNA and stability of CCNA2 protein in ESCC cells. (A) The effect of TIA1 on the stability of CCNA2 protein. KYSE140 (left) or KYSE180 (right) cells transiently infected with FLAG-CCNA2-expressiong retrovirus were treated with 10 nM TIA1 (TIA1 siRNA #1) or control siRNA for 48 h, and exposed to 0.1 mg/mL cycloheximide for indicated times. The levels of FLAG-CCNA2 protein were measured by western blot analysis using GAPDH as a loading control. **(B)** Polysomal profiles from cells that either were treated with 10 nM TIA1 (TIA1 siRNA #1) or control siRNA for 48 h. The relative abundance levels of *28S* and *18S* rRNAs were visualized from ethidium bromide-stained agarose gels. See Figure 6D for details.

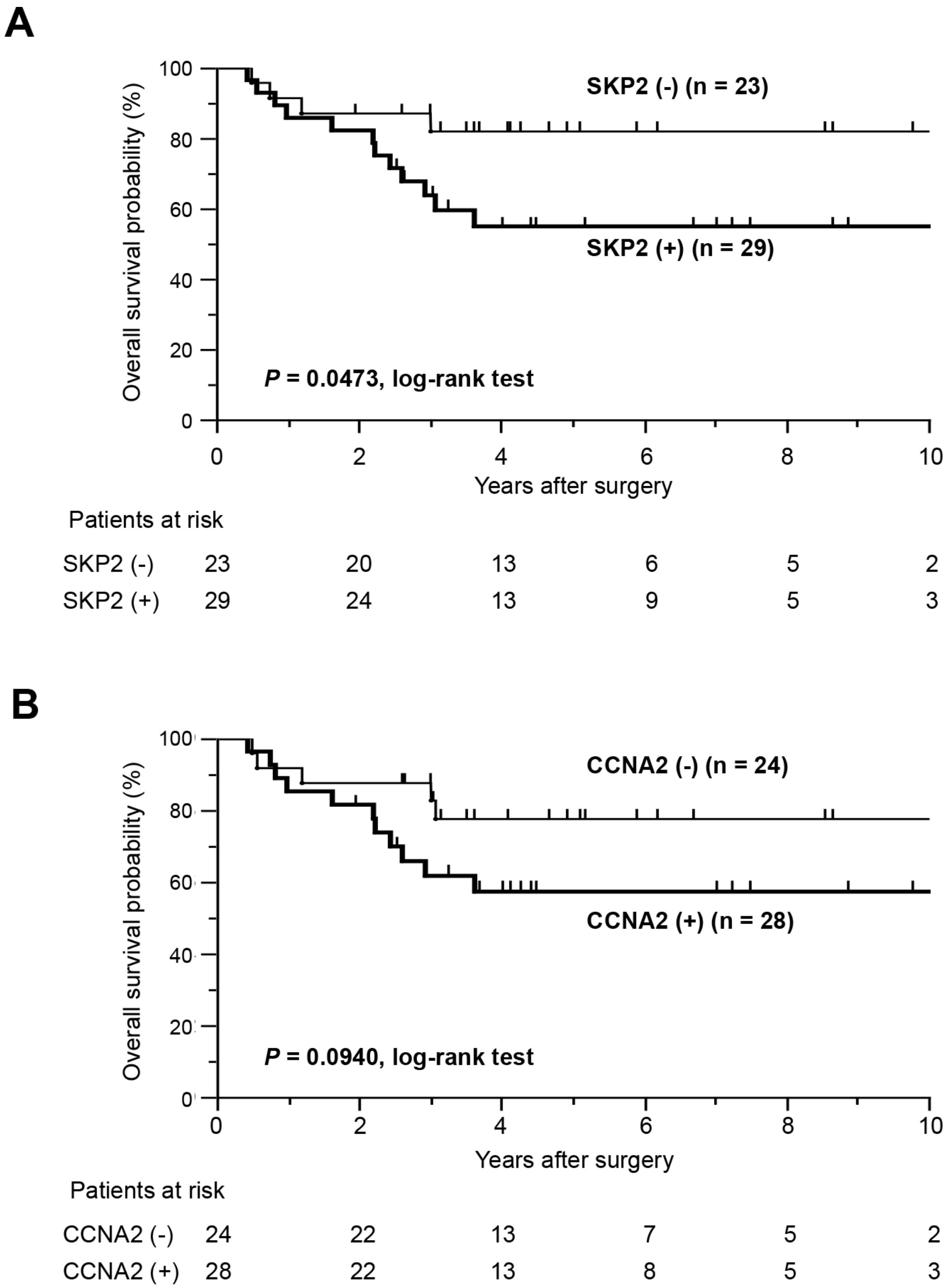

Supplementary Figure S10: Correlation between SKP2 or CCNA2 protein expression status and overall survival in patients with ESCC. Kaplan–Meier curves for the overall survival rates of 52 ESCC patients according to SKP2 (**A**) or CCNA2 (**B**) protein expression. The log-rank test was used for statistical analysis. Differences resulting in $P < 0.05$ are considered statistically significant.

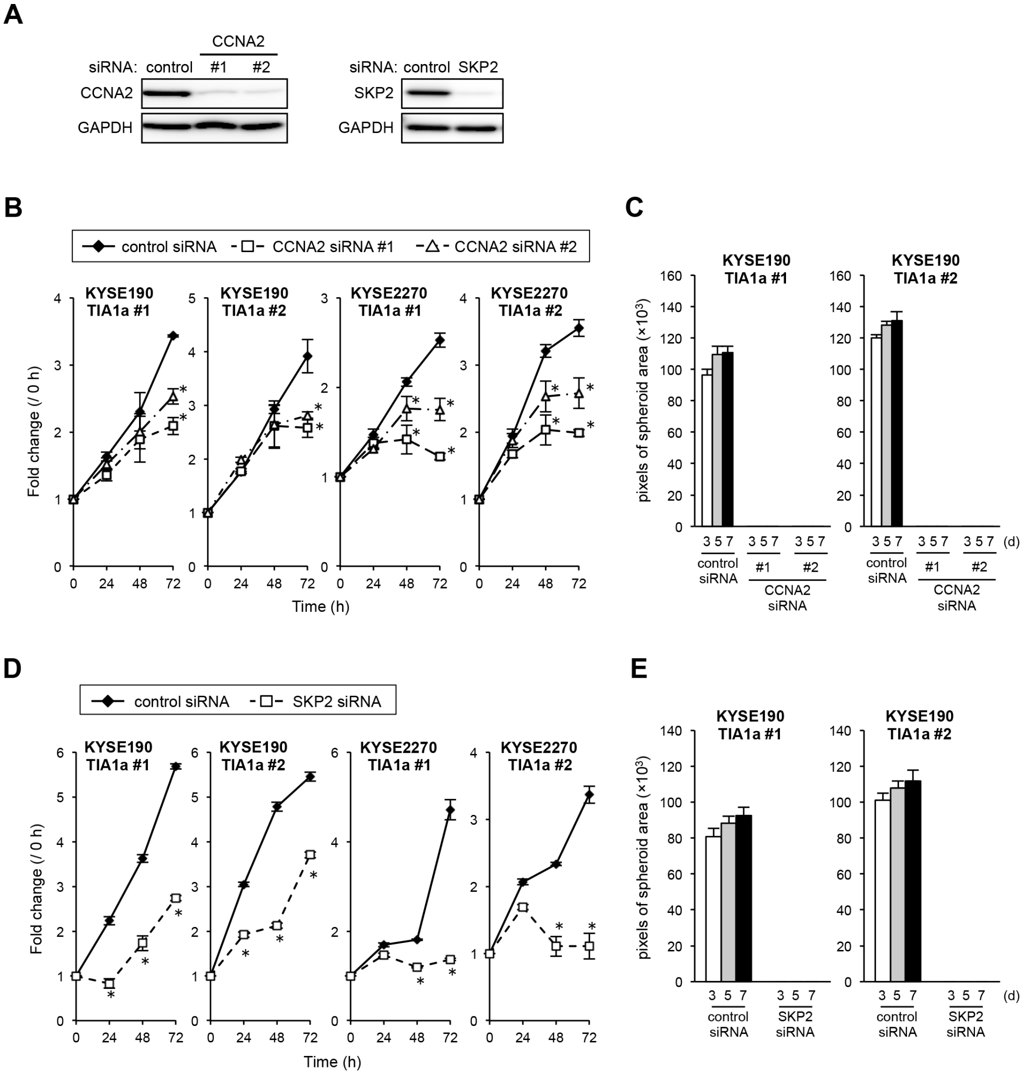

Supplementary Figure S11: Effects of SKP2 or CCNA2 silencing on the TIA1-induced anchorage-dependent and anchorage-independent growth of ESCC cells. (**A**) To assess the effect of each siRNA on target protein expression, the KYSE140 cells were transfected with each 10 nM CCNA2-specific (#1 or #2), SKP2-specific or control siRNA for 48 h, and the expression levels of CCNA2 (left) and SKP2 (right) protein were evaluated by western blot analysis using GAPDH as a loading control. (**B**) The KYSE190 or KYSE2270 cells stably expressing FLAG-tagged TIA1a protein were transfected with 10 nM CCNA2-specific (#1 or #2) or control siRNA for 24 h, and cellular proliferation was measured using a WST assay at the indicated times. The values are expressed as fold changes (mean ± SD, $n = 4$) compared with the respective values in control cells (0 h). *significantly different from the control value by Student's t test ($P < 0.05$). (**C**) TIA1a stable transfectants treated by each siRNA as described above were seeded in ultra-low attachment 96-well round bottom plates and incubated at 37°C for the indicated times (d, days). The areas of spheroids were determined as described in the Materials and Methods section (mean ± SD, $n = 8$). (**D**) The KYSE190 or KYSE2270 cells stably expressing FLAG-tagged TIA1a protein were transfected with 10 nM SKP2-specific or control siRNA for 24 h, and cellular proliferation was measured using a WST assay at the indicated times. (**E**) TIA1a stable transfectants treated by each siRNA as described above were seeded in ultra-low attachment 96-well round bottom plates and incubated at 37°C for the indicated times (d, days). The areas of spheroids were determined as described in the Materials and Methods section (mean ± SD, $n = 8$).

Supplementary Table S1: Association between clinicopathological characteristics and TIA1 expression in patients with neoadjuvant chemotherapy

Clinicopathological factors	*n*	TIA1 immunoreactivity (Cytoplasm)				*P* value[a]	TIA1 immunoreactivity (Nuceus)				*P* value[a]	TIA1 immunoreactivity (Whole)				*P* value[a]
		Positive (%)		Negative (%)			Positive (%)		Negative (%)			Positive (%)		Negative (%)		
Total	65	32	(49.2)	33	(50.8)		32	(49.2)	33	(50.8)		27	(41.5)	38	(58.5)	
Gender																
Male	51	28	(54.9)	23	(45.1)	0.0768	23	(45.1)	28	(54.9)	0.2011	22	(43.1)	29	(56.9)	0.6155
Female	14	4	(28.6)	10	(71.4)		9	(64.3)	5	(35.7)		5	(35.7)	9	(64.3)	
Age																
mean ± SD (yr)	63.9 ± 7.8	64.0	± 8.4	63.8	± 7.22	0.9267	63.6	± 6.8	64.2	± 8.7	0.6174	64.4	± 6.6	63.6	± 8.6	0.8258
Location[b]																
Upper	18	8	(44.4)	10	(55.6)	0.8006	9	(50.0)	11	(61.1)	0.8006	8	(44.4)	10	(55.6)	0.7733
Middle	27	13	(48.1)	14	(51.9)		13	(48.1)	14	(51.9)		12	(44.4)	15	(55.6)	
Lower	20	11	(55.0)	9	(45.0)		10	(50.0)	8	(40.0)		7	(35.0)	13	(65.0)	
Histopathological grading[c]																
Well and moderately differentiated	42	21	(50.0)	21	(50.0)	0.8669	21	(50.0)	21	(50.0)	0.8669	16	(38.1)	26	(61.9)	0.4475
Poorly	23	11	(47.8)	12	(52.2)		11	(47.8)	12	(52.2)		11	(47.8)	12	(52.2)	
Size																
Mean ± SD (mm)	47.1 ± 29.4	45.5	± 28.8	48.6	± 30.3	0.5032	44.6	± 28.9	49.5	± 30.0	0.3936	45.6	± 30.2	48.2	± 29.2	0.4518
Lymphatic invasion (ly)											0.7137					0.5714
Negative	31	14	(45.2)	17	(54.8)	0.5307	16	(51.6)	15	(48.4)		14	(45.2)	17	(54.8)	
Positive	34	18	(52.9)	16	(47.1)		16	(47.1)	18	(52.9)		13	(38.2)	21	(61.8)	
Venous invasion (v)											0.3895					0.6888
Negative	38	17	(44.7)	21	(55.3)	0.3895	17	(44.7)	21	(55.3)		15	(39.5)	23	(60.5)	
Positive	27	15	(55.6)	12	(44.4)		15	(55.6)	12	(44.4)		12	(44.4)	15	(55.6)	
Depth of tumor invasion (pT)						0.3004					0.9388					0.7690
pT1	18	7	(38.9)	11	(61.1)		9	(50.0)	9	(50.0)		8	(44.4)	10	(55.6)	
pT2-4	47	25	(53.2)	22	(46.8)		23	(48.9)	24	(51.1)		19	(40.4)	28	(59.6)	
N stage (pN)																
pN0	14	7	(50.0)	7	(50.0)	0.9482	9	(64.3)	5	(35.7)	0.2011	7	(50.0)	7	(50.0)	0.4705
pN1-3	51	25	(49.0)	26	(51.0)		23	(45.1)	28	(54.9)		20	(39.2)	31	(60.8)	
pStage																
pI	5	3	(60.0)	2	(40.0)	0.6152	2	(40.0)	3	(60.0)	0.6663	2	(40.0)	3	(60.0)	0.9420
pII-VI	60	29	(48.3)	31	(51.7)		30	(50.0)	30	(50.0)		25	(41.7)	35	(58.3)	

Statistically significant value are in boldface type.

[a]*P* value are from χ^2 or Fisher's exact test and were statistically significant at < 0.05.

[b]Upper, cervical + upper thoracic esophagus; Middle, mid-thoracic esophagus; Lower, lower thoracic + abdominal esophagus.

[c]Well, well differentiated SCC; Moderate, moderate differentiated SCC; Poorly, poorly differentiated SCC.

Supplementary Table S2: Association between clinicopathological characteristics and TIA1 expression in patients without neoadjuvant chemotherapy

Clinicopathological factors	*n*	TIA1 immunoreactivity (Cytoplasm)				*P* value[a]	TIA1 immunoreactivity (Nucleus)				*P* value[a]	TIA1 immunoreactivity (Whole)				*P* value[a]
		Positive (%)		Negative (%)			Positive (%)		Negative (%)			Positive (%)		Negative (%)		
Total	78	47	(60.3)	31	(39.7)		37	(47.4)	41	(52.6)		40	(51.3)	38	(48.7)	
Gender																
Male	66	41	(62.1)	25	(37.9)	0.4339	30	(45.5)	36	(54.5)	0.4110	35	(53.0)	31	(47.0)	0.4682
Female	12	6	(50.0)	6	(50.0)		7	(58.3)	5	(41.7)		5	(41.7)	7	(58.3)	
Age																
mean ± SD (yr)	62.6 ± 6.8	62.0	± 6.9	63.6	± 6.7	0.3112	61.4	± 6.4	64.0	±.7.1	0.1560	61.6	± 6.4	63.7	± 7.2	0.3000
Location[b]																
Upper	9	4	(44.4)	5	(55.6)	0.1561	4	(44.4)	5	(55.6)	0.3057	4	(44.4)	5	(55.6)	0.5808
Middle	42	23	(54.8)	19	(45.2)		17	(40.5)	25	(59.5)		20	(47.6)	22	(52.4)	
Lower	27	20	(74.1)	7	(25.9)		16	(59.3)	11	(40.7)		16	(59.3)	11	(40.7)	
Histopathological grading[c]																
Well and moderately differentiated	54	34	(63.0)	20	(37.0)	0.4654	24	(44.4)	30	(55.6)	0.4275	28	(51.9)	26	(48.1)	0.8800
Poorly	24	13	(54.2)	11	(45.8)		13	(54.2)	11	(45.8)		12	(50.0)	12	(50.0)	
Size																
Mean ± SD (mm)	43.7 ± 24.6	47.2	± 24.6	38.4	± 24.2	**0.0295**	47.5	± 26.0	40.3	± 23.1	0.0896	48.4	± 26.3	38.8	± 22.1	**0.0412**
Lymphatic invasion (ly)											0.8023					0.6584
Negative	37	21	(56.8)	16	(43.2)	0.5485	17	(45.9)	20	(54.1)		18	(48.6)	19	(51.4)	
Positive	41	26	(63.4)	15	(36.6)		20	(48.8)	21	(51.2)		22	(53.7)	19	(46.3)	
Venous invasion (v)											0.2793					0.5209
Negative	50	30	(60.0)	20	(40.0)	0.9507	26	(52.0)	24	(48.0)		27	(54.0)	23	(46.0)	
Positive	28	17	(60.7)	11	(39.3)		11	(39.3)	17	(60.7)		13	(46.4)	15	(53.6)	
Depth of tumor invasion (pT)						0.1262					0.4809					0.3700
pT1	37	19	(51.4)	18	(48.6)		16	(43.2)	21	(56.8)		17	(45.9)	20	(54.1)	
pT2-4	41	28	(68.3)	13	(31.7)		21	(51.2)	20	(48.8)		23	(56.1)	18	(43.9)	
N stage (pN)																
pN0	37	19	(51.4)	18	(48.6)	0.1262	14	(37.8)	23	(62.2)	0.1057	15	(40.5)	22	(59.5)	0.0704
pN1-3	41	28	(68.3)	13	(31.7)		23	(56.1)	18	(43.9)		25	(61.0)	16	(39.0)	
pStage																
pI	31	18	(58.1)	13	(41.9)	0.7482	13	(41.9)	18	(58.1)	0.4288	15	(48.4)	16	(51.6)	0.6778
pII–VI	47	29	(61.7)	18	(38.3)		24	(51.1)	23	(48.9)		25	(53.2)	22	(46.8)	

Statistically significant value are in boldface type.

[a]*P* value are from χ^2 or Fisher's exact test and were statistically significant at < 0.05.

[b]Upper, cervical + upper thoracic esophagus; Middle, mid-thoracic esophagus; Lower, lower thoracic + abdominal esophagus.

[c]Well, well differentiated SCC; Moderate, moderate differentiated SCC; Poorly, poorly differentiated SCC.

Supplementary Table S3: List of the candidate TIA-1 target genes

Gene Symbol	Gene Name	UniGeneID	Fold change (/GAPDH mRNA)	
			Microarray	RIP Seq
AAAS	achalasia, adrenocortical insufficiency, alacrimia	Hs.369144	7.82	4.37
AACS	acetoacetyl-CoA synthetase	Hs.656073	5.54	4.67
AAMP	angio-associated, migratory cell protein	Hs.83347	7.69	6.92
AARS	alanyl-tRNA synthetase	Hs.315137	2.88	4.91
ABCA13	ATP-binding cassette, sub-family A (ABC1), member 13	Hs.226568	19.66	2.67
ABCB7	ATP-binding cassette, sub-family B (MDR/TAP), member 7	Hs.370480	18.17	3.28
ABCC10	ATP-binding cassette, sub-family C (CFTR/MRP), member 10	Hs.55879	2.51	4.99
ABCC4	ATP-binding cassette, sub-family C (CFTR/MRP), member 4	Hs.508423	12.10	18.42
ABCD4	ATP-binding cassette, sub-family D (ALD), member 4	Hs.94395	7.18	5.58
ABCE1	ATP-binding cassette, sub-family E (OABP), member 1	Hs.12013	57.12	27.38
ABCF2	ATP-binding cassette, sub-family F (GCN20), member 2	Hs.654958	6.56	2.92
ABCG1	ATP-binding cassette, sub-family G (WHITE), member 1	Hs.124649	5.57	2.90
ABHD12	abhydrolase domain containing 12	Hs.441550	9.51	6.03
ABHD15	abhydrolase domain containing 15	Hs.106510	4.47	3.40
ABHD16A	abhydrolase domain containing 16A	Hs.388188	9.01	32.60
ABHD3	abhydrolase domain containing 3	Hs.397978	22.28	7.05
ABHD5	abhydrolase domain containing 5	Hs.19385	10.63	2.93
ABL1	c-abl oncogene 1, non-receptor tyrosine kinase	Hs.431048	4.81	3.56
ABL2	v-abl Abelson murine leukemia viral oncogene homolog 2	Hs.159472	4.65	7.75
ABLIM1	actin binding LIM protein 1	Hs.438236	10.26	6.14
ACAD10	acyl-CoA dehydrogenase family, member 10	Hs.331141	2.52	2.97
ACAT1	acetyl-CoA acetyltransferase 1	Hs.232375	18.56	14.72
ACER2	alkaline ceramidase 2	Hs.41379	4.23	8.60
ACIN1	apoptotic chromatin condensation inducer 1	Hs.124490	3.07	23.54
ACLY	ATP citrate lyase	Hs.387567	3.93	3.07
ACN9	ACN9 homolog (S. cerevisiae)	Hs.592269	76.57	3.06
ACO1	aconitase 1, soluble	Hs.567229	9.13	4.10
ACP2	acid phosphatase 2, lysosomal	Hs.532492	6.11	27.60
ACP6	acid phosphatase 6, lysophosphatidic	Hs.562154	6.74	5.66
ACSF2	acyl-CoA synthetase family member 2	Hs.288959	3.92	2.86
ACTG1	actin, gamma 1	Hs.514581	6.74	4.32
ACTL6A	actin-like 6A	Hs.435326	24.74	3.70
ACTN4	actinin, alpha 4	Hs.270291	6.28	8.47
ACTR1B	ARP1 actin-related protein 1 homolog B, centractin beta (yeast)	Hs.98791	9.43	6.88
ACTR2	ARP2 actin-related protein 2 homolog (yeast)	Hs.728857	39.14	10.29
ACTR3	ARP3 actin-related protein 3 homolog (yeast)	Hs.433512	36.72	3.39
ACYP1	acylphosphatase 1, erythrocyte (common) type	Hs.18573	48.17	9.46
ADAM10	ADAM metallopeptidase domain 10	Hs.578508	39.09	18.70
ADAM17	ADAM metallopeptidase domain 17	Hs.404914	20.71	8.57
ADAM19	ADAM metallopeptidase domain 19	Hs.483944	5.97	10.67
ADAM23	ADAM metallopeptidase domain 23	Hs.591643	14.30	13.92
ADAMTS1	ADAM metallopeptidase with thrombospondin type 1 motif, 1	Hs.643357	4.08	4.28
ADARB1	adenosine deaminase, RNA-specific, B1	Hs.474018	4.08	4.32

ADAT1	adenosine deaminase, tRNA-specific 1	Hs.729312	6.26	4.72
ADCK2	aarF domain containing kinase 2	Hs.534141	4.05	10.74
ADCK4	aarF domain containing kinase 4	Hs.130712	2.89	9.27
ADCY6	adenylate cyclase 6	Hs.525401	7.18	5.92
ADI1	acireductone dioxygenase 1	Hs.502773	12.27	3.64
ADIPOR1	adiponectin receptor 1	Hs.5298	7.60	7.39
ADIPOR2	adiponectin receptor 2	Hs.371642	21.62	3.50
ADK	adenosine kinase	Hs.656586	15.13	6.26
ADNP	activity-dependent neuroprotector homeobox	Hs.729009	25.85	5.42
ADPGK	ADP-dependent glucokinase	Hs.730668	24.23	19.41
ADPRHL2	ADP-ribosylhydrolase like 2	Hs.18021	4.46	3.91
ADRM1	adhesion regulating molecule 1	Hs.90107	2.85	2.50
AFG3L2	AFG3 ATPase family gene 3-like 2 (S. cerevisiae)	Hs.726355	4.50	5.15
AFMID	arylformamidase	Hs.558614	8.27	50.88
AGA	aspartylglucosaminidase	Hs.207776	9.56	6.45
AGAP1	ArfGAP with GTPase domain, ankyrin repeat and PH domain 1	Hs.435039	4.71	5.11
AGBL5	ATP/GTP binding protein-like 5	Hs.138207	7.82	8.76
AGK	acylglycerol kinase	Hs.730694	27.94	5.82
AGPAT6	1-acylglycerol-3-phosphate O-acyltransferase 6 (lysophosphatidic acid acyltransferase, zeta)	Hs.355753	4.41	4.60
AGPS	alkylglycerone phosphate synthase	Hs.516543	31.81	15.20
AGR2	anterior gradient 2 homolog (Xenopus laevis)	Hs.530009	15.08	4.75
AGTRAP	angiotensin II receptor-associated protein	Hs.464438	2.65	4.14
AHCY	adenosylhomocysteinase	Hs.388004	2.63	6.00
AHCYL1	adenosylhomocysteinase-like 1	Hs.485365	11.05	2.64
AHR	aryl hydrocarbon receptor	Hs.171189	36.37	7.59
AHSA1	AHA1, activator of heat shock 90 kDa protein ATPase homolog 1 (yeast)	Hs.204041	21.88	13.07
AIF1L	allograft inflammatory factor 1-like	Hs.4944	3.36	3.70
AIMP1	aminoacyl tRNA synthetase complex-interacting multifunctional protein 1	Hs.591680	52.88	4.62
AK2	adenylate kinase 2	Hs.470907	8.02	3.02
AK4	adenylate kinase 4	Hs.10862	14.15	3.66
AKAP1	A kinase (PRKA) anchor protein 1	Hs.463506	4.70	2.79
AKIP1	A kinase (PRKA) interacting protein 1	Hs.131180	10.44	3.17
AKIRIN1	akirin 1	Hs.293563	8.14	3.02
AKIRIN2	akirin 2	Hs.485915	7.45	4.93
AKR1A1	aldo-keto reductase family 1, member A1 (aldehyde reductase)	Hs.474584	2.62	2.79
AKT1S1	AKT1 substrate 1 (proline-rich)	Hs.515542	2.65	14.97
AKTIP	AKT interacting protein	Hs.380897	44.57	17.85
ALAS1	aminolevulinate, delta-, synthase 1	Hs.476308	9.69	7.26
ALDH1A1	aldehyde dehydrogenase 1 family, member A1	Hs.76392	14.25	9.73
ALDH1B1	aldehyde dehydrogenase 1 family, member B1	Hs.436219	6.78	4.78
ALDH2	aldehyde dehydrogenase 2 family (mitochondrial)	Hs.604551	10.41	2.96
ALDH3A1	aldehyde dehydrogenase 3 family, member A1	Hs.531682	4.24	3.93
ALG11	asparagine-linked glycosylation 11, alpha-1,2-mannosyltransferase homolog (yeast)	Hs.512963	10.32	5.05

ALG12	asparagine-linked glycosylation 12, alpha-1, 6-mannosyltransferase homolog (S. cerevisiae)	Hs.526711	5.64	3.64
ALKBH3	alkB, alkylation repair homolog 3 (E. coli)	Hs.720708	5.26	4.59
ALKBH4	alkB, alkylation repair homolog 4 (E. coli)	Hs.658598	5.72	2.60
ALKBH5	alkB, alkylation repair homolog 5 (E. coli)	Hs.730752	2.96	3.79
ALKBH6	alkB, alkylation repair homolog 6 (E. coli)	Hs.71941	7.25	8.04
AMD1	adenosylmethionine decarboxylase 1	Hs.159118	69.64	8.60
AMFR	autocrine motility factor receptor	Hs.295137	3.30	2.73
AMIGO2	adhesion molecule with Ig-like domain 2	Hs.121520	7.49	4.44
AMMECR1	Alport syndrome, mental retardation, midface hypoplasia and elliptocytosis chromosomal region gene 1	Hs.656243	12.65	2.93
ANAPC10	anaphase promoting complex subunit 10	Hs.480876	58.70	10.50
ANAPC13	anaphase promoting complex subunit 13	Hs.106909	8.89	19.63
ANG	angiogenin, ribonuclease, RNase A family, 5	Hs.593708	8.56	4.24
ANKLE2	ankyrin repeat and LEM domain containing 2	Hs.654628	7.72	4.77
ANKRD10	ankyrin repeat domain 10	Hs.525163	20.67	14.21
ANKRD11	ankyrin repeat domain 11	Hs.335003	2.81	7.12
ANKRD13A	ankyrin repeat domain 13A	Hs.528703	8.35	7.70
ANKRD27	ankyrin repeat domain 27 (VPS9 domain)	Hs.59236	5.34	11.95
ANKRD36BP1	ankyrin repeat domain 36B pseudogene 1	Hs.537067	11.00	16.07
ANKRD42	ankyrin repeat domain 42	Hs.503438	3.27	2.51
ANKZF1	ankyrin repeat and zinc finger domain containing 1	Hs.437647	3.93	3.40
ANP32A	acidic (leucine-rich) nuclear phosphoprotein 32 family, member A	Hs.458747	16.22	191.89
ANP32B	acidic (leucine-rich) nuclear phosphoprotein 32 family, member B	Hs.730654	10.51	4.11
ANP32C	acidic (leucine-rich) nuclear phosphoprotein 32 family, member C	Hs.661161	22.43	3.36
ANP32E	acidic (leucine-rich) nuclear phosphoprotein 32 family, member E	Hs.656466	135.73	44.31
ANXA1	annexin A1	Hs.494173	98.06	5.77
ANXA2	annexin A2	Hs.511605	4.45	9.48
ANXA4	annexin A4	Hs.422986	39.80	3.85
AP1G1	adaptor-related protein complex 1, gamma 1 subunit	Hs.461253	37.58	7.74
AP1M2	adaptor-related protein complex 1, mu 2 subunit	Hs.18894	5.42	5.45
AP1S1	adaptor-related protein complex 1, sigma 1 subunit	Hs.489365	5.54	2.59
AP2A1	adaptor-related protein complex 2, alpha 1 subunit	Hs.467125	3.10	7.87
AP2B1	adaptor-related protein complex 2, beta 1 subunit	Hs.514819	12.96	23.11
AP3S1	adaptor-related protein complex 3, sigma 1 subunit	Hs.728956	29.19	4.28
APBB3	amyloid beta (A4) precursor protein-binding, family B, member 3	Hs.529449	4.47	4.81
APEX1	APEX nuclease (multifunctional DNA repair enzyme) 1	Hs.73722	12.26	100.25
APH1A	anterior pharynx defective 1 homolog A (C. elegans)	Hs.108408	10.02	27.38
API5	apoptosis inhibitor 5	Hs.435771	44.03	72.75
APOBEC3C	apolipoprotein B mRNA editing enzyme, catalytic polypeptide-like 3C	Hs.441124	8.87	5.14
APOL6	apolipoprotein L, 6	Hs.257352	4.26	8.48
APOLD1	apolipoprotein L domain containing 1	Hs.23388	7.58	7.23
APTX	aprataxin	Hs.20158	7.57	3.45
AQP3	aquaporin 3 (Gill blood group)	Hs.234642	12.73	20.57
ARAF	v-raf murine sarcoma 3611 viral oncogene homolog	Hs.446641	12.91	9.51
ARCN1	archain 1	Hs.33642	16.50	30.64
AREG	amphiregulin	Hs.270833	25.51	5.09
ARF1	ADP-ribosylation factor 1	Hs.286221	30.95	40.48

ARG2	arginase, type II	Hs.226007	9.80	7.77
ARHGAP17	Rho GTPase activating protein 17	Hs.373793	4.62	4.66
ARHGAP19	Rho GTPase activating protein 19	Hs.80305	9.07	8.82
ARHGAP8	Rho GTPase activating protein 8	Hs.102336	2.95	5.59
ARHGDIA	Rho GDP dissociation inhibitor (GDI) alpha	Hs.159161	3.45	3.13
ARHGEF11	Rho guanine nucleotide exchange factor (GEF) 11	Hs.516954	5.04	10.77
ARHGEF19	Rho guanine nucleotide exchange factor (GEF) 19	Hs.591532	2.74	2.69
ARHGEF25	Rho guanine nucleotide exchange factor (GEF) 25	Hs.61581	5.22	19.20
ARL1	ADP-ribosylation factor-like 1	Hs.372616	113.59	3.83
ARL10	ADP-ribosylation factor-like 10	Hs.424552	2.64	7.88
ARL17B	ADP-ribosylation factor-like 17B	Hs.720602	5.53	5.96
ARL4A	ADP-ribosylation factor-like 4A	Hs.245540	19.95	12.14
ARL6IP1	ADP-ribosylation factor-like 6 interacting protein 1	Hs.634882	77.74	39.51
ARL6IP5	ADP-ribosylation-like factor 6 interacting protein 5	Hs.730695	26.24	3.40
ARL8B	ADP-ribosylation factor-like 8B	Hs.250009	74.20	18.51
ARMC10	armadillo repeat containing 10	Hs.287412	15.87	2.67
ARMC9	armadillo repeat containing 9	Hs.471610	9.47	5.21
ARMCX6	armadillo repeat containing, X-linked 6	Hs.83530	15.25	10.51
ARNT	aryl hydrocarbon receptor nuclear translocator	Hs.632446	4.14	6.29
ARNTL2	aryl hydrocarbon receptor nuclear translocator-like 2	Hs.663740	33.36	19.43
ARPC1A	actin related protein 2/3 complex, subunit 1A, 41 kDa	Hs.124126	5.77	3.17
ARPC1B	actin related protein 2/3 complex, subunit 1B, 41 kDa	Hs.489284	4.35	3.88
ARPC2	actin related protein 2/3 complex, subunit 2, 34 kDa	Hs.529303	17.23	4.15
ARPC4	actin related protein 2/3 complex, subunit 4, 20 kDa	Hs.323342	7.73	45.10
ARPC5L	actin related protein 2/3 complex, subunit 5-like	Hs.132499	4.49	3.05
ARPP19	cAMP-regulated phosphoprotein, 19 kDa	Hs.713393	69.51	11.29
ARRDC2	arrestin domain containing 2	Hs.515249	5.97	7.99
ARSB	arylsulfatase B	Hs.149103	4.96	24.17
ARSK	arylsulfatase family, member K	Hs.585051	8.49	11.00
ASAH1	N-acylsphingosine amidohydrolase (acid ceramidase) 1	Hs.527412	85.80	2.67
ASB3	ankyrin repeat and SOCS box containing 3	Hs.40763	43.03	4.48
ASB6	ankyrin repeat and SOCS box containing 6	Hs.125037	2.74	10.76
ASCC1	activating signal cointegrator 1 complex subunit 1	Hs.500007	25.58	10.42
ASCC3	activating signal cointegrator 1 complex subunit 3	Hs.486031	5.46	2.82
ASNA1	arsA arsenite transporter, ATP-binding, homolog 1 (bacterial)	Hs.465985	5.22	2.87
ASPH	aspartate beta-hydroxylase	Hs.332422	12.79	18.62
ASPRV1	aspartic peptidase, retroviral-like 1	Hs.516253	3.18	4.07
ASXL2	additional sex combs like 2 (Drosophila)	Hs.119815	10.75	8.45
ATAD1	ATPase family, AAA domain containing 1	Hs.435948	117.15	7.08
ATAT1	alpha tubulin acetyltransferase 1	Hs.654798	8.25	4.25
ATF3	activating transcription factor 3	Hs.460	6.55	4.70
ATF5	activating transcription factor 5	Hs.9754	2.74	10.09
ATG13	ATG13 autophagy related 13 homolog (S. cerevisiae)	Hs.127403	9.84	7.15
ATG16L1	ATG16 autophagy related 16-like 1 (S. cerevisiae)	Hs.529322	5.64	8.60
ATG3	ATG3 autophagy related 3 homolog (S. cerevisiae)	Hs.477126	23.15	4.98
ATG4A	ATG4 autophagy related 4 homolog A (S. cerevisiae)	Hs.8763	18.99	5.58
ATG7	ATG7 autophagy related 7 homolog (S. cerevisiae)	Hs.730676	2.92	7.44

ATG9A	ATG9 autophagy related 9 homolog A (S. cerevisiae)	Hs.323363	4.15	9.89
ATL2	atlastin GTPase 2	Hs.727652	44.30	73.57
ATMIN	ATM interactor	Hs.16349	7.36	9.69
ATN1	atrophin 1	Hs.143766	5.89	5.91
ATP13A3	ATPase type 13A3	Hs.529609	28.60	15.65
ATP2A2	ATPase, Ca++ transporting, cardiac muscle, slow twitch 2	Hs.506759	8.00	7.46
ATP2B4	ATPase, Ca++ transporting, plasma membrane 4	Hs.343522	3.62	4.51
ATP5C1	ATP synthase, H+ transporting, mitochondrial F1 complex, gamma polypeptide 1	Hs.271135	19.39	24.41
ATP5L2	ATP synthase, H+ transporting, mitochondrial Fo complex, subunit G2	Hs.664737	3.67	3.74
ATP5S	ATP synthase, H+ transporting, mitochondrial Fo complex, subunit s (factor B)	Hs.438489	9.43	3.49
ATP6V0E1	ATPase, H+ transporting, lysosomal 9 kDa, V0 subunit e1	Hs.484188	11.99	12.21
ATP6V1B2	ATPase, H+ transporting, lysosomal 56/58 kDa, V1 subunit B2	Hs.295917	17.55	9.14
ATP6V1C2	ATPase, H+ transporting, lysosomal 42 kDa, V1 subunit C2	Hs.580464	3.12	239.02
ATP6V1E1	ATPase, H+ transporting, lysosomal 31 kDa, V1 subunit E1	Hs.517338	20.72	21.18
ATP8B1	ATPase, aminophospholipid transporter, class I, type 8B, member 1	Hs.216623	42.50	2.69
ATP9B	ATPase, class II, type 9B	Hs.465475	12.22	6.25
ATRN	attractin	Hs.276252	3.99	5.24
ATXN2L	ataxin 2-like	Hs.460499	6.10	6.15
ATXN7L3	ataxin 7-like 3	Hs.512651	4.41	5.54
ATXN7L3B	ataxin 7-like 3B	Hs.727494	16.64	3.27
AURKA	aurora kinase A	Hs.250822	19.56	6.28
AURKAPS1	aurora kinase A pseudogene 1	Hs.654849	9.91	8.52
AURKB	aurora kinase B	Hs.442658	9.24	19.54
AVL9	AVL9 homolog (S. cerevisiase)	Hs.128056	7.85	8.22
AXIN1	axin 1	Hs.592082	4.75	14.75
AZIN1	antizyme inhibitor 1	Hs.459106	85.90	14.95
B2M	beta-2-microglobulin	Hs.534255	24.21	17.09
B3GALNT2	beta-1,3-N-acetylgalactosaminyltransferase 2	Hs.730772	9.83	10.42
B3GNT9	UDP-GlcNAc:betaGal beta-1, 3-N-acetylglucosaminyltransferase 9	Hs.513666	8.99	6.83
B3GNTL1	UDP-GlcNAc:betaGal beta-1, 3-N-acetylglucosaminyltransferase-like 1	Hs.730724	2.77	2.54
B4GALNT4	beta-1,4-N-acetyl-galactosaminyl transferase 4	Hs.148074	4.08	14.56
B4GALT1	UDP-Gal:betaGlcNAc beta 1, 4- galactosyltransferase, polypeptide 1	Hs.272011	6.41	13.83
B4GALT4	UDP-Gal:betaGlcNAc beta 1, 4- galactosyltransferase, polypeptide 4	Hs.13225	79.39	34.63
B4GALT7	xylosylprotein beta 1, 4-galactosyltransferase, polypeptide 7 (galactosyltransferase I)	Hs.455109	5.14	4.00
BAG2	BCL2-associated athanogene 2	Hs.729098	32.90	2.92
BAG3	BCL2-associated athanogene 3	Hs.523309	7.76	5.68
BAIAP2	BAI1-associated protein 2	Hs.128316	3.89	6.42
BANF1	barrier to autointegration factor 1	Hs.433759	16.23	9.84
BANP	BTG3 associated nuclear protein	Hs.461705	4.03	3.54
BARX1	BARX homeobox 1	Hs.164960	5.53	8.91
BAX	BCL2-associated X protein	Hs.624291	4.32	6.24
BAZ1B	bromodomain adjacent to zinc finger domain, 1B	Hs.647016	26.01	10.74
BAZ2A	bromodomain adjacent to zinc finger domain, 2A	Hs.314263	6.01	5.83
BBS2	Bardet-Biedl syndrome 2	Hs.333738	24.84	8.08
BBS4	Bardet-Biedl syndrome 4	Hs.208681	18.51	4.73

BBS5	Bardet-Biedl syndrome 5	Hs.233398	3.64	7.60
BCAM	basal cell adhesion molecule (Lutheran blood group)	Hs.625725	3.32	71.32
BCAS2	breast carcinoma amplified sequence 2	Hs.22960	64.96	7.51
BCAS3	breast carcinoma amplified sequence 3	Hs.655028	3.59	9.12
BCAS4	breast carcinoma amplified sequence 4	Hs.381178	9.68	24.54
BCAT1	branched chain amino-acid transaminase 1, cytosolic	Hs.438993	40.04	30.16
BCL11A	B-cell CLL/lymphoma 11A (zinc finger protein)	Hs.370549	9.34	13.85
BCL2L13	BCL2-like 13 (apoptosis facilitator)	Hs.631672	7.35	7.12
BCL2L2	BCL2-like 2	Hs.410026	14.69	2.91
BCL7A	B-cell CLL/lymphoma 7A	Hs.530970	8.26	3.05
BCL7B	B-cell CLL/lymphoma 7B	Hs.647051	20.36	6.33
BECN1	beclin 1, autophagy related	Hs.716464	9.99	2.63
BHLHE40	basic helix-loop-helix family, member e40	Hs.171825	10.39	16.18
BIN1	bridging integrator 1	Hs.193163	2.52	6.69
BLCAP	bladder cancer associated protein	Hs.472651	5.54	6.23
BLMH	bleomycin hydrolase	Hs.371914	31.85	6.37
BLOC1S2	biogenesis of lysosomal organelles complex-1, subunit 2	Hs.34906	75.01	9.22
BMP1	bone morphogenetic protein 1	Hs.1274	3.99	18.32
BMPR1A	bone morphogenetic protein receptor, type IA	Hs.524477	57.19	17.37
BMS1	BMS1 homolog, ribosome assembly protein (yeast)	Hs.10848	8.70	8.75
BNIP1	BCL2/adenovirus E1B 19 kDa interacting protein 1	Hs.145726	42.78	16.79
BNIP3L	BCL2/adenovirus E1B 19 kDa interacting protein 3-like	Hs.131226	59.68	28.65
BORA	bora, aurora kinase A activator	Hs.643464	84.74	9.88
BPNT1	3′(2′), 5′-bisphosphate nucleotidase 1	Hs.406134	11.63	16.05
BRAF	v-raf murine sarcoma viral oncogene homolog B1	Hs.550061	5.95	3.34
BRD4	bromodomain containing 4	Hs.187763	3.74	11.81
BRD7	bromodomain containing 7	Hs.437894	11.14	7.23
BRE	brain and reproductive organ-expressed (TNFRSF1A modulator)	Hs.258314	6.90	2.63
BRF2	BRF2, subunit of RNA polymerase III transcription initiation factor, BRF1-like	Hs.709301	9.23	2.93
BRIX1	BRX1, biogenesis of ribosomes, homolog (S. cerevisiae)	Hs.718510	34.41	9.88
BRK1	BRICK1, SCAR/WAVE actin-nucleating complex subunit	Hs.678537	7.18	5.51
BRMS1	breast cancer metastasis suppressor 1	Hs.100426	4.53	2.80
BSCL2	Berardinelli-Seip congenital lipodystrophy 2 (seipin)	Hs.533709	5.72	3.10
BSDC1	BSD domain containing 1	Hs.353454	7.08	22.51
BSG	basigin (Ok blood group)	Hs.501293	5.21	5.54
BTBD2	BTB (POZ) domain containing 2	Hs.465543	5.63	3.57
BTBD7	BTB (POZ) domain containing 7	Hs.525549	11.59	9.41
BTF3	basic transcription factor 3	Hs.591768	12.94	18.94
BTG1	B-cell translocation gene 1, anti-proliferative	Hs.255935	17.70	4.22
BTN2A2	butyrophilin, subfamily 2, member A2	Hs.373938	7.88	19.78
BTN3A1	butyrophilin, subfamily 3, member A1	Hs.191510	4.26	12.00
BTN3A2	butyrophilin, subfamily 3, member A2	Hs.376046	10.71	3.09
BUB1	budding uninhibited by benzimidazoles 1 homolog (yeast)	Hs.469649	38.01	14.11
BUB1B	budding uninhibited by benzimidazoles 1 homolog beta (yeast)	Hs.513645	44.99	10.98
BUD31	BUD31 homolog (S. cerevisiae)	Hs.380233	8.20	4.97
C1D	C1D nuclear receptor corepressor	Hs.602900	26.49	9.94
C1GALT1C1	C1GALT1-specific chaperone 1	Hs.643920	38.74	4.28

CA11	carbonic anhydrase XI	Hs.428446	9.97	4.97
CA9	carbonic anhydrase IX	Hs.63287	5.11	14.86
CABLES2	Cdk5 and Abl enzyme substrate 2	Hs.301040	6.08	2.86
CABYR	calcium binding tyrosine-(Y)-phosphorylation regulated	Hs.511983	14.50	15.17
CACHD1	cache domain containing 1	Hs.443891	5.23	7.50
CACYBP	calcyclin binding protein	Hs.508524	12.67	13.07
CALB1	calbindin 1, 28kDa	Hs.65425	191.56	9.57
CALM2	calmodulin 2 (phosphorylase kinase, delta)	Hs.468442	38.29	6.85
CALM3	calmodulin 3 (phosphorylase kinase, delta)	Hs.515487	4.26	3.74
CALML4	calmodulin-like 4	Hs.709550	5.55	12.46
CALU	calumenin	Hs.592258	39.86	12.13
CAMK2D	calcium/calmodulin-dependent protein kinase II delta	Hs.144114	8.40	17.55
CAMK2G	calcium/calmodulin-dependent protein kinase II gamma	Hs.523045	13.33	8.17
CAMKMT	calmodulin-lysine N-methyltransferase	Hs.468349	5.77	12.11
CANX	calnexin	Hs.567968	17.35	16.28
CAP1	CAP, adenylate cyclase-associated protein 1 (yeast)	Hs.370581	7.14	16.39
CAPN1	calpain 1, (mu/I) large subunit	Hs.502842	2.65	4.52
CAPN10	calpain 10	Hs.728234	5.64	6.20
CAPNS2	calpain, small subunit 2	Hs.660027	5.58	9.45
CAPZA1	capping protein (actin filament) muscle Z-line, alpha 1	Hs.514934	221.15	24.10
CARD11	caspase recruitment domain family, member 11	Hs.648101	3.02	6.73
CARKD	carbohydrate kinase domain containing	Hs.408324	7.71	13.49
CARS	cysteinyl-tRNA synthetase	Hs.274873	5.27	17.62
CASP2	caspase 2, apoptosis-related cysteine peptidase	Hs.368982	4.72	3.01
CASP9	caspase 9, apoptosis-related cysteine peptidase	Hs.329502	12.65	3.64
CAV1	caveolin 1, caveolae protein, 22 kDa	Hs.74034	23.73	11.61
CAV2	caveolin 2	Hs.212332	46.43	23.02
CBFA2T2	core-binding factor, runt domain, alpha subunit 2; translocated to, 2	Hs.153934	3.29	3.46
CBFB	core-binding factor, beta subunit	Hs.460988	57.71	11.38
CBR1	carbonyl reductase 1	Hs.88778	6.14	7.36
CBWD5	COBW domain containing 5	Hs.645337	24.88	8.48
CBX1	chromobox homolog 1	Hs.77254	24.54	9.00
CBX3	chromobox homolog 3	Hs.381189	17.43	23.81
CBX4	chromobox homolog 4	Hs.730763	8.22	17.89
CBX5	chromobox homolog 5	Hs.349283	21.86	4.50
CBX8	chromobox homolog 8	Hs.387258	17.15	9.93
CC2D1A	coiled-coil and C2 domain containing 1A	Hs.269592	2.69	3.20
CCBL1	cysteine conjugate-beta lyase, cytoplasmic	Hs.495250	5.36	3.30
CCBL2	cysteine conjugate-beta lyase 2	Hs.481898	32.35	22.93
CCDC104	coiled-coil domain containing 104	Hs.264208	22.39	8.78
CCDC109B	coiled-coil domain containing 109B	Hs.234149	32.22	8.51
CCDC115	coiled-coil domain containing 115	Hs.104203	18.93	11.49
CCDC117	coiled-coil domain containing 117	Hs.406460	37.12	15.61
CCDC134	coiled-coil domain containing 134	Hs.474991	2.53	5.71
CCDC136	coiled-coil domain containing 136	Hs.521178	3.27	5.57
CCDC24	coiled-coil domain containing 24	Hs.632394	4.40	10.11
CCDC28A	coiled-coil domain containing 28A	Hs.412019	32.03	4.27

CCDC47	coiled-coil domain containing 47	Hs.202011	24.57	39.80
CCDC50	coiled-coil domain containing 50	Hs.478682	16.35	17.32
CCDC53	coiled-coil domain containing 53	Hs.405692	52.02	6.12
CCDC58	coiled-coil domain containing 58	Hs.220594	28.75	41.19
CCDC59	coiled-coil domain containing 59	Hs.582627	72.49	14.15
CCDC88C	coiled-coil domain containing 88C	Hs.525536	2.85	2.99
CCDC92	coiled-coil domain containing 92	Hs.114111	10.98	5.79
CCL5	chemokine (C-C motif) ligand 5	Hs.514821	7.23	7.77
CCNA2	cyclin A2	Hs.58974	24.29	12.16
CCND1	cyclin D1	Hs.523852	12.75	11.39
CCNF	cyclin F	Hs.1973	3.61	8.39
CCNG1	cyclin G1	Hs.79101	10.74	10.79
CCNH	cyclin H	Hs.292524	40.33	9.27
CCNK	cyclin K	Hs.510409	10.30	8.19
CCNL1	cyclin L1	Hs.4859	4.63	2.92
CCNL2	cyclin L2	Hs.515704	3.37	2.82
CCNT1	cyclin T1	Hs.279906	13.77	13.56
CCNY	cyclin Y	Hs.14745	15.74	12.09
CCT6A	chaperonin containing TCP1, subunit 6A (zeta 1)	Hs.82916	38.07	5.06
CCT8	chaperonin containing TCP1, subunit 8 (theta)	Hs.125113	56.18	8.39
CCZ1	CCZ1 vacuolar protein trafficking and biogenesis associated homolog (S. cerevisiae)	Hs.530000	8.64	33.04
CD164	CD164 molecule, sialomucin	Hs.520313	38.68	31.93
CD2BP2	CD2 (cytoplasmic tail) binding protein 2	Hs.202677	3.55	6.52
CD302	CD302 molecule	Hs.130014	43.66	9.30
CD38	CD38 molecule	Hs.479214	10.79	3.20
CD3EAP	CD3e molecule, epsilon associated protein	Hs.710495	17.48	5.44
CD44	CD44 molecule (Indian blood group)	Hs.502328	21.39	5.77
CD46	CD46 molecule, complement regulatory protein	Hs.510402	10.32	23.14
CD47	CD47 molecule	Hs.446414	39.19	9.51
CD55	CD55 molecule, decay accelerating factor for complement (Cromer blood group)	Hs.126517	12.15	6.86
CD58	CD58 molecule	Hs.34341	63.34	11.10
CD9	CD9 molecule	Hs.114286	18.11	55.10
CD99	CD99 molecule	Hs.653349	8.35	13.16
CD99L2	CD99 molecule-like 2	Hs.522805	4.54	3.94
CDC123	cell division cycle 123 homolog (S. cerevisiae)	Hs.412842	24.22	10.74
CDC20	cell division cycle 20 homolog (S. cerevisiae)	Hs.524947	2.84	2.62
CDC23	cell division cycle 23 homolog (S. cerevisiae)	Hs.73625	17.52	8.64
CDC25C	cell division cycle 25 homolog C (S. pombe)	Hs.656	8.50	3.00
CDC26	cell division cycle 26 homolog (S. cerevisiae)	Hs.727648	16.61	2.86
CDC27	cell division cycle 27 homolog (S. cerevisiae)	Hs.463295	33.80	22.76
CDC37	cell division cycle 37 homolog (S. cerevisiae)	Hs.160958	5.33	6.13
CDC40	cell division cycle 40 homolog (S. cerevisiae)	Hs.428147	26.35	10.74
CDC42	cell division cycle 42 (GTP binding protein, 25 kDa)	Hs.467637	7.02	23.06
CDC42EP4	CDC42 effector protein (Rho GTPase binding) 4	Hs.3903	6.69	3.58
CDCA2	cell division cycle associated 2	Hs.33366	50.47	5.72
CDCA4	cell division cycle associated 4	Hs.34045	15.39	5.97

CDCA7	cell division cycle associated 7	Hs.470654	25.96	6.31
CDH13	cadherin 13, H-cadherin (heart)	Hs.654386	23.87	5.26
CDH24	cadherin 24, type 2	Hs.155912	4.76	6.64
CDIPT	CDP-diacylglycerol--inositol 3-phosphatidyltransferase	Hs.692949	6.25	10.92
CDK11A	cyclin-dependent kinase 11A	Hs.651228	8.31	4.33
CDK11B	cyclin-dependent kinase 11B	Hs.709182	3.07	2.53
CDK2	cyclin-dependent kinase 2	Hs.19192	5.60	22.72
CDK5	cyclin-dependent kinase 5	Hs.647078	4.98	10.75
CDK5RAP3	CDK5 regulatory subunit associated protein 3	Hs.20157	5.35	7.64
CDK6	cyclin-dependent kinase 6	Hs.119882	69.99	12.00
CDK7	cyclin-dependent kinase 7	Hs.184298	43.76	7.33
CDKN1B	cyclin-dependent kinase inhibitor 1B (p27, Kip1)	Hs.238990	10.65	7.50
CDKN2AIPNL	CDKN2A interacting protein N-terminal like	Hs.156506	8.71	4.50
CDKN3	cyclin-dependent kinase inhibitor 3	Hs.84113	14.15	9.16
CDV3	CDV3 homolog (mouse)	Hs.518265	3.90	3.52
CDYL	chromodomain protein, Y-like	Hs.269092	11.54	7.12
CEL	carboxyl ester lipase (bile salt-stimulated lipase)	Hs.533258	7.97	3.93
CENPA	centromere protein A	Hs.1594	16.93	3.42
CENPB	centromere protein B, 80kDa	Hs.516855	3.75	4.65
CENPF	centromere protein F, 350/400kDa (mitosin)	Hs.497741	46.39	9.04
CENPO	centromere protein O	Hs.467898	3.86	3.09
CENPP	centromere protein P	Hs.713775	7.77	3.58
CENPW	centromere protein W	Hs.486401	11.84	3.28
CEP250	centrosomal protein 250 kDa	Hs.443976	3.40	3.44
CEP63	centrosomal protein 63 kDa	Hs.443301	20.17	11.04
CEP85	centrosomal protein 85 kDa	Hs.63795	5.78	3.55
CEP95	centrosomal protein 95 kDa	Hs.569713	8.62	5.02
CEPT1	choline/ethanolamine phosphotransferase 1	Hs.363572	22.28	14.79
CERK	ceramide kinase	Hs.200668	7.44	4.63
CERS2	ceramide synthase 2	Hs.730616	21.44	6.89
CES2	carboxylesterase 2	Hs.282975	7.26	3.02
CETN2	centrin, EF-hand protein, 2	Hs.82794	51.66	6.00
CFDP1	craniofacial development protein 1	Hs.461361	31.03	11.12
CFL1	cofilin 1 (non-muscle)	Hs.170622	7.18	17.52
CGB	chorionic gonadotropin, beta polypeptide	Hs.446683	3.19	3.73
CHAD	chondroadherin	Hs.97220	3.66	12.85
CHAF1A	chromatin assembly factor 1, subunit A (p150)	Hs.79018	3.60	2.64
CHCHD3	coiled-coil-helix-coiled-coil-helix domain containing 3	Hs.655010	12.98	4.03
CHD1L	chromodomain helicase DNA binding protein 1-like	Hs.191164	6.83	3.93
CHD4	chromodomain helicase DNA binding protein 4	Hs.162233	6.84	6.42
CHEK1	checkpoint kinase 1	Hs.24529	20.88	19.51
CHEK2	checkpoint kinase 2	Hs.291363	24.74	9.02
CHERP	calcium homeostasis endoplasmic reticulum protein	Hs.631627	4.02	7.29
CHIC2	cysteine-rich hydrophobic domain 2	Hs.335393	7.06	4.61
CHMP1A	charged multivesicular body protein 1A	Hs.589427	3.18	4.75
CHMP4B	charged multivesicular body protein 4B	Hs.472471	4.95	5.54
CHMP5	charged multivesicular body protein 5	Hs.635313	34.82	4.00

CHMP6	charged multivesicular body protein 6	Hs.514560	2.74	2.91
CHMP7	charged multivesicular body protein 7	Hs.5019	5.97	6.32
CHPT1	choline phosphotransferase 1	Hs.293077	14.92	17.30
CHST14	carbohydrate (N-acetylgalactosamine 4-0) sulfotransferase 14	Hs.442449	19.46	62.73
CHST3	carbohydrate (chondroitin 6) sulfotransferase 3	Hs.158304	3.16	4.89
CHTOP	chromatin target of PRMT1	Hs.728799	3.73	19.70
CIDEB	cell death-inducing DFFA-like effector b	Hs.642693	9.38	20.44
CINP	cyclin-dependent kinase 2 interacting protein	Hs.129634	6.58	9.47
CISD1	CDGSH iron sulfur domain 1	Hs.370102	12.46	4.43
CISD2	CDGSH iron sulfur domain 2	Hs.444955	29.06	7.00
CITED2	Cbp/p300-interacting transactivator, with Glu/Asp-rich carboxy-terminal domain, 2	Hs.82071	6.71	8.25
CKAP5	cytoskeleton associated protein 5	Hs.201253	58.63	11.01
CKLF	chemokine-like factor	Hs.15159	22.35	7.07
CKS1B	CDC28 protein kinase regulatory subunit 1B	Hs.374378	5.49	3.01
CLASP2	cytoplasmic linker associated protein 2	Hs.108614	11.76	8.15
CLCC1	chloride channel CLIC-like 1	Hs.658489	17.56	27.37
CLCF1	cardiotrophin-like cytokine factor 1	Hs.502977	6.12	2.75
CLDN12	claudin 12	Hs.258576	16.24	16.09
CLDN22	claudin 22	Hs.333179	11.11	3.03
CLDND1	claudin domain containing 1	Hs.531371	88.98	12.24
CLIC1	chloride intracellular channel 1	Hs.414565	8.59	3.33
CLIC4	chloride intracellular channel 4	Hs.440544	23.78	11.63
CLINT1	clathrin interactor 1	Hs.644000	30.30	5.32
CLIP2	CAP-GLY domain containing linker protein 2	Hs.647018	3.08	5.90
CLIP4	CAP-GLY domain containing linker protein family, member 4	Hs.122927	16.54	9.75
CLK2	CDC-like kinase 2	Hs.73986	7.03	5.93
CLN3	ceroid-lipofuscinosis, neuronal 3	Hs.534667	4.18	5.16
CLN5	ceroid-lipofuscinosis, neuronal 5	Hs.30213	21.12	4.26
CLPTM1	cleft lip and palate associated transmembrane protein 1	Hs.444441	3.21	5.47
CLPTM1L	CLPTM1-like	Hs.444673	6.62	2.70
CLPX	ClpX caseinolytic peptidase X homolog (E. coli)	Hs.113823	34.08	11.47
CLUAP1	clusterin associated protein 1	Hs.155995	30.62	5.87
CMAS	cytidine monophosphate N-acetylneuraminic acid synthetase	Hs.311346	28.97	34.06
CMIP	c-Maf inducing protein	Hs.594095	14.39	3.57
CMPK1	cytidine monophosphate (UMP-CMP) kinase 1, cytosolic	Hs.11463	31.49	30.07
CMTM6	CKLF-like MARVEL transmembrane domain containing 6	Hs.380627	50.07	27.92
CNN3	calponin 3, acidic	Hs.483454	19.18	11.39
CNNM2	cyclin M2	Hs.730766	3.33	6.57
CNOT6L	CCR4-NOT transcription complex, subunit 6-like	Hs.592519	18.71	13.05
CNOT7	CCR4-NOT transcription complex, subunit 7	Hs.645009	57.88	13.74
CNPPD1	cyclin Pas1/PHO80 domain containing 1	Hs.4973	6.56	7.21
CNPY3	canopy 3 homolog (zebrafish)	Hs.414099	4.78	11.67
CNTN1	contactin 1	Hs.143434	13.54	31.89
CNTNAP1	contactin associated protein 1	Hs.408730	7.65	3.43
CNTROB	centrobin, centrosomal BRCA2 interacting protein	Hs.348012	4.01	3.06
COA5	cytochrome C oxidase assembly factor 5	Hs.596537	60.35	18.44
COG2	component of oligomeric golgi complex 2	Hs.211800	10.83	3.26

COG5	component of oligomeric golgi complex 5	Hs.239631	5.88	18.96
COIL	coilin	Hs.532795	42.14	3.21
COL10A1	collagen, type X, alpha 1	Hs.520339	5.75	7.09
COL4A6	collagen, type IV, alpha 6	Hs.145586	5.47	5.26
COL5A1	collagen, type V, alpha 1	Hs.210283	5.37	4.29
COMMD10	COMM domain containing 10	Hs.483136	40.22	7.56
COMMD2	COMM domain containing 2	Hs.432729	24.57	44.79
COMMD7	COMM domain containing 7	Hs.408427	4.65	4.18
COMMD9	COMM domain containing 9	Hs.279836	8.55	3.15
COMT	catechol-O-methyltransferase	Hs.370408	13.98	7.65
COPB1	coatomer protein complex, subunit beta 1	Hs.339278	201.19	8.10
COPG2	coatomer protein complex, subunit gamma 2	Hs.6421	6.23	5.95
COPS4	COP9 constitutive photomorphogenic homolog subunit 4 (Arabidopsis)	Hs.190384	36.04	2.85
COPS7A	COP9 constitutive photomorphogenic homolog subunit 7A (Arabidopsis)	Hs.530823	9.58	5.56
COQ2	coenzyme Q2 homolog, prenyltransferase (yeast)	Hs.729069	11.50	19.15
COQ6	coenzyme Q6 homolog, monooxygenase (S. cerevisiae)	Hs.131555	32.44	28.08
COQ7	coenzyme Q7 homolog, ubiquinone (yeast)	Hs.157113	16.46	5.01
COQ9	coenzyme Q9 homolog (S. cerevisiae)	Hs.513632	12.47	3.54
CORO1B	coronin, actin binding protein, 1B	Hs.6191	4.59	17.98
CORO1C	coronin, actin binding protein, 1C	Hs.330384	13.17	5.45
COX11	COX11 cytochrome c oxidase assembly homolog (yeast)	Hs.591171	14.00	10.69
COX17	COX17 cytochrome c oxidase assembly homolog (S. cerevisiae)	Hs.534383	10.52	4.92
COX3	cytochrome c oxidase III	Hs.631493	3.80	18.51
COX7A2L	cytochrome c oxidase subunit VIIa polypeptide 2 like	Hs.339639	10.60	5.18
CPNE2	copine II	Hs.339809	13.16	50.32
CPSF1	cleavage and polyadenylation specific factor 1, 160 kDa	Hs.493202	2.72	5.62
CPSF3	cleavage and polyadenylation specific factor 3, 73 kDa	Hs.515972	33.57	16.74
CPSF6	cleavage and polyadenylation specific factor 6, 68 kDa	Hs.369606	12.16	9.12
CPVL	carboxypeptidase, vitellogenic-like	Hs.233389	10.88	5.23
CRCP	CGRP receptor component	Hs.571280	8.17	11.62
CRELD1	cysteine-rich with EGF-like domains 1	Hs.9383	7.51	3.91
CRIP2	cysteine-rich protein 2	Hs.534309	2.66	5.26
CRIPT	cysteine-rich PDZ-binding protein	Hs.133998	30.45	4.42
CRKL	v-crk sarcoma virus CT10 oncogene homolog (avian)-like	Hs.5613	15.27	10.02
CRLF3	cytokine receptor-like factor 3	Hs.370168	4.86	9.37
CRYBB2	crystallin, beta B2	Hs.373074	5.71	4.55
CSAG3	CSAG family, member 3	Hs.522810	4.80	18.89
CSE1L	CSE1 chromosome segregation 1-like (yeast)	Hs.90073	67.99	9.10
CSF1	colony stimulating factor 1 (macrophage)	Hs.591402	3.64	10.99
CSK	c-src tyrosine kinase	Hs.77793	5.56	9.58
CSNK1D	casein kinase 1, delta	Hs.631725	7.04	5.17
CSNK2A1	casein kinase 2, alpha 1 polypeptide	Hs.644056	5.73	15.19
CSNK2A2	casein kinase 2, alpha prime polypeptide	Hs.82201	14.20	17.20
CSPG5	chondroitin sulfate proteoglycan 5 (neuroglycan C)	Hs.45127	4.25	3.35
CSTA	cystatin A (stefin A)	Hs.518198	14.66	6.39
CSTB	cystatin B (stefin B)	Hs.695	17.96	4.07

CT45A5	cancer/testis antigen family 45, member A5	Hs.535081	7.32	9.00
CTBP1	C-terminal binding protein 1	Hs.208597	4.62	15.31
CTBP2	C-terminal binding protein 2	Hs.501345	9.34	6.15
CTBS	chitobiase, di-N-acetyl-	Hs.513557	61.06	7.67
CTDNEP1	CTD nuclear envelope phosphatase 1	Hs.513913	6.72	3.36
CTDP1	CTD (carboxy-terminal domain, RNA polymerase II, polypeptide A) phosphatase, subunit 1	Hs.465490	4.40	5.38
CTDSP1	CTD (carboxy-terminal domain, RNA polymerase II, polypeptide A) small phosphatase 1	Hs.444468	5.82	3.45
CTDSPL	CTD (carboxy-terminal domain, RNA polymerase II, polypeptide A) small phosphatase-like	Hs.475963	6.79	3.72
CTF1	cardiotrophin 1	Hs.483811	10.26	2.80
CTH	cystathionase (cystathionine gamma-lyase)	Hs.19904	28.34	3.06
CTHRC1	collagen triple helix repeat containing 1	Hs.405614	17.43	16.83
CTNNAL1	catenin (cadherin-associated protein), alpha-like 1	Hs.58488	19.29	2.98
CTNNB1	catenin (cadherin-associated protein), beta 1, 88 kDa	Hs.476018	4.38	4.59
CTNNBIP1	catenin, beta interacting protein 1	Hs.463759	9.04	4.72
CTNND1	catenin (cadherin-associated protein), delta 1	Hs.166011	12.30	17.68
CTSC	cathepsin C	Hs.128065	14.71	41.30
CTSZ	cathepsin Z	Hs.252549	3.28	15.25
CTTN	cortactin	Hs.596164	5.25	2.97
CTU2	cytosolic thiouridylase subunit 2 homolog (S. pombe)	Hs.592074	2.52	2.57
CUL1	cullin 1	Hs.146806	11.03	4.87
CUL4A	cullin 4A	Hs.339735	18.82	7.05
CUX1	cut-like homeobox 1	Hs.191482	8.25	11.36
CWC15	CWC15 spliceosome-associated protein homolog (S. cerevisiae)	Hs.503597	20.30	4.83
CWF19L1	CWF19-like 1, cell cycle control (S. pombe)	Hs.215502	10.05	12.02
CWF19L2	CWF19-like 2, cell cycle control (S. pombe)	Hs.212140	12.37	4.70
CXADR	coxsackie virus and adenovirus receptor	Hs.634837	29.26	25.79
CXCL1	chemokine (C-X-C motif) ligand 1 (melanoma growth stimulating activity, alpha)	Hs.789	17.61	2.73
CXCL16	chemokine (C-X-C motif) ligand 16	Hs.730800	2.56	3.57
CXCR3	chemokine (C-X-C motif) receptor 3	Hs.198252	2.94	4.63
CYB5A	cytochrome b5 type A (microsomal)	Hs.465413	25.20	7.03
CYB5D1	cytochrome b5 domain containing 1	Hs.27475	8.11	24.33
CYB5D2	cytochrome b5 domain containing 2	Hs.513871	5.61	3.21
CYBRD1	cytochrome b reductase 1	Hs.221941	15.40	4.09
CYP1A1	cytochrome P450, family 1, subfamily A, polypeptide 1	Hs.72912	10.83	3.22
CYP1B1	cytochrome P450, family 1, subfamily B, polypeptide 1	Hs.154654	38.76	7.52
CYP2S1	cytochrome P450, family 2, subfamily S, polypeptide 1	Hs.98370	4.04	3.24
CYP4F11	cytochrome P450, family 4, subfamily F, polypeptide 11	Hs.187393	6.14	6.43
CYP4F3	cytochrome P450, family 4, subfamily F, polypeptide 3	Hs.106242	8.10	7.40
CYTH1	cytohesin 1	Hs.191215	3.54	4.19
CYTH2	cytohesin 2	Hs.144011	3.69	5.88
DAG1	dystroglycan 1 (dystrophin-associated glycoprotein 1)	Hs.76111	6.09	4.34
DAP	death-associated protein	Hs.75189	19.98	14.22
DARS2	aspartyl-tRNA synthetase 2, mitochondrial	Hs.647707	41.11	7.21
DAZAP2	DAZ associated protein 2	Hs.369761	8.77	3.53

DBNDD1	dysbindin (dystrobrevin binding protein 1) domain containing 1	Hs.301394	4.98	3.29
DBP	D site of albumin promoter (albumin D-box) binding protein	Hs.414480	3.36	2.78
DCAF12	DDB1 and CUL4 associated factor 12	Hs.493750	9.85	3.21
DCAF13	DDB1 and CUL4 associated factor 13	Hs.532265	41.09	17.30
DCAF15	DDB1 and CUL4 associated factor 15	Hs.443636	8.08	16.67
DCAF4	DDB1 and CUL4 associated factor 4	Hs.331491	4.79	3.22
DCAF8	DDB1 and CUL4 associated factor 8	Hs.632447	3.94	31.07
DCAKD	dephospho-CoA kinase domain containing	Hs.463148	5.01	11.84
DCBLD1	discoidin, CUB and LCCL domain containing 1	Hs.658304	3.36	7.51
DCBLD2	discoidin, CUB and LCCL domain containing 2	Hs.203691	12.68	8.00
DCTD	dCMP deaminase	Hs.183850	55.66	37.43
DCTN4	dynactin 4 (p62)	Hs.675564	52.43	5.25
DCTN6	dynactin 6	Hs.158427	32.19	7.55
DDA1	DET1 and DDB1 associated 1	Hs.466154	6.69	31.49
DDIT3	DNA-damage-inducible transcript 3	Hs.505777	20.54	27.31
DDIT4	DNA-damage-inducible transcript 4	Hs.523012	15.20	4.78
DDX1	DEAD (Asp-Glu-Ala-Asp) box polypeptide 1	Hs.440599	70.74	3.34
DDX11	DEAD/H (Asp-Glu-Ala-Asp/His) box polypeptide 11	Hs.443960	4.59	2.51
DDX17	DEAD (Asp-Glu-Ala-Asp) box polypeptide 17	Hs.528305	5.04	35.84
DDX19B	DEAD (Asp-Glu-Ala-As) box polypeptide 19B	Hs.221761	6.74	5.65
DDX20	DEAD (Asp-Glu-Ala-Asp) box polypeptide 20	Hs.591405	31.76	2.84
DDX31	DEAD (Asp-Glu-Ala-Asp) box polypeptide 31	Hs.660767	4.96	7.04
DDX39B	DEAD (Asp-Glu-Ala-Asp) box polypeptide 39B	Hs.254042	11.99	35.97
DDX5	DEAD (Asp-Glu-Ala-Asp) box polypeptide 5	Hs.279806	27.03	6.95
DDX55	DEAD (Asp-Glu-Ala-Asp) box polypeptide 55	Hs.286173	13.52	6.96
DDX6	DEAD (Asp-Glu-Ala-Asp) box polypeptide 6	Hs.408461	15.19	14.94
DECR1	2,4-dienoyl CoA reductase 1, mitochondrial	Hs.492212	27.46	26.24
DEDD	death effector domain containing	Hs.146406	3.92	15.67
DENND2C	DENN/MADD domain containing 2C	Hs.654928	10.45	5.09
DENND5A	DENN/MADD domain containing 5A	Hs.501857	8.82	2.65
DERL1	Der1-like domain family, member 1	Hs.241576	20.43	6.80
DGCR5	DiGeorge syndrome critical region gene 5 (non-protein coding)	Hs.646438	5.26	6.43
DGUOK	deoxyguanosine kinase	Hs.469022	10.60	7.24
DHDDS	dehydrodolichyl diphosphate synthase	Hs.369385	5.25	12.37
DHFR	dihydrofolate reductase	Hs.648635	24.42	5.96
DHPS	deoxyhypusine synthase	Hs.79064	3.23	3.22
DHRS1	dehydrogenase/reductase (SDR family) member 1	Hs.348350	6.51	4.96
DHRS2	dehydrogenase/reductase (SDR family) member 2	Hs.272499	5.59	2.63
DHRS4	dehydrogenase/reductase (SDR family) member 4	Hs.692654	4.06	3.74
DHRS7	dehydrogenase/reductase (SDR family) member 7	Hs.59719	15.08	8.44
DHX33	DEAH (Asp-Glu-Ala-His) box polypeptide 33	Hs.250456	6.22	4.21
DHX35	DEAH (Asp-Glu-Ala-His) box polypeptide 35	Hs.444520	22.50	3.90
DHX38	DEAH (Asp-Glu-Ala-His) box polypeptide 38	Hs.151218	3.78	7.44
DHX57	DEAH (Asp-Glu-Ala-Asp/His) box polypeptide 57	Hs.468226	4.67	9.16
DHX9	DEAH (Asp-Glu-Ala-His) box polypeptide 9	Hs.191518	14.08	52.07
DIRC2	disrupted in renal carcinoma 2	Hs.477346	18.17	3.36
DKC1	dyskeratosis congenita 1, dyskerin	Hs.4747	22.23	2.77

DKK1	dickkopf 1 homolog (Xenopus laevis)	Hs.40499	50.13	8.26
DKK3	dickkopf 3 homolog (Xenopus laevis)	Hs.292156	6.52	7.01
DLEU1	deleted in lymphocytic leukemia 1 (non-protein coding)	Hs.591229	36.97	4.17
DLG1	discs, large homolog 1 (Drosophila)	Hs.292549	12.66	13.50
DLGAP5	discs, large (Drosophila) homolog-associated protein 5	Hs.77695	83.48	13.55
DLX6-AS1	DLX6 antisense RNA 1 (non-protein coding)	Hs.34969	26.91	3.12
DNAAF2	dynein, axonemal, assembly factor 2	Hs.231761	14.02	8.86
DNAJA1	DnaJ (Hsp40) homolog, subfamily A, member 1	Hs.445203	28.44	8.10
DNAJB11	DnaJ (Hsp40) homolog, subfamily B, member 11	Hs.317192	13.22	5.94
DNAJB2	DnaJ (Hsp40) homolog, subfamily B, member 2	Hs.77768	5.36	6.07
DNAJB5	DnaJ (Hsp40) homolog, subfamily B, member 5	Hs.237506	5.41	3.69
DNAJB6	DnaJ (Hsp40) homolog, subfamily B, member 6	Hs.593923	40.78	13.05
DNAJC1	DnaJ (Hsp40) homolog, subfamily C, member 1	Hs.499000	6.19	2.80
DNAJC11	DnaJ (Hsp40) homolog, subfamily C, member 11	Hs.462640	6.78	3.38
DNAJC13	DnaJ (Hsp40) homolog, subfamily C, member 13	Hs.12707	13.64	5.23
DNAJC14	DnaJ (Hsp40) homolog, subfamily C, member 14	Hs.709320	3.54	16.41
DNAJC19	DnaJ (Hsp40) homolog, subfamily C, member 19	Hs.230601	38.68	12.47
DNAJC22	DnaJ (Hsp40) homolog, subfamily C, member 22	Hs.659300	5.57	5.59
DNAJC7	DnaJ (Hsp40) homolog, subfamily C, member 7	Hs.500156	31.20	18.10
DNASE1L1	deoxyribonuclease I-like 1	Hs.401929	13.13	3.83
DNASE2	deoxyribonuclease II, lysosomal	Hs.118243	7.74	4.40
DNM2	dynamin 2	Hs.211463	2.54	9.22
DNMBP	dynamin binding protein	Hs.500771	12.07	6.40
DNPEP	aspartyl aminopeptidase	Hs.258551	6.20	13.45
DOCK9	dedicator of cytokinesis 9	Hs.596105	6.22	13.83
DOHH	deoxyhypusine hydroxylase/monooxygenase	Hs.515064	4.59	4.78
DOK4	docking protein 4	Hs.279832	5.98	2.63
DOT1L	DOT1-like, histone H3 methyltransferase (S. cerevisiae)	Hs.713641	2.54	3.25
DPF1	D4, zinc and double PHD fingers family 1	Hs.631576	5.31	5.95
DPH3P1	DPH3, KTI11 homolog (S. cerevisiae) pseudogene 1	Hs.126248	9.04	4.11
DPH5	DPH5 homolog (S. cerevisiae)	Hs.440776	23.78	4.00
DPM1	dolichyl-phosphate mannosyltransferase polypeptide 1, catalytic subunit	Hs.654951	78.44	11.33
DPM2	dolichyl-phosphate mannosyltransferase polypeptide 2, regulatory subunit	Hs.108973	6.91	36.96
DPP9	dipeptidyl-peptidase 9	Hs.515081	6.03	7.37
DPY19L1	dpy-19-like 1 (C. elegans)	Hs.408623	70.91	51.11
DPY30	dpy-30 homolog (C. elegans)	Hs.531788	17.45	3.33
DRAM1	DNA-damage regulated autophagy modulator 1	Hs.730859	71.97	2.96
DRAM2	DNA-damage regulated autophagy modulator 2	Hs.485606	47.03	13.40
DSC2	desmocollin 2	Hs.95612	11.53	42.32
DSCR3	Down syndrome critical region gene 3	Hs.369488	8.04	6.21
DSG2	desmoglein 2	Hs.412597	26.53	80.78
DSG3	desmoglein 3	Hs.1925	33.74	2.51
DSN1	DSN1, MIND kinetochore complex component, homolog (S. cerevisiae)	Hs.632268	35.91	8.25
DST	dystonin	Hs.604915	12.12	4.93
DTL	denticleless homolog (Drosophila)	Hs.656473	36.70	3.08

DTX2	deltex homolog 2 (Drosophila)	Hs.187058	2.51	6.65
DUOX2	dual oxidase 2	Hs.71377	4.03	3.48
DUSP11	dual specificity phosphatase 11 (RNA/RNP complex 1-interacting)	Hs.14611	11.18	13.03
DUSP22	dual specificity phosphatase 22	Hs.29106	6.96	5.71
DUSP3	dual specificity phosphatase 3	Hs.181046	15.69	5.45
DUSP9	dual specificity phosphatase 9	Hs.721355	3.36	10.45
DVL3	dishevelled, dsh homolog 3 (Drosophila)	Hs.388116	6.65	2.91
DYNC1I2	dynein, cytoplasmic 1, intermediate chain 2	Hs.546250	38.51	7.28
DYNC1LI2	dynein, cytoplasmic 1, light intermediate chain 2	Hs.369068	16.35	14.06
DYNLRB1	dynein, light chain, roadblock-type 1	Hs.593920	4.31	14.46
DYNLT1	dynein, light chain, Tctex-type 1	Hs.445999	5.35	14.95
DYNLT3	dynein, light chain, Tctex-type 3	Hs.446392	30.66	9.66
DZIP1	DAZ interacting protein 1	Hs.656580	23.66	8.73
E2F4	E2F transcription factor 4, p107/p130-binding	Hs.108371	10.02	19.25
E2F6	E2F transcription factor 6	Hs.603093	11.78	4.83
EARS2	glutamyl-tRNA synthetase 2, mitochondrial (putative)	Hs.620541	7.72	6.78
EBAG9	estrogen receptor binding site associated, antigen, 9	Hs.409368	68.72	21.36
EBI3	Epstein-Barr virus induced 3	Hs.501452	9.55	2.76
EBPL	emopamil binding protein-like	Hs.433278	3.04	3.52
ECE1	endothelin converting enzyme 1	Hs.195080	3.51	12.79
ECH1	enoyl CoA hydratase 1, peroxisomal	Hs.196176	3.47	9.73
EDEM1	ER degradation enhancer, mannosidase alpha-like 1	Hs.224616	5.16	12.36
EDEM2	ER degradation enhancer, mannosidase alpha-like 2	Hs.720177	4.26	3.50
EDN1	endothelin 1	Hs.511899	12.17	5.06
EEF1A1	eukaryotic translation elongation factor 1 alpha 1	Hs.535192	5.75	16.95
EEF1E1	eukaryotic translation elongation factor 1 epsilon 1	Hs.726163	40.63	14.58
EEF2	eukaryotic translation elongation factor 2	Hs.515070	2.57	5.32
EEFSEC	eukaryotic elongation factor, selenocysteine-tRNA-specific	Hs.477498	2.52	5.49
EFNA1	ephrin-A1	Hs.516664	21.78	10.90
EFNA3	ephrin-A3	Hs.516656	8.85	41.20
EFNB2	ephrin-B2	Hs.149239	22.60	3.28
EGFR	epidermal growth factor receptor	Hs.488293	5.75	10.27
EHMT1	euchromatic histone-lysine N-methyltransferase 1	Hs.495511	2.54	4.14
EI24	etoposide induced 2.4 mRNA	Hs.730638	21.45	6.55
EID2	EP300 interacting inhibitor of differentiation 2	Hs.18949	22.00	4.57
EIF1B	eukaryotic translation initiation factor 1B	Hs.315230	13.74	2.64
EIF2A	eukaryotic translation initiation factor 2A, 65 kDa	Hs.655782	31.02	54.40
EIF2AK1	eukaryotic translation initiation factor 2-alpha kinase 1	Hs.728827	27.50	6.67
EIF2AK2	eukaryotic translation initiation factor 2-alpha kinase 2	Hs.131431	27.38	23.17
EIF2B1	eukaryotic translation initiation factor 2B, subunit 1 alpha, 26 kDa	Hs.728874	14.98	2.98
EIF2B5	eukaryotic translation initiation factor 2B, subunit 5 epsilon, 82 kDa	Hs.283551	4.10	3.69
EIF2D	eukaryotic translation initiation factor 2D	Hs.497581	25.88	8.52
EIF3A	eukaryotic translation initiation factor 3, subunit A	Hs.688653	9.96	16.54
EIF3F	eukaryotic translation initiation factor 3, subunit F	Hs.516023	3.32	3.47
EIF4A1	eukaryotic translation initiation factor 4A1	Hs.129673	5.38	3.70
EIF4A2	eukaryotic translation initiation factor 4A2	Hs.518475	17.97	5.26
EIF4B	eukaryotic translation initiation factor 4B	Hs.648394	12.98	17.01

EIF4E	eukaryotic translation initiation factor 4E	Hs.249718	34.99	22.08
EIF5A	eukaryotic translation initiation factor 5A	Hs.534314	2.53	19.88
ELAVL1	ELAV (embryonic lethal, abnormal vision, Drosophila)-like 1 (Hu antigen R)	Hs.184492	6.83	7.09
ELF1	E74-like factor 1 (ets domain transcription factor)	Hs.135646	13.45	20.21
ELF3	E74-like factor 3 (ets domain transcription factor, epithelial-specific)	Hs.730618	6.62	4.21
ELK4	ELK4, ETS-domain protein (SRF accessory protein 1)	Hs.497520	4.37	8.30
ELL	elongation factor RNA polymerase II	Hs.515260	3.84	8.44
ELL3	elongation factor RNA polymerase II-like 3	Hs.706346	19.93	9.54
ELMO2	engulfment and cell motility 2	Hs.210469	5.31	6.22
ELOF1	elongation factor 1 homolog (S. cerevisiae)	Hs.631633	3.51	2.50
ELOVL1	ELOVL fatty acid elongase 1	Hs.25597	4.66	3.66
ELOVL5	ELOVL fatty acid elongase 5	Hs.725124	11.63	17.60
ELP4	elongation protein 4 homolog (S. cerevisiae)	Hs.175534	49.43	4.02
EMB	embigin	Hs.561411	40.01	10.85
EME1	essential meiotic endonuclease 1 homolog 1 (S. pombe)	Hs.514330	6.48	8.46
EML2	echinoderm microtubule associated protein like 2	Hs.24178	6.35	8.51
EN1	engrailed homeobox 1	Hs.271977	5.54	5.43
ENDOD1	endonuclease domain containing 1	Hs.167115	42.78	3.64
ENDOV	endonuclease V	Hs.728933	3.18	8.69
ENOPH1	enolase-phosphatase 1	Hs.18442	62.01	10.11
ENOSF1	enolase superfamily member 1	Hs.369762	10.62	2.96
ENTPD4	ectonucleoside triphosphate diphosphohydrolase 4	Hs.444389	5.48	4.72
ENY2	enhancer of yellow 2 homolog (Drosophila)	Hs.492555	28.91	2.70
EPB41L1	erythrocyte membrane protein band 4.1-like 1	Hs.437422	3.70	3.37
EPCAM	epithelial cell adhesion molecule	Hs.542050	33.88	3.02
EPHA2	EPH receptor A2	Hs.171596	9.91	59.56
EPN2	epsin 2	Hs.730624	8.15	4.39
EPOR	erythropoietin receptor	Hs.631624	10.40	7.91
EPRS	glutamyl-prolyl-tRNA synthetase	Hs.497788	30.69	5.30
EPT1	ethanolaminephosphotransferase 1 (CDP-ethanolamine-specific)	Hs.189073	24.42	20.09
ERF	Ets2 repressor factor	Hs.655969	4.05	4.74
ERGIC1	endoplasmic reticulum-golgi intermediate compartment (ERGIC) 1	Hs.509163	3.42	2.62
ERGIC2	ERGIC and golgi 2	Hs.339453	201.23	38.68
ERGIC3	ERGIC and golgi 3	Hs.472558	3.47	3.11
ERI3	ERI1 exoribonuclease family member 3	Hs.132497	5.94	4.43
ERLIN1	ER lipid raft associated 1	Hs.150087	8.08	91.64
ERMP1	endoplasmic reticulum metallopeptidase 1	Hs.591078	18.84	35.55
ERO1L	ERO1-like (S. cerevisiae)	Hs.592304	43.63	29.66
ERP29	endoplasmic reticulum protein 29	Hs.75841	4.40	8.48
ESD	esterase D	Hs.432491	23.50	18.71
ESPN	espin	Hs.147953	3.11	4.24
ESRP1	epithelial splicing regulatory protein 1	Hs.487471	15.12	15.13
ESYT2	extended synaptotagmin-like protein 2	Hs.490795	33.11	9.69
ETFA	electron-transfer-flavoprotein, alpha polypeptide	Hs.39925	11.31	26.47
ETFDH	electron-transferring-flavoprotein dehydrogenase	Hs.155729	16.65	2.51
ETV4	ets variant 4	Hs.434059	3.58	6.41
ETV6	ets variant 6	Hs.504765	3.18	5.34

EWSR1	Ewing sarcoma breakpoint region 1	Hs.374477	8.77	18.96
EXD2	exonuclease 3′-5′ domain containing 2	Hs.533878	5.76	18.62
EXOC7	exocyst complex component 7	Hs.514496	2.93	2.58
EXOSC10	exosome component 10	Hs.632368	11.89	5.58
EXOSC2	exosome component 2	Hs.654643	6.17	3.96
EXOSC6	exosome component 6	Hs.660633	3.77	8.24
EXOSC7	exosome component 7	Hs.719958	18.75	7.37
EXOSC9	exosome component 9	Hs.91728	17.52	4.74
EXT1	exostosin 1	Hs.492618	9.52	24.27
EYA3	eyes absent homolog 3 (Drosophila)	Hs.185774	6.89	8.79
F3	coagulation factor III (thromboplastin, tissue factor)	Hs.62192	38.61	4.87
F8A2	coagulation factor VIII-associated 2	Hs.593687	3.97	2.83
FAAH	fatty acid amide hydrolase	Hs.720143	2.82	2.87
FABP5	fatty acid binding protein 5 (psoriasis-associated)	Hs.408061	13.78	11.97
FADS3	fatty acid desaturase 3	Hs.21765	4.15	4.40
FAF2	Fas associated factor family member 2	Hs.484242	44.31	7.47
FAIM	Fas apoptotic inhibitory molecule	Hs.173438	76.66	7.40
FAM103A1	family with sequence similarity 103, member A1	Hs.727661	21.88	4.59
FAM115A	family with sequence similarity 115, member A	Hs.406492	8.48	26.46
FAM118A	family with sequence similarity 118, member A	Hs.265018	2.52	2.64
FAM120A	family with sequence similarity 120A	Hs.372003	6.98	11.59
FAM120AOS	family with sequence similarity 120A opposite strand	Hs.350364	7.34	4.20
FAM127B	family with sequence similarity 127, member B	Hs.460924	8.08	13.50
FAM131A	family with sequence similarity 131, member A	Hs.591307	17.26	16.63
FAM132A	family with sequence similarity 132, member A	Hs.197613	2.56	4.34
FAM132B	family with sequence similarity 132, member B	Hs.24951	4.56	5.99
FAM134C	family with sequence similarity 134, member C	Hs.632262	3.63	3.40
FAM160A1	family with sequence similarity 160, member A1	Hs.633810	3.01	12.43
FAM168B	family with sequence similarity 168, member B	Hs.534679	24.48	42.69
FAM171A1	family with sequence similarity 171, member A1	Hs.66762	4.18	4.45
FAM177A1	family with sequence similarity 177, member A1	Hs.446357	10.69	4.74
FAM185A	family with sequence similarity 185, member A	Hs.202543	13.75	5.52
FAM201B	family with sequence similarity 201, member B	Hs.98178	16.51	10.37
FAM20B	family with sequence similarity 20, member B	Hs.730715	30.84	3.15
FAM24B	family with sequence similarity 24, member B	Hs.114648	13.38	12.32
FAM32A	family with sequence similarity 32, member A	Hs.631614	7.78	2.85
FAM3A	family with sequence similarity 3, member A	Hs.289108	2.99	9.37
FAM3C	family with sequence similarity 3, member C	Hs.434053	191.76	34.20
FAM45A	family with sequence similarity 45, member A	Hs.730780	33.47	14.14
FAM50A	family with sequence similarity 50, member A	Hs.54277	3.43	3.18
FAM64A	family with sequence similarity 64, member A	Hs.592116	12.45	11.49
FAM69A	family with sequence similarity 69, member A	Hs.180946	14.46	6.92
FAM72A	family with sequence similarity 72, member A	Hs.661924	24.85	3.31
FAM86A	family with sequence similarity 86, member A	Hs.406461	3.94	6.26
FAM86C1	family with sequence similarity 86, member C1	Hs.591652	2.58	2.59
FAM86C2P	family with sequence similarity 86, member A pseudogene	Hs.535094	2.97	13.74
FAM96A	family with sequence similarity 96, member A	Hs.439548	56.05	6.32

FANCC	Fanconi anemia, complementation group C	Hs.494529	9.51	7.37
FANCI	Fanconi anemia, complementation group I	Hs.513126	14.65	4.28
FANCL	Fanconi anemia, complementation group L	Hs.631890	48.83	13.23
FARP1	FERM, RhoGEF (ARHGEF) and pleckstrin domain protein 1 (chondrocyte-derived)	Hs.403917	6.51	11.94
FARS2	phenylalanyl-tRNA synthetase 2, mitochondrial	Hs.484547	5.08	3.48
FARSB	phenylalanyl-tRNA synthetase, beta subunit	Hs.471452	18.38	7.20
FAS	Fas (TNF receptor superfamily, member 6)	Hs.244139	42.65	10.43
FASTKD2	FAST kinase domains 2	Hs.5930	41.79	4.65
FBLIM1	filamin binding LIM protein 1	Hs.530101	8.37	8.43
FBN2	fibrillin 2	Hs.519294	28.42	11.34
FBXL12	F-box and leucine-rich repeat protein 12	Hs.12439	9.21	14.15
FBXL2	F-box and leucine-rich repeat protein 2	Hs.475872	8.97	6.41
FBXL4	F-box and leucine-rich repeat protein 4	Hs.728297	35.60	4.28
FBXL5	F-box and leucine-rich repeat protein 5	Hs.643433	30.59	5.04
FBXO17	F-box protein 17	Hs.531770	4.09	3.64
FBXO22	F-box protein 22	Hs.591115	6.81	6.69
FBXO25	F-box protein 25	Hs.438454	8.53	6.93
FBXO34	F-box protein 34	Hs.525348	27.91	4.08
FBXO6	F-box protein 6	Hs.464419	6.42	5.13
FBXO8	F-box protein 8	Hs.76917	31.50	10.08
FBXO9	F-box protein 9	Hs.216653	11.57	2.80
FCF1	FCF1 small subunit (SSU) processome component homolog (S. cerevisiae)	Hs.579828	62.05	12.86
FEM1A	fem-1 homolog a (C. elegans)	Hs.515082	3.15	7.19
FEN1	flap structure-specific endonuclease 1	Hs.409065	15.83	16.43
FEZ2	fasciculation and elongation protein zeta 2 (zygin II)	Hs.258563	15.60	9.98
FGF11	fibroblast growth factor 11	Hs.655193	6.66	4.40
FGFR3	fibroblast growth factor receptor 3	Hs.1420	5.29	2.55
FGFRL1	fibroblast growth factor receptor-like 1	Hs.193326	5.98	3.20
FH	fumarate hydratase	Hs.592490	18.53	2.66
FHL2	four and a half LIM domains 2	Hs.443687	3.51	5.27
FHL3	four and a half LIM domains 3	Hs.57687	5.23	7.17
FIBCD1	fibrinogen C domain containing 1	Hs.133205	3.24	3.10
FIP1L1	FIP1 like 1 (S. cerevisiae)	Hs.624245	15.11	2.79
FIZ1	FLT3-interacting zinc finger 1	Hs.515617	5.50	3.22
FKBP1A	FK506 binding protein 1A, 12 kDa	Hs.471933	10.70	27.02
FKBP3	FK506 binding protein 3, 25 kDa	Hs.509226	27.88	8.60
FKBP4	FK506 binding protein 4, 59 kDa	Hs.524183	3.47	5.43
FKBP8	FK506 binding protein 8, 38 kDa	Hs.173464	2.84	2.58
FKBP9	FK506 binding protein 9, 63 kDa	Hs.103934	8.54	20.26
FLOT2	flotillin 2	Hs.514038	3.13	10.21
FLT3LG	fms-related tyrosine kinase 3 ligand	Hs.428	4.82	6.72
FLVCR1-AS1	FLVCR1 antisense RNA 1 (non-protein coding)	Hs.552649	7.44	4.12
FNBP1	formin binding protein 1	Hs.189409	13.68	4.41
FNTA	farnesyltransferase, CAAX box, alpha	Hs.370312	26.41	2.63
FOXF2	forkhead box F2	Hs.484423	6.27	6.32
FOXJ3	forkhead box J3	Hs.26023	21.18	3.46

FOXL2	forkhead box L2	Hs.289292	3.17	5.03
FOXN2	forkhead box N2	Hs.468478	53.33	10.20
FOXO3	forkhead box O3	Hs.220950	15.59	3.12
FOXRED1	FAD-dependent oxidoreductase domain containing 1	Hs.317190	2.86	3.83
FRMD8	FERM domain containing 8	Hs.578433	7.16	5.08
FRRS1	ferric-chelate reductase 1	Hs.454779	4.50	2.79
FSTL1	follistatin-like 1	Hs.269512	43.84	4.29
FTO	fat mass and obesity associated	Hs.528833	16.03	8.08
FTSJ2	FtsJ homolog 2 (E. coli)	Hs.279877	5.75	10.84
FUBP1	far upstream element (FUSE) binding protein 1	Hs.567380	21.42	8.64
FUBP3	far upstream element (FUSE) binding protein 3	Hs.98751	9.94	37.16
FUCA2	fucosidase, alpha-L- 2, plasma	Hs.17680	9.85	6.46
FUNDC1	FUN14 domain containing 1	Hs.7549	4.70	2.83
FURIN	furin (paired basic amino acid cleaving enzyme)	Hs.513153	3.44	16.94
FUS	fused in sarcoma	Hs.46894	7.11	16.38
FUT10	fucosyltransferase 10 (alpha (1, 3) fucosyltransferase)	Hs.458713	4.09	8.42
FUT11	fucosyltransferase 11 (alpha (1, 3) fucosyltransferase)	Hs.588854	6.01	10.88
FUT8	fucosyltransferase 8 (alpha (1, 6) fucosyltransferase)	Hs.654961	26.26	3.66
FXN	frataxin	Hs.20685	8.09	4.12
FXR1	fragile X mental retardation, autosomal homolog 1	Hs.478407	111.87	25.07
FXR2	fragile X mental retardation, autosomal homolog 2	Hs.52788	3.93	19.73
FXYD3	FXYD domain containing ion transport regulator 3	Hs.301350	5.17	24.14
FYTTD1	forty-two-three domain containing 1	Hs.277533	73.45	20.07
FZD6	frizzled family receptor 6	Hs.591863	123.77	18.07
G3BP2	GTPase activating protein (SH3 domain) binding protein 2	Hs.303676	45.24	3.33
G6PC3	glucose 6 phosphatase, catalytic, 3	Hs.294005	3.77	11.99
GABARAPL1	GABA(A) receptor-associated protein like 1	Hs.524250	20.24	6.16
GABARAPL2	GABA(A) receptor-associated protein-like 2	Hs.461379	17.88	7.28
GABPB1	GA binding protein transcription factor, beta subunit 1	Hs.654350	15.27	7.71
GADD45A	growth arrest and DNA-damage-inducible, alpha	Hs.80409	22.99	4.87
GAGE7	G antigen 7	Hs.460641	4.35	8.13
GAK	cyclin G associated kinase	Hs.369607	3.17	6.22
GALC	galactosylceramidase	Hs.513439	46.78	8.77
GALE	UDP-galactose-4-epimerase	Hs.632380	4.45	3.39
GALNS	galactosamine (N-acetyl)-6-sulfate sulfatase	Hs.271383	3.94	6.10
GALNT1	UDP-N-acetyl-alpha-D-galactosamine:polypeptide N-acetylgalactosaminyltransferase 1 (GalNAc-T1)	Hs.514806	90.63	31.99
GALNT12	UDP-N-acetyl-alpha-D-galactosamine:polypeptide N-acetylgalactosaminyltransferase 12 (GalNAc-T12)	Hs.47099	3.65	5.48
GALNT2	UDP-N-acetyl-alpha-D-galactosamine:polypeptide N-acetylgalactosaminyltransferase 2 (GalNAc-T2)	Hs.654649	12.81	22.90
GARS	glycyl-tRNA synthetase	Hs.404321	10.40	7.32
GAS1	growth arrest-specific 1	Hs.65029	3.26	4.02
GATC	glutamyl-tRNA(Gln) amidotransferase, subunit C homolog (bacterial)	Hs.728777	3.34	6.84
GBAS	glioblastoma amplified sequence	Hs.591069	62.01	7.46
GCH1	GTP cyclohydrolase 1	Hs.86724	21.96	13.40
GDA	guanine deaminase	Hs.494163	32.74	9.51
GDI2	GDP dissociation inhibitor 2	Hs.299055	19.54	33.22

GEM	GTP binding protein overexpressed in skeletal muscle	Hs.654463	15.77	4.39
GFM2	G elongation factor, mitochondrial 2	Hs.277154	43.02	11.81
GFOD1	glucose-fructose oxidoreductase domain containing 1	Hs.484686	3.62	4.91
GGCT	gamma-glutamylcyclotransferase	Hs.530024	17.31	51.67
GGT7	gamma-glutamyltransferase 7	Hs.433738	4.68	5.72
GIGYF2	GRB10 interacting GYF protein 2	Hs.565319	4.22	13.51
GJA1	gap junction protein, alpha 1, 43 kDa	Hs.74471	35.82	6.52
GJB3	gap junction protein, beta 3, 31 kDa	Hs.522561	7.20	6.72
GJB5	gap junction protein, beta 5, 31.1 kDa	Hs.198249	2.90	2.59
GJB6	gap junction protein, beta 6, 30 kDa	Hs.511757	36.64	29.95
GLB1L	galactosidase, beta 1-like	Hs.181173	9.46	4.10
GLE1	GLE1 RNA export mediator homolog (yeast)	Hs.522418	4.75	8.61
GLIPR2	GLI pathogenesis-related 2	Hs.493819	4.23	4.45
GLO1	glyoxalase I	Hs.268849	46.86	16.72
GLOD4	glyoxalase domain containing 4	Hs.279061	18.23	2.52
GLRX	glutaredoxin (thioltransferase)	Hs.28988	33.21	3.50
GLRX3	glutaredoxin 3	Hs.42644	10.97	2.74
GLT8D1	glycosyltransferase 8 domain containing 1	Hs.297304	33.45	4.56
GLTP	glycolipid transfer protein	Hs.381256	8.95	43.37
GLTPD2	glycolipid transfer protein domain containing 2	Hs.721461	2.97	3.04
GLUL	glutamate-ammonia ligase	Hs.518525	7.50	5.29
GMEB2	glucocorticoid modulatory element binding protein 2	Hs.473286	2.62	3.54
GMPPA	GDP-mannose pyrophosphorylase A	Hs.27059	2.82	10.49
GNA11	guanine nucleotide binding protein (G protein), alpha 11 (Gq class)	Hs.650575	5.23	6.17
GNA12	guanine nucleotide binding protein (G protein) alpha 12	Hs.487341	5.71	3.06
GNA15	guanine nucleotide binding protein (G protein), alpha 15 (Gq class)	Hs.73797	3.02	4.04
GNB2	guanine nucleotide binding protein (G protein), beta polypeptide 2	Hs.185172	3.26	4.25
GNB5	guanine nucleotide binding protein (G protein), beta 5	Hs.155090	8.83	7.61
GNG10	guanine nucleotide binding protein (G protein), gamma 10	Hs.534196	20.78	5.02
GNG4	guanine nucleotide binding protein (G protein), gamma 4	Hs.159711	4.66	7.43
GNG5	guanine nucleotide binding protein (G protein), gamma 5	Hs.645427	10.63	5.00
GNL3	guanine nucleotide binding protein-like 3 (nucleolar)	Hs.313544	69.42	8.95
GNPAT	glyceronephosphate O-acyltransferase	Hs.498028	16.43	3.72
GNPNAT1	glucosamine-phosphate N-acetyltransferase 1	Hs.702056	27.09	3.59
GNPTAB	N-acetylglucosamine-1-phosphate transferase, alpha and beta subunits	Hs.46850	10.17	4.89
GOLGA7	golgin A7	Hs.654773	32.07	20.70
GOLPH3	golgi phosphoprotein 3 (coat-protein)	Hs.408909	26.48	6.06
GOSR2	golgi SNAP receptor complex member 2	Hs.463278	7.26	4.99
GPATCH3	G patch domain containing 3	Hs.10903	3.22	4.12
GPATCH4	G patch domain containing 4	Hs.193832	9.88	22.15
GPBP1L1	GC-rich promoter binding protein 1-like 1	Hs.238432	24.02	6.30
GPC3	glypican 3	Hs.644108	8.54	3.36
GPC4	glypican 4	Hs.58367	14.65	2.90
GPD1L	glycerol-3-phosphate dehydrogenase 1-like	Hs.82432	31.40	3.72
GPD2	glycerol-3-phosphate dehydrogenase 2 (mitochondrial)	Hs.512382	56.90	11.85
GPN1	GPN-loop GTPase 1	Hs.18259	15.65	2.75
GPNMB	glycoprotein (transmembrane) nmb	Hs.190495	6.15	11.93

GPR108	G protein-coupled receptor 108	Hs.167641	3.61	3.37
GPR125	G protein-coupled receptor 125	Hs.99195	6.84	2.65
GPR137B	G protein-coupled receptor 137B	Hs.498160	15.07	5.69
GPR160	G protein-coupled receptor 160	Hs.231320	60.24	3.59
GPR87	G protein-coupled receptor 87	Hs.591292	46.05	16.14
GPRASP2	G protein-coupled receptor associated sorting protein 2	Hs.522729	4.87	4.93
GPRC5A	G protein-coupled receptor, family C, group 5, member A	Hs.631733	5.26	4.04
GRB2	growth factor receptor-bound protein 2	Hs.444356	12.65	8.43
GRB7	growth factor receptor-bound protein 7	Hs.86859	3.78	4.45
GRHL2	grainyhead-like 2 (Drosophila)	Hs.661088	5.70	16.12
GRHPR	glyoxylate reductase/hydroxypyruvate reductase	Hs.155742	5.52	4.51
GRINA	glutamate receptor, ionotropic, N-methyl D-aspartate-associated protein 1 (glutamate binding)	Hs.594634	2.97	3.80
GRPEL1	GrpE-like 1, mitochondrial (E. coli)	Hs.443723	9.33	2.88
GRTP1	growth hormone regulated TBC protein 1	Hs.170904	18.38	11.67
GSG2	germ cell associated 2 (haspin)	Hs.534059	3.56	7.96
GSK3B	glycogen synthase kinase 3 beta	Hs.445733	12.89	4.72
GSPT1	G1 to S phase transition 1	Hs.528780	12.53	22.93
GSR	glutathione reductase	Hs.271510	4.21	5.29
GSTA4	glutathione S-transferase alpha 4	Hs.485557	28.88	6.09
GSTO2	glutathione S-transferase omega 2	Hs.203634	4.78	3.37
GTF2A1	general transcription factor IIA, 1, 19/37 kDa	Hs.592334	11.17	30.21
GTF2A2	general transcription factor IIA, 2, 12 kDa	Hs.512934	35.27	2.68
GTF2B	general transcription factor IIB	Hs.481852	32.54	5.85
GTF2E1	general transcription factor IIE, polypeptide 1, alpha 56 kDa	Hs.445272	41.39	3.92
GTF2F2	general transcription factor IIF, polypeptide 2, 30 kDa	Hs.654582	90.57	7.71
GTF2H3	general transcription factor IIH, polypeptide 3, 34 kDa	Hs.355348	16.43	7.42
GTF2H4	general transcription factor IIH, polypeptide 4, 52 kDa	Hs.485070	8.65	6.35
GTF2IRD1	GTF2I repeat domain containing 1	Hs.647056	4.49	2.63
GTPBP2	GTP binding protein 2	Hs.485449	8.51	11.10
GTPBP4	GTP binding protein 4	Hs.215766	37.13	2.75
GTSE1	G-2 and S-phase expressed 1	Hs.386189	5.60	2.65
GYG1	glycogenin 1	Hs.477892	3.20	3.46
GYLTL1B	glycosyltransferase-like 1B	Hs.86543	8.06	7.72
H1F0	H1 histone family, member 0	Hs.226117	15.46	3.07
H1FX	H1 histone family, member X	Hs.75307	6.01	27.67
H2AFV	H2A histone family, member V	Hs.488189	4.31	2.57
H2AFY	H2A histone family, member Y	Hs.420272	5.11	18.51
HABP4	hyaluronan binding protein 4	Hs.494567	13.21	4.93
HADHA	hydroxyacyl-CoA dehydrogenase/3-ketoacyl-CoA thiolase/enoyl-CoA hydratase (trifunctional protein), alpha subunit	Hs.516032	5.69	6.72
HADHB	hydroxyacyl-CoA dehydrogenase/3-ketoacyl-CoA thiolase/enoyl-CoA hydratase (trifunctional protein), beta subunit	Hs.515848	10.49	2.51
HARS2	histidyl-tRNA synthetase 2, mitochondrial (putative)	Hs.432560	13.99	3.20
HAS3	hyaluronan synthase 3	Hs.592069	3.40	3.24
HAUS1	HAUS augmin-like complex, subunit 1	Hs.436617	51.51	13.25
HAUS2	HAUS augmin-like complex, subunit 2	Hs.14347	33.44	7.06
HAUS4	HAUS augmin-like complex, subunit 4	Hs.442782	3.35	2.71

HAUS5	HAUS augmin-like complex, subunit 5	Hs.7426	3.75	2.99
HAUS8	HAUS augmin-like complex, subunit 8	Hs.404088	7.70	2.93
HCCS	holocytochrome c synthase	Hs.211571	13.12	3.96
HCG18	HLA complex group 18 (non-protein coding)	Hs.485041	8.56	374.72
HCG4	HLA complex group 4 (non-protein coding)	Hs.660431	17.13	49.90
HCG4B	HLA complex group 4B (non-protein coding)	Hs.181244	5.55	2653.00
HDAC6	histone deacetylase 6	Hs.6764	2.57	3.02
HDAC8	histone deacetylase 8	Hs.310536	11.20	18.16
HDGF	hepatoma-derived growth factor	Hs.506748	3.76	2.89
HDHD3	haloacid dehalogenase-like hydrolase domain containing 3	Hs.7739	2.80	5.12
HDLBP	high density lipoprotein binding protein	Hs.471851	3.09	3.75
HEATR3	HEAT repeat containing 3	Hs.313917	3.52	5.70
HECTD1	HECT domain containing 1	Hs.708017	23.86	2.60
HECTD3	HECT domain containing 3	Hs.525084	4.13	2.97
HEG1	HEG homolog 1 (zebrafish)	Hs.477420	10.95	2.95
HERPUD2	HERPUD family member 2	Hs.729113	4.31	6.24
HEXA	hexosaminidase A (alpha polypeptide)	Hs.604479	6.13	5.29
HEXB	hexosaminidase B (beta polypeptide)	Hs.69293	30.30	12.81
HGSNAT	heparan-alpha-glucosaminide N-acetyltransferase	Hs.600384	3.94	5.64
HHEX	hematopoietically expressed homeobox	Hs.118651	9.78	5.33
HHLA3	HERV-H LTR-associating 3	Hs.142245	7.09	4.57
HIATL1	hippocampus abundant transcript-like 1	Hs.727498	11.52	14.22
HIBADH	3-hydroxyisobutyrate dehydrogenase	Hs.406758	38.85	6.59
HIBCH	3-hydroxyisobutyryl-CoA hydrolase	Hs.656685	11.04	9.55
HIF1AN	hypoxia inducible factor 1, alpha subunit inhibitor	Hs.500788	6.08	9.30
HIGD2A	HIG1 hypoxia inducible domain family, member 2A	Hs.534575	7.27	2.57
HINFP	histone H4 transcription factor	Hs.504091	4.36	2.70
HIPK1	homeodomain interacting protein kinase 1	Hs.532363	11.88	7.64
HIRIP3	HIRA interacting protein 3	Hs.592046	11.23	2.90
HIST2H2BE	histone cluster 2, H2be	Hs.2178	4.26	21.03
HLA-B	major histocompatibility complex, class I, B	Hs.77961	2.59	3.17
HLA-DOB	major histocompatibility complex, class II, DO beta	Hs.1802	10.02	5.78
HLA-F-AS1	HLA-F antisense RNA 1 (non-protein coding)	Hs.646985	3.14	8.93
HM13	histocompatibility (minor) 13	Hs.373741	2.96	20.37
HMGB1	high mobility group box 1	Hs.434102	11.34	23.06
HMGB2	high mobility group box 2	Hs.434953	52.55	6.68
HMGCL	3-hydroxymethyl-3-methylglutaryl-CoA lyase	Hs.533444	3.39	7.67
HMGN1	high mobility group nucleosome binding domain 1	Hs.356285	29.43	8.17
HMGN3	high mobility group nucleosomal binding domain 3	Hs.77558	34.72	36.43
HMGN4	high mobility group nucleosomal binding domain 4	Hs.236774	65.43	22.75
HN1L	hematological and neurological expressed 1-like	Hs.513261	6.37	4.47
HNRNPA1	heterogeneous nuclear ribonucleoprotein A1	Hs.546261	10.46	6.20
HNRNPA1L2	heterogeneous nuclear ribonucleoprotein A1-like 2	Hs.447506	10.84	5.44
HNRNPA3	heterogeneous nuclear ribonucleoprotein A3	Hs.516539	27.09	18.21
HNRNPAB	heterogeneous nuclear ribonucleoprotein A/B	Hs.591731	11.02	18.18
HNRNPD	heterogeneous nuclear ribonucleoprotein D (AU-rich element RNA binding protein 1, 37 kDa)	Hs.480073	20.12	5.00
HNRNPF	heterogeneous nuclear ribonucleoprotein F	Hs.808	11.19	16.53

HNRNPH3	heterogeneous nuclear ribonucleoprotein H3 (2H9)	Hs.643472	147.91	40.83
HNRNPK	heterogeneous nuclear ribonucleoprotein K	Hs.522257	10.66	54.31
HNRNPL	heterogeneous nuclear ribonucleoprotein L	Hs.644906	3.36	3.72
HNRNPR	heterogeneous nuclear ribonucleoprotein R	Hs.373763	98.14	19.17
HNRNPU	heterogeneous nuclear ribonucleoprotein U (scaffold attachment factor A)	Hs.106212	20.21	21.70
HNRNPUL1	heterogeneous nuclear ribonucleoprotein U-like 1	Hs.155218	8.72	7.79
HOMER2	homer homolog 2 (Drosophila)	Hs.578443	7.15	4.36
HOMEZ	homeobox and leucine zipper encoding	Hs.632332	10.44	4.46
HOXA13	homeobox A13	Hs.592172	16.65	4.50
HOXB3	homeobox B3	Hs.654560	5.82	9.61
HOXB4	homeobox B4	Hs.664706	11.34	2.89
HOXC10	homeobox C10	Hs.44276	5.47	17.42
HOXC4	homeobox C4	Hs.549040	12.56	2.66
HOXC8	homeobox C8	Hs.664500	17.41	4.78
HOXD10	homeobox D10	Hs.123070	16.37	3.14
HPCAL1	hippocalcin-like 1	Hs.580427	6.19	6.43
HPSE	heparanase	Hs.44227	19.37	8.56
HRSP12	heat-responsive protein 12	Hs.18426	66.99	8.32
HS6ST2	heparan sulfate 6-O-sulfotransferase 2	Hs.385956	21.01	4.73
HSBP1	heat shock factor binding protein 1	Hs.250899	16.65	3.82
HSBP1L1	heat shock factor binding protein 1-like 1	Hs.191582	12.03	8.11
HSD11B1L	hydroxysteroid (11-beta) dehydrogenase 1-like	Hs.631840	5.44	2.82
HSD17B11	hydroxysteroid (17-beta) dehydrogenase 11	Hs.594923	33.32	5.72
HSD17B14	hydroxysteroid (17-beta) dehydrogenase 14	Hs.18788	2.53	3.65
HSD17B6	hydroxysteroid (17-beta) dehydrogenase 6 homolog (mouse)	Hs.524513	32.84	5.28
HSD17B7	hydroxysteroid (17-beta) dehydrogenase 7	Hs.492925	5.88	16.52
HSF2	heat shock transcription factor 2	Hs.158195	33.35	6.75
HSF4	heat shock transcription factor 4	Hs.512156	2.66	2.77
HSP90AA1	heat shock protein 90 kDa alpha (cytosolic), class A member 1	Hs.525600	44.25	13.53
HSP90AB1	heat shock protein 90 kDa alpha (cytosolic), class B member 1	Hs.509736	10.23	22.52
HSP90B1	heat shock protein 90 kDa beta (Grp94), member 1	Hs.192374	66.00	52.15
HSPA14	heat shock 70 kDa protein 14	Hs.534169	29.71	7.57
HSPA4	heat shock 70 kDa protein 4	Hs.90093	88.97	23.81
HSPA8	heat shock 70 kDa protein 8	Hs.180414	3.25	81.78
HSPA9	heat shock 70 kDa protein 9 (mortalin)	Hs.184233	19.76	4.74
HSPB11	heat shock protein family B (small), member 11	Hs.525462	5.27	52.89
HSPE1	heat shock 10 kDa protein 1 (chaperonin 10)	Hs.1197	28.67	5.70
HSPH1	heat shock 105 kDa/110 kDa protein 1	Hs.36927	74.91	9.86
HTATIP2	HIV-1 Tat interactive protein 2, 30 kDa	Hs.90753	31.80	28.70
HTR2C	5-hydroxytryptamine (serotonin) receptor 2C	Hs.149037	14.51	11.01
HTRA3	HtrA serine peptidase 3	Hs.479119	4.81	11.44
HYOU1	hypoxia up-regulated 1	Hs.277704	15.85	108.49
IARS	isoleucyl-tRNA synthetase	Hs.445403	6.94	5.30
IARS2	isoleucyl-tRNA synthetase 2, mitochondrial	Hs.262823	50.86	12.04
ID3	inhibitor of DNA binding 3, dominant negative helix-loop-helix protein	Hs.76884	12.41	11.92
IDH1	isocitrate dehydrogenase 1 (NADP+), soluble	Hs.593422	17.42	4.29
IDH3B	isocitrate dehydrogenase 3 (NAD+) beta	Hs.436405	4.80	5.75

IDI1	isopentenyl-diphosphate delta isomerase 1	Hs.283652	9.69	3.38
IFNGR1	interferon gamma receptor 1	Hs.520414	81.06	4.23
IFNGR2	interferon gamma receptor 2 (interferon gamma transducer 1)	Hs.634632	14.55	11.49
IFRD1	interferon-related developmental regulator 1	Hs.7879	18.84	6.84
IFT20	intraflagellar transport 20 homolog (Chlamydomonas)	Hs.705431	16.21	14.81
IFT27	intraflagellar transport 27 homolog (Chlamydomonas)	Hs.415172	10.25	7.52
IGF2BP2	insulin-like growth factor 2 mRNA binding protein 2	Hs.35354	8.43	18.40
IGFBP4	insulin-like growth factor binding protein 4	Hs.462998	11.64	29.42
IGSF8	immunoglobulin superfamily, member 8	Hs.332012	4.41	5.15
IK	IK cytokine, down-regulator of HLA II	Hs.421245	21.89	9.26
IL10RB	interleukin 10 receptor, beta	Hs.654593	18.08	7.17
IL15RA	interleukin 15 receptor, alpha	Hs.524117	10.63	15.43
IL17RD	interleukin 17 receptor D	Hs.150725	6.07	4.41
IL2RG	interleukin 2 receptor, gamma	Hs.84	6.62	7.96
IL32	interleukin 32	Hs.943	4.41	4.66
IL6R	interleukin 6 receptor	Hs.709210	3.87	14.51
ILF2	interleukin enhancer binding factor 2, 45 kDa	Hs.75117	3.22	20.88
ILF3	interleukin enhancer binding factor 3, 90 kDa	Hs.465885	4.01	2.90
IMMP1L	IMP1 inner mitochondrial membrane peptidase-like (S. cerevisiae)	Hs.502223	11.30	13.27
IMMT	inner membrane protein, mitochondrial	Hs.148559	5.27	23.19
IMPAD1	inositol monophosphatase domain containing 1	Hs.438689	59.74	11.11
INADL	InaD-like (Drosophila)	Hs.478125	3.93	5.66
ING1	inhibitor of growth family, member 1	Hs.46700	5.16	3.46
ING2	inhibitor of growth family, member 2	Hs.107153	39.76	4.01
INHBA	inhibin, beta A	Hs.583348	23.89	3.79
INO80B	INO80 complex subunit B	Hs.410786	3.78	16.41
INO80C	INO80 complex subunit C	Hs.464903	8.27	5.78
INPP5A	inositol polyphosphate-5-phosphatase, 40 kDa	Hs.523360	4.82	7.04
INPP5F	inositol polyphosphate-5-phosphatase F	Hs.369755	13.65	3.42
INSIG2	insulin induced gene 2	Hs.7089	82.94	23.13
INSR	insulin receptor	Hs.465744	9.05	3.40
INTS12	integrator complex subunit 12	Hs.480454	9.97	5.63
INTS5	integrator complex subunit 5	Hs.458390	2.71	10.10
INTS8	integrator complex subunit 8	Hs.727669	13.91	5.42
IPO5	importin 5	Hs.712598	45.01	18.51
IQCJ-SCHIP1	IQCJ-SCHIP1 readthrough	Hs.134665	32.57	19.37
IRF2BPL	interferon regulatory factor 2 binding protein-like	Hs.728364	3.00	16.11
IRF6	interferon regulatory factor 6	Hs.719361	10.87	15.87
IRF9	interferon regulatory factor 9	Hs.1706	10.45	3.69
IRS2	insulin receptor substrate 2	Hs.442344	8.40	4.36
IRX5	iroquois homeobox 5	Hs.435730	5.71	3.06
ISG20L2	interferon stimulated exonuclease gene 20 kDa-like 2	Hs.301904	6.83	2.73
ISOC1	isochorismatase domain containing 1	Hs.483296	19.88	3.31
ISY1	ISY1 splicing factor homolog (S. cerevisiae)	Hs.512661	8.63	8.26
ITFG1	integrin alpha FG-GAP repeat containing 1	Hs.42217	48.94	8.78
ITGA6	integrin, alpha 6	Hs.133397	29.30	11.16
ITGAE	integrin, alpha E (antigen CD103, human mucosal lymphocyte antigen 1; alpha polypeptide)	Hs.513867	32.09	7.78

ITGB1	integrin, beta 1 (fibronectin receptor, beta polypeptide, antigen CD29 includes MDF2, MSK12)	Hs.643813	16.76	92.82
ITM2B	integral membrane protein 2B	Hs.643683	20.73	3.77
ITSN2	intersectin 2	Hs.432562	4.02	10.00
JAG1	jagged 1	Hs.728907	6.65	5.49
JKAMP	JNK1/MAPK8-associated membrane protein	Hs.446850	22.11	3.44
JMJD6	jumonji domain containing 6	Hs.514505	11.65	17.79
JUNB	jun B proto-oncogene	Hs.25292	6.48	28.41
JUND	jun D proto-oncogene	Hs.2780	5.03	28.92
KANSL2	KAT8 regulatory NSL complex subunit 2	Hs.505412	10.82	3.31
KAT5	K(lysine) acetyltransferase 5	Hs.397010	3.17	2.99
KAT7	K(lysine) acetyltransferase 7	Hs.21907	13.67	14.84
KBTBD4	kelch repeat and BTB (POZ) domain containing 4	Hs.718483	19.00	13.04
KCNJ15	potassium inwardly-rectifying channel, subfamily J, member 15	Hs.411299	12.37	8.46
KCNK1	potassium channel, subfamily K, member 1	Hs.208544	4.52	23.25
KCNK6	potassium channel, subfamily K, member 6	Hs.240395	3.86	4.26
KCNN4	potassium intermediate/small conductance calcium-activated channel, subfamily N, member 4	Hs.10082	4.33	3.21
KCTD1	potassium channel tetramerisation domain containing 1	Hs.526630	18.30	2.64
KCTD2	potassium channel tetramerisation domain containing 2	Hs.514468	4.29	3.49
KCTD5	potassium channel tetramerisation domain containing 5	Hs.61960	31.30	26.04
KCTD9	potassium channel tetramerisation domain containing 9	Hs.72071	34.78	23.59
KDELC2	KDEL (Lys-Asp-Glu-Leu) containing 2	Hs.83286	17.41	11.62
KDELR1	KDEL (Lys-Asp-Glu-Leu) endoplasmic reticulum protein retention receptor 1	Hs.515515	5.82	5.44
KDELR3	KDEL (Lys-Asp-Glu-Leu) endoplasmic reticulum protein retention receptor 3	Hs.730819	12.09	2.68
KDM1A	lysine (K)-specific demethylase 1A	Hs.591518	8.45	18.04
KDM5C	lysine (K)-specific demethylase 5C	Hs.631768	2.70	3.67
KDSR	3-ketodihydrosphingosine reductase	Hs.74050	35.88	11.55
KGFLP1	fibroblast growth factor 7 pseudogene	Hs.535063	5.01	9.72
KHDRBS1	KH domain containing, RNA binding, signal transduction associated 1	Hs.445893	22.55	2.54
KHSRP	KH-type splicing regulatory protein	Hs.91142	2.72	3.25
KIF1B	kinesin family member 1B	Hs.97858	10.49	18.38
KIF20A	kinesin family member 20A	Hs.718626	18.46	39.22
KIF3B	kinesin family member 3B	Hs.369670	28.32	5.30
KIF5B	kinesin family member 5B	Hs.327736	23.58	18.73
KIFC1	kinesin family member C1	Hs.436912	5.27	5.95
KLC1	kinesin light chain 1	Hs.20107	9.53	13.67
KLC2	kinesin light chain 2	Hs.280792	3.07	5.12
KLF5	Kruppel-like factor 5 (intestinal)	Hs.508234	81.67	29.99
KLF6	Kruppel-like factor 6	Hs.4055	3.90	16.19
KLF7	Kruppel-like factor 7 (ubiquitous)	Hs.471221	8.09	13.16
KLHDC3	kelch domain containing 3	Hs.412468	7.90	13.41
KLHL12	kelch-like 12 (Drosophila)	Hs.706793	10.96	15.93
KLHL18	kelch-like 18 (Drosophila)	Hs.517946	10.66	4.72
KLHL26	kelch-like 26 (Drosophila)	Hs.250632	6.33	4.74
KLHL36	kelch-like 36 (Drosophila)	Hs.578546	6.44	2.72

KLHL7	kelch-like 7 (Drosophila)	Hs.654817	27.93	11.79
KRAS	v-Ki-ras2 Kirsten rat sarcoma viral oncogene homolog	Hs.505033	26.63	21.92
KRT13	keratin 13	Hs.654550	4.12	3.82
KRT5	keratin 5	Hs.433845	3.99	3.31
KRT6C	keratin 6C	Hs.709234	7.89	7.17
KRT80	keratin 80	Hs.140978	5.65	4.02
KTN1	kinectin 1 (kinesin receptor)	Hs.509414	151.23	18.96
LAMC2	laminin, gamma 2	Hs.591484	8.87	4.20
LAMP2	lysosomal-associated membrane protein 2	Hs.496684	36.46	22.51
LAMTOR1	late endosomal/lysosomal adaptor, MAPK and MTOR activator 1	Hs.530753	5.95	4.54
LAP3	leucine aminopeptidase 3	Hs.570791	9.05	3.11
LARP4B	La ribonucleoprotein domain family, member 4B	Hs.631814	4.71	5.16
LBR	lamin B receptor	Hs.435166	56.18	3.58
LDB1	LIM domain binding 1	Hs.454418	7.13	56.73
LDHB	lactate dehydrogenase B	Hs.446149	12.49	3.28
LDLRAP1	low density lipoprotein receptor adaptor protein 1	Hs.590911	10.77	24.23
LEMD3	LEM domain containing 3	Hs.728281	42.12	5.88
LENG1	leukocyte receptor cluster (LRC) member 1	Hs.590974	4.74	11.51
LENG8	leukocyte receptor cluster (LRC) member 8	Hs.502378	4.53	8.82
LEO1	Leo1, Paf1/RNA polymerase II complex component, homolog (S. cerevisiae)	Hs.567662	35.89	18.62
LETMD1	LETM1 domain containing 1	Hs.655272	13.83	2.68
LGALS8	lectin, galactoside-binding, soluble, 8	Hs.4082	7.34	11.47
LGALSL	lectin, galactoside-binding-like	Hs.372208	50.34	4.26
LGMN	legumain	Hs.18069	25.23	78.38
LGR4	leucine-rich repeat containing G protein-coupled receptor 4	Hs.502176	133.75	16.91
LHFPL2	lipoma HMGIC fusion partner-like 2	Hs.79299	26.91	4.36
LIMK2	LIM domain kinase 2	Hs.474596	5.37	3.35
LIMS1	LIM and senescent cell antigen-like domains 1	Hs.597715	21.76	4.47
LIN52	lin-52 homolog (C. elegans)	Hs.612866	27.97	7.97
LIN9	lin-9 homolog (C. elegans)	Hs.120817	17.98	7.17
LIPH	lipase, member H	Hs.68864	8.64	5.17
LITAF	lipopolysaccharide-induced TNF factor	Hs.459940	14.31	5.24
LIX1L	Lix1 homolog (mouse)-like	Hs.730828	19.36	9.37
LLPH	LLP homolog, long-term synaptic facilitation (Aplysia)	Hs.554187	63.58	21.87
LMAN2	lectin, mannose-binding 2	Hs.75864	5.27	7.00
LMO4	LIM domain only 4	Hs.436792	24.78	7.36
LNPEP	leucyl/cystinyl aminopeptidase	Hs.628728	49.31	26.66
LOC344887	NmrA-like family domain containing 1 pseudogene	Hs.128803	15.31	14.04
LOC643454	adaptor-related protein complex 3, sigma 1 subunit pseudogene	Hs.662086	18.95	3.78
LOC648044	guanine nucleotide binding protein (G protein), gamma 12-like	Hs.535131	5.36	2.74
LOC728061	hCG2003663	Hs.655858	3.66	58.23
LPAR2	lysophosphatidic acid receptor 2	Hs.122575	2.54	4.20
LPAR5	lysophosphatidic acid receptor 5	Hs.155538	8.65	9.95
LPCAT1	lysophosphatidylcholine acyltransferase 1	Hs.368853	9.64	13.89
LPCAT2	lysophosphatidylcholine acyltransferase 2	Hs.460857	57.08	34.56
LPCAT4	lysophosphatidylcholine acyltransferase 4	Hs.352614	44.52	106.45
LPHN2	latrophilin 2	Hs.24212	10.24	11.46

LPIN1	lipin 1	Hs.467740	7.60	7.93
LPPR2	lipid phosphate phosphatase-related protein type 2	Hs.6846	3.41	4.54
LRCH3	leucine-rich repeats and calponin homology (CH) domain containing 3	Hs.518414	6.00	9.04
LRIG2	leucine-rich repeats and immunoglobulin-like domains 2	Hs.448972	5.39	10.97
LRP10	low density lipoprotein receptor-related protein 10	Hs.525232	3.24	13.55
LRP12	low density lipoprotein receptor-related protein 12	Hs.600630	44.89	5.34
LRP5	low density lipoprotein receptor-related protein 5	Hs.6347	2.78	3.20
LRPPRC	leucine-rich PPR-motif containing	Hs.368084	21.86	20.44
LRR1	leucine rich repeat protein 1	Hs.451090	8.53	8.69
LRRC23	leucine rich repeat containing 23	Hs.155586	7.58	5.39
LRRC3	leucine rich repeat containing 3	Hs.326579	6.22	10.39
LRRC37A2	leucine rich repeat containing 37, member A2	Hs.730868	4.80	3.59
LRRC41	leucine rich repeat containing 41	Hs.144941	6.94	7.90
LRRC57	leucine rich repeat containing 57	Hs.234681	54.08	5.71
LRRC8D	leucine rich repeat containing 8 family, member D	Hs.482087	23.81	5.96
LRRFIP2	leucine rich repeat (in FLII) interacting protein 2	Hs.730738	27.60	15.70
LRRK1	leucine-rich repeat kinase 1	Hs.407918	3.73	5.02
LSG1	large subunit GTPase 1 homolog (S. cerevisiae)	Hs.728879	18.01	3.90
LSM12	LSM12 homolog (S. cerevisiae)	Hs.355570	14.77	8.46
LSM14A	LSM14A, SCD6 homolog A (S. cerevisiae)	Hs.407368	30.80	43.97
LSM14B	LSM14B, SCD6 homolog B (S. cerevisiae)	Hs.105379	3.21	17.60
LSM4	LSM4 homolog, U6 small nuclear RNA associated (S. cerevisiae)	Hs.515255	6.89	24.82
LSR	lipolysis stimulated lipoprotein receptor	Hs.466507	4.77	8.85
LTBR	lymphotoxin beta receptor (TNFR superfamily, member 3)	Hs.1116	6.61	2.67
LUZP6	leucine zipper protein 6	Hs.602015	17.25	8.06
LY6E	lymphocyte antigen 6 complex, locus E	Hs.521903	3.12	6.86
LYN	v-yes-1 Yamaguchi sarcoma viral related oncogene homolog	Hs.491767	23.08	5.74
LYPD1	LY6/PLAUR domain containing 1	Hs.728939	3.10	3.19
LYPD3	LY6/PLAUR domain containing 3	Hs.631594	6.85	11.54
LYPLA1	lysophospholipase I	Hs.730675	35.72	7.20
LYPLA2	lysophospholipase II	Hs.533479	5.35	3.06
LYRM4	LYR motif containing 4	Hs.387755	11.26	3.30
LYSMD2	LysM, putative peptidoglycan-binding, domain containing 2	Hs.603629	38.93	4.05
LYSMD4	LysM, putative peptidoglycan-binding, domain containing 4	Hs.562568	11.88	3.58
LZIC	leucine zipper and CTNNBIP1 domain containing	Hs.327252	27.16	7.99
LZTR1	leucine-zipper-like transcription regulator 1	Hs.78788	3.58	2.98
M6PR	mannose-6-phosphate receptor (cation dependent)	Hs.134084	12.73	8.88
MACF1	microtubule-actin crosslinking factor 1	Hs.472475	3.95	3.58
MAD2L1	MAD2 mitotic arrest deficient-like 1 (yeast)	Hs.591697	36.55	10.39
MAF1	MAF1 homolog (S. cerevisiae)	Hs.19673	3.14	3.96
MAFF	v-maf musculoaponeurotic fibrosarcoma oncogene homolog F (avian)	Hs.517617	8.68	11.37
MAFG	v-maf musculoaponeurotic fibrosarcoma oncogene homolog G (avian)	Hs.252229	6.63	4.18
MAFIP	MAFF interacting protein	Hs.721300	5.43	5.37
MAGEA12	melanoma antigen family A, 12	Hs.169246	13.08	3.31
MAGEA4	melanoma antigen family A, 4	Hs.37107	17.05	10.50
MAGED2	melanoma antigen family D, 2	Hs.522665	5.32	7.66
MAGEF1	melanoma antigen family F, 1	Hs.306123	16.36	12.36

MAGOH	mago-nashi homolog, proliferation-associated (Drosophila)	Hs.421576	23.40	4.97
MAL2	mal, T-cell differentiation protein 2 (gene/pseudogene)	Hs.201083	98.34	10.16
MAMDC4	MAM domain containing 4	Hs.376780	3.78	52.05
MAN1A2	mannosidase, alpha, class 1A, member 2	Hs.435938	13.60	5.05
MAN2B2	mannosidase, alpha, class 2B, member 2	Hs.188464	10.40	8.34
MANBA	mannosidase, beta A, lysosomal	Hs.480415	27.19	8.17
MAP1LC3B	microtubule-associated protein 1 light chain 3 beta	Hs.356061	26.78	9.81
MAP2K1	mitogen-activated protein kinase kinase 1	Hs.145442	30.79	9.76
MAP2K3	mitogen-activated protein kinase kinase 3	Hs.514012	5.20	10.65
MAP2K4	mitogen-activated protein kinase kinase 4	Hs.514681	39.05	6.43
MAP3K13	mitogen-activated protein kinase kinase kinase 13	Hs.591306	17.43	5.72
MAPK1	mitogen-activated protein kinase 1	Hs.431850	20.84	17.86
MAPK12	mitogen-activated protein kinase 12	Hs.432642	2.80	12.15
MAPK14	mitogen-activated protein kinase 14	Hs.485233	3.52	10.77
MAPK1IP1L	mitogen-activated protein kinase 1 interacting protein 1-like	Hs.594338	24.52	3.33
MAPK3	mitogen-activated protein kinase 3	Hs.861	4.71	4.58
MAPK8IP1	mitogen-activated protein kinase 8 interacting protein 1	Hs.234249	2.61	2.90
MAPKAP1	mitogen-activated protein kinase associated protein 1	Hs.495138	21.34	41.36
MAPKAPK2	mitogen-activated protein kinase-activated protein kinase 2	Hs.643566	6.03	3.74
MAPKAPK5	mitogen-activated protein kinase-activated protein kinase 5	Hs.413901	31.48	8.86
MAPRE1	microtubule-associated protein, RP/EB family, member 1	Hs.472437	17.84	7.48
MARCKSL1	MARCKS-like 1	Hs.75061	7.73	6.76
MARK3	MAP/microtubule affinity-regulating kinase 3	Hs.35828	31.01	4.78
MARVELD3	MARVEL domain containing 3	Hs.513706	5.27	3.39
MAT2A	methionine adenosyltransferase II, alpha	Hs.516157	5.37	3.35
MAT2B	methionine adenosyltransferase II, beta	Hs.54642	24.52	29.02
MATN2	matrilin 2	Hs.189445	5.70	7.80
MAX	MYC associated factor X	Hs.285354	9.58	17.08
MAZ	MYC-associated zinc finger protein (purine-binding transcription factor)	Hs.23650	4.23	40.79
MB21D1	Mab-21 domain containing 1	Hs.658405	7.56	5.07
MB21D2	Mab-21 domain containing 2	Hs.151443	10.93	3.63
MBD4	methyl-CpG binding domain protein 4	Hs.35947	37.77	8.58
MBD6	methyl-CpG binding domain protein 6	Hs.524523	3.23	2.97
MBIP	MAP3K12 binding inhibitory protein 1	Hs.368647	50.46	14.27
MBNL1	muscleblind-like (Drosophila)	Hs.201858	68.35	10.69
MBTD1	mbt domain containing 1	Hs.656803	13.49	10.70
MBTPS2	membrane-bound transcription factor peptidase, site 2	Hs.443490	4.54	4.20
MCCC2	methylcrotonoyl-CoA carboxylase 2 (beta)	Hs.604789	10.94	43.01
MCFD2	multiple coagulation factor deficiency 2	Hs.730655	15.20	8.29
MCM4	minichromosome maintenance complex component 4	Hs.460184	10.77	4.45
MCMBP	minichromosome maintenance complex binding protein	Hs.124246	31.56	2.70
MCOLN1	mucolipin 1	Hs.631858	2.85	4.65
MDM2	Mdm2 p53 binding protein homolog (mouse)	Hs.484551	9.18	17.21
MEAF6	MYST/Esa1-associated factor 6	Hs.17118	12.15	3.87
MED10	mediator complex subunit 10	Hs.13885	23.20	60.53
MED11	mediator complex subunit 11	Hs.513885	15.41	4.34
MED20	mediator complex subunit 20	Hs.278434	11.96	15.97

MED22	mediator complex subunit 22	Hs.78354	5.13	5.47
MED30	mediator complex subunit 30	Hs.492612	11.48	5.38
MED4	mediator complex subunit 4	Hs.181112	142.37	13.75
MED6	mediator complex subunit 6	Hs.497353	9.18	12.60
MED8	mediator complex subunit 8	Hs.301756	14.72	6.25
MEGF9	multiple EGF-like-domains 9	Hs.494977	94.41	26.12
MELK	maternal embryonic leucine zipper kinase	Hs.184339	70.58	11.44
MEN1	multiple endocrine neoplasia I	Hs.423348	7.59	3.73
MEPCE	methylphosphate capping enzyme	Hs.178011	2.82	7.54
MESDC2	mesoderm development candidate 2	Hs.578450	9.26	2.50
MEST	mesoderm specific transcript homolog (mouse)	Hs.270978	18.61	3.19
MET	met proto-oncogene (hepatocyte growth factor receptor)	Hs.132966	93.47	4.92
METAP1	methionyl aminopeptidase 1	Hs.480364	48.65	3.68
METAP2	methionyl aminopeptidase 2	Hs.444986	69.80	5.88
METTL10	methyltransferase like 10	Hs.468488	6.01	7.31
METTL17	methyltransferase like 17	Hs.512693	16.60	12.37
METTL21A	methyltransferase like 21A	Hs.664764	6.65	22.47
METTL21B	methyltransferase like 21B	Hs.632720	11.55	4.91
METTL5	methyltransferase like 5	Hs.470553	34.51	4.78
METTL7B	methyltransferase like 7B	Hs.51483	3.65	3.64
METTL8	methyltransferase like 8	Hs.135146	35.05	6.77
MEX3A	mex-3 homolog A (C. elegans)	Hs.591496	8.10	3.10
MEX3C	mex-3 homolog C (C. elegans)	Hs.465144	50.95	5.75
MFAP1	microfibrillar-associated protein 1	Hs.61418	41.74	3.22
MFAP2	microfibrillar-associated protein 2	Hs.389137	8.48	6.44
MFAP3	microfibrillar-associated protein 3	Hs.432818	105.92	26.07
MFGE8	milk fat globule-EGF factor 8 protein	Hs.3745	5.61	15.33
MFI2	antigen p97 (melanoma associated) identified by monoclonal antibodies 133.2 and 96.5	Hs.184727	7.19	13.86
MFSD10	major facilitator superfamily domain containing 10	Hs.632581	2.59	5.53
MGAT1	mannosyl (alpha-1, 3-)-glycoprotein beta-1, 2-N-acetylglucosaminyltransferase	Hs.519818	4.33	15.74
MGEA5	meningioma expressed antigen 5 (hyaluronidase)	Hs.500842	8.41	5.36
MGST1	microsomal glutathione S-transferase 1	Hs.389700	42.97	4.20
MGST3	microsomal glutathione S-transferase 3	Hs.191734	10.59	14.02
MICAL3	microtubule associated monoxygenase, calponin and LIM domain containing 3	Hs.528024	2.74	12.46
MIER1	mesoderm induction early response 1 homolog (Xenopus laevis)	Hs.605432	18.97	41.26
MIF4GD	MIF4G domain containing	Hs.325631	8.20	24.02
MINK1	misshapen-like kinase 1	Hs.443417	3.29	12.55
MINPP1	multiple inositol-polyphosphate phosphatase 1	Hs.121260	18.03	18.78
MIS18A	MIS18 kinetochore protein homolog A (S. pombe)	Hs.190518	39.84	3.65
MKI67	antigen identified by monoclonal antibody Ki-67	Hs.80976	11.40	9.21
MKKS	McKusick-Kaufman syndrome	Hs.472119	21.14	6.01
MKLN1	muskelin 1, intracellular mediator containing kelch motifs	Hs.44693	7.27	4.68
MKNK1	MAP kinase interacting serine/threonine kinase 1	Hs.371594	18.83	5.75
MKRN2	makorin ring finger protein 2	Hs.591666	24.55	3.84
MLEC	malectin	Hs.507074	17.12	22.86

MLF1	myeloid leukemia factor 1	Hs.85195	41.61	12.64
MLF2	myeloid leukemia factor 2	Hs.524214	11.61	6.50
MLLT6	myeloid/lymphoid or mixed-lineage leukemia (trithorax homolog, Drosophila); translocated to, 6	Hs.91531	2.92	9.08
MMACHC	methylmalonic aciduria (cobalamin deficiency) cblC type, with homocystinuria	Hs.13024	4.76	3.08
MMADHC	methylmalonic aciduria (cobalamin deficiency) cblD type, with homocystinuria	Hs.5324	18.78	2.74
MMGT1	membrane magnesium transporter 1	Hs.110702	70.95	4.10
MMP11	matrix metallopeptidase 11 (stromelysin 3)	Hs.143751	4.37	3.89
MMP14	matrix metallopeptidase 14 (membrane-inserted)	Hs.2399	3.30	11.56
MOB1A	MOB kinase activator 1A	Hs.728829	29.43	47.31
MOB3B	MOB kinase activator 3B	Hs.369022	35.67	11.73
MOB3C	MOB kinase activator 3C	Hs.632401	8.98	3.08
MOCS1	molybdenum cofactor synthesis 1	Hs.718492	4.38	5.11
MOCS2	molybdenum cofactor synthesis 2	Hs.163645	26.85	11.13
MORC4	MORC family CW-type zinc finger 4	Hs.496544	12.72	2.71
MORF4L1	mortality factor 4 like 1	Hs.374503	28.38	2.64
MORF4L2	mortality factor 4 like 2	Hs.326387	40.64	4.67
MORN4	MORN repeat containing 4	Hs.217409	12.24	5.07
MOSPD3	motile sperm domain containing 3	Hs.521086	12.69	4.45
MOV10	Mov10, Moloney leukemia virus 10, homolog (mouse)	Hs.514941	3.78	9.08
MPDU1	mannose-P-dolichol utilization defect 1	Hs.246381	3.54	10.57
MPG	N-methylpurine-DNA glycosylase	Hs.459596	5.33	2.58
MPRIP	myosin phosphatase Rho interacting protein	Hs.513971	3.41	13.20
MPZL1	myelin protein zero-like 1	Hs.493919	9.94	2.88
MR1	major histocompatibility complex, class I-related	Hs.13500	7.43	14.63
MREG	melanoregulin	Hs.620391	16.70	7.80
MRFAP1	Morf4 family associated protein 1	Hs.406590	21.51	18.27
MSI2	musashi homolog 2 (Drosophila)	Hs.658922	11.77	4.54
MSL3P1	male-specific lethal 3 homolog (Drosophila) pseudogene 1	Hs.355809	19.49	7.73
MSTO1	misato homolog 1 (Drosophila)	Hs.656547	3.13	6.19
MT1X	metallothionein 1×	Hs.374950	4.21	2.96
MTA2	metastasis associated 1 family, member 2	Hs.173043	3.74	3.70
MTA3	metastasis associated 1 family, member 3	Hs.435413	13.19	11.45
MTHFD1L	methylenetetrahydrofolate dehydrogenase (NADP+ dependent) 1-like	Hs.591343	4.39	5.73
MTHFD2	methylenetetrahydrofolate dehydrogenase (NADP+ dependent) 2, methenyltetrahydrofolate cyclohydrolase	Hs.469030	25.23	5.45
MTHFSD	methenyltetrahydrofolate synthetase domain containing	Hs.343627	8.59	4.99
MTMR1	myotubularin related protein 1	Hs.347187	9.79	7.54
MTSS1L	metastasis suppressor 1-like	Hs.432387	6.66	3.85
MTX2	metaxin 2	Hs.470728	35.77	8.94
MUC1	mucin 1, cell surface associated	Hs.89603	4.93	10.30
MUT	methylmalonyl CoA mutase	Hs.485527	79.57	4.58
MXD1	MAX dimerization protein 1	Hs.468908	7.81	10.67
MXD3	MAX dimerization protein 3	Hs.726161	2.74	3.15
MXI1	MAX interactor 1	Hs.501023	12.91	16.67
MXRA7	matrix-remodelling associated 7	Hs.250723	6.76	4.92

MYC	v-myc myelocytomatosis viral oncogene homolog (avian)	Hs.202453	4.77	8.46
MYD88	myeloid differentiation primary response gene (88)	Hs.82116	18.36	6.42
MYO1D	myosin ID	Hs.602063	6.15	8.50
MYO1E	myosin IE	Hs.654506	8.79	2.66
MYPOP	Myb-related transcription factor, partner of profilin	Hs.515478	6.84	21.72
NAA20	N(alpha)-acetyltransferase 20, NatB catalytic subunit	Hs.368783	25.65	5.11
NAA38	N(alpha)-acetyltransferase 38, NatC auxiliary subunit	Hs.655046	43.57	3.44
NAA40	N(alpha)-acetyltransferase 40, NatD catalytic subunit, homolog (S. cerevisiae)	Hs.523753	5.05	3.60
NAA50	N(alpha)-acetyltransferase 50, NatE catalytic subunit	Hs.372378	31.25	8.79
NAAA	N-acylethanolamine acid amidase	Hs.437365	9.66	4.10
NAB1	NGFI-A binding protein 1 (EGR1 binding protein 1)	Hs.730686	44.92	8.23
NACA	nascent polypeptide-associated complex alpha subunit	Hs.505735	18.08	3.68
NADK	NAD kinase	Hs.654792	4.75	10.41
NAGK	N-acetylglucosamine kinase	Hs.7036	6.51	3.04
NAP1L1	nucleosome assembly protein 1-like 1	Hs.524599	59.28	11.92
NAPA	N-ethylmaleimide-sensitive factor attachment protein, alpha	Hs.126938	5.72	10.29
NAPG	N-ethylmaleimide-sensitive factor attachment protein, gamma	Hs.464622	21.98	5.99
NARS	asparaginyl-tRNA synthetase	Hs.465224	68.23	9.35
NASP	nuclear autoantigenic sperm protein (histone-binding)	Hs.319334	27.85	3.79
NBAS	neuroblastoma amplified sequence	Hs.467759	5.00	3.21
NBR1	neighbor of BRCA1 gene 1	Hs.277721	11.64	10.60
NCAPD3	non-SMC condensin II complex, subunit D3	Hs.438550	19.03	3.18
NCK2	NCK adaptor protein 2	Hs.529244	4.95	4.71
NCKIPSD	NCK interacting protein with SH3 domain	Hs.655006	4.23	3.94
NCL	nucleolin	Hs.79110	34.04	3.50
NCOR1	nuclear receptor corepressor 1	Hs.462323	5.80	8.59
ND2	MTND2	Hs.631494	4.20	29.52
ND4	NADH dehydrogenase, subunit 4 (complex I)	Hs.465808	3.83	70.03
ND5	NADH dehydrogenase, subunit 5 (complex I)	Hs.723616	3.76	41.70
NDEL1	nudE nuclear distribution gene E homolog (A. nidulans)-like 1	Hs.372123	22.80	12.89
NDFIP1	Nedd4 family interacting protein 1	Hs.9788	10.35	9.62
NDFIP2	Nedd4 family interacting protein 2	Hs.525093	73.30	11.55
NDUFA10	NADH dehydrogenase (ubiquinone) 1 alpha subcomplex, 10, 42 kDa	Hs.277677	5.15	5.76
NDUFA4L2	NADH dehydrogenase (ubiquinone) 1 alpha subcomplex, 4-like 2	Hs.725525	10.99	34.57
NDUFA6	NADH dehydrogenase (ubiquinone) 1 alpha subcomplex, 6, 14 kDa	Hs.274416	4.79	2.73
NDUFA9	NADH dehydrogenase (ubiquinone) 1 alpha subcomplex, 9, 39 kDa	Hs.75227	3.42	3.78
NDUFAF4	NADH dehydrogenase (ubiquinone) 1 alpha subcomplex, assembly factor 4	Hs.512144	14.88	4.13
NDUFB5	NADH dehydrogenase (ubiquinone) 1 beta subcomplex, 5, 16 kDa	Hs.730674	13.08	2.50
NDUFB6	NADH dehydrogenase (ubiquinone) 1 beta subcomplex, 6, 17 kDa	Hs.493668	14.30	2.61
NDUFC2	NADH dehydrogenase (ubiquinone) 1, subcomplex unknown, 2, 14.5 kDa	Hs.407860	19.10	7.29
NDUFS2	NADH dehydrogenase (ubiquinone) Fe-S protein 2, 49 kDa (NADH-coenzyme Q reductase)	Hs.173611	5.04	6.19
NDUFS4	NADH dehydrogenase (ubiquinone) Fe-S protein 4, 18 kDa (NADH-coenzyme Q reductase)	Hs.528222	18.58	3.27
NEAT1	nuclear paraspeckle assembly transcript 1 (non-protein coding)	Hs.729910	28.62	34.26

NEDD1	neural precursor cell expressed, developmentally down-regulated 1	Hs.728880	264.98	2.98
NEDD4L	neural precursor cell expressed, developmentally down-regulated 4-like	Hs.185677	18.09	9.79
NEDD9	neural precursor cell expressed, developmentally down-regulated 9	Hs.673866	4.83	41.46
NEK6	NIMA (never in mitosis gene a)-related kinase 6	Hs.197071	5.60	7.15
NET1	neuroepithelial cell transforming 1	Hs.25155	21.79	3.33
NEURL1B	neuralized homolog 1B (Drosophila)	Hs.91521	4.11	4.78
NFIC	nuclear factor I/C (CCAAT-binding transcription factor)	Hs.170131	2.58	8.57
NFIX	nuclear factor I/X (CCAAT-binding transcription factor)	Hs.257970	2.73	13.15
NFKB2	nuclear factor of kappa light polypeptide gene enhancer in B-cells 2 (p49/p100)	Hs.73090	2.94	7.32
NFKBIB	nuclear factor of kappa light polypeptide gene enhancer in B-cells inhibitor, beta	Hs.9731	5.20	9.13
NFX1	nuclear transcription factor, X-box binding 1	Hs.413074	7.52	4.74
NFYC	nuclear transcription factor Y, gamma	Hs.713051	11.92	5.64
NGFR	nerve growth factor receptor	Hs.415768	3.44	3.57
NGRN	neugrin, neurite outgrowth associated	Hs.730606	10.43	8.96
NIF3L1	NIF3 NGG1 interacting factor 3-like 1 (S. cerevisiae)	Hs.145284	29.61	4.18
NINJ2	ninjurin 2	Hs.656450	6.96	3.48
NINL	ninein-like	Hs.631508	3.19	5.49
NIPSNAP1	nipsnap homolog 1 (C. elegans)	Hs.173878	14.51	14.01
NIPSNAP3A	nipsnap homolog 3A (C. elegans)	Hs.530275	24.16	3.67
NIT1	nitrilase 1	Hs.599361	6.35	2.67
NKAP	NFKB activating protein	Hs.522771	22.63	4.63
NKIRAS2	NFKB inhibitor interacting Ras-like 2	Hs.632252	8.69	4.72
NME4	non-metastatic cells 4, protein expressed in	Hs.9235	2.62	5.31
NNAT	neuronatin	Hs.504703	2.69	340.79
NOB1	NIN1/RPN12 binding protein 1 homolog (S. cerevisiae)	Hs.271695	13.60	8.22
NOL10	nucleolar protein 10	Hs.222494	8.56	12.08
NOL11	nucleolar protein 11	Hs.463936	40.30	12.35
NOLC1	nucleolar and coiled-body phosphoprotein 1	Hs.523238	19.91	3.04
NONO	non-POU domain containing, octamer-binding	Hs.533282	8.77	5.01
NOP56	NOP56 ribonucleoprotein homolog (yeast)	Hs.376064	2.51	3.49
NPC1	Niemann-Pick disease, type C1	Hs.715623	14.65	6.92
NPEPPS	aminopeptidase puromycin sensitive	Hs.443837	5.09	11.96
NPNT	nephronectin	Hs.518921	65.19	15.78
NPR3	natriuretic peptide receptor C/guanylate cyclase C (atrionatriuretic peptide receptor C)	Hs.13528	23.80	6.87
NPTN	neuroplastin	Hs.187866	11.60	19.09
NQO1	NAD(P)H dehydrogenase, quinone 1	Hs.406515	12.64	6.57
NR1H2	nuclear receptor subfamily 1, group H, member 2	Hs.432976	7.05	7.50
NR2C2AP	nuclear receptor 2C2-associated protein	Hs.708153	13.10	6.38
NRARP	NOTCH-regulated ankyrin repeat protein	Hs.535075	9.51	7.37
NRAS	neuroblastoma RAS viral (v-ras) oncogene homolog	Hs.486502	18.28	19.62
NRBF2	nuclear receptor binding factor 2	Hs.449628	10.44	25.50
NRBP1	nuclear receptor binding protein 1	Hs.515876	7.61	7.55
NRBP2	nuclear receptor binding protein 2	Hs.521926	7.60	4.13
NRD1	nardilysin (N-arginine dibasic convertase)	Hs.584782	25.92	4.35

NRG1	neuregulin 1	Hs.668810	16.80	3.05
NRGN	neurogranin (protein kinase C substrate, RC3)	Hs.524116	4.66	3.29
NRIP3	nuclear receptor interacting protein 3	Hs.523467	5.60	6.02
NRL	neural retina leucine zipper	Hs.652297	2.76	2.55
NRP1	neuropilin 1	Hs.131704	7.71	17.09
NRP2	neuropilin 2	Hs.471200	4.21	11.48
NRSN2	neurensin 2	Hs.416024	3.27	3.38
NSD1	nuclear receptor binding SET domain protein 1	Hs.106861	3.47	10.97
NSF	N-ethylmaleimide-sensitive factor	Hs.431279	11.64	10.95
NSFL1C	NSFL1 (p97) cofactor (p47)	Hs.12865	6.81	5.33
NSMAF	neutral sphingomyelinase (N-SMase) activation associated factor	Hs.372000	69.57	12.86
NSMCE2	non-SMC element 2, MMS21 homolog (S. cerevisiae)	Hs.388297	17.82	8.12
NSMCE4A	non-SMC element 4 homolog A (S. cerevisiae)	Hs.258798	18.59	4.89
NSUN4	NOP2/Sun domain family, member 4	Hs.163424	7.38	2.79
NT5DC1	5′-nucleotidase domain containing 1	Hs.520341	50.62	14.01
NTAN1	N-terminal asparagine amidase	Hs.592045	8.70	8.67
NTPCR	nucleoside-triphosphatase, cancer-related	Hs.642715	4.20	4.03
NTS	neurotensin	Hs.80962	53.31	5.70
NUAK1	NUAK family, SNF1-like kinase, 1	Hs.728863	3.40	6.15
NUCB2	nucleobindin 2	Hs.654599	137.48	15.83
NUDC	nuclear distribution gene C homolog (A. nidulans)	Hs.263812	3.32	3.75
NUDCD2	NudC domain containing 2	Hs.140443	42.32	3.01
NUDT12	nudix (nucleoside diphosphate linked moiety X)-type motif 12	Hs.434289	2.79	2.97
NUDT16	nudix (nucleoside diphosphate linked moiety X)-type motif 16	Hs.282050	7.83	23.97
NUDT19	nudix (nucleoside diphosphate linked moiety X)-type motif 19	Hs.203961	21.22	4.92
NUDT2	nudix (nucleoside diphosphate linked moiety X)-type motif 2	Hs.493767	6.02	3.86
NUDT21	nudix (nucleoside diphosphate linked moiety X)-type motif 21	Hs.528834	25.53	19.93
NUDT5	nudix (nucleoside diphosphate linked moiety X)-type motif 5	Hs.555956	9.61	2.85
NUDT9	nudix (nucleoside diphosphate linked moiety X)-type motif 9	Hs.149500	41.93	2.88
NUP153	nucleoporin 153 kDa	Hs.601591	31.91	3.76
NUP155	nucleoporin 155 kDa	Hs.547696	33.17	10.26
NUP188	nucleoporin 188 kDa	Hs.308340	3.11	6.63
NUP210	nucleoporin 210 kDa	Hs.475525	3.85	2.71
NUP43	nucleoporin 43 kDa	Hs.510375	15.27	7.01
NUP50	nucleoporin 50 kDa	Hs.475103	25.38	17.11
NUP88	nucleoporin 88 kDa	Hs.584784	16.48	6.68
NUP93	nucleoporin 93 kDa	Hs.276878	9.85	9.52
NUP98	nucleoporin 98 kDa	Hs.524750	14.34	3.91
NUPL2	nucleoporin like 2	Hs.408241	25.41	4.40
NUS1	nuclear undecaprenyl pyrophosphate synthase 1 homolog (S. cerevisiae)	Hs.289008	33.33	12.16
NUSAP1	nucleolar and spindle associated protein 1	Hs.615092	56.33	3.09
NUTF2	nuclear transport factor 2	Hs.356630	11.57	16.97
NXF1	nuclear RNA export factor 1	Hs.523739	5.42	5.51
OAF	OAF homolog (Drosophila)	Hs.445081	12.60	12.38
OAS3	2′-5′-oligoadenylate synthetase 3, 100 kDa	Hs.528634	3.64	11.12
OCIAD1	OCIA domain containing 1	Hs.518750	56.81	7.90
OCLN	occludin	Hs.592605	12.42	54.16

OGG1	8-oxoguanine DNA glycosylase	Hs.380271	9.39	7.81
OLFM2	olfactomedin 2	Hs.169743	13.11	16.89
OMA1	OMA1 homolog, zinc metallopeptidase (S. cerevisiae)	Hs.425769	49.26	6.54
ORAI2	ORAI calcium release-activated calcium modulator 2	Hs.363308	4.29	3.20
ORC1	origin recognition complex, subunit 1	Hs.17908	6.88	4.46
ORC3	origin recognition complex, subunit 3	Hs.410228	34.61	5.31
ORC5	origin recognition complex, subunit 5	Hs.432948	52.57	19.79
ORC6	origin recognition complex, subunit 6	Hs.49760	65.58	22.54
ORMDL1	ORM1-like 1 (S. cerevisiae)	Hs.700632	22.51	31.20
ORMDL3	ORM1-like 3 (S. cerevisiae)	Hs.514151	4.42	6.53
OSBP	oxysterol binding protein	Hs.597091	22.92	6.13
OSBPL1A	oxysterol binding protein-like 1A	Hs.370725	18.23	6.46
OSBPL9	oxysterol binding protein-like 9	Hs.21938	33.93	17.46
OSMR	oncostatin M receptor	Hs.120658	13.19	7.19
OSR2	odd-skipped related 2 (Drosophila)	Hs.253247	3.21	2.75
OSTC	oligosaccharyltransferase complex subunit	Hs.445803	36.45	10.78
OTOA	otoancorin	Hs.408336	3.43	9.87
OTUD1	OTU domain containing 1	Hs.499042	12.36	2.70
OTUD5	OTU domain containing 5	Hs.496098	5.81	3.03
OXA1L	oxidase (cytochrome c) assembly 1-like	Hs.151134	14.90	5.23
OXSR1	oxidative-stress responsive 1	Hs.475970	64.76	8.56
P2RX5	purinergic receptor P2X, ligand-gated ion channel, 5	Hs.12956	3.55	4.88
P2RY1	purinergic receptor P2Y, G-protein coupled, 1	Hs.654526	8.81	9.01
P4HA1	prolyl 4-hydroxylase, alpha polypeptide I	Hs.500047	19.65	3.10
PABPC1	poly(A) binding protein, cytoplasmic 1	Hs.387804	25.94	8.48
PABPC4	poly(A) binding protein, cytoplasmic 4 (inducible form)	Hs.169900	6.38	9.18
PACSIN2	protein kinase C and casein kinase substrate in neurons 2	Hs.162877	3.15	3.64
PACSIN3	protein kinase C and casein kinase substrate in neurons 3	Hs.334639	3.16	6.93
PAF1	Paf1, RNA polymerase II associated factor, homolog (S. cerevisiae)	Hs.466714	3.62	2.91
PAICS	phosphoribosylaminoimidazole carboxylase, phosphoribosylaminoimidazole succinocarboxamide synthetase	Hs.518774	54.62	4.74
PAIP1	poly(A) binding protein interacting protein 1	Hs.482038	109.79	21.67
PAIP2	poly(A) binding protein interacting protein 2	Hs.396644	92.10	9.95
PAIP2B	poly(A) binding protein interacting protein 2B	Hs.416735	9.00	8.93
PAK4	p21 protein (Cdc42/Rac)-activated kinase 4	Hs.20447	3.84	12.93
PALLD	palladin, cytoskeletal associated protein	Hs.151220	8.63	12.76
PAM	peptidylglycine alpha-amidating monooxygenase	Hs.369430	18.75	3.49
PANK2	pantothenate kinase 2	Hs.516859	18.25	7.87
PANK3	pantothenate kinase 3	Hs.591729	78.94	30.96
PANK4	pantothenate kinase 4	Hs.26156	4.71	3.80
PANX2	pannexin 2	Hs.440092	2.80	9.12
PAPOLG	poly(A) polymerase gamma	Hs.387471	41.56	4.92
PAPSS1	3′-phosphoadenosine 5′-phosphosulfate synthase 1	Hs.368610	7.69	9.77
PAQR4	progestin and adipoQ receptor family member IV	Hs.351474	3.40	4.66
PAQR7	progestin and adipoQ receptor family member VII	Hs.523652	3.70	19.90
PARN	poly(A)-specific ribonuclease	Hs.253197	18.61	6.32
PARP1	poly (ADP-ribose) polymerase 1	Hs.177766	4.60	12.28
PARP12	poly (ADP-ribose) polymerase family, member 12	Hs.12646	5.78	3.04

PARP6	poly (ADP-ribose) polymerase family, member 6	Hs.270244	4.81	5.40
PARVB	parvin, beta	Hs.475074	2.85	23.17
PATL1	protein associated with topoisomerase II homolog 1 (yeast)	Hs.591960	16.14	6.02
PAX9	paired box 9	Hs.132576	10.98	6.02
PBK	PDZ binding kinase	Hs.104741	42.19	5.96
PBX2	pre-B-cell leukemia homeobox 2	Hs.509545	9.21	9.73
PBX3	pre-B-cell leukemia homeobox 3	Hs.428027	3.79	5.45
PBXIP1	pre-B-cell leukemia homeobox interacting protein 1	Hs.505806	3.16	17.02
PCBD2	pterin-4 alpha-carbinolamine dehydratase/dimerization cofactor of hepatocyte nuclear factor 1 alpha (TCF1) 2	Hs.710014	9.50	2.59
PCBP1	poly(rC) binding protein 1	Hs.2853	9.97	3.62
PCBP2	poly(rC) binding protein 2	Hs.546271	10.44	12.10
PCCA	propionyl CoA carboxylase, alpha polypeptide	Hs.80741	33.25	3.40
PCCB	propionyl CoA carboxylase, beta polypeptide	Hs.63788	16.62	4.00
PCDHB12	protocadherin beta 12	Hs.429820	3.72	2.76
PCDHB15	protocadherin beta 15	Hs.130757	22.66	5.65
PCDHGA2	protocadherin gamma subfamily A, 2	Hs.368160	3.58	5.45
PCDHGA8	protocadherin gamma subfamily A, 8	Hs.368160	4.74	14.62
PCGF1	polycomb group ring finger 1	Hs.316750	16.66	7.74
PCID2	PCI domain containing 2	Hs.729648	9.63	3.06
PCK2	phosphoenolpyruvate carboxykinase 2 (mitochondrial)	Hs.75812	2.66	10.07
PCMT1	protein-L-isoaspartate (D-aspartate) O-methyltransferase	Hs.279257	13.72	9.70
PCNA	proliferating cell nuclear antigen	Hs.147433	23.35	6.07
PCYOX1	prenylcysteine oxidase 1	Hs.567502	23.58	3.18
PCYT1A	phosphate cytidylyltransferase 1, choline, alpha	Hs.135997	4.29	2.50
PDAP1	PDGFA associated protein 1	Hs.632296	4.29	17.54
PDCD10	programmed cell death 10	Hs.478150	49.76	6.03
PDCD11	programmed cell death 11	Hs.239499	2.62	4.26
PDCD2	programmed cell death 2	Hs.367900	12.17	3.31
PDCD2L	programmed cell death 2-like	Hs.515344	18.14	2.74
PDCD5	programmed cell death 5	Hs.443831	38.02	5.52
PDCD6	programmed cell death 6	Hs.50823	7.24	14.56
PDCL	phosducin-like	Hs.271749	22.29	32.08
PDCL3	phosducin-like 3	Hs.720825	11.98	2.76
PDGFA	platelet-derived growth factor alpha polypeptide	Hs.535898	5.22	3.39
PDGFB	platelet-derived growth factor beta polypeptide	Hs.1976	11.03	10.36
PDGFC	platelet derived growth factor C	Hs.570855	43.85	2.73
PDIA3	protein disulfide isomerase family A, member 3	Hs.591095	10.32	6.68
PDIA4	protein disulfide isomerase family A, member 4	Hs.93659	5.65	7.21
PDIA5	protein disulfide isomerase family A, member 5	Hs.477352	12.07	8.11
PDIA6	protein disulfide isomerase family A, member 6	Hs.212102	13.32	20.64
PDK3	pyruvate dehydrogenase kinase, isozyme 3	Hs.296031	10.84	3.92
PDLIM1	PDZ and LIM domain 1	Hs.368525	15.33	18.15
PDLIM5	PDZ and LIM domain 5	Hs.480311	8.17	15.55
PDPK1	3-phosphoinositide dependent protein kinase-1	Hs.459691	6.79	3.15
PDPN	podoplanin	Hs.468675	8.03	2.99
PDSS1	prenyl (decaprenyl) diphosphate synthase, subunit 1	Hs.558468	7.73	8.89
PDSS2	prenyl (decaprenyl) diphosphate synthase, subunit 2	Hs.730764	39.83	12.20

PDXDC1	pyridoxal-dependent decarboxylase domain containing 1	Hs.370781	15.24	10.44
PDXP	pyridoxal (pyridoxine, vitamin B6) phosphatase	Hs.632762	4.82	3.84
PEA15	phosphoprotein enriched in astrocytes 15	Hs.517216	15.23	13.92
PEBP1	phosphatidylethanolamine binding protein 1	Hs.433863	9.24	3.68
PECR	peroxisomal trans-2-enoyl-CoA reductase	Hs.281680	11.14	6.26
PEF1	penta-EF-hand domain containing 1	Hs.470417	8.42	4.93
PELI2	pellino homolog 2 (Drosophila)	Hs.657926	46.37	5.28
PES1	pescadillo homolog 1, containing BRCT domain (zebrafish)	Hs.517543	5.19	8.29
PET112	PET112 homolog (yeast)	Hs.119316	7.26	7.74
PEX11A	peroxisomal biogenesis factor 11 alpha	Hs.31034	7.14	2.75
PEX13	peroxisomal biogenesis factor 13	Hs.161377	40.98	8.26
PFDN2	prefoldin subunit 2	Hs.492516	21.97	6.82
PFDN4	prefoldin subunit 4	Hs.91161	54.81	20.60
PFKFB3	6-phosphofructo-2-kinase/fructose-2, 6-biphosphatase 3	Hs.195471	4.12	8.20
PFN1	profilin 1	Hs.494691	10.26	25.74
PFN2	profilin 2	Hs.91747	73.12	40.70
PGAM1	phosphoglycerate mutase 1 (brain)	Hs.632918	6.75	2.70
PGAM5	phosphoglycerate mutase family member 5	Hs.102558	4.13	58.11
PGD	phosphogluconate dehydrogenase	Hs.464071	10.00	3.85
PGM5P2	phosphoglucomutase 5 pseudogene 2	Hs.571593	14.82	6.57
PGS1	phosphatidylglycerophosphate synthase 1	Hs.654671	5.46	5.61
PHB2	prohibitin 2	Hs.504620	10.34	2.89
PHC1	polyhomeotic homolog 1 (Drosophila)	Hs.305985	5.72	52.24
PHC2	polyhomeotic homolog 2 (Drosophila)	Hs.524271	10.36	6.18
PHF1	PHD finger protein 1	Hs.166204	5.54	3.49
PHF23	PHD finger protein 23	Hs.644724	9.29	7.70
PHF5A	PHD finger protein 5A	Hs.474980	22.01	11.24
PHF6	PHD finger protein 6	Hs.356501	9.40	11.97
PHKB	phosphorylase kinase, beta	Hs.78060	42.52	4.94
PHKG2	phosphorylase kinase, gamma 2 (testis)	Hs.65735	3.52	4.84
PHYH	phytanoyl-CoA 2-hydroxylase	Hs.498732	12.52	8.03
PI4K2A	phosphatidylinositol 4-kinase type 2 alpha	Hs.25300	5.67	3.67
PIAS2	protein inhibitor of activated STAT, 2	Hs.658013	57.79	10.68
PICALM	phosphatidylinositol binding clathrin assembly protein	Hs.163893	26.40	18.12
PIGF	phosphatidylinositol glycan anchor biosynthesis, class F	Hs.468415	30.52	3.43
PIGH	phosphatidylinositol glycan anchor biosynthesis, class H	Hs.553497	16.63	3.02
PIGU	phosphatidylinositol glycan anchor biosynthesis, class U	Hs.253319	15.55	9.96
PIGY	phosphatidylinositol glycan anchor biosynthesis, class Y	Hs.26136	16.02	5.02
PIK3R4	phosphoinositide-3-kinase, regulatory subunit 4	Hs.149032	27.02	21.81
PILRB	paired immunoglobin-like type 2 receptor beta	Hs.632314	6.71	4.72
PINX1	PIN2/TERF1 interacting, telomerase inhibitor 1	Hs.490991	6.22	3.55
PIP4K2C	phosphatidylinositol-5-phosphate 4-kinase, type II, gamma	Hs.144502	10.48	7.86
PIP5K1C	phosphatidylinositol-4-phosphate 5-kinase, type I, gamma	Hs.282177	2.53	4.53
PITPNB	phosphatidylinositol transfer protein, beta	Hs.705323	97.12	7.70
PITRM1	pitrilysin metallopeptidase 1	Hs.528300	4.86	3.81
PKIG	protein kinase (cAMP-dependent, catalytic) inhibitor gamma	Hs.472831	4.57	7.57
PKN2	protein kinase N2	Hs.440833	12.23	24.04

PLAU	plasminogen activator, urokinase	Hs.77274	14.32	3.99
PLAUR	plasminogen activator, urokinase receptor	Hs.466871	10.24	12.58
PLBD2	phospholipase B domain containing 2	Hs.115896	3.15	10.04
PLCXD1	phosphatidylinositol-specific phospholipase C, X domain containing 1	Hs.522568	3.64	4.64
PLD1	phospholipase D1, phosphatidylcholine-specific	Hs.382865	18.56	7.31
PLD3	phospholipase D family, member 3	Hs.257008	3.46	6.72
PLEKHA1	pleckstrin homology domain containing, family A (phosphoinositide binding specific) member 1	Hs.643512	45.07	5.20
PLEKHB2	pleckstrin homology domain containing, family B (evectins) member 2	Hs.469944	9.07	3.67
PLEKHF2	pleckstrin homology domain containing, family F (with FYVE domain) member 2	Hs.29724	58.87	7.34
PLEKHG6	pleckstrin homology domain containing, family G (with RhoGef domain) member 6	Hs.631660	3.57	10.12
PLEKHJ1	pleckstrin homology domain containing, family J member 1	Hs.501353	2.56	3.74
PLIN3	perilipin 3	Hs.140452	5.66	9.20
PLK1	polo-like kinase 1	Hs.592049	8.87	26.86
PLK2	polo-like kinase 2	Hs.398157	10.32	3.05
PLLP	plasmolipin	Hs.632215	8.93	21.65
PLOD1	procollagen-lysine, 2-oxoglutarate 5-dioxygenase 1	Hs.75093	2.75	13.66
PLOD2	procollagen-lysine, 2-oxoglutarate 5-dioxygenase 2	Hs.477866	175.74	24.86
PLRG1	pleiotropic regulator 1	Hs.249996	7.71	2.75
PLS3	plastin 3	Hs.496622	228.36	39.86
PLSCR3	phospholipid scramblase 3	Hs.534591	6.30	14.40
PMF1-BGLAP	PMF1-BGLAP readthrough	Hs.530479	4.19	5.64
PMM1	phosphomannomutase 1	Hs.75835	3.82	2.61
PMM2	phosphomannomutase 2	Hs.625732	17.54	4.85
PMS2	PMS2 postmeiotic segregation increased 2 (S. cerevisiae)	Hs.659871	9.30	6.09
PMS2L2	postmeiotic segregation increased 2-like 2 pseudogene	Hs.729714	5.66	29.38
PNKD	paroxysmal nonkinesigenic dyskinesia	Hs.98475	6.33	2.80
PNMA1	paraneoplastic antigen MA1	Hs.194709	21.61	2.64
PODXL2	podocalyxin-like 2	Hs.591290	3.51	5.97
POGZ	pogo transposable element with ZNF domain	Hs.489873	3.43	4.07
POLA2	polymerase (DNA directed), alpha 2 (70 kD subunit)	Hs.201897	4.72	5.68
POLD3	polymerase (DNA-directed), delta 3, accessory subunit	Hs.82502	23.10	4.17
POLDIP3	polymerase (DNA-directed), delta interacting protein 3	Hs.505802	3.94	3.88
POLG2	polymerase (DNA directed), gamma 2, accessory subunit	Hs.437009	39.95	3.44
POLR1D	polymerase (RNA) I polypeptide D, 16 kDa	Hs.507584	18.88	6.78
POLR2B	polymerase (RNA) II (DNA directed) polypeptide B, 140 kDa	Hs.602757	37.24	12.88
POLR2J	polymerase (RNA) II (DNA directed) polypeptide J, 13.3 kDa	Hs.654952	3.77	4.78
POLR2J4	polymerase (RNA) II (DNA directed) polypeptide J4, pseudogene	Hs.657028	4.51	14.49
POLR2K	polymerase (RNA) II (DNA directed) polypeptide K, 7.0 kDa	Hs.351475	37.20	5.90
POLR3D	polymerase (RNA) III (DNA directed) polypeptide D, 44 kDa	Hs.148342	5.85	2.88
POPDC3	popeye domain containing 3	Hs.458336	50.49	4.84
POU2F2	POU class 2 homeobox 2	Hs.654420	4.76	8.24
PPA1	pyrophosphatase (inorganic) 1	Hs.437403	40.05	4.21
PPAN	peter pan homolog (Drosophila)	Hs.14468	4.24	5.25
PPAP2C	phosphatidic acid phosphatase type 2C	Hs.465506	4.77	4.82
PPAPDC1B	phosphatidic acid phosphatase type 2 domain containing 1B	Hs.567619	31.64	4.35

PPAPDC2	phosphatidic acid phosphatase type 2 domain containing 2	Hs.107510	49.94	7.90
PPAT	phosphoribosyl pyrophosphate amidotransferase	Hs.331420	53.46	24.18
PPCS	phosphopantothenoylcysteine synthetase	Hs.706662	6.85	14.00
PPFIA1	protein tyrosine phosphatase, receptor type, f polypeptide (PTPRF), interacting protein (liprin), alpha 1	Hs.530749	25.12	25.54
PPFIBP1	PTPRF interacting protein, binding protein 1 (liprin beta 1)	Hs.172445	19.92	14.66
PPHLN1	periphilin 1	Hs.444157	6.04	11.94
PPIC	peptidylprolyl isomerase C (cyclophilin C)	Hs.110364	28.54	3.72
PPID	peptidylprolyl isomerase D	Hs.183958	99.55	7.48
PPIE	peptidylprolyl isomerase E (cyclophilin E)	Hs.524690	8.02	5.00
PPIH	peptidylprolyl isomerase H (cyclophilin H)	Hs.256639	2.72	30.86
PPM1A	protein phosphatase, Mg2+/Mn2+ dependent, 1A	Hs.130036	12.32	28.85
PPM1G	protein phosphatase, Mg2+/Mn2+ dependent, 1G	Hs.643951	5.06	3.98
PPM1K	protein phosphatase, Mg2+/Mn2+ dependent, 1K	Hs.291000	3.24	8.65
PPM1M	protein phosphatase, Mg2+/Mn2+ dependent, 1M	Hs.373560	6.50	5.55
PPM1N	protein phosphatase, Mg2+/Mn2+ dependent, 1N (putative)	Hs.532872	22.73	7.30
PPME1	protein phosphatase methylesterase 1	Hs.503251	3.92	7.26
PPP1CA	protein phosphatase 1, catalytic subunit, alpha isozyme	Hs.183994	2.77	4.67
PPP1CC	protein phosphatase 1, catalytic subunit, gamma isozyme	Hs.79081	26.40	5.29
PPP1R11	protein phosphatase 1, regulatory (inhibitor) subunit 11	Hs.82887	16.77	245.01
PPP1R15A	protein phosphatase 1, regulatory subunit 15A	Hs.631593	13.13	21.42
PPP1R18	protein phosphatase 1, regulatory subunit 18	Hs.101150	4.52	5.35
PPP1R2	protein phosphatase 1, regulatory (inhibitor) subunit 2	Hs.535731	14.06	15.53
PPP1R7	protein phosphatase 1, regulatory subunit 7	Hs.36587	18.07	4.19
PPP1R8	protein phosphatase 1, regulatory subunit 8	Hs.533474	12.77	3.02
PPP2CA	protein phosphatase 2, catalytic subunit, alpha isozyme	Hs.105818	29.14	10.87
PPP2CB	protein phosphatase 2, catalytic subunit, beta isozyme	Hs.491440	25.55	16.43
PPP2R1A	protein phosphatase 2, regulatory subunit A, alpha	Hs.467192	4.04	6.94
PPP2R2A	protein phosphatase 2, regulatory subunit B, alpha	Hs.146339	82.23	14.03
PPP2R3C	protein phosphatase 2, regulatory subunit B'', gamma	Hs.530712	26.37	3.30
PPP2R5E	protein phosphatase 2, regulatory subunit B', epsilon isoform	Hs.334868	55.10	6.31
PPP4C	protein phosphatase 4, catalytic subunit	Hs.534338	5.86	5.47
PPP4R2	protein phosphatase 4, regulatory subunit 2	Hs.431092	15.94	47.67
PPP5C	protein phosphatase 5, catalytic subunit	Hs.654604	10.60	60.52
PQBP1	polyglutamine binding protein 1	Hs.534384	4.52	3.30
PQLC1	PQ loop repeat containing 1	Hs.288284	2.99	6.01
PQLC3	PQ loop repeat containing 3	Hs.274415	26.66	3.51
PRAME	preferentially expressed antigen in melanoma	Hs.30743	2.72	11.76
PRC1	protein regulator of cytokinesis 1	Hs.366401	40.00	3.55
PRDX3	peroxiredoxin 3	Hs.523302	48.24	18.50
PRELID2	PRELI domain containing 2	Hs.314261	16.72	4.05
PREP	prolyl endopeptidase	Hs.436564	9.45	6.31
PRICKLE3	prickle homolog 3 (Drosophila)	Hs.632802	2.93	17.13
PRIM1	primase, DNA, polypeptide 1 (49 kDa)	Hs.534339	23.65	4.97
PRIM2	primase, DNA, polypeptide 2 (58 kDa)	Hs.654580	26.34	10.41
PRKACA	protein kinase, cAMP-dependent, catalytic, alpha	Hs.631630	9.73	15.43
PRKAR1B	protein kinase, cAMP-dependent, regulatory, type I, beta	Hs.520851	3.53	11.18
PRKCSH	protein kinase C substrate 80K-H	Hs.610830	2.60	15.39

PRKRIP1	PRKR interacting protein 1 (IL11 inducible)	Hs.406395	4.58	4.24
PRMT2	protein arginine methyltransferase 2	Hs.154163	27.33	37.73
PRMT5	protein arginine methyltransferase 5	Hs.367854	6.02	2.64
PRMT6	protein arginine methyltransferase 6	Hs.26006	21.18	2.53
PRODH	proline dehydrogenase (oxidase) 1	Hs.517352	3.05	3.74
PROSC	proline synthetase co-transcribed homolog (bacterial)	Hs.304792	16.49	7.32
PRPF31	PRP31 pre-mRNA processing factor 31 homolog (S. cerevisiae)	Hs.515598	7.56	3.07
PRPSAP2	phosphoribosyl pyrophosphate synthetase-associated protein 2	Hs.632236	28.78	3.65
PRR11	proline rich 11	Hs.631750	19.21	13.00
PRR14	proline rich 14	Hs.293629	8.67	4.77
PRRC1	proline-rich coiled-coil 1	Hs.483259	25.03	14.74
PRRC2C	proline-rich coiled-coil 2C	Hs.494614	23.46	14.04
PRRG2	proline rich Gla (G-carboxyglutamic acid) 2	Hs.35101	2.55	2.86
PRRG4	proline rich Gla (G-carboxyglutamic acid) 4 (transmembrane)	Hs.471695	34.54	9.51
PRSS16	protease, serine, 16 (thymus)	Hs.274407	8.40	5.65
PSAP	prosaposin	Hs.523004	12.47	16.56
PSAT1	phosphoserine aminotransferase 1	Hs.494261	26.07	5.99
PSEN1	presenilin 1	Hs.3260	10.88	7.11
PSMA1	proteasome (prosome, macropain) subunit, alpha type, 1	Hs.102798	5.32	16.92
PSMA2	proteasome (prosome, macropain) subunit, alpha type, 2	Hs.333786	16.78	3.23
PSMA3	proteasome (prosome, macropain) subunit, alpha type, 3	Hs.558799	55.69	11.77
PSMA4	proteasome (prosome, macropain) subunit, alpha type, 4	Hs.251531	18.56	5.08
PSMA7	proteasome (prosome, macropain) subunit, alpha type, 7	Hs.233952	5.73	6.62
PSMB2	proteasome (prosome, macropain) subunit, beta type, 2	Hs.471441	6.18	2.73
PSMB8	proteasome (prosome, macropain) subunit, beta type, 8 (large multifunctional peptidase 7)	Hs.180062	3.72	5.41
PSMC4	proteasome (prosome, macropain) 26S subunit, ATPase, 4	Hs.211594	5.50	2.91
PSMC5	proteasome (prosome, macropain) 26S subunit, ATPase, 5	Hs.79387	6.29	14.64
PSMC6	proteasome (prosome, macropain) 26S subunit, ATPase, 6	Hs.156171	78.13	18.49
PSMD1	proteasome (prosome, macropain) 26S subunit, non-ATPase, 1	Hs.3887	15.98	4.26
PSMD11	proteasome (prosome, macropain) 26S subunit, non-ATPase, 11	Hs.655396	7.14	5.59
PSMD12	proteasome (prosome, macropain) 26S subunit, non-ATPase, 12	Hs.592689	60.38	17.25
PSMD3	proteasome (prosome, macropain) 26S subunit, non-ATPase, 3	Hs.12970	4.37	71.69
PSMD6	proteasome (prosome, macropain) 26S subunit, non-ATPase, 6	Hs.152536	16.49	3.33
PSME1	proteasome (prosome, macropain) activator subunit 1 (PA28 alpha)	Hs.75348	3.10	14.00
PSME3	proteasome (prosome, macropain) activator subunit 3 (PA28 gamma; Ki)	Hs.152978	33.65	8.57
PSMF1	proteasome (prosome, macropain) inhibitor subunit 1 (PI31)	Hs.471917	5.52	5.68
PSMG2	proteasome (prosome, macropain) assembly chaperone 2	Hs.464652	20.37	5.11
PSPC1	paraspeckle component 1	Hs.721735	7.79	11.80
PSRC1	proline/serine-rich coiled-coil 1	Hs.405925	8.75	2.90
PTBP1	polypyrimidine tract binding protein 1	Hs.172550	12.22	13.53
PTGR1	prostaglandin reductase 1	Hs.584864	15.52	35.57
PTHLH	parathyroid hormone-like hormone	Hs.591159	11.32	18.10
PTK7	PTK7 protein tyrosine kinase 7	Hs.90572	4.84	14.34
PTMA	prothymosin, alpha	Hs.459927	4.10	6.27
PTP4A1	protein tyrosine phosphatase type IVA, member 1	Hs.227777	24.42	7.01
PTP4A3	protein tyrosine phosphatase type IVA, member 3	Hs.43666	3.62	3.00

PTPLA	protein tyrosine phosphatase-like (proline instead of catalytic arginine), member A	Hs.114062	10.43	2.51
PTPLB	protein tyrosine phosphatase-like (proline instead of catalytic arginine), member b	Hs.705480	9.69	8.43
PTPN1	protein tyrosine phosphatase, non-receptor type 1	Hs.417549	11.05	2.88
PTPN6	protein tyrosine phosphatase, non-receptor type 6	Hs.63489	4.32	6.15
PTPRK	protein tyrosine phosphatase, receptor type, K	Hs.155919	4.86	23.04
PTPRZ1	protein tyrosine phosphatase, receptor-type, Z polypeptide 1	Hs.489824	39.79	12.52
PTS	6-pyruvoyltetrahydropterin synthase	Hs.503860	38.02	2.68
PTTG1IP	pituitary tumor-transforming 1 interacting protein	Hs.474010	4.43	3.17
PUM2	pumilio homolog 2 (Drosophila)	Hs.467824	15.93	16.34
PURB	purine-rich element binding protein B	Hs.728785	23.23	3.49
PUS1	pseudouridylate synthase 1	Hs.592004	7.23	2.86
PUS3	pseudouridylate synthase 3	Hs.660922	16.78	4.41
PVR	poliovirus receptor	Hs.171844	5.60	18.52
PXMP2	peroxisomal membrane protein 2, 22 kDa	Hs.430299	2.66	5.24
PXN	paxillin	Hs.446336	6.86	16.68
PYCR2	pyrroline-5-carboxylate reductase family, member 2	Hs.654718	9.50	19.40
PYGL	phosphorylase, glycogen, liver	Hs.282417	18.12	3.52
PYGO2	pygopus homolog 2 (Drosophila)	Hs.533597	8.35	4.27
QKI	QKI, KH domain containing, RNA binding	Hs.510324	6.74	38.84
QPCTL	glutaminyl-peptide cyclotransferase-like	Hs.631556	4.24	7.47
QSOX2	quiescin Q6 sulfhydryl oxidase 2	Hs.657864	10.20	3.70
R3HCC1	R3H domain and coiled-coil containing 1	Hs.458644	3.20	2.92
R3HDM2	R3H domain containing 2	Hs.443673	10.14	2.63
RAB10	RAB10, member RAS oncogene family	Hs.467960	78.18	6.08
RAB12	RAB12, member RAS oncogene family	Hs.270074	7.37	5.41
RAB13	RAB13, member RAS oncogene family	Hs.151536	14.34	15.97
RAB1A	RAB1A, member RAS oncogene family	Hs.310645	45.89	4.03
RAB1B	RAB1B, member RAS oncogene family	Hs.300816	4.72	10.35
RAB20	RAB20, member RAS oncogene family	Hs.508720	6.89	8.94
RAB28	RAB28, member RAS oncogene family	Hs.656060	23.50	27.56
RAB2B	RAB2B, member RAS oncogene family	Hs.22399	10.18	3.66
RAB38	RAB38, member RAS oncogene family	Hs.591975	10.02	4.05
RAB3GAP1	RAB3 GTPase activating protein subunit 1 (catalytic)	Hs.306327	47.63	6.67
RAB4A	RAB4A, member RAS oncogene family	Hs.296169	9.46	10.92
RAB4B	RAB4B, member RAS oncogene family	Hs.631539	4.97	7.55
RAB5C	RAB5C, member RAS oncogene family	Hs.650382	12.67	62.04
RAB9A	RAB9A, member RAS oncogene family	Hs.495704	62.75	13.01
RABGAP1	RAB GTPase activating protein 1	Hs.271341	5.71	14.11
RABGAP1L	RAB GTPase activating protein 1-like	Hs.585378	21.36	39.54
RABL2A	RAB, member of RAS oncogene family-like 2A	Hs.446425	6.44	12.09
RAC1	ras-related C3 botulinum toxin substrate 1 (rho family, small GTP binding protein Rac1)	Hs.413812	39.43	4.60
RAD1	RAD1 homolog (S. pombe)	Hs.38114	15.19	11.49
RAD21	RAD21 homolog (S. pombe)	Hs.81848	97.44	4.18
RAD23B	RAD23 homolog B (S. cerevisiae)	Hs.521640	27.97	28.26
RAF1	v-raf-1 murine leukemia viral oncogene homolog 1	Hs.159130	22.73	17.88

RALB	v-ral simian leukemia viral oncogene homolog B (ras related; GTP binding protein)	Hs.469820	14.49	2.62
RALBP1	ralA binding protein 1	Hs.528993	11.23	3.95
RALGAPB	Ral GTPase activating protein, beta subunit (non-catalytic)	Hs.598850	18.89	8.80
RAP1B	RAP1B, member of RAS oncogene family	Hs.369920	48.37	3.81
RASA4	RAS p21 protein activator 4	Hs.696339	3.23	7.13
RASSF1	Ras association (RalGDS/AF-6) domain family member 1	Hs.476270	4.21	6.52
RASSF7	Ras association (RalGDS/AF-6) domain family (N-terminal) member 7	Hs.72925	2.54	4.27
RBBP5	retinoblastoma binding protein 5	Hs.519230	4.97	26.97
RBFOX2	RNA binding protein, fox-1 homolog (C. elegans) 2	Hs.282998	11.49	14.23
RBL1	retinoblastoma-like 1 (p107)	Hs.207745	4.12	6.50
RBL2	retinoblastoma-like 2 (p130)	Hs.513609	35.09	11.76
RBM17	RNA binding motif protein 17	Hs.498548	15.88	3.71
RBM22	RNA binding motif protein 22	Hs.713564	14.98	3.18
RBM23	RNA binding motif protein 23	Hs.4997	12.12	13.37
RBM3	RNA binding motif (RNP1, RRM) protein 3	Hs.301404	9.66	6.37
RBM38	RNA binding motif protein 38	Hs.236361	8.45	5.94
RBM39	RNA binding motif protein 39	Hs.282901	28.76	10.90
RBM4	RNA binding motif protein 4	Hs.533712	9.17	25.63
RBM4B	RNA binding motif protein 4B	Hs.618713	25.75	16.53
RBM8A	RNA binding motif protein 8A	Hs.591455	18.19	3.33
RBMS1	RNA binding motif, single stranded interacting protein 1	Hs.470412	51.61	5.09
RBMX	RNA binding motif protein, X-linked	Hs.380118	14.70	34.23
RBMXL1	RNA binding motif protein, X-linked-like 1	Hs.481898	37.22	23.73
RC3H2	ring finger and CCCH-type domains 2	Hs.709775	8.29	17.76
RCAN1	regulator of calcineurin 1	Hs.282326	30.15	4.32
RCAN3	RCAN family member 3	Hs.656799	12.32	6.60
RCC1	regulator of chromosome condensation 1	Hs.469723	4.82	7.51
RCE1	RCE1 homolog, prenyl protein peptidase (S. cerevisiae)	Hs.654972	5.50	6.00
RCL1	RNA terminal phosphate cyclase-like 1	Hs.194121	4.97	5.28
RCN1	reticulocalbin 1, EF-hand calcium binding domain	Hs.97887	31.95	5.12
RCN3	reticulocalbin 3, EF-hand calcium binding domain	Hs.567550	2.75	9.69
RDH10	retinol dehydrogenase 10 (all-trans)	Hs.244940	11.74	3.28
RDH11	retinol dehydrogenase 11 (all-trans/9-cis/11-cis)	Hs.719925	36.65	5.32
RECQL5	RecQ protein-like 5	Hs.632229	2.78	19.52
REEP5	receptor accessory protein 5	Hs.429608	97.00	3.25
RELA	v-rel reticuloendotheliosis viral oncogene homolog A (avian)	Hs.502875	4.73	3.44
REPIN1	replication initiator 1	Hs.647086	6.80	4.05
RETSAT	retinol saturase (all-trans-retinol 13, 14-reductase)	Hs.440401	3.68	38.50
RFC2	replication factor C (activator 1) 2, 40 kDa	Hs.647062	9.86	2.97
RFC4	replication factor C (activator 1) 4, 37 kDa	Hs.714318	18.58	3.62
RFNG	RFNG O-fucosylpeptide 3-beta-N-acetylglucosaminyltransferase	Hs.569700	4.77	7.60
RFT1	RFT1 homolog (S. cerevisiae)	Hs.631910	5.90	10.79
RFWD2	ring finger and WD repeat domain 2	Hs.523744	15.01	6.85
RFWD3	ring finger and WD repeat domain 3	Hs.567525	8.61	2.74
RGP1	RGP1 retrograde golgi transport homolog (S. cerevisiae)	Hs.493804	2.65	4.88
RGS12	regulator of G-protein signaling 12	Hs.527061	3.59	6.40
RGS14	regulator of G-protein signaling 14	Hs.9347	4.07	3.05

RGS2	regulator of G-protein signaling 2, 24 kDa	Hs.78944	85.28	11.97
RHOB	ras homolog gene family, member B	Hs.502876	6.00	4.67
RHOQ	ras homolog gene family, member Q	Hs.709193	18.89	24.88
RHOV	ras homolog gene family, member V	Hs.447901	5.49	3.16
RHPN2	rhophilin, Rho GTPase binding protein 2	Hs.692717	2.71	6.40
RIC8A	resistance to inhibitors of cholinesterase 8 homolog A (C. elegans)	Hs.592292	4.12	5.73
RIMKLB	ribosomal modification protein rimK-like family member B	Hs.504670	14.44	25.70
RING1	ring finger protein 1	Hs.631989	5.69	5.45
RIPK2	receptor-interacting serine-threonine kinase 2	Hs.103755	26.78	5.08
RNASE10	ribonuclease, RNase A family, 10 (non-active)	Hs.451057	4.18	3.71
RNASE4	ribonuclease, RNase A family, 4	Hs.593708	27.82	35.56
RNASEH1	ribonuclease H1	Hs.568006	23.13	11.80
RNASEH2B	ribonuclease H2, subunit B	Hs.306291	49.55	12.84
RNF115	ring finger protein 115	Hs.523550	10.00	14.55
RNF121	ring finger protein 121	Hs.368554	7.33	2.88
RNF13	ring finger protein 13	Hs.12333	86.97	7.60
RNF139	ring finger protein 139	Hs.730771	47.73	2.51
RNF167	ring finger protein 167	Hs.7158	5.33	8.25
RNF2	ring finger protein 2	Hs.591490	14.76	6.78
RNF212	ring finger protein 212	Hs.248290	14.53	24.71
RNF217	ring finger protein 217	Hs.368639	7.85	5.36
RNF220	ring finger protein 220	Hs.456557	3.82	2.54
RNF223	ring finger protein 223	Hs.568137	3.72	4.88
RNF25	ring finger protein 25	Hs.471403	3.55	4.91
RNF40	ring finger protein 40	Hs.65238	2.62	2.57
RNF44	ring finger protein 44	Hs.434888	8.62	2.74
RNF7	ring finger protein 7	Hs.134623	17.68	17.69
RNPC3	RNA-binding region (RNP1, RRM) containing 3	Hs.632423	8.68	9.20
RNPS1	RNA binding protein S1, serine-rich domain	Hs.355643	20.24	7.20
RP9	retinitis pigmentosa 9 (autosomal dominant)	Hs.326805	21.97	5.76
RPA2	replication protein A2, 32 kDa	Hs.79411	18.04	2.68
RPAIN	RPA interacting protein	Hs.462086	17.57	8.10
RPE	ribulose-5-phosphate-3-epimerase	Hs.282260	8.21	8.23
RPF1	ribosome production factor 1 homolog (S. cerevisiae)	Hs.481202	7.50	16.25
RPIA	ribose 5-phosphate isomerase A	Hs.469264	19.07	3.63
RPL15	ribosomal protein L15	Hs.381219	12.86	4.75
RPL31	ribosomal protein L31	Hs.469473	4.51	7.49
RPL36A-HNRNPH2	RPL36A-HNRNPH2 readthrough	Hs.432485	5.03	30.03
RPL37	ribosomal protein L37	Hs.80545	3.74	5.56
RPL3L	ribosomal protein L3-like	Hs.657266	2.51	4.89
RPL4	ribosomal protein L4	Hs.644628	6.88	2.65
RPL5	ribosomal protein L5	Hs.532359	12.52	5.36
RPL7L1	ribosomal protein L7-like 1	Hs.520133	8.07	6.94
RPN1	ribophorin I	Hs.518244	5.27	6.72
RPP38	ribonuclease P/MRP 38 kDa subunit	Hs.94986	13.97	4.09
RPS6	ribosomal protein S6	Hs.408073	2.89	28.44
RPS6KA1	ribosomal protein S6 kinase, 90 kDa, polypeptide 1	Hs.149957	3.23	2.66

RPUSD4	RNA pseudouridylate synthase domain containing 4	Hs.150458	6.44	4.54
RRAGC	Ras-related GTP binding C	Hs.532461	15.92	4.92
RRAGD	Ras-related GTP binding D	Hs.31712	11.08	8.14
RREB1	ras responsive element binding protein 1	Hs.298248	3.83	10.95
RRN3	RRN3 RNA polymerase I transcription factor homolog (S. cerevisiae)	Hs.460078	13.23	97.53
RRP1B	ribosomal RNA processing 1 homolog B (S. cerevisiae)	Hs.565725	13.83	2.86
RRP7A	ribosomal RNA processing 7 homolog A (S. cerevisiae)	Hs.730765	3.01	24.07
RRS1	RRS1 ribosome biogenesis regulator homolog (S. cerevisiae)	Hs.71827	9.79	2.90
RSBN1	round spermatid basic protein 1	Hs.486285	11.40	4.47
RSG1	REM2 and RAB-like small GTPase 1	Hs.546430	3.37	3.94
RSL24D1	ribosomal L24 domain containing 1	Hs.274772	134.89	22.61
RTN4RL2	reticulon 4 receptor-like 2	Hs.502618	4.52	13.16
RUFY1	RUN and FYVE domain containing 1	Hs.306769	35.90	10.50
RUNDC1	RUN domain containing 1	Hs.632255	5.82	3.42
RUNX1	runt-related transcription factor 1	Hs.149261	5.40	6.71
RUSC1-AS1	RUSC1 antisense RNA 1 (non-protein coding)	Hs.731131	3.47	15.70
RUVBL1	RuvB-like 1 (E. coli)	Hs.272822	11.42	29.09
RWDD1	RWD domain containing 1	Hs.532164	44.84	7.03
RWDD2B	RWD domain containing 2B	Hs.34136	19.71	8.42
RXRB	retinoid X receptor, beta	Hs.388034	5.39	5.15
S100A11	S100 calcium binding protein A11	Hs.417004	8.98	5.43
S100A14	S100 calcium binding protein A14	Hs.288998	12.34	5.80
S1PR5	sphingosine-1-phosphate receptor 5	Hs.501561	6.94	15.62
SAA1	serum amyloid A1	Hs.632144	3.40	4.65
SAAL1	serum amyloid A-like 1	Hs.591998	15.82	4.34
SAFB2	scaffold attachment factor B2	Hs.655392	4.84	6.28
SAMD10	sterile alpha motif domain containing 10	Hs.27189	20.32	2.69
SAMD8	sterile alpha motif domain containing 8	Hs.727615	13.30	2.95
SAMHD1	SAM domain and HD domain 1	Hs.580681	4.18	8.39
SAP30	Sin3A-associated protein, 30 kDa	Hs.591715	33.42	3.31
SAP30BP	SAP30 binding protein	Hs.655088	5.36	3.99
SAP30L	SAP30-like	Hs.592566	15.33	5.81
SAR1A	SAR1 homolog A (S. cerevisiae)	Hs.499960	9.16	2.75
SAR1B	SAR1 homolog B (S. cerevisiae)	Hs.432984	60.40	3.65
SARS	seryl-tRNA synthetase	Hs.531176	10.12	7.36
SART1	squamous cell carcinoma antigen recognized by T cells	Hs.502883	3.27	3.17
SART3	squamous cell carcinoma antigen recognized by T cells 3	Hs.584842	6.19	7.08
SAT1	spermidine/spermine N1-acetyltransferase 1	Hs.28491	8.16	11.14
SBDS	Shwachman-Bodian-Diamond syndrome	Hs.110445	16.18	14.81
SCAMP2	secretory carrier membrane protein 2	Hs.458917	5.64	21.61
SCAMP4	secretory carrier membrane protein 4	Hs.144980	10.26	21.48
SCARB2	scavenger receptor class B, member 2	Hs.349656	92.89	4.35
SCD	stearoyl-CoA desaturase (delta-9-desaturase)	Hs.558396	3.95	14.27
SCD5	stearoyl-CoA desaturase 5	Hs.379191	6.42	73.87
SCFD1	sec1 family domain containing 1	Hs.369168	185.91	15.53
SCG2	secretogranin II	Hs.516726	75.17	44.42
SCLY	selenocysteine lyase	Hs.471785	7.45	4.36

SCO1	SCO cytochrome oxidase deficient homolog 1 (yeast)	Hs.14511	18.24	3.93
SCPEP1	serine carboxypeptidase 1	Hs.514950	9.25	14.65
SDC4	syndecan 4	Hs.632267	6.65	2.85
SDCBP	syndecan binding protein (syntenin)	Hs.200804	54.60	14.48
SDF2	stromal cell-derived factor 2	Hs.514036	7.76	9.96
SDF4	stromal cell derived factor 4	Hs.42806	6.74	27.14
SDHAF2	succinate dehydrogenase complex assembly factor 2	Hs.313247	10.61	2.63
SDHB	succinate dehydrogenase complex, subunit B, iron sulfur (Ip)	Hs.465924	21.36	6.24
SDHC	succinate dehydrogenase complex, subunit C, integral membrane protein, 15 kDa	Hs.444472	18.73	7.27
SDHD	succinate dehydrogenase complex, subunit D, integral membrane protein	Hs.647227	38.05	13.62
SDR39U1	short chain dehydrogenase/reductase family 39U, member 1	Hs.643552	7.05	2.99
SEC13	SEC13 homolog (S. cerevisiae)	Hs.166924	2.67	7.20
SEC22A	SEC22 vesicle trafficking protein homolog A (S. cerevisiae)	Hs.477361	13.97	2.50
SEC22B	SEC22 vesicle trafficking protein homolog B (S. cerevisiae) (gene/pseudogene)	Hs.632438	12.15	4.33
SEC22C	SEC22 vesicle trafficking protein homolog C (S. cerevisiae)	Hs.445892	29.22	15.08
SEC23A	Sec23 homolog A (S. cerevisiae)	Hs.272927	68.84	23.08
SEC23B	Sec23 homolog B (S. cerevisiae)	Hs.369373	29.36	24.41
SEC31A	SEC31 homolog A (S. cerevisiae)	Hs.370024	20.29	8.30
SEC61A1	Sec61 alpha 1 subunit (S. cerevisiae)	Hs.518236	4.17	5.60
SEC61G	Sec61 gamma subunit	Hs.488282	7.46	5.23
SEH1L	SEH1-like (S. cerevisiae)	Hs.301048	53.18	23.42
SEL1L3	sel-1 suppressor of lin-12-like 3 (C. elegans)	Hs.479384	24.85	4.36
SELT	selenoprotein T	Hs.369052	76.08	25.54
SEMA4A	sema domain, immunoglobulin domain (Ig), transmembrane domain (TM) and short cytoplasmic domain, (semaphorin) 4A	Hs.408846	11.02	12.12
SEMA4B	sema domain, immunoglobulin domain (Ig), transmembrane domain (TM) and short cytoplasmic domain, (semaphorin) 4B	Hs.474935	7.73	47.62
SEMA6B	sema domain, transmembrane domain (TM), and cytoplasmic domain, (semaphorin) 6B	Hs.465642	3.28	5.61
SENP2	SUMO1/sentrin/SMT3 specific peptidase 2	Hs.401388	41.51	5.58
SENP3	SUMO1/sentrin/SMT3 specific peptidase 3	Hs.513926	13.93	8.94
SEPN1	selenoprotein N, 1	Hs.323396	3.34	13.31
SERBP1	SERPINE1 mRNA binding protein 1	Hs.730604	7.17	11.31
SERF1B	small EDRK-rich factor 1B (centromeric)	Hs.559428	11.89	5.48
SERF2	small EDRK-rich factor 2	Hs.424126	4.95	2.69
SERINC2	serine incorporator 2	Hs.270655	3.19	17.93
SERINC3	serine incorporator 3	Hs.272168	14.95	5.71
SERP1	stress-associated endoplasmic reticulum protein 1	Hs.518326	10.32	10.83
SERPINB6	serpin peptidase inhibitor, clade B (ovalbumin), member 6	Hs.519523	3.64	2.51
SERPINE2	serpin peptidase inhibitor, clade E (nexin, plasminogen activator inhibitor type 1), member 2	Hs.38449	13.70	7.38
SERTAD3	SERTA domain containing 3	Hs.515412	3.94	6.71
SESN2	sestrin 2	Hs.469543	4.33	5.69
SESN3	sestrin 3	Hs.659934	18.67	10.90
SET	SET nuclear oncogene	Hs.436687	13.01	10.77
SETD3	SET domain containing 3	Hs.510407	9.42	29.57

SETD5	SET domain containing 5	Hs.288164	5.46	13.00
SETD8	SET domain containing (lysine methyltransferase) 8	Hs.443735	9.47	5.28
SF1	splicing factor 1	Hs.502829	2.75	11.35
SF3B1	splicing factor 3b, subunit 1, 155 kDa	Hs.632554	2.85	25.80
SFT2D1	SFT2 domain containing 1	Hs.487143	12.16	5.36
SFT2D2	SFT2 domain containing 2	Hs.645435	3.98	18.79
SFT2D3	SFT2 domain containing 3	Hs.345849	23.44	3.56
SGCE	sarcoglycan, epsilon	Hs.371199	91.86	5.19
SH2B1	SH2B adaptor protein 1	Hs.15744	3.40	3.88
SH3BP1	SH3-domain binding protein 1	Hs.601143	2.93	19.80
SH3GLB2	SH3-domain GRB2-like endophilin B2	Hs.460238	4.64	10.35
SH3RF1	SH3 domain containing ring finger 1	Hs.301804	7.98	2.69
SH3RF2	SH3 domain containing ring finger 2	Hs.443728	4.29	9.97
SH3YL1	SH3 domain containing, Ysc84-like 1 (S. cerevisiae)	Hs.730730	81.24	4.85
SHB	Src homology 2 domain containing adaptor protein B	Hs.521482	4.14	2.79
SHC1	SHC (Src homology 2 domain containing) transforming protein 1	Hs.433795	6.17	7.24
SHFM1	split hand/foot malformation (ectrodactyly) type 1	Hs.489201	22.81	4.15
SIAH1	seven in absentia homolog 1 (Drosophila)	Hs.713615	12.44	8.57
SIKE1	suppressor of IKBKE 1	Hs.709277	27.28	6.14
SIRPA	signal-regulatory protein alpha	Hs.581021	5.45	3.84
SIRT2	sirtuin 2	Hs.466693	7.83	11.66
SIRT5	sirtuin 5	Hs.567431	5.26	6.65
SIRT6	sirtuin 6	Hs.423756	6.28	2.52
SKP1	S-phase kinase-associated protein 1	Hs.171626	8.17	3.79
SKP2	S-phase kinase-associated protein 2 (p45)	Hs.23348	22.00	10.52
SLC11A2	solute carrier family 11 (proton-coupled divalent metal ion transporters), member 2	Hs.505545	7.39	5.54
SLC12A6	solute carrier family 12 (potassium/chloride transporters), member 6	Hs.510939	14.44	23.74
SLC12A8	solute carrier family 12 (potassium/chloride transporters), member 8	Hs.658514	7.36	14.51
SLC13A2	solute carrier family 13 (sodium-dependent dicarboxylate transporter), member 2	Hs.102307	4.37	3.54
SLC15A4	solute carrier family 15, member 4	Hs.728188	5.16	4.32
SLC16A1	solute carrier family 16, member 1 (monocarboxylic acid transporter 1)	Hs.75231	24.95	3.48
SLC16A10	solute carrier family 16, member 10 (aromatic amino acid transporter)	Hs.591327	6.96	3.68
SLC16A11	solute carrier family 16, member 11 (monocarboxylic acid transporter 11)	Hs.336564	3.59	3.09
SLC16A9	solute carrier family 16, member 9 (monocarboxylic acid transporter 9)	Hs.499709	64.14	5.82
SLC17A5	solute carrier family 17 (anion/sugar transporter), member 5	Hs.597422	15.08	3.15
SLC1A3	solute carrier family 1 (glial high affinity glutamate transporter), member 3	Hs.481918	19.90	18.12
SLC1A5	solute carrier family 1 (neutral amino acid transporter), member 5	Hs.631582	3.20	5.87
SLC20A2	solute carrier family 20 (phosphate transporter), member 2	Hs.653173	5.74	5.29
SLC23A2	solute carrier family 23 (nucleobase transporters), member 2	Hs.516866	10.90	6.42
SLC25A10	solute carrier family 25 (mitochondrial carrier; dicarboxylate transporter), member 10	Hs.548187	2.66	3.39
SLC25A11	solute carrier family 25 (mitochondrial carrier; oxoglutarate carrier), member 11	Hs.184877	3.04	3.48
SLC25A28	solute carrier family 25, member 28	Hs.403790	7.30	4.21
SLC25A33	solute carrier family 25, member 33	Hs.568613	7.11	3.16

SLC25A36	solute carrier family 25, member 36	Hs.144130	63.37	9.65
SLC25A42	solute carrier family 25, member 42	Hs.303669	16.16	4.95
SLC27A2	solute carrier family 27 (fatty acid transporter), member 2	Hs.11729	15.04	5.11
SLC29A2	solute carrier family 29 (nucleoside transporters), member 2	Hs.569017	10.17	18.37
SLC30A5	solute carrier family 30 (zinc transporter), member 5	Hs.631975	8.17	18.86
SLC30A7	solute carrier family 30 (zinc transporter), member 7	Hs.533903	12.03	8.09
SLC31A1	solute carrier family 31 (copper transporters), member 1	Hs.532315	10.96	12.58
SLC33A1	solute carrier family 33 (acetyl-CoA transporter), member 1	Hs.478031	12.35	5.84
SLC35A1	solute carrier family 35 (CMP-sialic acid transporter), member A1	Hs.423163	33.17	17.19
SLC35A2	solute carrier family 35 (UDP-galactose transporter), member A2	Hs.21899	7.31	17.99
SLC35A4	solute carrier family 35, member A4	Hs.406840	3.15	20.78
SLC35B1	solute carrier family 35, member B1	Hs.154073	13.54	2.90
SLC35B2	solute carrier family 35, member B2	Hs.182885	5.30	4.54
SLC35D2	solute carrier family 35, member D2	Hs.494556	21.79	14.71
SLC35F2	solute carrier family 35, member F2	Hs.524014	8.77	4.35
SLC35F5	solute carrier family 35, member F5	Hs.632527	24.57	25.96
SLC37A1	solute carrier family 37 (glycerol-3-phosphate transporter), member 1	Hs.547009	6.38	10.07
SLC37A2	solute carrier family 37 (glycerol-3-phosphate transporter), member 2	Hs.352661	3.55	8.05
SLC38A2	solute carrier family 38, member 2	Hs.221847	47.62	17.18
SLC38A6	solute carrier family 38, member 6	Hs.200738	20.03	6.21
SLC38A9	solute carrier family 38, member 9	Hs.649685	39.62	4.23
SLC39A1	solute carrier family 39 (zinc transporter), member 1	Hs.730664	8.45	19.57
SLC39A14	solute carrier family 39 (zinc transporter), member 14	Hs.491232	3.49	6.56
SLC39A7	solute carrier family 39 (zinc transporter), member 7	Hs.631995	6.87	9.48
SLC39A9	solute carrier family 39 (zinc transporter), member 9	Hs.432690	5.61	6.06
SLC3A2	solute carrier family 3 (activators of dibasic and neutral amino acid transport), member 2	Hs.502769	5.11	5.46
SLC44A1	solute carrier family 44, member 1	Hs.573495	15.51	6.40
SLC47A1	solute carrier family 47, member 1	Hs.232054	4.57	4.36
SLC4A1AP	solute carrier family 4 (anion exchanger), member 1, adaptor protein	Hs.306000	36.87	6.34
SLC4A2	solute carrier family 4, anion exchanger, member 2 (erythrocyte membrane protein band 3-like 1)	Hs.647069	3.70	6.55
SLC50A1	solute carrier family 50 (sugar transporter), member 1	Hs.292154	9.13	5.93
SLC5A2	solute carrier family 5 (sodium/glucose cotransporter), member 2	Hs.709195	2.51	37.91
SLC5A6	solute carrier family 5 (sodium-dependent vitamin transporter), member 6	Hs.435735	3.55	6.31
SLC6A15	solute carrier family 6 (neutral amino acid transporter), member 15	Hs.44424	8.78	19.39
SLC6A9	solute carrier family 6 (neurotransmitter transporter, glycine), member 9	Hs.442590	5.16	3.57
SLC7A11	solute carrier family 7 (anionic amino acid transporter light chain, xc- system), member 11	Hs.390594	90.14	3.85
SLC9A6	solute carrier family 9 (sodium/hydrogen exchanger), member 6	Hs.62185	48.47	4.99
SLCO2A1	solute carrier organic anion transporter family, member 2A1	Hs.518270	12.00	2.96
SLCO3A1	solute carrier organic anion transporter family, member 3A1	Hs.311187	10.05	9.96
SLITRK5	SLIT and NTRK-like family, member 5	Hs.591208	8.81	5.07
SLITRK6	SLIT and NTRK-like family, member 6	Hs.525105	44.28	16.60
SMAGP	small cell adhesion glycoprotein	Hs.652389	12.35	7.45
SMARCA2	SWI/SNF related, matrix associated, actin dependent regulator of chromatin, subfamily a, member 2	Hs.298990	4.13	16.95

SMARCC1	SWI/SNF related, matrix associated, actin dependent regulator of chromatin, subfamily c, member 1	Hs.476179	10.99	7.17
SMARCD2	SWI/SNF related, matrix associated, actin dependent regulator of chromatin, subfamily d, member 2	Hs.250581	6.33	3.26
SMARCE1	SWI/SNF related, matrix associated, actin dependent regulator of chromatin, subfamily e, member 1	Hs.730637	26.31	13.12
SMC1A	structural maintenance of chromosomes 1A	Hs.211602	4.89	12.40
SMEK1	SMEK homolog 1, suppressor of mek1 (Dictyostelium)	Hs.533887	18.23	4.06
SMG1	smg-1 homolog, phosphatidylinositol 3-kinase-related kinase (C. elegans)	Hs.460179	3.75	7.90
SMG6	smg-6 homolog, nonsense mediated mRNA decay factor (C. elegans)	Hs.448342	4.86	4.00
SMG8	smg-8 homolog, nonsense mediated mRNA decay factor (C. elegans)	Hs.7296	11.88	3.53
SMG9	smg-9 homolog, nonsense mediated mRNA decay factor (C. elegans)	Hs.466875	5.50	6.30
SMN1	survival of motor neuron 1, telomeric	Hs.535788	48.42	3.91
SMNDC1	survival motor neuron domain containing 1	Hs.632093	30.99	3.90
SMOX	spermine oxidase	Hs.433337	11.71	3.23
SMPD1	sphingomyelin phosphodiesterase 1, acid lysosomal	Hs.498173	4.04	4.92
SMPDL3B	sphingomyelin phosphodiesterase, acid-like 3B	Hs.123659	4.67	3.22
SMS	spermine synthase	Hs.727552	17.39	14.54
SMU1	smu-1 suppressor of mec-8 and unc-52 homolog (C. elegans)	Hs.655351	4.45	6.50
SMUG1	single-strand-selective monofunctional uracil-DNA glycosylase 1	Hs.632721	3.00	3.99
SNAI1	snail homolog 1 (Drosophila)	Hs.48029	6.65	5.45
SNAPIN	SNAP-associated protein	Hs.32018	12.22	4.11
SNHG5	small nucleolar RNA host gene 5 (non-protein coding)	Hs.292457	14.84	3.27
SNN	stannin	Hs.618526	12.03	3.54
SNORA40	small nucleolar RNA, H/ACA box 40	Hs.625539	3.17	19.05
SNORA71B	small nucleolar RNA, H/ACA box 71B	Hs.400876	3.63	4.12
SNORD33	small nucleolar RNA, C/D box 33	Hs.523185	2.75	493.28
SNORD80	small nucleolar RNA, C/D box 80	Hs.531856	4.75	47.57
SNORD86	small nucleolar RNA, C/D box 86	Hs.376064	3.92	9.36
SNRNP27	small nuclear ribonucleoprotein 27 kDa (U4/U6.U5)	Hs.54649	29.34	4.00
SNRNP35	small nuclear ribonucleoprotein 35 kDa (U11/U12)	Hs.632738	7.41	5.50
SNRNP40	small nuclear ribonucleoprotein 40 kDa (U5)	Hs.33962	4.71	14.27
SNRNP70	small nuclear ribonucleoprotein 70 kDa (U1)	Hs.467097	4.48	6.78
SNRPA	small nuclear ribonucleoprotein polypeptide A	Hs.466775	7.04	3.12
SNRPA1	small nuclear ribonucleoprotein polypeptide A'	Hs.528763	22.49	13.62
SNRPB2	small nuclear ribonucleoprotein polypeptide B	Hs.280378	47.40	19.84
SNRPC	small nuclear ribonucleoprotein polypeptide C	Hs.1063	9.87	17.88
SNRPD1	small nuclear ribonucleoprotein D1 polypeptide 16 kDa	Hs.464734	17.27	2.95
SNRPF	small nuclear ribonucleoprotein polypeptide F	Hs.105465	27.21	5.88
SNRPN	small nuclear ribonucleoprotein polypeptide N	Hs.564847	7.89	5.51
SNURF	SNRPN upstream reading frame	Hs.564847	6.77	3.18
SNW1	SNW domain containing 1	Hs.445498	57.87	6.16
SNX11	sorting nexin 11	Hs.15827	6.19	3.57
SNX14	sorting nexin 14	Hs.485871	36.81	7.55
SNX15	sorting nexin 15	Hs.80132	12.31	9.57
SNX18	sorting nexin 18	Hs.432755	5.26	5.49
SNX19	sorting nexin 19	Hs.444024	21.74	5.52

SNX2	sorting nexin 2	Hs.713554	120.50	13.64
SNX22	sorting nexin 22	Hs.708268	2.91	48.83
SNX24	sorting nexin 24	Hs.483200	7.18	11.50
SNX27	sorting nexin family member 27	Hs.192326	7.60	3.80
SNX5	sorting nexin 5	Hs.316890	36.35	2.95
SNX6	sorting nexin 6	Hs.356647	97.41	7.75
SNX7	sorting nexin 7	Hs.197015	23.71	3.77
SOCS3	suppressor of cytokine signaling 3	Hs.527973	4.88	2.68
SON	SON DNA binding protein	Hs.517262	12.00	29.34
SORT1	sortilin 1	Hs.485195	32.80	4.75
SPAG7	sperm associated antigen 7	Hs.90436	7.04	8.99
SPATS2L	spermatogenesis associated, serine-rich 2-like	Hs.120323	39.09	8.83
SPCS1	signal peptidase complex subunit 1 homolog (S. cerevisiae)	Hs.11125	13.82	4.47
SPECC1	sperm antigen with calponin homology and coiled-coil domains 1	Hs.431045	4.63	6.24
SPG7	spastic paraplegia 7 (pure and complicated autosomal recessive)	Hs.185597	3.99	14.69
SPIN1	spindlin 1	Hs.146804	28.73	22.71
SPINK2	serine peptidase inhibitor, Kazal type 2 (acrosin-trypsin inhibitor)	Hs.98243	20.30	3.81
SPOP	speckle-type POZ protein	Hs.730639	26.18	6.48
SPP1	secreted phosphoprotein 1	Hs.313	13.40	9.60
SPPL2A	signal peptide peptidase-like 2A	Hs.401537	27.86	7.06
SPPL3	signal peptide peptidase-like 3	Hs.507087	6.66	5.16
SPRYD3	SPRY domain containing 3	Hs.343334	5.28	4.30
SPTLC1	serine palmitoyltransferase, long chain base subunit 1	Hs.90458	31.46	22.18
SPTLC2	serine palmitoyltransferase, long chain base subunit 2	Hs.435661	11.39	7.90
SRC	v-src sarcoma (Schmidt-Ruppin A-2) viral oncogene homolog (avian)	Hs.195659	5.64	5.44
SRD5A1	steroid-5-alpha-reductase, alpha polypeptide 1 (3-oxo-5 alpha-steroid delta 4-dehydrogenase alpha 1)	Hs.552	17.22	4.14
SRP19	signal recognition particle 19 kDa	Hs.637001	4.78	62.24
SRP68	signal recognition particle 68 kDa	Hs.514495	17.23	24.21
SRP9	signal recognition particle 9 kDa	Hs.511425	75.39	33.56
SRPK1	SRSF protein kinase 1	Hs.443861	55.75	4.25
SRPR	signal recognition particle receptor (docking protein)	Hs.368376	9.39	3.03
SRRD	SRR1 domain containing	Hs.709914	4.08	3.49
SRRM1	serine/arginine repetitive matrix 1	Hs.18192	7.80	3.89
SRSF11	serine/arginine-rich splicing factor 11	Hs.479693	20.88	18.62
SRSF2	serine/arginine-rich splicing factor 2	Hs.584801	14.23	5.66
SRSF5	serine/arginine-rich splicing factor 5	Hs.632326	15.05	3.25
SRSF6	serine/arginine-rich splicing factor 6	Hs.6891	11.77	12.52
SRSF7	serine/arginine-rich splicing factor 7	Hs.309090	17.46	8.46
SRSF9	serine/arginine-rich splicing factor 9	Hs.706889	9.74	15.43
SRXN1	sulfiredoxin 1	Hs.516830	14.47	2.88
SSBP1	single-stranded DNA binding protein 1	Hs.490394	20.21	6.88
SSBP2	single-stranded DNA binding protein 2	Hs.102735	17.07	4.04
SSH1	slingshot homolog 1 (Drosophila)	Hs.199763	5.41	6.01
SSH3	slingshot homolog 3 (Drosophila)	Hs.29173	4.29	8.23
SSR1	signal sequence receptor, alpha	Hs.114033	45.01	5.28
SSR2	signal sequence receptor, beta (translocon-associated protein beta)	Hs.74564	5.03	2.67

SSR3	signal sequence receptor, gamma (translocon-associated protein gamma)	Hs.518346	24.22	39.54
ST3GAL3	ST3 beta-galactoside alpha-2, 3-sialyltransferase 3	Hs.597915	6.99	7.00
STAG3L2	stromal antigen 3-like 2	Hs.632310	8.57	3.15
STAG3L4	stromal antigen 3-like 4	Hs.632013	8.52	5.36
STAM	signal transducing adaptor molecule (SH3 domain and ITAM motif) 1	Hs.335391	5.58	11.68
STARD3NL	STARD3 N-terminal like	Hs.728820	22.79	5.44
STAT3	signal transducer and activator of transcription 3 (acute-phase response factor)	Hs.463059	5.18	16.78
STAU2	staufen, RNA binding protein, homolog 2 (Drosophila)	Hs.561815	45.94	16.18
STC2	stanniocalcin 2	Hs.233160	27.79	7.81
STEAP1	six transmembrane epithelial antigen of the prostate 1	Hs.61635	48.47	6.27
STEAP1B	STEAP family member 1B	Hs.729825	31.75	4.86
STEAP3	STEAP family member 3, metalloreductase	Hs.647822	2.81	3.11
STIP1	stress-induced-phosphoprotein 1	Hs.337295	6.69	37.97
STK24	serine/threonine kinase 24	Hs.508514	8.51	11.08
STMN3	stathmin-like 3	Hs.639609	4.72	9.62
STOM	stomatin	Hs.253903	41.82	9.38
STOML2	stomatin (EPB72)-like 2	Hs.3439	7.18	6.93
STRADA	STE20-related kinase adaptor alpha	Hs.514402	7.59	7.36
STT3A	STT3, subunit of the oligosaccharyltransferase complex, homolog A (S. cerevisiae)	Hs.504237	3.72	8.51
STT3B	STT3, subunit of the oligosaccharyltransferase complex, homolog B (S. cerevisiae)	Hs.475812	31.52	56.28
STX10	syntaxin 10	Hs.43812	5.92	3.42
STX16	syntaxin 16	Hs.307913	10.42	7.00
STX18	syntaxin 18	Hs.584913	8.20	11.83
STX1A	syntaxin 1A (brain)	Hs.647024	5.53	10.04
STXBP2	syntaxin binding protein 2	Hs.515104	4.29	10.98
SUB1	SUB1 homolog (S. cerevisiae)	Hs.229641	34.26	23.05
SUCLG1	succinate-CoA ligase, alpha subunit	Hs.270428	12.01	3.33
SUDS3	suppressor of defective silencing 3 homolog (S. cerevisiae)	Hs.416630	15.16	5.33
SUGT1	SGT1, suppressor of G2 allele of SKP1 (S. cerevisiae)	Hs.281902	9.98	24.80
SULF1	sulfatase 1	Hs.409602	53.93	3.98
SULT1A4	sulfotransferase family, cytosolic, 1A, phenol-preferring, member 4	Hs.730631	5.27	2.89
SUMF2	sulfatase modifying factor 2	Hs.279696	5.02	4.83
SUMO3	SMT3 suppressor of mif two 3 homolog 3 (S. cerevisiae)	Hs.474005	14.90	2.57
SUN2	Sad1 and UNC84 domain containing 2	Hs.517622	2.57	22.36
SUPT3H	suppressor of Ty 3 homolog (S. cerevisiae)	Hs.368325	8.12	17.39
SUPT7L	suppressor of Ty 7 (S. cerevisiae)-like	Hs.6232	7.49	4.11
SUPV3L1	suppressor of var1, 3-like 1 (S. cerevisiae)	Hs.106469	6.49	4.06
SURF4	surfeit 4	Hs.512465	4.22	3.04
SUV39H1	suppressor of variegation 3-9 homolog 1 (Drosophila)	Hs.522639	2.79	3.21
SV2A	synaptic vesicle glycoprotein 2A	Hs.516153	5.88	11.32
SYAP1	synapse associated protein 1	Hs.489336	19.72	2.92
SYBU	syntabulin (syntaxin-interacting)	Hs.390738	14.46	8.71
SYNCRIP	synaptotagmin binding, cytoplasmic RNA interacting protein	Hs.571177	43.48	22.86
SYNE2	spectrin repeat containing, nuclear envelope 2	Hs.729020	3.40	2.92

SYNGR3	synaptogyrin 3	Hs.435277	4.46	3.23
SYNJ2BP	synaptojanin 2 binding protein	Hs.443661	17.45	3.11
SYPL1	synaptophysin-like 1	Hs.80919	53.80	11.48
SYT17	synaptotagmin XVII	Hs.258326	3.64	3.10
TAB2	TGF-beta activated kinase 1/MAP3K7 binding protein 2	Hs.269775	40.73	6.39
TACO1	translational activator of mitochondrially encoded cytochrome c oxidase I	Hs.174134	4.55	6.13
TADA1	transcriptional adaptor 1	Hs.435967	15.57	5.97
TAF11	TAF11 RNA polymerase II, TATA box binding protein (TBP)-associated factor, 28 kDa	Hs.112444	18.40	5.04
TAF15	TAF15 RNA polymerase II, TATA box binding protein (TBP)-associated factor, 68 kDa	Hs.402752	18.27	10.56
TAF3	TAF3 RNA polymerase II, TATA box binding protein (TBP)-associated factor, 140 kDa	Hs.527688	6.42	5.05
TAF9	TAF9 RNA polymerase II, TATA box binding protein (TBP)-associated factor, 32 kDa	Hs.653163	175.46	40.08
TAGLN2	transgelin 2	Hs.517168	7.26	8.96
TAPBPL	TAP binding protein-like	Hs.504597	3.69	3.76
TARDBP	TAR DNA binding protein	Hs.300624	19.63	15.07
TARS	threonyl-tRNA synthetase	Hs.481860	40.29	4.66
TAX1BP1	Tax1 (human T-cell leukemia virus type I) binding protein 1	Hs.34576	67.68	10.88
TAX1BP3	Tax1 (human T-cell leukemia virus type I) binding protein 3	Hs.12956	10.05	17.22
TAZ	tafazzin	Hs.409911	2.81	4.47
TBC1D17	TBC1 domain family, member 17	Hs.631587	3.05	3.42
TBC1D22A	TBC1 domain family, member 22A	Hs.435044	3.17	6.92
TBC1D22B	TBC1 domain family, member 22B	Hs.485270	6.06	5.08
TBC1D24	TBC1 domain family, member 24	Hs.353087	3.91	4.84
TBC1D7	TBC1 domain family, member 7	Hs.484678	8.33	5.16
TBCB	tubulin folding cofactor B	Hs.31053	2.96	16.65
TBCC	tubulin folding cofactor C	Hs.75064	8.02	3.77
TBL1X	transducin (beta)-like 1X-linked	Hs.495656	8.99	6.10
TBL1XR1	transducin (beta)-like 1 X-linked receptor 1	Hs.715026	32.13	37.88
TBPL1	TBP-like 1	Hs.486507	15.20	3.99
TCEA1	transcription elongation factor A (SII), 1	Hs.491745	27.14	18.61
TCEA3	transcription elongation factor A (SII), 3	Hs.446354	3.83	7.72
TCEAL4	transcription elongation factor A (SII)-like 4	Hs.194329	59.13	6.55
TCEAL8	transcription elongation factor A (SII)-like 8	Hs.389734	22.20	9.64
TCEB3	transcription elongation factor B (SIII), polypeptide 3 (110 kDa, elongin A)	Hs.15535	14.04	9.76
TCF20	transcription factor 20 (AR1)	Hs.475018	3.11	10.07
TCF7L2	transcription factor 7-like 2 (T-cell specific, HMG-box)	Hs.593995	3.05	10.34
TCHH	trichohyalin	Hs.432416	3.70	5.28
TCP1	t-complex 1	Hs.363137	8.97	24.32
TDG	thymine-DNA glycosylase	Hs.584809	31.48	11.79
TDP2	tyrosyl-DNA phosphodiesterase 2	Hs.403010	73.24	4.63
TEAD2	TEA domain family member 2	Hs.515534	4.27	19.51
TERF1	telomeric repeat binding factor (NIMA-interacting) 1	Hs.442707	48.90	24.18
TEX2	testis expressed 2	Hs.175414	15.24	5.42
TEX261	testis expressed 261	Hs.516087	11.50	5.80

TFAM	transcription factor A, mitochondrial	Hs.642966	22.47	19.22
TFAP2C	transcription factor AP-2 gamma (activating enhancer binding protein 2 gamma)	Hs.473152	6.02	4.97
TFB1M	transcription factor B1, mitochondrial	Hs.279908	10.20	4.40
TFDP1	transcription factor Dp-1	Hs.79353	13.37	10.34
TFG	TRK-fused gene	Hs.518123	25.44	45.67
TFPI2	tissue factor pathway inhibitor 2	Hs.438231	6.39	3.56
TFPT	TCF3 (E2A) fusion partner (in childhood Leukemia)	Hs.590939	3.31	2.74
TFRC	transferrin receptor (p90, CD71)	Hs.529618	64.83	7.13
TGFA	transforming growth factor, alpha	Hs.170009	8.85	16.16
TGFB1	transforming growth factor, beta 1	Hs.645227	2.50	6.91
TGFB3	transforming growth factor, beta 3	Hs.592317	7.09	4.25
TGFBR2	transforming growth factor, beta receptor II (70/80 kDa)	Hs.82028	7.03	14.20
TGFBRAP1	transforming growth factor, beta receptor associated protein 1	Hs.446350	3.08	4.15
TGIF2	TGFB-induced factor homeobox 2	Hs.632264	5.53	14.51
TGOLN2	trans-golgi network protein 2	Hs.593382	8.88	2.93
THAP11	THAP domain containing 11	Hs.632200	7.21	16.77
THG1L	tRNA-histidine guanylyltransferase 1-like (S. cerevisiae)	Hs.353090	17.33	6.40
THNSL2	threonine synthase-like 2 (S. cerevisiae)	Hs.516179	3.72	12.45
THOC3	THO complex 3	Hs.484227	16.75	3.80
THOC5	THO complex 5	Hs.75361	4.02	6.45
THOC6	THO complex 6 homolog (Drosophila)	Hs.412304	4.03	25.07
THOC7	THO complex 7 homolog (Drosophila)	Hs.288151	41.03	9.30
TIAL1	TIA1 cytotoxic granule-associated RNA binding protein-like 1	Hs.501203	3.33	26.51
TIMM22	translocase of inner mitochondrial membrane 22 homolog (yeast)	Hs.592108	9.65	5.12
TIMM23	translocase of inner mitochondrial membrane 23 homolog (yeast)	Hs.524308	8.88	3.00
TIMM44	translocase of inner mitochondrial membrane 44 homolog (yeast)	Hs.465784	11.45	17.81
TIPRL	TIP41, TOR signaling pathway regulator-like (S. cerevisiae)	Hs.209431	22.86	3.87
TLE1	transducin-like enhancer of split 1 (E(sp1) homolog, Drosophila)	Hs.689805	3.29	8.53
TLN1	talin 1	Hs.471014	2.79	7.40
TM2D2	TM2 domain containing 2	Hs.7471	14.04	3.45
TM2D3	TM2 domain containing 3	Hs.288912	9.42	24.02
TM4SF1	transmembrane 4 L six family member 1	Hs.730617	14.35	6.72
TM7SF3	transmembrane 7 superfamily member 3	Hs.438641	11.44	2.64
TM9SF1	transmembrane 9 superfamily member 1	Hs.91586	8.80	5.29
TM9SF2	transmembrane 9 superfamily member 2	Hs.654824	18.39	7.91
TM9SF3	transmembrane 9 superfamily member 3	Hs.500674	53.15	15.51
TMBIM1	transmembrane BAX inhibitor motif containing 1	Hs.591605	7.03	7.07
TMC8	transmembrane channel-like 8	Hs.592102	8.48	9.59
TMCO1	transmembrane and coiled-coil domains 1	Hs.31498	18.32	7.01
TMCO3	transmembrane and coiled-coil domains 3	Hs.317593	20.26	24.04
TMED1	transmembrane emp24 protein transport domain containing 1	Hs.515139	3.55	4.83
TMED10	transmembrane emp24-like trafficking protein 10 (yeast)	Hs.74137	52.57	5.53
TMED4	transmembrane emp24 protein transport domain containing 4	Hs.598832	2.51	4.45
TMED5	transmembrane emp24 protein transport domain containing 5	Hs.482873	24.65	6.11
TMED7-TICAM2	TMED7-TICAM2 readthrough	Hs.642817	3.96	2.86
TMED9	transmembrane emp24 protein transport domain containing 9	Hs.279929	8.65	8.16

TMEM101	transmembrane protein 101	Hs.514211	9.02	5.89
TMEM106A	transmembrane protein 106A	Hs.536474	5.64	16.46
TMEM106B	transmembrane protein 106B	Hs.396358	149.22	15.09
TMEM117	transmembrane protein 117	Hs.444668	47.28	7.13
TMEM120B	transmembrane protein 120B	Hs.644504	3.25	12.02
TMEM123	transmembrane protein 123	Hs.503709	124.55	15.41
TMEM128	transmembrane protein 128	Hs.696606	60.72	7.79
TMEM138	transmembrane protein 138	Hs.406530	28.80	4.06
TMEM14A	transmembrane protein 14A	Hs.94896	14.74	3.71
TMEM14C	transmembrane protein 14C	Hs.519557	13.28	3.10
TMEM164	transmembrane protein 164	Hs.496572	2.57	2.92
TMEM165	transmembrane protein 165	Hs.479766	8.61	33.91
TMEM167A	transmembrane protein 167A	Hs.355606	40.77	7.45
TMEM167B	transmembrane protein 167B	Hs.82933	30.41	11.05
TMEM17	transmembrane protein 17	Hs.308028	22.55	3.03
TMEM170A	transmembrane protein 170A	Hs.487510	15.68	6.56
TMEM171	transmembrane protein 171	Hs.162246	12.06	3.25
TMEM179B	transmembrane protein 179B	Hs.381134	4.95	4.74
TMEM18	transmembrane protein 18	Hs.43899	23.97	10.06
TMEM184A	transmembrane protein 184A	Hs.592174	3.98	19.03
TMEM198	transmembrane protein 198	Hs.446664	3.17	2.69
TMEM199	transmembrane protein 199	Hs.707906	7.15	3.52
TMEM2	transmembrane protein 2	Hs.494146	4.64	21.21
TMEM201	transmembrane protein 201	Hs.632365	2.73	3.19
TMEM203	transmembrane protein 203	Hs.726301	7.93	3.11
TMEM206	transmembrane protein 206	Hs.445386	18.95	3.17
TMEM209	transmembrane protein 209	Hs.267245	39.62	4.00
TMEM216	transmembrane protein 216	Hs.26745	26.01	6.28
TMEM222	transmembrane protein 222	Hs.469171	5.24	5.08
TMEM234	transmembrane protein 234	Hs.272299	4.39	4.53
TMEM25	transmembrane protein 25	Hs.564188	7.24	4.42
TMEM30A	transmembrane protein 30A	Hs.108530	134.30	8.31
TMEM38B	transmembrane protein 38B	Hs.411925	23.70	6.82
TMEM39A	transmembrane protein 39A	Hs.594171	12.27	2.70
TMEM40	transmembrane protein 40	Hs.475502	6.14	3.58
TMEM41A	transmembrane protein 41A	Hs.692209	5.10	33.30
TMEM41B	transmembrane protein 41B	Hs.594563	20.40	4.76
TMEM43	transmembrane protein 43	Hs.517817	11.09	3.21
TMEM44	transmembrane protein 44	Hs.478729	4.06	3.41
TMEM5	transmembrane protein 5	Hs.216386	15.07	4.67
TMEM50A	transmembrane protein 50A	Hs.705699	72.45	17.58
TMEM53	transmembrane protein 53	Hs.22157	3.34	35.06
TMEM57	transmembrane protein 57	Hs.189782	9.04	14.61
TMEM59	transmembrane protein 59	Hs.523262	6.91	42.59
TMEM62	transmembrane protein 62	Hs.511175	11.73	5.05
TMEM69	transmembrane protein 69	Hs.436502	42.08	8.47
TMEM87A	transmembrane protein 87A	Hs.730697	9.20	13.53

TMEM9	transmembrane protein 9	Hs.181444	4.00	4.89
TMOD3	tropomodulin 3 (ubiquitous)	Hs.4998	19.31	17.82
TMPPE	transmembrane protein with metallophosphoesterase domain	Hs.443031	4.61	49.31
TMPRSS13	transmembrane protease, serine 13	Hs.266308	7.02	4.75
TMSB10	thymosin beta 10	Hs.446574	3.25	5.44
TMSB4X	thymosin beta 4, X-linked	Hs.437277	23.83	44.27
TMTC1	transmembrane and tetratricopeptide repeat containing 1	Hs.401954	9.79	4.64
TMUB2	transmembrane and ubiquitin-like domain containing 2	Hs.181391	16.22	4.07
TMX1	thioredoxin-related transmembrane protein 1	Hs.125221	74.91	2.83
TMX4	thioredoxin-related transmembrane protein 4	Hs.169358	29.94	8.16
TNC	tenascin C	Hs.143250	4.36	7.43
TNFAIP8	tumor necrosis factor, alpha-induced protein 8	Hs.656274	26.04	6.33
TNFRSF1A	tumor necrosis factor receptor superfamily, member 1A	Hs.279594	3.52	2.79
TNFRSF9	tumor necrosis factor receptor superfamily, member 9	Hs.86447	6.37	3.58
TNFSF10	tumor necrosis factor (ligand) superfamily, member 10	Hs.478275	45.50	40.73
TNFSF12	tumor necrosis factor (ligand) superfamily, member 12	Hs.54673	7.79	5.81
TNIP2	TNFAIP3 interacting protein 2	Hs.726088	4.68	4.60
TNK2	tyrosine kinase, non-receptor, 2	Hs.518513	4.84	5.35
TNPO3	transportin 3	Hs.193613	12.01	19.04
TNRC6B	trinucleotide repeat containing 6B	Hs.372082	7.80	2.88
TOB1	transducer of ERBB2, 1	Hs.730705	9.80	6.25
TOMM20	translocase of outer mitochondrial membrane 20 homolog (yeast)	Hs.533192	26.04	6.08
TOMM40	translocase of outer mitochondrial membrane 40 homolog (yeast)	Hs.655909	5.63	6.08
TOP1	topoisomerase (DNA) I	Hs.472737	19.57	37.38
TOR1AIP1	torsin A interacting protein 1	Hs.496459	39.06	16.55
TOR1B	torsin family 1, member B (torsin B)	Hs.252682	6.32	2.80
TOR3A	torsin family 3, member A	Hs.584957	8.04	18.80
TP53	tumor protein p53	Hs.654481	6.41	67.06
TP53BP1	tumor protein p53 binding protein 1	Hs.440968	13.79	4.50
TP53I11	tumor protein p53 inducible protein 11	Hs.554791	4.61	8.80
TP63	tumor protein p63	Hs.137569	5.59	124.36
TP73	tumor protein p73	Hs.192132	6.93	16.56
TPD52L1	tumor protein D52-like 1	Hs.591347	16.23	4.75
TPD52L2	tumor protein D52-like 2	Hs.473296	11.56	7.34
TPM1	tropomyosin 1 (alpha)	Hs.133892	9.79	7.60
TPM4	tropomyosin 4	Hs.631618	10.16	7.49
TPRG1L	tumor protein p63 regulated 1-like	Hs.20529	13.20	8.30
TPRKB	TP53RK binding protein	Hs.157401	22.86	2.95
TPT1	tumor protein, translationally-controlled 1	Hs.374596	5.71	2.64
TPX2	TPX2, microtubule-associated, homolog (Xenopus laevis)	Hs.244580	24.55	9.92
TRA2B	transformer 2 beta homolog (Drosophila)	Hs.533122	8.19	8.05
TRABD	TraB domain containing	Hs.728286	2.87	11.52
TRAF3	TNF receptor-associated factor 3	Hs.510528	6.91	7.15
TRAF3IP2	TRAF3 interacting protein 2	Hs.561514	6.28	5.67
TRAM1	translocation associated membrane protein 1	Hs.491988	222.28	15.36
TRAP1	TNF receptor-associated protein 1	Hs.30345	3.25	2.55
TRAPPC2P1	trafficking protein particle complex 2 pseudogene 1	Hs.446620	10.01	9.09

TRIAP1	TP53 regulated inhibitor of apoptosis 1	Hs.69499	14.87	7.01
TRIM14	tripartite motif containing 14	Hs.575631	3.93	5.53
TRIM26	tripartite motif containing 26	Hs.720054	3.30	87.36
TRIM32	tripartite motif containing 32	Hs.591910	9.83	4.51
TRIM39	tripartite motif containing 39	Hs.413493	7.42	331.24
TRIM59	tripartite motif containing 59	Hs.212957	15.53	3.70
TRIM68	tripartite motif containing 68	Hs.523438	17.45	5.07
TRIP10	thyroid hormone receptor interactor 10	Hs.515094	7.70	7.07
TRIP13	thyroid hormone receptor interactor 13	Hs.436187	12.20	5.23
TRIT1	tRNA isopentenyltransferase 1	Hs.356554	38.05	11.07
TRMT2B	TRM2 tRNA methyltransferase 2 homolog B (S. cerevisiae)	Hs.496501	7.55	5.50
TRMT6	tRNA methyltransferase 6 homolog (S. cerevisiae)	Hs.128791	13.21	5.62
TRPC4AP	transient receptor potential cation channel, subfamily C, member 4 associated protein	Hs.168073	5.86	7.86
TSC22D4	TSC22 domain family, member 4	Hs.469798	4.39	5.84
TSEN2	tRNA splicing endonuclease 2 homolog (S. cerevisiae)	Hs.335550	5.91	8.95
TSFM	Ts translation elongation factor, mitochondrial	Hs.632704	11.19	2.96
TSN	translin	Hs.75066	18.42	11.93
TSNAX	translin-associated factor X	Hs.13318	13.51	5.24
TSPAN13	tetraspanin 13	Hs.364544	17.70	23.87
TSPAN14	tetraspanin 14	Hs.718943	3.41	5.30
TSPYL4	TSPY-like 4	Hs.284141	14.33	3.30
TSSC1	tumor suppressing subtransferable candidate 1	Hs.502770	3.98	3.44
TTBK2	tau tubulin kinase 2	Hs.646511	7.35	3.76
TTC1	tetratricopeptide repeat domain 1	Hs.519718	28.80	16.97
TTC17	tetratricopeptide repeat domain 17	Hs.191186	7.13	40.41
TTC22	tetratricopeptide repeat domain 22	Hs.16230	3.98	7.74
TTC27	tetratricopeptide repeat domain 27	Hs.468125	30.68	4.19
TTC39C	tetratricopeptide repeat domain 39C	Hs.128576	8.60	4.36
TTC4	tetratricopeptide repeat domain 4	Hs.729029	7.70	3.14
TTC9C	tetratricopeptide repeat domain 9C	Hs.31704	3.99	9.71
TTLL5	tubulin tyrosine ligase-like family, member 5	Hs.709609	6.94	12.91
TTYH2	tweety homolog 2 (Drosophila)	Hs.27935	10.01	12.21
TTYH3	tweety homolog 3 (Drosophila)	Hs.440899	4.45	6.16
TUBA1A	tubulin, alpha 1a	Hs.654422	10.13	4.18
TUBB	tubulin, beta class I	Hs.636480	4.38	2.74
TUBB4A	tubulin, beta 4A class IVa	Hs.110837	7.82	32.81
TUBD1	tubulin, delta 1	Hs.463638	27.39	6.53
TUBG2	tubulin, gamma 2	Hs.708059	5.61	4.65
TUBGCP3	tubulin, gamma complex associated protein 3	Hs.224152	7.07	6.86
TWF1	twinfilin, actin-binding protein, homolog 1 (Drosophila)	Hs.189075	157.49	19.96
TWF2	twinfilin, actin-binding protein, homolog 2 (Drosophila)	Hs.436439	5.27	4.96
TWIST1	twist homolog 1 (Drosophila)	Hs.66744	5.20	28.38
TXNDC11	thioredoxin domain containing 11	Hs.313847	6.84	4.72
TXNDC12	thioredoxin domain containing 12 (endoplasmic reticulum)	Hs.476033	18.49	32.49
TXNDC5	thioredoxin domain containing 5 (endoplasmic reticulum)	Hs.150837	5.95	8.24
TXNDC9	thioredoxin domain containing 9	Hs.536122	23.16	7.72
TXNRD1	thioredoxin reductase 1	Hs.728817	7.20	44.55

TXNRD3	thioredoxin reductase 3	Hs.477475	26.79	14.33
TYSND1	trypsin domain containing 1	Hs.533655	4.62	7.52
TYW1	tRNA-yW synthesizing protein 1 homolog (S. cerevisiae)	Hs.520917	7.39	3.48
U2AF1L4	U2 small nuclear RNA auxiliary factor 1-like 4	Hs.351558	4.16	4.58
UAP1L1	UDP-N-acteylglucosamine pyrophosphorylase 1-like 1	Hs.142076	3.38	2.50
UBA2	ubiquitin-like modifier activating enzyme 2	Hs.631580	54.49	6.80
UBAC2	UBA domain containing 2	Hs.508545	24.90	12.98
UBAP1	ubiquitin associated protein 1	Hs.268963	16.15	3.45
UBE2D3	ubiquitin-conjugating enzyme E2D 3	Hs.518773	8.52	18.14
UBE2E2	ubiquitin-conjugating enzyme E2E 2	Hs.475688	22.60	7.50
UBE2E3	ubiquitin-conjugating enzyme E2E 3	Hs.470804	12.20	27.66
UBE2F	ubiquitin-conjugating enzyme E2F (putative)	Hs.471785	10.38	14.48
UBE2G1	ubiquitin-conjugating enzyme E2G 1	Hs.462035	33.96	5.70
UBE2H	ubiquitin-conjugating enzyme E2H	Hs.643548	18.42	4.64
UBE2I	ubiquitin-conjugating enzyme E2I	Hs.302903	2.89	9.89
UBE2J2	ubiquitin-conjugating enzyme E2, J2	Hs.191987	5.15	5.98
UBE2L6	ubiquitin-conjugating enzyme E2L 6	Hs.425777	6.92	5.64
UBE2M	ubiquitin-conjugating enzyme E2M	Hs.406068	3.07	4.06
UBE2V1	ubiquitin-conjugating enzyme E2 variant 1	Hs.420529	11.79	4.58
UBE2W	ubiquitin-conjugating enzyme E2W (putative)	Hs.718604	12.75	10.51
UBE2Z	ubiquitin-conjugating enzyme E2Z	Hs.514297	4.18	3.53
UBE3B	ubiquitin protein ligase E3B	Hs.374067	2.75	4.91
UBE3C	ubiquitin protein ligase E3C	Hs.118351	5.18	9.37
UBP1	upstream binding protein 1 (LBP-1a)	Hs.729120	38.41	5.63
UBQLN1	ubiquilin 1	Hs.9589	29.48	15.30
UBQLN2	ubiquilin 2	Hs.179309	29.61	4.05
UCHL3	ubiquitin carboxyl-terminal esterase L3 (ubiquitin thiolesterase)	Hs.162241	20.40	3.02
UCHL5	ubiquitin carboxyl-terminal hydrolase L5	Hs.145469	70.90	25.57
UFC1	ubiquitin-fold modifier conjugating enzyme 1	Hs.301412	7.91	9.41
UFM1	ubiquitin-fold modifier 1	Hs.727566	6.25	3.22
UFSP2	UFM1-specific peptidase 2	Hs.713548	17.83	6.24
UGCG	UDP-glucose ceramide glucosyltransferase	Hs.304249	10.61	3.78
UGGT2	UDP-glucose glycoprotein glucosyltransferase 2	Hs.193226	36.16	11.83
UGT1A6	UDP glucuronosyltransferase 1 family, polypeptide A6	Hs.554822	15.88	7.13
UGT1A8	UDP glucuronosyltransferase 1 family, polypeptide A8	Hs.554822	18.50	9.80
UGT8	UDP glycosyltransferase 8	Hs.144197	12.83	3.91
UHMK1	U2AF homology motif (UHM) kinase 1	Hs.127310	7.59	13.98
UHRF2	ubiquitin-like with PHD and ring finger domains 2	Hs.493401	10.62	12.92
ULBP1	UL16 binding protein 1	Hs.653255	65.83	18.54
ULBP2	UL16 binding protein 2	Hs.656778	25.67	12.42
ULK3	unc-51-like kinase 3 (C. elegans)	Hs.513034	11.19	6.15
UNC119	unc-119 homolog (C. elegans)	Hs.410455	20.85	15.63
UNC119B	unc-119 homolog B (C. elegans)	Hs.127610	13.31	9.65
UNC50	unc-50 homolog (C. elegans)	Hs.13370	40.93	8.39
UNKL	unkempt homolog (Drosophila)-like	Hs.643536	3.28	3.81
URM1	ubiquitin related modifier 1	Hs.495229	3.10	13.86
UROS	uroporphyrinogen III synthase	Hs.501376	3.71	10.50

USP10	ubiquitin specific peptidase 10	Hs.136778	2.98	4.53
USP11	ubiquitin specific peptidase 11	Hs.171501	2.57	2.82
USP21	ubiquitin specific peptidase 21	Hs.8015	3.92	22.66
USP30	ubiquitin specific peptidase 30	Hs.486434	6.20	8.10
USP42	ubiquitin specific peptidase 42	Hs.31856	2.73	4.10
USP48	ubiquitin specific peptidase 48	Hs.467524	45.86	6.64
UTP11L	UTP11-like, U3 small nucleolar ribonucleoprotein, (yeast)	Hs.472038	48.78	17.73
UTP14A	UTP14, U3 small nucleolar ribonucleoprotein, homolog A (yeast)	Hs.458598	3.85	6.31
UTP18	UTP18, small subunit (SSU) processome component, homolog (yeast)	Hs.709327	17.56	5.34
UTP3	UTP3, small subunit (SSU) processome component, homolog (S. cerevisiae)	Hs.322901	80.18	7.60
UXS1	UDP-glucuronate decarboxylase 1	Hs.730756	20.22	3.86
VAC14	Vac14 homolog (S. cerevisiae)	Hs.445061	3.70	13.02
VAMP3	vesicle-associated membrane protein 3 (cellubrevin)	Hs.66708	16.60	9.50
VANGL2	vang-like 2 (van gogh, Drosophila)	Hs.99477	4.32	2.84
VAPB	VAMP (vesicle-associated membrane protein)-associated protein B and C	Hs.182625	8.26	8.65
VAT1	vesicle amine transport protein 1 homolog (T. californica)	Hs.514199	2.93	4.57
VAV3	vav 3 guanine nucleotide exchange factor	Hs.267659	56.57	3.26
VCL	vinculin	Hs.643896	24.50	2.98
VDAC1	voltage-dependent anion channel 1	Hs.519320	13.20	13.78
VDAC3	voltage-dependent anion channel 3	Hs.699301	15.82	3.54
VEGFA	vascular endothelial growth factor A	Hs.73793	4.98	3.57
VGLL4	vestigial like 4 (Drosophila)	Hs.38032	7.00	4.35
VIPR1	vasoactive intestinal peptide receptor 1	Hs.348500	7.31	3.20
VKORC1L1	vitamin K epoxide reductase complex, subunit 1-like 1	Hs.427232	55.46	10.10
VLDLR	very low density lipoprotein receptor	Hs.370422	3.62	10.97
VOPP1	vesicular, overexpressed in cancer, prosurvival protein 1	Hs.488307	13.51	2.56
VPS29	vacuolar protein sorting 29 homolog (S. cerevisiae)	Hs.600114	9.63	13.36
VPS33A	vacuolar protein sorting 33 homolog A (S. cerevisiae)	Hs.592009	9.62	4.38
VPS33B	vacuolar protein sorting 33 homolog B (yeast)	Hs.728254	2.67	2.91
VPS35	vacuolar protein sorting 35 homolog (S. cerevisiae)	Hs.454528	32.48	6.12
VPS39	vacuolar protein sorting 39 homolog (S. cerevisiae)	Hs.88025	5.77	12.87
VPS54	vacuolar protein sorting 54 homolog (S. cerevisiae)	Hs.48499	19.60	25.12
VRK3	vaccinia related kinase 3	Hs.443330	7.15	13.62
VSIG10	V-set and immunoglobulin domain containing 10	Hs.187624	2.82	5.26
VTA1	Vps20-associated 1 homolog (S. cerevisiae)	Hs.431367	51.36	41.69
VWA1	von Willebrand factor A domain containing 1	Hs.449009	3.11	2.89
WAC	WW domain containing adaptor with coiled-coil	Hs.730605	47.41	13.54
WASF2	WAS protein family, member 2	Hs.469244	3.46	3.78
WBP2	WW domain binding protein 2	Hs.514489	8.62	8.46
WBSCR16	Williams-Beuren syndrome chromosome region 16	Hs.723684	7.64	17.32
WDR1	WD repeat domain 1	Hs.128548	5.88	18.50
WDR13	WD repeat domain 13	Hs.521973	2.55	4.31
WDR20	WD repeat domain 20	Hs.36859	7.84	19.95
WDR3	WD repeat domain 3	Hs.310809	10.99	8.02
WDR4	WD repeat domain 4	Hs.248815	2.89	2.70
WDR41	WD repeat domain 41	Hs.482573	13.32	2.72

WDR48	WD repeat domain 48	Hs.109778	32.11	2.87
WDR59	WD repeat domain 59	Hs.280951	6.40	2.72
WDR61	WD repeat domain 61	Hs.513055	25.11	8.46
WDR70	WD repeat domain 70	Hs.213690	12.52	3.69
WDR73	WD repeat domain 73	Hs.728276	5.52	2.94
WDR74	WD repeat domain 74	Hs.730651	3.24	3.56
WDR77	WD repeat domain 77	Hs.204773	12.11	8.54
WDR91	WD repeat domain 91	Hs.459858	4.58	4.66
WDR92	WD repeat domain 92	Hs.631877	27.58	5.65
WDYHV1	WDYHV motif containing 1	Hs.18029	11.63	3.69
WEE1	WEE1 homolog (S. pombe)	Hs.249441	16.22	3.18
WFDC2	WAP four-disulfide core domain 2	Hs.2719	2.98	3.71
WHSC1	Wolf-Hirschhorn syndrome candidate 1	Hs.113876	9.42	29.68
WIBG	within bgcn homolog (Drosophila)	Hs.505687	4.87	7.73
WIPF2	WAS/WASL interacting protein family, member 2	Hs.421622	7.22	2.79
WNT7B	wingless-type MMTV integration site family, member 7B	Hs.512714	3.97	43.63
WRAP53	WD repeat containing, antisense to TP53	Hs.437460	4.62	133.30
WRAP73	WD repeat containing, antisense to TP73	Hs.31714	7.67	4.56
WRB	tryptophan rich basic protein	Hs.198308	37.64	35.60
WRNIP1	Werner helicase interacting protein 1	Hs.236828	12.53	9.11
WTAP	Wilms tumor 1 associated protein	Hs.446091	11.81	74.84
WWC3	WWC family member 3	Hs.527524	4.16	2.84
WWOX	WW domain containing oxidoreductase	Hs.461453	3.02	14.77
WWP2	WW domain containing E3 ubiquitin protein ligase 2	Hs.408458	10.76	7.13
XPNPEP1	X-prolyl aminopeptidase (aminopeptidase P) 1, soluble	Hs.390623	14.03	6.35
XPO1	exportin 1 (CRM1 homolog, yeast)	Hs.370770	51.63	17.54
XPO5	exportin 5	Hs.203206	4.76	9.08
XPO6	exportin 6	Hs.460468	5.61	37.06
XPOT	exportin, tRNA (nuclear export receptor for tRNAs)	Hs.85951	41.06	67.46
XRCC6BP1	XRCC6 binding protein 1	Hs.61188	27.06	5.60
YAP1	Yes-associated protein 1	Hs.503692	7.59	6.09
YARS	tyrosyl-tRNA synthetase	Hs.213264	5.11	13.42
YARS2	tyrosyl-tRNA synthetase 2, mitochondrial	Hs.505231	10.87	3.31
YBEY	ybeY metallopeptidase (putative)	Hs.474066	5.47	4.96
YBX1	Y box binding protein 1	Hs.473583	11.38	17.58
YEATS2	YEATS domain containing 2	Hs.632575	3.61	5.12
YIPF5	Yip1 domain family, member 5	Hs.372050	21.58	3.26
YME1L1	YME1-like 1 (S. cerevisiae)	Hs.499145	173.27	25.40
YPEL3	yippee-like 3 (Drosophila)	Hs.513491	4.95	7.91
YPEL5	yippee-like 5 (Drosophila)	Hs.515890	54.36	3.74
YTHDF1	YTH domain family, member 1	Hs.11747	5.12	4.66
YTHDF2	YTH domain family, member 2	Hs.532286	19.73	3.39
YWHAB	tyrosine 3-monooxygenase/tryptophan 5-monooxygenase activation protein, beta polypeptide	Hs.643544	46.75	3.68
YWHAE	tyrosine 3-monooxygenase/tryptophan 5-monooxygenase activation protein, epsilon polypeptide	Hs.513851	5.14	295.89
YWHAH	tyrosine 3-monooxygenase/tryptophan 5-monooxygenase activation protein, eta polypeptide	Hs.226755	13.19	7.13

YWHAQ	tyrosine 3-monooxygenase/tryptophan 5-monooxygenase activation protein, theta polypeptide	Hs.74405	15.15	6.68
YWHAZ	tyrosine 3-monooxygenase/tryptophan 5-monooxygenase activation protein, zeta polypeptide	Hs.492407	11.55	14.73
YY1AP1	YY1 associated protein 1	Hs.584927	5.14	15.62
ZBTB22	zinc finger and BTB domain containing 22	Hs.206770	4.90	19.31
ZBTB43	zinc finger and BTB domain containing 43	Hs.355581	15.30	6.70
ZBTB44	zinc finger and BTB domain containing 44	Hs.178499	7.16	19.87
ZBTB7A	zinc finger and BTB domain containing 7A	Hs.591384	4.94	6.67
ZBTB9	zinc finger and BTB domain containing 9	Hs.591805	5.72	5.32
ZC3H11A	zinc finger CCCH-type containing 11A	Hs.532399	12.17	6.40
ZC3H18	zinc finger CCCH-type containing 18	Hs.93670	4.64	9.74
ZCCHC14	zinc finger, CCHC domain containing 14	Hs.156231	18.54	9.11
ZCCHC3	zinc finger, CCHC domain containing 3	Hs.28608	10.37	2.95
ZCRB1	zinc finger CCHC-type and RNA binding motif 1	Hs.496279	19.17	8.36
ZDHHC13	zinc finger, DHHC-type containing 13	Hs.188569	19.95	16.48
ZDHHC18	zinc finger, DHHC-type containing 18	Hs.523710	8.50	6.78
ZFAND1	zinc finger, AN1-type domain 1	Hs.655453	50.12	10.65
ZFAND3	zinc finger, AN1-type domain 3	Hs.36959	9.53	21.05
ZFAND6	zinc finger, AN1-type domain 6	Hs.730626	46.47	5.05
ZFHX3	zinc finger homeobox 3	Hs.598297	10.67	4.05
ZFP62	zinc finger protein 62 homolog (mouse)	Hs.509227	8.00	8.43
ZFP64	zinc finger protein 64 homolog (mouse)	Hs.473082	4.67	4.79
ZFP91	zinc finger protein 91 homolog (mouse)	Hs.524920	24.39	6.91
ZFPL1	zinc finger protein-like 1	Hs.98170	3.14	9.59
ZHX2	zinc fingers and homeoboxes 2	Hs.377090	4.47	5.03
ZKSCAN4	zinc finger with KRAB and SCAN domains 4	Hs.44720	4.87	6.49
ZMAT5	zinc finger, matrin-type 5	Hs.713647	3.25	7.63
ZMPSTE24	zinc metallopeptidase (STE24 homolog, S. cerevisiae)	Hs.132642	63.21	3.44
ZMYM6NB	ZMYM6 neighbor	Hs.533986	24.08	12.24
ZMYND19	zinc finger, MYND-type containing 19	Hs.128096	5.34	12.91
ZNF131	zinc finger protein 131	Hs.535804	31.10	6.74
ZNF134	zinc finger protein 134	Hs.469694	14.49	12.05
ZNF155	zinc finger protein 155	Hs.502127	4.26	5.50
ZNF16	zinc finger protein 16	Hs.493225	4.00	3.65
ZNF165	zinc finger protein 165	Hs.535177	22.84	3.02
ZNF174	zinc finger protein 174	Hs.155204	3.95	3.99
ZNF2	zinc finger protein 2	Hs.590916	3.68	3.53
ZNF207	zinc finger protein 207	Hs.500775	25.45	5.89
ZNF213	zinc finger protein 213	Hs.115284	3.06	2.61
ZNF217	zinc finger protein 217	Hs.155040	18.52	8.72
ZNF226	zinc finger protein 226	Hs.145956	20.54	4.43
ZNF232	zinc finger protein 232	Hs.279914	8.72	2.91
ZNF24	zinc finger protein 24	Hs.514802	21.26	3.87
ZNF275	zinc finger protein 275	Hs.348963	7.76	3.99
ZNF296	zinc finger protein 296	Hs.192237	3.90	7.31
ZNF326	zinc finger protein 326	Hs.306221	19.47	23.83
ZNF414	zinc finger protein 414	Hs.515114	3.93	8.82

ZNF462	zinc finger protein 462	Hs.370379	19.94	2.88
ZNF488	zinc finger protein 488	Hs.27788	6.04	5.50
ZNF511	zinc finger protein 511	Hs.422113	3.93	12.97
ZNF513	zinc finger protein 513	Hs.515872	6.51	7.32
ZNF530	zinc finger protein 530	Hs.97111	4.26	2.83
ZNF543	zinc finger protein 543	Hs.202544	6.15	2.92
ZNF544	zinc finger protein 544	Hs.438994	9.61	16.19
ZNF556	zinc finger protein 556	Hs.287433	5.52	4.30
ZNF561	zinc finger protein 561	Hs.720081	26.20	5.98
ZNF562	zinc finger protein 562	Hs.371107	19.43	12.85
ZNF575	zinc finger protein 575	Hs.213534	3.80	5.44
ZNF655	zinc finger protein 655	Hs.599798	34.53	4.46
ZNF691	zinc finger protein 691	Hs.20879	5.94	5.42
ZNF696	zinc finger protein 696	Hs.512740	3.89	2.76
ZNF703	zinc finger protein 703	Hs.726062	4.35	15.16
ZNF74	zinc finger protein 74	Hs.517418	3.58	2.73
ZNF75A	zinc finger protein 75a	Hs.513292	20.93	8.40
ZNF770	zinc finger protein 770	Hs.730754	16.27	15.64
ZNF79	zinc finger protein 79	Hs.522399	3.02	4.04
ZNF821	zinc finger protein 821	Hs.643531	4.62	2.79
ZNF830	zinc finger protein 830	Hs.413678	54.08	4.81
ZNF839	zinc finger protein 839	Hs.730727	7.82	2.86
ZNRD1	zinc ribbon domain containing 1	Hs.57813	6.10	10.23
ZWINT	ZW10 interactor	Hs.591363	23.59	6.03
ZYX	zyxin	Hs.490415	6.33	7.44

Supplementary Table S4: Functional charts of TIA1 target genes

GO Term (Biological Process)	*P* Value (FDR)
GO:0006396 RNA processing	6.04E–14
GO:0000278 mitotic cell cycle	6.49E–14
GO:0008380 RNA splicing	1.45E–12
GO:0016071 mRNA metabolic process	1.47E–11
GO:0007049 cell cycle	4.23E–11
GO:0006397 mRNA processing	1.37E–10
GO:0022402 cell cycle process	3.01E–10
GO:0015031 protein transport	8.13E–10
GO:0045184 establishment of protein localization	1.02E–09
GO:0008104 protein localization	2.31E–09
GO:0046907 intracellular transport	2.30E–08
GO:0009057 macromolecule catabolic process	3.06E–08
GO:0000375 RNA splicing, via transesterification reactions	5.07E–07
GO:0000377 RNA splicing, via transesterification reactions with bulged adenosine as nucleophile	5.07E–07
GO:0000398 nuclear mRNA splicing, via spliceosome	5.07E–07
GO:0044265 cellular macromolecule catabolic process	1.68E–06
GO:0048285 organelle fission	1.28E–05
GO:0000280 nuclear division	1.36E–05
GO:0007067 mitosis	1.36E–05
GO:0000087 M phase of mitotic cell cycle	3.00E–05
GO:0032268 regulation of cellular protein metabolic process	3.35E–05
GO:0030163 protein catabolic process	5.54E–05
GO:0022403 cell cycle phase	8.79E–05

GO:0010605 negative regulation of macromolecule metabolic process	1.23E–04
GO:0015931 nucleobase, nucleoside, nucleotide and nucleic acid transport	2.97E–04
GO:0032269 negative regulation of cellular protein metabolic process	3.11E–04
GO:0031400 negative regulation of protein modification process	4.64E–04
GO:0051248 negative regulation of protein metabolic process	4.93E–04
GO:0051603 proteolysis involved in cellular protein catabolic process	5.16E–04
GO:0051726 regulation of cell cycle	6.28E–04
GO:0044257 cellular protein catabolic process	6.93E–04
GO:0051028 mRNA transport	7.37E–04
GO:0031396 regulation of protein ubiquitination	8.62E–04
GO:0050657 nucleic acid transport	0.001204561
GO:0050658 RNA transport	0.001204561
GO:0051236 establishment of RNA localization	0.001204561
GO:0034613 cellular protein localization	0.001557277
GO:0070727 cellular macromolecule localization	0.00220973
GO:0006457 protein folding	0.002623674
GO:0006403 RNA localization	0.002691409
GO:0051439 regulation of ubiquitin-protein ligase activity during mitotic cell cycle	0.002762372
GO:0048193 Golgi vesicle transport	0.002791835
GO:0051325 interphase	0.004104229
GO:0043161 proteasomal ubiquitin-dependent protein catabolic process	0.004484037
GO:0010498 proteasomal protein catabolic process	0.004484037
GO:0043632 modification-dependent macromolecule catabolic process	0.005012986
GO:0019941 modification-dependent protein catabolic process	0.005012986
GO:0051329 interphase of mitotic cell cycle	0.005745841
GO:0051438 regulation of ubiquitin-protein ligase activity	0.006737282
GO:0031398 positive regulation of protein ubiquitination	0.011111449
GO:0051437 positive regulation of ubiquitin-protein ligase activity during mitotic cell cycle	0.013588336
GO:0051340 regulation of ligase activity	0.015561087
GO:0006886 intracellular protein transport	0.018079622
GO:0051436 negative regulation of ubiquitin-protein ligase activity during mitotic cell cycle	0.018921247
GO:0031145 anaphase-promoting complex-dependent proteasomal ubiquitin-dependent protein catabolic process	0.018921247
GO:0031397 negative regulation of protein ubiquitination	0.023216809
GO:0051443 positive regulation of ubiquitin-protein ligase activity	0.024497378
GO:0051352 negative regulation of ligase activity	0.03425712
GO:0051444 negative regulation of ubiquitin-protein ligase activity	0.03425712
GO:0051301 cell division	0.035862823
GO:0016192 vesicle-mediated transport	0.042988783
GO:0034660 ncRNA metabolic process	0.048939173

Supplementary Table S5: List of the candidate TIA1 target mRNAs encoding cell cycle associating factors

Gene Symbol	Gene Name	UniGeneID	Fold change (/GAPDH mRNA)	
			Microarray	RIP Seq
ABL1	c-abl oncogene 1, non-receptor tyrosine kinase	Hs.431048	4.81	3.56
ADAM17	ADAM metallopeptidase domain 17	Hs.404914	20.71	8.57
AHR	aryl hydrocarbon receptor	Hs.171189	36.37	7.59
ALG11	asparagine-linked glycosylation 11, alpha-1, 2-mannosyltransferase homolog (yeast)	Hs.512963	10.32	5.05
ANAPC10	anaphase promoting complex subunit 10	Hs.480876	58.7	10.5
ANAPC13	anaphase promoting complex subunit 13	Hs.106909	8.89	19.63
ANXA1	annexin A1	Hs.494173	98.06	5.77
ARHGAP8	Rho GTPase activating protein 8	Hs.102336	2.95	5.59

ARHGEF11	Rho guanine nucleotide exchange factor (GEF) 11	Hs.516954	5.04	10.77
AURKA	aurora kinase A	Hs.250822	19.56	6.28
AURKAPS1	aurora kinase A pseudogene 1	Hs.654849	9.91	8.52
AURKB	aurora kinase B	Hs.442658	9.24	19.54
BANP	BTG3 associated nuclear protein	Hs.461705	4.03	3.54
BAX	BCL2-associated X protein	Hs.624291	4.32	6.24
BBS4	Bardet-Biedl syndrome 4	Hs.208681	18.51	4.73
BCAT1	branched chain amino-acid transaminase 1, cytosolic	Hs.438993	40.04	30.16
BUB1	budding uninhibited by benzimidazoles 1 homolog (yeast)	Hs.469649	38.01	14.11
BUB1B	budding uninhibited by benzimidazoles 1 homolog beta (yeast)	Hs.513645	44.99	10.98
CABLES2	Cdk5 and Abl enzyme substrate 2	Hs.301040	6.08	2.86
CALM2	calmodulin 2 (phosphorylase kinase, delta)	Hs.468442	38.29	6.85
CALM3	calmodulin 3 (phosphorylase kinase, delta)	Hs.515487	4.26	3.74
CAMK2D	calcium/calmodulin-dependent protein kinase II delta	Hs.144114	8.4	17.55
CAMK2G	calcium/calmodulin-dependent protein kinase II gamma	Hs.523045	13.33	8.17
CCNA2	cyclin A2	Hs.58974	24.29	12.16
CCND1	cyclin D1	Hs.523852	12.75	11.39
CCNF	cyclin F	Hs.1973	3.61	8.39
CCNG1	cyclin G1	Hs.79101	10.74	10.79
CCNH	cyclin H	Hs.292524	40.33	9.27
CCNK	cyclin K	Hs.510409	10.3	8.19
CCNT1	cyclin T1	Hs.279906	13.77	13.56
CDC123	cell division cycle 123 homolog (S. cerevisiae)	Hs.412842	24.22	10.74
CDC20	cell division cycle 20 homolog (S. cerevisiae)	Hs.524947	2.84	2.62
CDC23	cell division cycle 23 homolog (S. cerevisiae)	Hs.73625	17.52	8.64
CDC25C	cell division cycle 25 homolog C (S. pombe)	Hs.656	8.5	3
CDC26	cell division cycle 26 homolog (S. cerevisiae)	Hs.727648	16.61	2.86
CDC27	cell division cycle 27 homolog (S. cerevisiae)	Hs.463295	33.8	22.76
CDC40	cell division cycle 40 homolog (S. cerevisiae)	Hs.428147	26.35	10.74
CDC42	cell division cycle 42 (GTP binding protein, 25 kDa)	Hs.467637	7.02	23.06
CDCA2	cell division cycle associated 2	Hs.33366	50.47	5.72
CDK11A	cyclin-dependent kinase 11A	Hs.651228	8.31	4.33
CDK11B	cyclin-dependent kinase 11B	Hs.709182	3.07	2.53
CDK2	cyclin-dependent kinase 2	Hs.19192	5.6	22.72
CDK5	cyclin-dependent kinase 5	Hs.647078	4.98	10.75
CDK6	cyclin-dependent kinase 6	Hs.119882	69.99	12
CDK7	cyclin-dependent kinase 7	Hs.184298	43.76	7.33
CDKN1B	cyclin-dependent kinase inhibitor 1B (p27, Kip1)	Hs.238990	10.65	7.5
CDKN3	cyclin-dependent kinase inhibitor 3	Hs.84113	14.15	9.16
CENPA	centromere protein A	Hs.1594	16.93	3.42
CENPF	centromere protein F, 350/400 kDa (mitosin)	Hs.497741	46.39	9.04
CENPO	centromere protein O	Hs.467898	3.86	3.09
CEP250	centrosomal protein 250 kDa	Hs.443976	3.4	3.44
CEP63	centrosomal protein 63 kDa	Hs.443301	20.17	11.04
CETN2	centrin, EF-hand protein, 2	Hs.82794	51.66	6
CFL1	cofilin 1 (non-muscle)	Hs.170622	7.18	17.52
CHAF1A	chromatin assembly factor 1, subunit A (p150)	Hs.79018	3.6	2.64
CHEK1	checkpoint kinase 1	Hs.24529	20.88	19.51
CHEK2	checkpoint kinase 2	Hs.291363	24.74	9.02
CHMP1A	charged multivesicular body protein 1A	Hs.589427	3.18	4.75
CINP	cyclin-dependent kinase 2 interacting protein	Hs.129634	6.58	9.47
CKAP5	cytoskeleton associated protein 5	Hs.201253	58.63	11.01
CKS1B	CDC28 protein kinase regulatory subunit 1B	Hs.374378	5.49	3.01
CLASP2	cytoplasmic linker associated protein 2	Hs.108614	11.76	8.15
CNTROB	centrobin, centrosomal BRCA2 interacting protein	Hs.348012	4.01	3.06

CTNNB1	catenin (cadherin-associated protein), beta 1, 88 kDa	Hs.476018	4.38	4.59
CUL1	cullin 1	Hs.146806	11.03	4.87
CUL4A	cullin 4A	Hs.339735	18.82	7.05
DDIT3	DNA-damage-inducible transcript 3	Hs.505777	20.54	27.31
DDX11	DEAD/H (Asp-Glu-Ala-Asp/His) box polypeptide 11	Hs.443960	4.59	2.51
DLGAP5	discs, large (Drosophila) homolog-associated protein 5	Hs.77695	83.48	13.55
DNM2	dynamin 2	Hs.211463	2.54	9.22
DSN1	DSN1, MIND kinetochore complex component, homolog (S. cerevisiae)	Hs.632268	35.91	8.25
DST	dystonin	Hs.604915	12.12	4.93
E2F4	E2F transcription factor 4, p107/p130-binding	Hs.108371	10.02	19.25
E2F6	E2F transcription factor 6	Hs.603093	11.78	4.83
EGFR	epidermal growth factor receptor	Hs.488293	5.75	10.27
FANCI	Fanconi anemia, complementation group I	Hs.513126	14.65	4.28
GADD45A	growth arrest and DNA-damage-inducible, alpha	Hs.80409	22.99	4.87
GAK	cyclin G associated kinase	Hs.369607	3.17	6.22
GAS1	growth arrest-specific 1	Hs.65029	3.26	4.02
GSG2	germ cell associated 2 (haspin)	Hs.534059	3.56	7.96
GSK3B	glycogen synthase kinase 3 beta	Hs.445733	12.89	4.72
GSPT1	G1 to S phase transition 1	Hs.528780	12.53	22.93
GTSE1	G-2 and S-phase expressed 1	Hs.386189	5.6	2.65
HAUS1	HAUS augmin-like complex, subunit 1	Hs.436617	51.51	13.25
HAUS2	HAUS augmin-like complex, subunit 2	Hs.14347	33.44	7.06
HAUS4	HAUS augmin-like complex, subunit 4	Hs.442782	3.35	2.71
HAUS5	HAUS augmin-like complex, subunit 5	Hs.7426	3.75	2.99
HAUS8	HAUS augmin-like complex, subunit 8	Hs.404088	7.7	2.93
HHEX	hematopoietically expressed homeobox	Hs.118651	9.78	5.33
HINFP	histone H4 transcription factor	Hs.504091	4.36	2.7
HOXB4	homeobox B4	Hs.664706	11.34	2.89
ILF3	interleukin enhancer binding factor 3, 90 kDa	Hs.465885	4.01	2.9
ING1	inhibitor of growth family, member 1	Hs.46700	5.16	3.46
INHBA	inhibin, beta A	Hs.583348	23.89	3.79
IRF6	interferon regulatory factor 6	Hs.719361	10.87	15.87
ITGAE	integrin, alpha E (antigen CD103, human mucosal lymphocyte antigen 1; alpha polypeptide)	Hs.513867	32.09	7.78
ITGB1	integrin, beta 1 (fibronectin receptor, beta polypeptide, antigen CD29 includes MDF2, MSK12)	Hs.643813	16.76	92.82
KHDRBS1	KH domain containing, RNA binding, signal transduction associated 1	Hs.445893	22.55	2.54
KIFC1	kinesin family member C1	Hs.436912	5.27	5.95
KLHDC3	kelch domain containing 3	Hs.412468	7.9	13.41
LIN9	lin-9 homolog (C. elegans)	Hs.120817	17.98	7.17
MACF1	microtubule-actin crosslinking factor 1	Hs.472475	3.95	3.58
MAD2L1	MAD2 mitotic arrest deficient-like 1 (yeast)	Hs.591697	36.55	10.39
MAP2K1	mitogen-activated protein kinase kinase 1	Hs.145442	30.79	9.76
MAPK1	mitogen-activated protein kinase 1	Hs.431850	20.84	17.86
MAPK12	mitogen-activated protein kinase 12	Hs.432642	2.8	12.15
MAPK3	mitogen-activated protein kinase 3	Hs.861	4.71	4.58
MAPRE1	microtubule-associated protein, RP/EB family, member 1	Hs.472437	17.84	7.48
MDM2	Mdm2 p53 binding protein homolog (mouse)	Hs.484551	9.18	17.21
MEN1	multiple endocrine neoplasia I	Hs.423348	7.59	3.73
MKI67	antigen identified by monoclonal antibody Ki-67	Hs.80976	11.4	9.21
MLF1	myeloid leukemia factor 1	Hs.85195	41.61	12.64
MYC	v-myc myelocytomatosis viral oncogene homolog (avian)	Hs.202453	4.77	8.46
NASP	nuclear autoantigenic sperm protein (histone-binding)	Hs.319334	27.85	3.79
NCAPD3	non-SMC condensin II complex, subunit D3	Hs.438550	19.03	3.18
NCOR1	nuclear receptor corepressor 1	Hs.462323	5.8	8.59

NEDD1	neural precursor cell expressed, developmentally down-regulated 1	Hs.728880	264.98	2.98
NEDD9	neural precursor cell expressed, developmentally down-regulated 9	Hs.673866	4.83	41.46
NEK6	NIMA (never in mitosis gene a)-related kinase 6	Hs.197071	5.6	7.15
NOLC1	nucleolar and coiled-body phosphoprotein 1	Hs.523238	19.91	3.04
NUDC	nuclear distribution gene C homolog (A. nidulans)	Hs.263812	3.32	3.75
NUP43	nucleoporin 43 kDa	Hs.510375	15.27	7.01
NUSAP1	nucleolar and spindle associated protein 1	Hs.615092	56.33	3.09
PBK	PDZ binding kinase	Hs.104741	42.19	5.96
PDPN	podoplanin	Hs.468675	8.03	2.99
PES1	pescadillo homolog 1, containing BRCT domain (zebrafish)	Hs.517543	5.19	8.29
PEX11A	peroxisomal biogenesis factor 11 alpha	Hs.31034	7.14	2.75
PINX1	PIN2/TERF1 interacting, telomerase inhibitor 1	Hs.490991	6.22	3.55
PLK1	polo-like kinase 1	Hs.592049	8.87	26.86
PLK2	polo-like kinase 2	Hs.398157	10.32	3.05
PPM1G	protein phosphatase, Mg2+/Mn2+ dependent, 1G	Hs.643951	5.06	3.98
PPP1CA	protein phosphatase 1, catalytic subunit, alpha isozyme	Hs.183994	2.77	4.67
PPP1CC	protein phosphatase 1, catalytic subunit, gamma isozyme	Hs.79081	26.4	5.29
PPP1R15A	protein phosphatase 1, regulatory subunit 15A	Hs.631593	13.13	21.42
PPP5C	protein phosphatase 5, catalytic subunit	Hs.654604	10.6	60.52
PRC1	protein regulator of cytokinesis 1	Hs.366401	40	3.55
PSMA1	proteasome (prosome, macropain) subunit, alpha type, 1	Hs.102798	5.32	16.92
PSMA2	proteasome (prosome, macropain) subunit, alpha type, 2	Hs.333786	16.78	3.23
PSMA3	proteasome (prosome, macropain) subunit, alpha type, 3	Hs.558799	55.69	11.77
PSMA4	proteasome (prosome, macropain) subunit, alpha type, 4	Hs.251531	18.56	5.08
PSMA7	proteasome (prosome, macropain) subunit, alpha type, 7	Hs.233952	5.73	6.62
PSMB2	proteasome (prosome, macropain) subunit, beta type, 2	Hs.471441	6.18	2.73
PSMB8	proteasome (prosome, macropain) subunit, beta type, 8 (large multifunctional peptidase 7)	Hs.180062	3.72	5.41
PSMC4	proteasome (prosome, macropain) 26S subunit, ATPase, 4	Hs.211594	5.5	2.91
PSMC5	proteasome (prosome, macropain) 26S subunit, ATPase, 5	Hs.79387	6.29	14.64
PSMC6	proteasome (prosome, macropain) 26S subunit, ATPase, 6	Hs.156171	78.13	18.49
PSMD1	proteasome (prosome, macropain) 26S subunit, non-ATPase, 1	Hs.3887	15.98	4.26
PSMD11	proteasome (prosome, macropain) 26S subunit, non-ATPase, 11	Hs.655396	7.14	5.59
PSMD12	proteasome (prosome, macropain) 26S subunit, non-ATPase, 12	Hs.592689	60.38	17.25
PSMD3	proteasome (prosome, macropain) 26S subunit, non-ATPase, 3	Hs.12970	4.37	71.69
PSMD6	proteasome (prosome, macropain) 26S subunit, non-ATPase, 6	Hs.152536	16.49	3.33
PSME1	proteasome (prosome, macropain) activator subunit 1 (PA28 alpha)	Hs.75348	3.1	14
PSME3	proteasome (prosome, macropain) activator subunit 3 (PA28 gamma; Ki)	Hs.152978	33.65	8.57
PSMF1	proteasome (prosome, macropain) inhibitor subunit 1 (PI31)	Hs.471917	5.52	5.68
PSRC1	proline/serine-rich coiled-coil 1	Hs.405925	8.75	2.9
PTP4A1	protein tyrosine phosphatase type IVA, member 1	Hs.227777	24.42	7.01
RABGAP1	RAB GTPase activating protein 1	Hs.271341	5.71	14.11
RAD1	RAD1 homolog (S. pombe)	Hs.38114	15.19	11.49
RAD21	RAD21 homolog (S. pombe)	Hs.81848	97.44	4.18
RASSF1	Ras association (RalGDS/AF-6) domain family member 1	Hs.476270	4.21	6.52
RBL1	retinoblastoma-like 1 (p107)	Hs.207745	4.12	6.5
RBL2	retinoblastoma-like 2 (p130)	Hs.513609	35.09	11.76
RBM38	RNA binding motif protein 38	Hs.236361	8.45	5.94
RCC1	regulator of chromosome condensation 1	Hs.469723	4.82	7.51
RGS14	regulator of G-protein signaling 14	Hs.9347	4.07	3.05
RGS2	regulator of G-protein signaling 2, 24 kDa	Hs.78944	85.28	11.97
RNF2	ring finger protein 2	Hs.591490	14.76	6.78
RRS1	RRS1 ribosome biogenesis regulator homolog (S. cerevisiae)	Hs.71827	9.79	2.9
RUVBL1	RuvB-like 1 (E. coli)	Hs.272822	11.42	29.09

SART1	squamous cell carcinoma antigen recognized by T cells	Hs.502883	3.27	3.17
SBDS	Shwachman-Bodian-Diamond syndrome	Hs.110445	16.18	14.81
SEH1L	SEH1-like (S. cerevisiae)	Hs.301048	53.18	23.42
SESN2	sestrin 2	Hs.469543	4.33	5.69
SESN3	sestrin 3	Hs.659934	18.67	10.9
SETD8	SET domain containing (lysine methyltransferase) 8	Hs.443735	9.47	5.28
SF1	splicing factor 1	Hs.502829	2.75	11.35
SIAH1	seven in absentia homolog 1 (Drosophila)	Hs.713615	12.44	8.57
SIRT2	sirtuin 2	Hs.466693	7.83	11.66
SKP1	S-phase kinase-associated protein 1	Hs.171626	8.17	3.79
SKP2	S-phase kinase-associated protein 2 (p45)	Hs.23348	22	10.52
SMC1A	structural maintenance of chromosomes 1A	Hs.211602	4.89	12.4
SPIN1	spindlin 1	Hs.146804	28.73	22.71
STEAP3	STEAP family member 3, metalloreductase	Hs.647822	2.81	3.11
STRADA	STE20-related kinase adaptor alpha	Hs.514402	7.59	7.36
SUGT1	SGT1, suppressor of G2 allele of SKP1 (S. cerevisiae)	Hs.281902	9.98	24.8
SUN2	Sad1 and UNC84 domain containing 2	Hs.517622	2.57	22.36
SUV39H1	suppressor of variegation 3-9 homolog 1 (Drosophila)	Hs.522639	2.79	3.21
TARDBP	TAR DNA binding protein	Hs.300624	19.63	15.07
TCF7L2	transcription factor 7-like 2 (T-cell specific, HMG-box)	Hs.593995	3.05	10.34
TERF1	telomeric repeat binding factor (NIMA-interacting) 1	Hs.442707	48.9	24.18
TFDP1	transcription factor Dp-1	Hs.79353	13.37	10.34
TGFB1	transforming growth factor, beta 1	Hs.645227	2.5	6.91
TOP1	topoisomerase (DNA) I	Hs.472737	19.57	37.38
TP53	tumor protein p53	Hs.654481	6.41	67.06
TP73	tumor protein p73	Hs.192132	6.93	16.56
TPD52L1	tumor protein D52-like 1	Hs.591347	16.23	4.75
TPX2	TPX2, microtubule-associated, homolog (Xenopus laevis)	Hs.244580	24.55	9.92
TRIP13	thyroid hormone receptor interactor 13	Hs.436187	12.2	5.23
TUBB	tubulin, beta class I	Hs.636480	4.38	2.74
UBE2I	ubiquitin-conjugating enzyme E2I	Hs.302903	2.89	9.89
UHMK1	U2AF homology motif (UHM) kinase 1	Hs.127310	7.59	13.98
UHRF2	ubiquitin-like with PHD and ring finger domains 2	Hs.493401	10.62	12.92
WEE1	WEE1 homolog (S. pombe)	Hs.249441	16.22	3.18
WTAP	Wilms tumor 1 associated protein	Hs.446091	11.81	74.84
ZNF655	zinc finger protein 655	Hs.599798	34.53	4.46
ZNF830	zinc finger protein 830	Hs.413678	54.08	4.81
ZWINT	ZW10 interactor	Hs.591363	23.59	6.03

Supplementary Table S6: List of antibodies used in this study

Antibody name	Vender[a]	ID	Purpose[b]
anti-TIA1	Santa Cruz Biotechnology	C-20	IHC (1:500 dilution)/ FIC (1:500 dilution)/ Western blotting
anti-TIA1	Medical & Biological Laboratories	RN014P	RIP
anti-Ki-67	DAKO	MIB-1	IHC (1:100 dilution)/ FIC (1:100 dilution)
anti-SKP2	Cell Signaling Technology	D3G5	IHC[a] (1:500 dilution)/ Western blotting
anti-CCNA2	Novocastra	6E6	IHC (1:100 dilution)
anti-CCNA2	Cell Signaling Technology	BF683	Western blotting
anti-p27	Santa Cruz Biotechnology	C-19	Western blotting
anti-hnRNPC1/C2	Santa Cruz Biotechnology	H-105	Western blotting
anti-Chk1	Santa Cruz Biotechnology	G-4	Western blotting

anti-Chk2	Santa Cruz Biotechnology	B-4	Western blotting
anti-GAPDH	Santa Cruz Biotechnology	6C5	Western blotting
anti-CASP3	Cell Signaling Technology	9662	Western blotting
anti-CASP7	Cell Signaling Technology	9492	Western blotting
anti-PARP	Cell Signaling Technology	9542	Western blotting
anti-β-tubulin	Cell Signaling Technology	9F3	Western blotting
anti-FLAG	Sigma	M2	FIC(1:20000 dilution)/ Western blotting
anti-p21 antibody	Medical & Biological Laboratories	K0081-3	Western blotting
Alexa Fluor 488-labeled goat anti-mouse	Molecular Probes		FIC(1:500 dilution)
Alexa Fluor 594 goat anti-rabbit	Molecular Probes		FIC(1:500 dilution)
Alexa Fluor 594 donkey anti-goat	Molecular Probes		FIC(1:500 dilution)

[a]Santa Cruz Biotechnology, Santa Cruz, CA, USA; Medical & Biological Laboratories, Nagoya, Japan; DAKO, Glostrup, Denmark; Cell Signaling Technology, Danvers, MA, USA; Novocastra, Newcastle, UK; Sigma, St. Louis, MO, USA; Molecular Probes, Eugene, OR, USA.
[b]IHC, immunohistochemistry; FIC, fluorescence immunocytochemistry; RIP, RNA immunoprecipitation.

Supplementary Table S7: List of primer sets used in PCR and qPCR

Gene name		Sequence
**qPCR**		
GAPDH	Forward	5′-AGCCACATCGCTCAGACAC-3′
	Reverse	5′-GCCCAATACGACCAAATCC-3′
18S rRNA	Forward	5′-GGCCCTGTAATTGGAATGAGTC-3′
	Reverse	5′-CCAAGATCCAACTACGAGCTT-3′
TIA1 (total)	Forward	5′-GGACGGAAGATAATGGGTAAGGAAG-3′
	Reverse	5′-CTGAGATCACCAACAAAGACATGG-3′
TIA1 variant 1	Forward	5′-GGACGGAAGATAATGGGTAAGGAAG-3′
	Reverse	5′-CAAAGACATGGAAATGATTGCTTGTATC-3′
TIA1 variant 2	Forward	5′-GGACGGAAGATAATGGGTAAGGAAG-3′
	Reverse	5′-CTGACAACGGTACTACTGCTTGTATC-3′
CCNA2	Forward	5′-GCCAGTGAGTGTTAATGAAGTACC-3′
	Reverse	5′-TCCACGAGGATAGCTCTCATACTG-3′
CHK2	Forward	5′-TGAGGCTGCGGAGAGTGT-3′
	Reverse	5′-GACTCCCGAGACATCACGAC-3′
CHK1	Forward	5′-CAACAAACCCCTCAAGAAAGG-3′
	Reverse	5′-TGGATTGAATGTGCTTAGAAAATC-3′
CDK6	Forward	5′-TGATCAACTAGGAAAAATCTTGGA-3′
	Reverse	5′-GGCAACATCTCTAGGCCAGT-3′
CCND1	Forward	5′-GCCTCTAAGATGAAGGAGACCATC-3′
	Reverse	5′-GTTCCACTTGAGCTTGTTCACC-3′
FAS	Forward	5′-GTATGTGAACACTGTGACCCTTG-3′
	Reverse	5′-AAGCCACCCCAAGTTAGATCTG-3′
MAPK1	Forward	5′-TGGTACAGGGCTCCAGAAATTATG-3′
	Reverse	5′-CAGCTGGTCAAGATAATGCTTCCC-3′

MAP2K1	Forward	5′-CTGACATATCTGAGGGAGAAGCAC-3′
	Reverse	5′-CCAAAGTCACAGAGCTTGATCTCC-3′
SKP2	Forward	5′-CCTTTCTGGGTGTTCTGGATTCTC-3′
	Reverse	5′-GCCACCTGTACATGCTTTTCAG-3′
TFDP1	Forward	5′-ACGTCTAACGGCACAAGGTT-3′
	Reverse	5′-CTGAGACCCATTGGAGCTTG-3′
FLAG_SKP2	Forward	5′-CAGGAATTCATGCACAGGAAGCA-3′
	Reverse	5′-CCATGCCTGACAGCAGTTC-3′
luc2	Forward	5′-AGTTCTTCATGCCCGTGTTG-3′
	Reverse	5′-TTTGCAGCCCTTTCTTGCTC-3′
**Construction of expression plasmids for TIA1**		
Full coding TIA1 for pCMV3-Tag1A	Forward[a]	5′-AAAAGGATCCATGGAGGACGAGATGCCCAAGACTC-3′
	Reverse[b]	5′-AAAAGCGGCCGCTCACTGGGTTTCATACCCTGCCACT-3′
Tagged TIA1 for pMXs-Neo	Forward[c]	5′-AAAAGAATTCATGGATTACAAGGATGACGACGATA-3′
	Reverse[b]	5′-AAAAGCGGCCGCTCACTGGGTTTCATACCCTGCCACT-3′
**Construction of expression plasmids for SKP2**		
	Forward[e]	5′-AAAAGAATTCATGCACAGGAAGCACCTCC-3′
	Reverse[d]	5′-AAAACTCGAGTCATAGACAACTGGGCTTTTG-3′
	Forward[d]	5′-AAAACTCGAGATGGATTACAAGGATGACGACGATA-3′
	Reverse[b]	5′-AAAAGCGGCCGCTCATAGACAACTGGGCTTTTG-3′
**Construction of expression plasmids for CCNA2**		
	Forward[a]	5′-AAAAGGATCCATGTTCGGCAACTCTGCG-3′
	Reverse[d]	5′-AAAACTCGAGTTACAGATTTAGTGTCTCTGGTGGG-3′
	Forward[c]	5′-AAAAGAATTCATGGATTACAAGGATGACGACGATA-3′
	Reverse[d]	5′-AAAACTCGAGTTACAGATTTAGTGTCTCTGGTGGG-3′
**Construction of reporter plasmids for SKP2 3′UTR**		
	Forward[e]	5′-AAAAGCTAGCCAGGAGATATGGGGCATCAA-3′
	Reverse[d]	5′-AAAACTCGAGAGTTTGGAAGTTCTGTATGTTTGA-3′
**Biotin-pulldown analysis[f]**		
CCNA2 5′ UTR	Forward	5′-T7-CCATTTCAATAGTCGCGGGA -3′
	Reverse	5′-CACTGCTCCCGGGAGTGG-3′
CCNA2 CDS	Forward	5′-T7-ATGTTGGGCAACTCTGCG -3′
	Reverse	5′-TTACAGATTTAGTGTCTCTGGTGGG-3′
CCNA2 3′ UTR	Forward	5′-T7-CAATGAAAGACTGCCTTTGTTTT -3″
	Reverse	5′-AAGGTAACAAATTTCTGGTTTATTTCA-3′
SKP2 5′ UTR	Forward	5′-T7-AATTCCCAGCAGGCCTT -3′
	Reverse	5′-AGCGTCCGCAGGCCCG-3′
SKP2 CDS	Forward	5′-T7-ATGCACAGGAAGCACCTC -3′
	Reverse	5′-TCATAGACAACTGGGCTTTTGC-3′
SKP2 3′ UTR	Forward	5′-T7-CAGGAGATATGGGGCATCAA -3′
	Reverse	5′-AGTTTGGAAGTTCTGTATGTTTGA -3′

[a]*Bam*HI site is underlined.
[b]*Not*I site is underlined.
[c]*Eco*RI site is underlined.
[d]*Xho*I site is underlined.
[e]*NheI* site is underlined.
[f]T7, T7 RNA polymerase promoter sequence: CCAAGCTTCTAATACGACTCACTATAGGGAGA.

Supplementary Table S8: List of siRNAs for silencing target genes

Gene name	siRNA name	Vender	ID	Sequence
TIA1 (all)	#1	QIAGEN		5′-GGGCUAACAGAACAACUAATT-3′
	#2	Ambion	s14131	5′-GCGUCAGACUUUUUCACCATT-3′
	#3	Ambion	s14133	5′-CGCUCCAAAGAGUACAUAUTT-3′
TIA1a (*TIA1* variant 2)	#1	Sigma-Aldrich		5′-CACACAGCGUUCACAAGAUTT-3′
	#2	Sigma-Aldrich		5′-GCGUUCACAAGAUCAUUUCTT-3′
TIA1b (*TIA1* variant1)		Sigma-Aldrich		5′-GCAAUCAUUUCCAUGUCUUTT-3′
CCNA2	#1	Ambion	s2513	5′-GAUAUACCCUGGAAAGUCUTT-3′
	#2	Ambion	s2514	5′-GGAUGGUAGUUUUGAGUCATT-3′
SKP2		SantaCruz	sc-36499	consist of pools of three to five target-specific siRNAs
control siRNA		QIAGEN	SI03650318	AllStars Negative Control siRNA
		Ambion	4390846	Silencer Select Negative Control #2
		Sigma-Aldrich	SIC-001	Universal Negative Control #1
